# The lipidomic architecture of the mouse brain

**DOI:** 10.1038/s41586-026-11050-0

**Published:** 2026-09-23

**Authors:** Luca Fusar Bassini, Halima Hannah Schede, Laura Capolupo, Leila Haj Abdullah Alieh, Irmak Kaysudu, Francesca Venturi, Hannah Hochgerner, Alessandro Valente, Colas Droin, Daniel Trejo Banos, Irina Khven, Jean Andrea Maillat, Anne-Laure Mahul-Mellier, Antonino Asaro, Doğukan H. Ülgen, Pavel Barahtjan, Ece Z. Asirim, Anita Nasrallah, Carmen Sandi, Ekaterina Krymova, Giovanni D’Angelo, Gioele La Manno

**Affiliations:** 1 Laboratory of Brain Development and Biological Data Science, Brain Mind Institute, Faculty of Life Sciences, École Polytechnique Fédérale de Lausanne (EPFL), Lausanne, Switzerland; 2 Lipid Cell Biology Laboratory, Institute of Bioengineering and Global Health Institute, Faculty of Life Sciences, École Polytechnique Fédérale de Lausanne (EPFL), Lausanne, Switzerland; 3 The Laboratory of Single Cell Molecular Neurobiology, Technion, Haifa, Israel; 4 Swiss Data Science Center, Zurich, Switzerland; 5 Laboratory of Behavioral Genetics, Brain Mind Institute, Faculty of Life Sciences, École Polytechnique Fédérale de Lausanne (EPFL), Lausanne, Switzerland; 6 Present Address: Implexis, London, UK; 7 Present Address: Department of Biosystems Science and Engineering, ETH Zürich, Basel, Switzerland; 8 Present Address: Laboratory of Computational, Quantitative and Synthetic Biology - Sorbonne University, Paris, France; 9 Present Address: Hydroclimat, Aubagne, France; 10 Present Address: Centre for Artificial Intelligence, ZHAW School of Engineering, Winterthur, Switzerland; 11 Present Address: UCB Farchim, Bulle, Switzerland

**Keywords:** Lipidomics, Molecular neuroscience, Cellular neuroscience, Multicellular systems

## Abstract

Lipids are fundamental components of the brain, crucial for synaptic transmission and signal propagation. Altered brain lipid composition is associated with common and rare neuropathologies, yet the spatial organization of the mammalian brain lipidome remains insufficiently characterized compared with other modalities^1–6^. Here we mapped the membrane-lipid architecture of the adult mouse brain at micrometric scale, across sexes and during pregnancy. This lipid brain atlas reveals that lipids describe a fine-grained biochemical structure that aligns with functional anatomy. Membrane-lipid spatial heterogeneity clusters into territories, which we termed ‘lipizones’. Lipizones partially mirror cell-type territories, but also capture distal axon terminals. Through lipizones, we (1) reveal the organizing principles of the grey matter lipidome, related to connectivity and cytoarchitecture; (2) discover a new axis of oligodendrocyte heterogeneity in the white matter; and (3) find biochemical zonation in the choroid plexus and in the ventricular walls. We show that this lipidomic architecture can adapt to changing physiological needs. In the brain of pregnant female mice, the white matter is metabolically activated and the cortex undergoes a lipizone-specific remodelling that is particularly pronounced in layer 4. These results are a foundational resource (https://lbae-v2.epfl.ch/) poised to reshape the understanding of lipids in brain development, physiology and pathology.

## Abstract (web summary)

A spatial lipidomic atlas of the mouse brain reveals more than 500 biochemical territories that mirror cell types and connectivity, capture oligodendrocyte heterogeneity and ventricular zonation, and are remodelled during pregnancy.

## Main

Lipids account for most of the brain dry weight^7^. They form the cell membranes that constitute myelin, axons, dendrites and synapses. Seminal studies have examined the lipid composition of the mammalian brain across anatomical structures and cell classes^8–12^. However, a systematic survey of the brain lipid metabolic architecture^13^ in relation to cell-type composition, functional anatomy, connectivity and physiological variation is lacking.

To fill this gap, we used matrix-assisted laser desorption/ionization mass spectrometry imaging (MALDI–MSI), previously used to map brain lipids in two-dimensional (2D) and three-dimensional (3D) imaging^11,12,14^. We mapped the distribution of 172 lipids across 109 brain sections from 11 mice, covering the entire brain volume (5 µm laser spot size and 25 µm interpixel distance, thus sampling portions of one to four cells per pixel). We identified 539 lipidome-defined brain clusters, which we termed ‘lipizones’. We characterized lipizones in relation to anatomy, cell-type composition, cell compartments, connectivity and biochemistry. We investigated inter-individual and sex-related differences and, to probe physiological variation, charted the spatial lipidome in pregnant mice.

## A 3D lipidomic atlas of the mouse brain

**Fig. 1:**
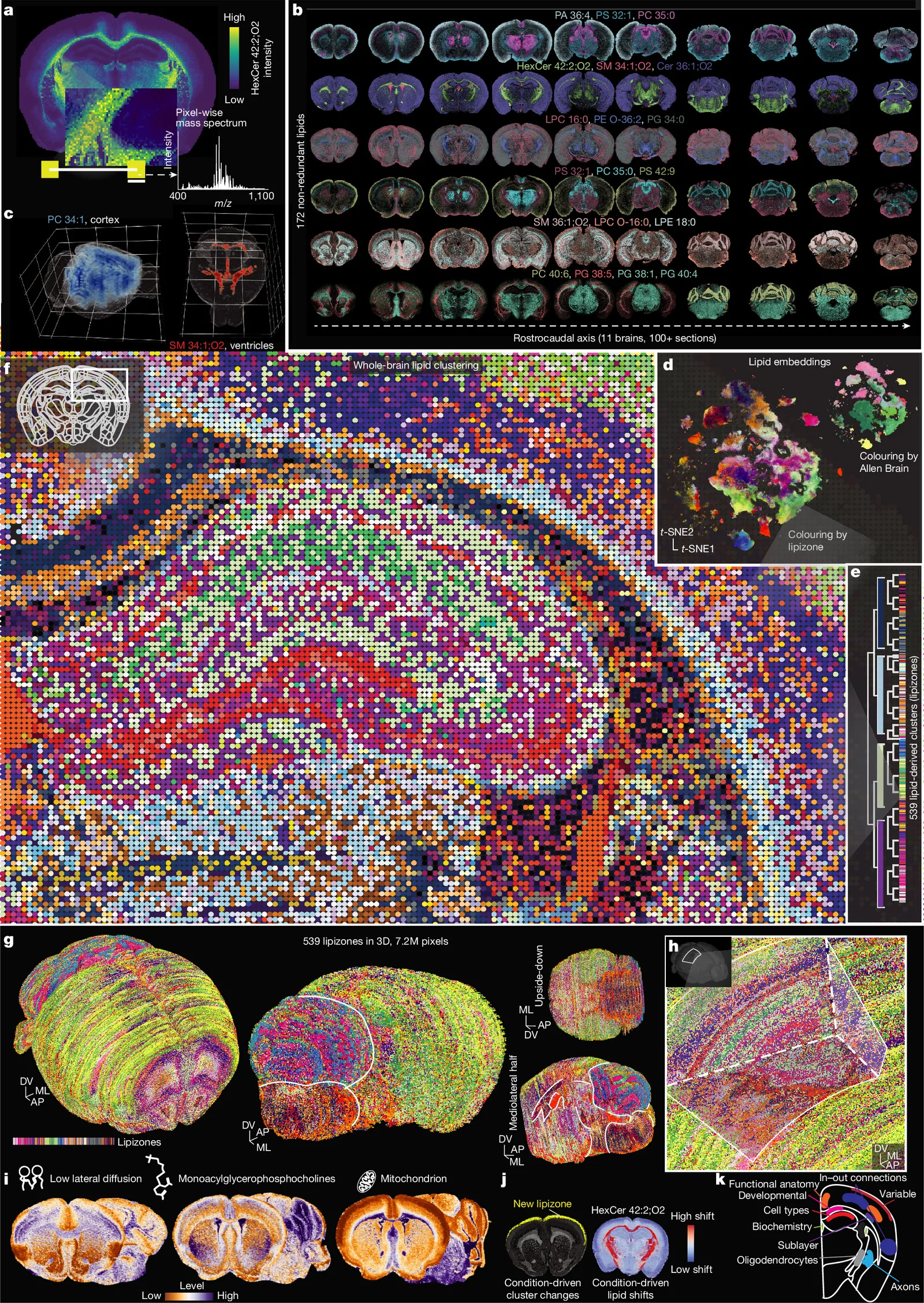
The mouse lipid brain atlas. **a**, Schematic of a MALDI–MSI experiment measuring the mass spectrum of individual 5 µm desorption points (pixels), 25 µm interpixel distance, along an adult brain coronal section; HexCer 42:2;O2 distribution is displayed as an example. **b**, Overview of MALDI–MSI composite images for selected annotated lipids along the rostrocaudal axis. **c**, Three-dimensional distributions of selected lipids obtained interpolating serial sections. **d**, *t*-Distributed stochastic neighbour embedding (*t*-SNE) of pixels in lipid space, coloured by lipizone and by Allen Brain region. **e**, Thumbnail of the (truncated) hierarchical tree of lipizones. **f**, Zoom-in showing individual MALDI–MSI pixels as dots, coloured by lipizone. Dots are enlarged for visualization purposes. **g**, Whole-brain 3D visualizations of pixels coloured by their corresponding lipizone. AP, antero-posterior; DV, dorso-ventral; ML, medio-lateral. **h**, Close-up revealing the lipizone structure near the hippocampal formation. **i**, Spatial distributions for different lipid programme scores estimated by the lipiMap algorithm. **j**, Example of comparative results of lipidomic variations enabled by the atlas. The example shown is physiological variation in pregnancy. **k**, Schematic illustrating the lipidome organizing principles. Scale bars, 25 μm and 5 μm (**a**).

We built a comprehensive lipidomic atlas of the adult mouse brain, measuring coronal sections from male and female brains. Using MALDI–MSI in positive ion mode and the unified Mass Imaging Analyser (uMAIA)^15^, we mapped 26,874 peaks across 7.2 million pixels (Fig. 1a,b and Supplementary Fig. 1a–o; Methods), which were warped into the Allen Brain Atlas (ABA) Common Coordinate Framework (CCF) (ABA CCFv.3 (ref. ^16^)) for systematic comparison with anatomy and other modalities (Supplementary Fig. 1b–d; Methods). A total of 1,400 peaks passed noise quality control (Supplementary Fig. 1e–j and Supplementary Tables 1 and 2; Methods).

For most downstream analysis, we worked with 172 nonredundant lipids annotated with high confidence (Supplementary Table 2a; Methods). Overall, the relative whole-brain abundances of lipids within classes were consistent with those reported previously by liquid chromatography–mass spectrometry (LC–MS)^9^ at the regional level (Supplementary Fig. 2a–f). Classes phosphatidylglycerol (PG) and phosphatidylinositol (PI) ionise efficiently only in negative-ion mode (Supplementary Fig. 2d); their positive-mode peaks correlate weakly with LC–MS and are possibly isobaric, so the reader should treat these annotations as tentative and draw no biological conclusions from them.

**Extended Data Fig. 1:**
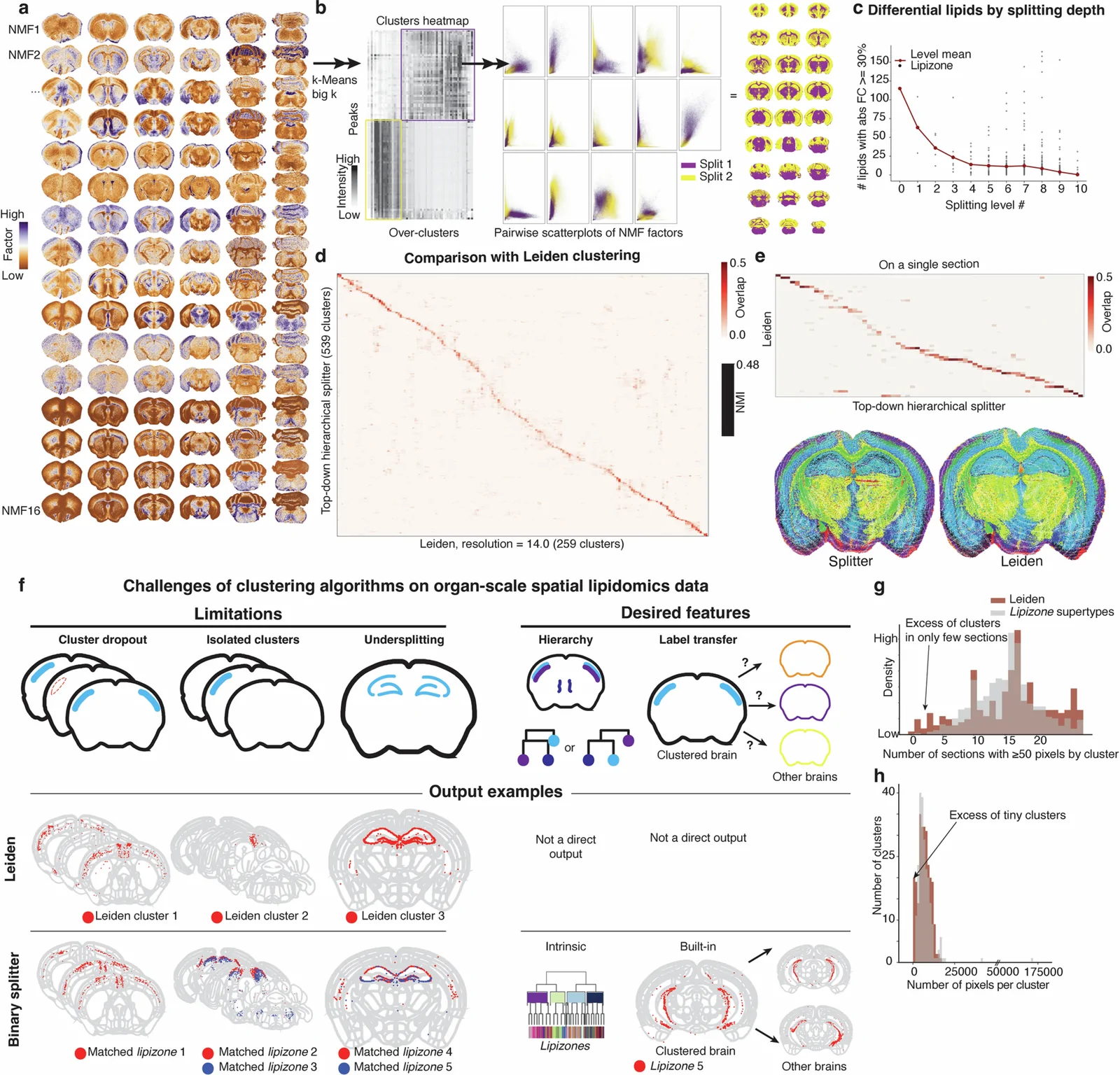
Embedding and clustering methods. (**a**) Spatial plot of the global, harmonized NMF embeddings for representative sections. (**b**) Left: hierarchical binary split procedure illustrated for the first split: heatmap of cluster centroids by m/z peaks sorted by backSPIN for cluster aggregation. The two resulting clusters are highlighted in violet and yellow. Center: scatterplot of the first binary split shown along NMF-pair scatters; right: the same clusters visualized spatially. (**c**) Strip plot of the number of lipids with absolute fold change over 30% by clustering splitting level, with overlaid mean trend. (**d**) Heatmap of sorted, normalized confusion matrix comparing the clustering method used in this study against Leiden clustering, evaluated across the entire brain. The inset shows the NMI score between the two clustering methods. (**e**) Heatmap of sorted, normalized confusion matrix comparing the clustering method used in this study against a Leiden clustering, on a single section, accompanied by color-matched spatial plots, where colors correspond to the clusters obtained from these two clustering strategies. (**f**) Vignette illustrating the challenges of clustering in organ-scale spatial lipidomics, and corresponding data for Leiden and the top-down custom binary splitter that we used. Leiden clustering was found to suffer from cluster dropout (i.e., clusters could skip some sections), isolation of clusters (implausible, given our dense section sampling scheme), and undersplitting (high residual within-cluster variability, that resulted in further splits using the binary splitter). (**g**) Frequency histogram for the number of sections a cluster reliably appears in for the binary splitter and Leiden clustering. (**h**) Frequency histogram of the cluster size variability for the clusters obtained with the binary splitter vs Leiden clustering.

We first analysed sections from two densely sampled male brains (hereafter, ‘brain 1’ as a reference and ‘brain 2’ for validation, more than 25 sections each). We then extended our analysis across additional sparsely sampled brains: three male, three female and three pregnant (embryonic day (E)13.5) female brains. For embedding and clustering, we used lipids selected for high spatial variability, low dropout and consistency across sections (Supplementary Fig. 1k and Supplementary Table 2b; Methods). To capture the latent structure, we applied non-negative matrix factorization (NMF)^17^, identifying 16 molecular signatures, followed by batch harmonization^18^ (Extended Data Fig. 1a; Methods). We imputed lipids when dropped out^19^(Supplementary Fig. 1l–o; Methods); 3D interpolation yielded a 6-million-pixel 3D map (Fig. 1c; Methods). Subsequent Leiden clustering revealed spatially organized domains aligned with anatomical structures.

A binary hierarchical splitting algorithm refined clusters iteratively using local and global lipidomic variability (Fig. 1d–h and Extended Data Fig. 1b,c; Methods). The resulting clusters agreed with Leiden clustering but were more robust to batch effects and revealed finer substructure (Extended Data Fig. 1d–h). The algorithm trains classifiers to validate the learnt splits and can transfer them onto the other brains (Supplementary Fig. 3a–g; Methods). The clustering was validated across 11 brains (biological replicates) in terms of spatial correlation (Supplementary Fig. 4a–d). A total of 27 alternative clustering regimes were also tested and compared quantitatively (Supplementary Fig. 4a,c); the main discoveries were replicable across them (Supplementary Fig. 4b and Supplementary Data File 2). Our collection of methods is available in the Enhanced uMAIA for Clustering Lipizones, Imputation, and Differential analysis (EUCLID) package (Fig. 1a–k; Methods). The Lipid Brain Atlas can be explored at https://lbae-v2.epfl.ch/.

## A lipid axis of brain architecture

**Extended Data Fig. 2:**
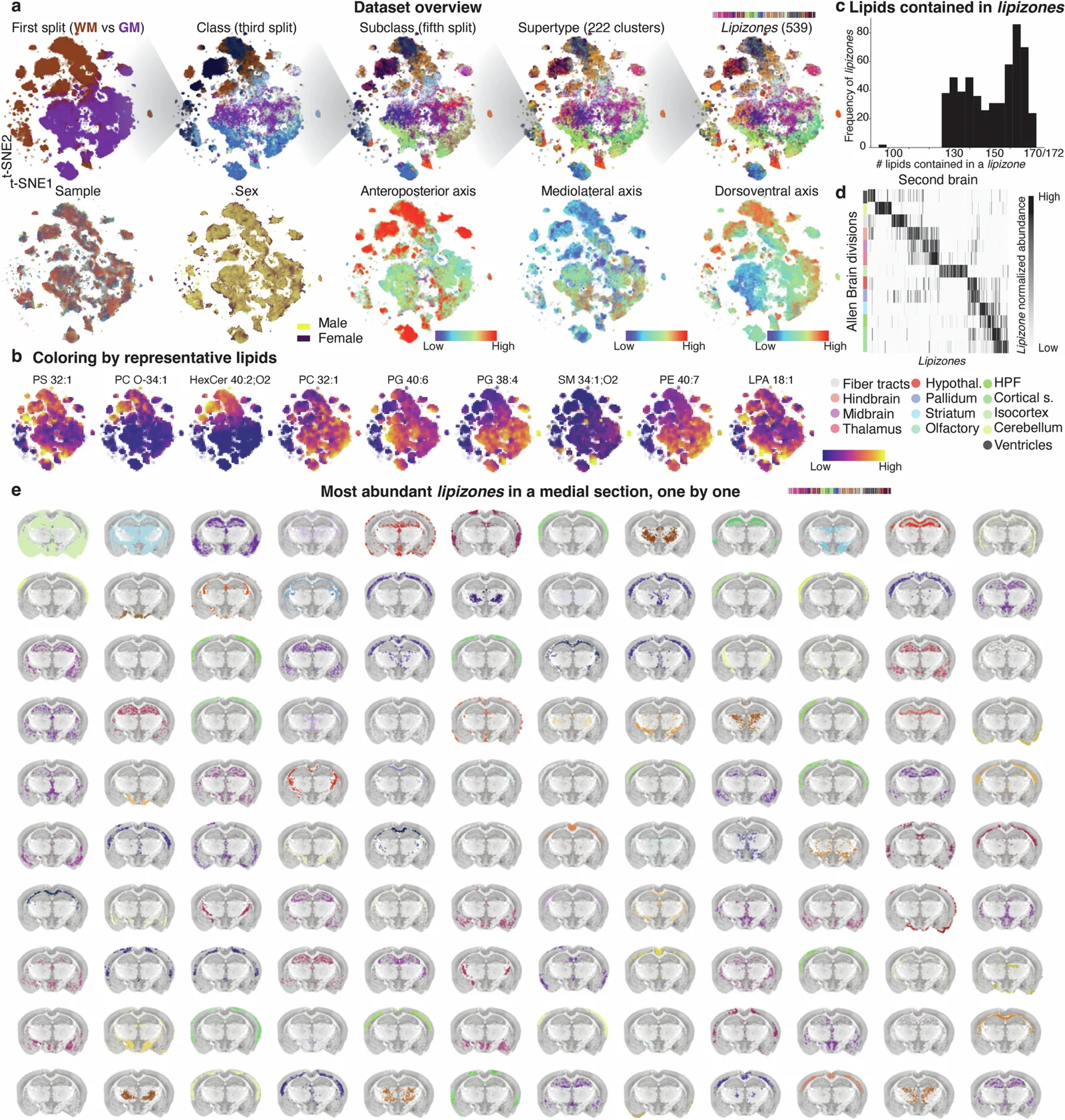
Dataset overview in feature space and physical space. (**a**) tSNEs colored by relevant clustering levels and metadata. (**b**) tSNEs colored by representative lipids. (**c**) Frequency histogram for the number of lipids contained in a *lipizone* across *lipizones*. (**d**) Heatmap replicating Fig. 2e on brain #2, sorted by brain #1, representing *lipizone* normalized abundances by Allen Brain division. (**e**) Spatial plot of the most abundant *lipizones* in a single section, shown individually, color-coded.

**Extended Data Fig. 3:**
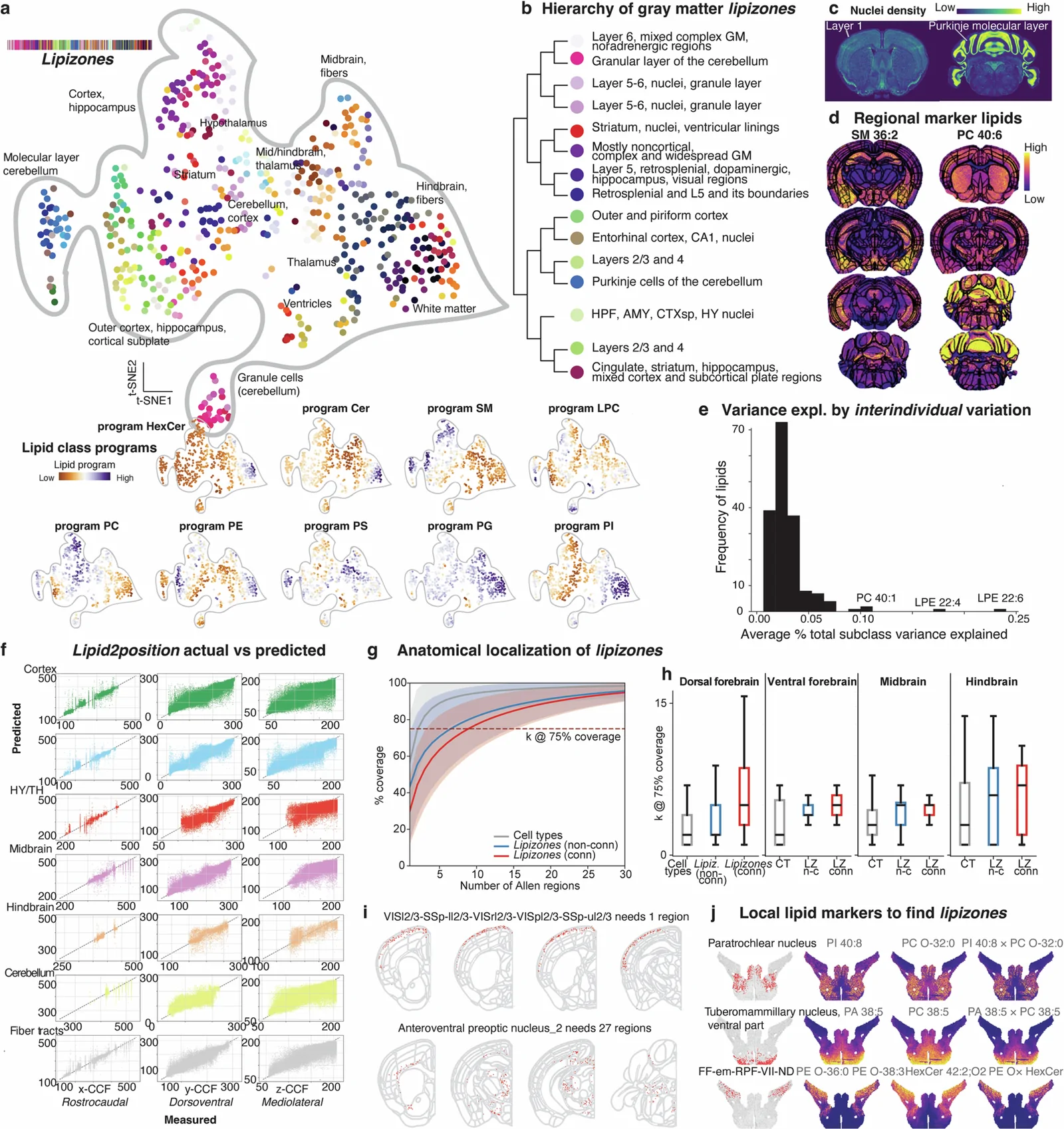
Organization of *lipizones* in terms of lipid classes and compared to brain anatomy. (**a**) t-SNEs of *lipizone* centroids, colored by *lipizones* and by the lipid programs representing lipid classes. (**b**) Dendrogram showing the early lipidomic splits in the gray matter-rich branch, manually labelled with anatomy-based names. (**c**) Representative Allen Brain nuclei density images showing low nuclei density in outer cortex and Purkinje molecular layer. (**d**) Spatial plots of two example regional marker lipids, SM 36:2;O2 (cortical subplate and hippocampal formation) and PC 40:6 (cerebellum), for four representative sections along the anteroposterior axis each. A contour is overlaid, highlighting the CCF registration quality. (**e**) Frequency histogram for the average fraction of total subclass variance explained by inter-individual variation, across lipids. (**f**) Pairwise scatterplots for the ground truth and lipidome-reconstructed 3D spatial coordinates by the “*Lipid2position*” model (1 column per brain axis); one point is a brain pixel; subplots are organized and colored by ABA division. (**g**) Cumulative distribution of the number of anatomical regions needed to cover increasing fractions of *lipizone* or cell type area, averaged across *lipizones* (subdivided by whether they are connectivity-related or not) and Allen Brain Atlas cell supertypes. The line represents the mean; the shaded area encompasses ± 1 s.d. The dashed dark red line marks 75% area coverage. (**h**) Box plots showing, for each brain division, the number of ABA regions needed to cover 75% of *lipizone* (or cell type) area. (**i**) Examples of *lipizones* at the extremes of the cumulative distribution saturation: a lipizone contained within a single Allen region and a lipizone spanning 27 regions to cover 75% of its area. (**j**) Spatial plots of representative hypothalamic *lipizones* (red on grey) alongside their local marker lipids, illustrating how such *lipizones* can be recognized in localized studies.

The 539 terminal lipizones (Extended Data Fig. 2a–e, Supplementary Figs. 5a and 6a,b and Extended Data Fig. 3a–j) were structured hierarchically into 8 classes (splitting level 3), 31 subclasses (level 5) and 222 supertypes (level 8). Lipizones exhibited symmetry along the medio-lateral axis, and all but two contained at least 74% of the 172 profiled lipids at detectable levels (Extended Data Fig. 2c).

**Fig. 2:**
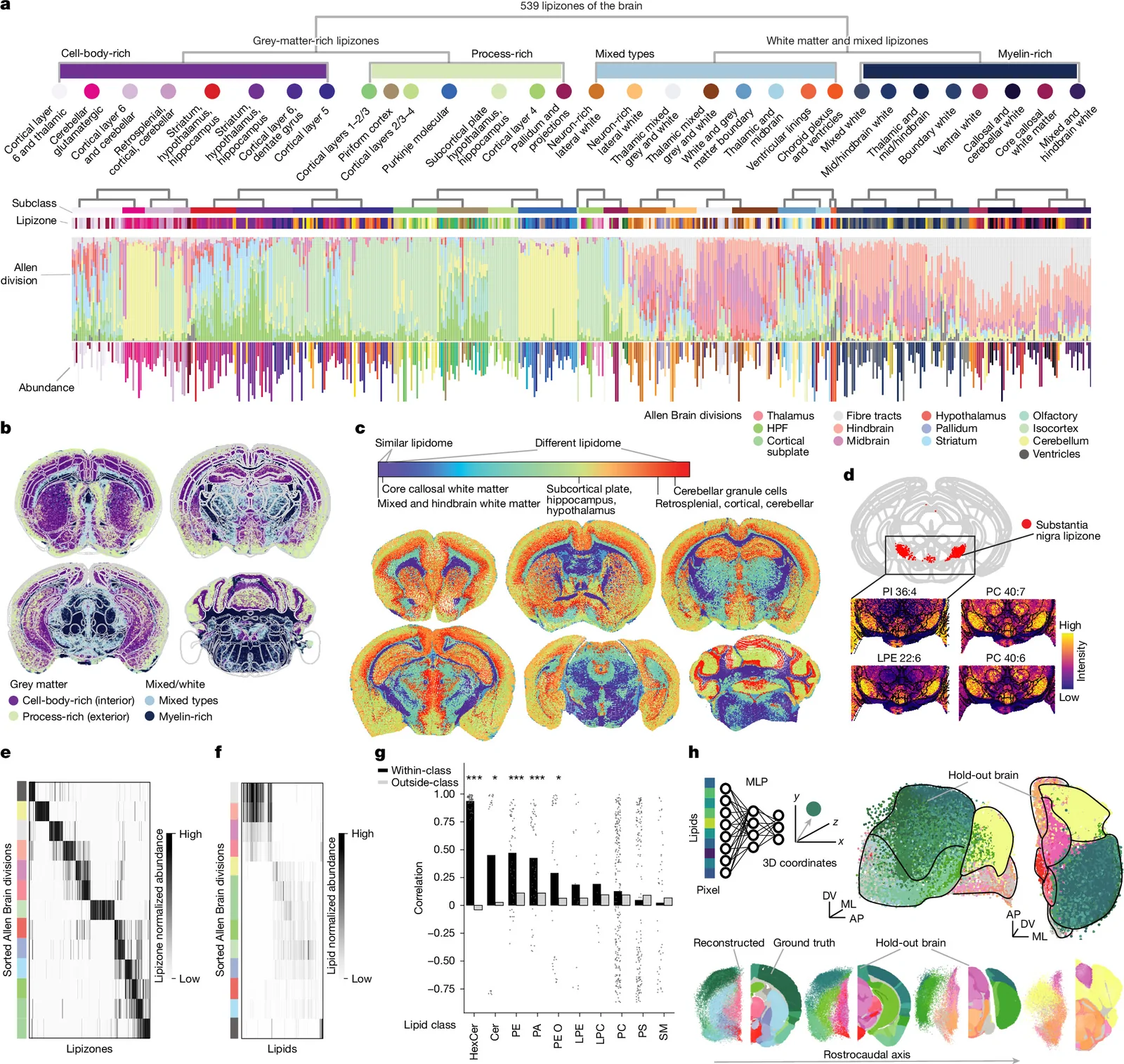
Comparison of lipizones and brain anatomy. **a**, Summary visualization of the lipizones of the brain, including an annotated dendrogram of the 31 lipid-driven subclasses. For each lipizone, we display the distribution of its pixels across anatomical divisions and their abundance across the entire brain (highest values clipped). **b**, Sections coloured by the four main lipidomic subdivisions of the brain. **c**, Global lipidomic patterns. Sections with pixels coloured by lipidomic similarity; similar colour indicates similar lipidome. **d**, Highlight on a lipizone that co-localizes with the substantia nigra, with the distribution of its local lipid markers. **e**, Lipids combinatorially recapitulate anatomy. Heatmap of lipizone normalized abundance by Allen division. **f**, Heatmap of lipid normalized abundance by Allen division. **g**, Limited within-class correlation; bar plots showing the average Pearson’s correlation between lipids of the same class and of different classes (*n* = 172 lipids; one-sided Mann–Whitney *U* test, within-class versus between-class, *q* for BH-FDR = 0.05, ***q < 0.001, **q < 0.01, *q < 0.05). Individual lipid–lipid correlations are overlaid as a strip plot. PA, phosphatidic acid. **h**, Summary of the analysis of pixel localization prediction based on lipidome. Top left, schematic of the approach; top right, 3D visualization of an entirely held-out brain; bottom, single section visualizations. MLP, multi-layer perceptron. Across all panels, pixels are coloured according to their ground truth Allen Brain colour.

The primary division in the hierarchy separated grey and white-matter-rich lipizones (Fig. 2a). White matter lipizones localized to fibre tracts, hindbrain and midbrain, as well as to the thalamus and hypothalamus, and could be divided further into lipizones associated with oligodendrocyte-rich, neuron-poor regions and those linked to areas receiving extensive neuronal input.

Within the grey matter, lipizones were organized following an interior–exterior pattern (Fig. 2b). The interior group comprised predominantly anatomical regions enriched in glutamatergic neurons, whereas the exterior group included areas with abundant GABAergic populations (Extended Data Fig. 3b and Supplementary Table 3).

The sequential formation of layers during brain development^20^ may explain the early bifurcation between deep- and mid-surface cortical layers. However, the lipidomic similarity between cerebellar Purkinje arborization and outer cortex, as well as between granule cells and inner cortex, remained unexplained. This subdivision was driven by myelin lipids (hexosylceramides (HexCers) and ceramides (Cers)), which are more abundant near fibre tracts and arbor vitae, that is, in the inner cortical layers and the granule cells of the cerebellum. The relative enrichment of excitatory synaptic membranes^21^, particularly in areas with few cell bodies (Extended Data Fig. 3c), such as cortical layer 1 and the Purkinje molecular layer^22^, may contribute to this main structural division.

As the hierarchy showed structured anatomical organization (Fig. 2c), we named lipizones after the Allen Brain regions in which they were most enriched (Fig. 2d and Supplementary Fig. 3f,g; Methods). We provide the lipizones lipidomes as Supplementary Table 4. Terminal lipizones showed a strong bidirectional association with the 13 main divisions of the Allen Brain Atlas (Fig. 2e and Extended Data Fig. 2d), a pattern exposed only partially by individual lipids (Fig. 2f).

Exceptions were phosphatidylethanolamine (PE)-O 38:7, restricted to the isocortex; sphingomyelin (SM) 36:2;O2, localized to the amygdala and the hippocampus; SM 34:1;O2 and SM 38:1;O2, confined to the ventricles and phosphatidylcholine (PC) 40:6, enriched in the cerebellum (Extended Data Fig. 3d). The scarcity of single-lipid markers probably reflects the fact that distinct metabolic states modulate the relative abundance of membrane lipids, rather than producing on–off patterns.

Many lipid species diverged spatially from other members of their class, especially for lysophosphatidylcholine (LPC), lysophosphatidylethanolamine (LPE), PC, phosphatidylserine (PS) and SM (no difference in within- versus between-class spatial correlation; Benjamini–Hochberg (BH) false discovery rate (FDR) (BH-FDR)-corrected one-sided Mann–Whitney, FDR = 0.05; Fig. 2g and Supplementary Fig. 2e,f), indicating that future analyses should preserve individual-species granularity where possible.

When examining inter-individual variation in the three sparsely sampled male and three female animals, we found that, for 90% of the lipids, 1.1% to 6.3% of the total subclass variance could be attributed to individual differences (*P* < 0.05 for 97.6% lipid-subclass pairs, one-sided *F*-test for excess variance; Extended Data Fig. 3e and Supplementary Methods).

Lipizones often recapitulated specific tissue niches, for example, the substantia nigra reticulata, marked distinctly by several phospholipids, including LPE 22:6, PC 40:7 and PC 40:6 (Fig. 2d). Although whole-brain lipid markers are scarce, region-specific markers can be identified (Extended Data Fig. 3j). We provide region-average lipidomes (Supplementary Table 5) and these within-region markers (Supplementary Table 6) to support regionally focused neurometabolic studies. Hippocampus and cortical subplate, which derive developmentally from the pallium and are related transcriptionally^5^, shared several lipizones (Fig. 2e). Similarly, pallidum and striatum, both derived from medial and/or lateral ganglionic eminences of the subpallium^23^, shared many lipizones. This parcellation offers a framework for biochemical stratification (implemented in EUCLID, whose label transfer function maps lipizones onto new samples; Methods).

Having established structured regional heterogeneity, we asked whether lipid distributions encode enough information to predict anatomical location. To test this, we trained a multi-layer perceptron on brain 1 to predict 3D spatial coordinates using only lipid profiles as input. The model generalized well to the entirely held-out brain 2, reconstructing regional boundaries without relying on spatial information (Fig. 2h, Extended Data Fig. 3f and Supplementary Methods).

## Lipids are predicted partly by RNA

**Fig. 3:**
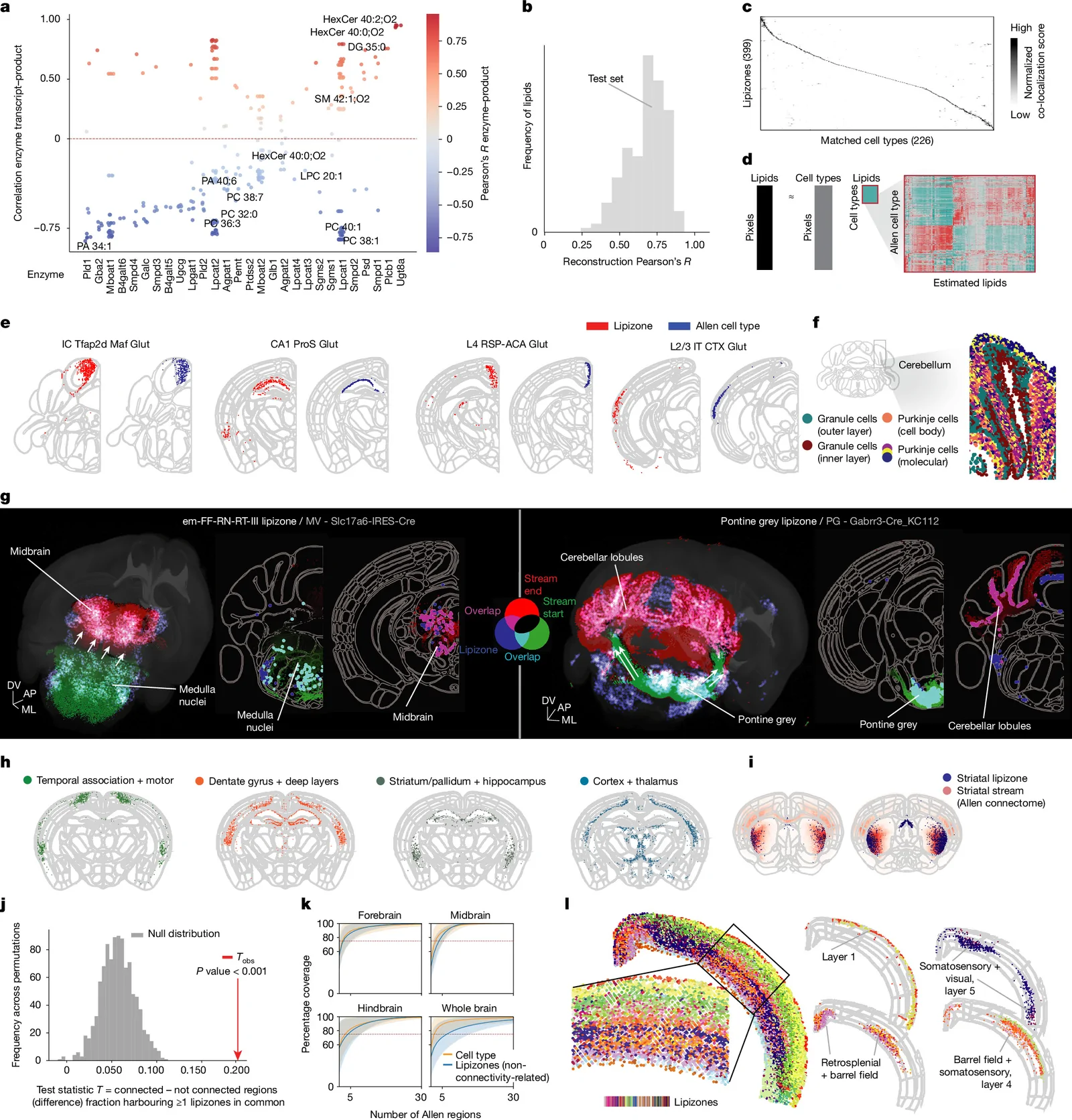
Organizing principles of the brain lipidome. **a**, Strip plot of Pearson correlation between enzyme expression and corresponding lipid products (several per enzyme), computed on cell-type averages (*n* = 338 enzyme–product pairs). **b**, Histogram of reconstruction accuracies (Pearson’s *R*) of lipid level prediction from the transcriptome using XGBoost regression. **c**, Co-localization matrix (reciprocal enrichment) for matching cell-type territories and lipizones. **d**, Schematic of the cell-type lipidome deconvolution method, and miniature heatmap of the cell-type × lipid matrix inferred (Supplementary Fig. 9). **e**, Spatial plots of lipizones and the cell-type territories (cell bodies) with which they co-localize. Cell-type territory names follow the Allen nomenclature. **f**, Spatial zoom-in on the granule cells layer of the cerebellum and its neighbourhood, coloured by lipizones named by location. **g**, Integration of lipizones with cell types and connectivity. Three- and two-dimensional visualizations of two representative lipizones that probably include cell bodies (cyan) and their distal terminals (magenta). Background, connectomic streams (green→black→red); non-overlapping lipizone pixels are in blue. Grey titles, injection site and mouse line. **h**, Spatial plots of selected lipid clusters spanning anatomically distant yet connected brain regions. **i**, Spatial plot showing the overlap between a striatal lipizone and a connectomic stream. **j**, Connected regions share lipizones. Histogram of the permutation null distribution for the difference statistic *T* (connected − not-connected fraction), for the frequency of sharing a lipizone for two Allen anatomical regions. Red arrow, observed *T* value. Exact one-sided *P* value by distance-conditional permutation test (*P* < 0.001; 1,000 permutations). **k**, Anatomical location of lipizones. Cumulative distributions across lipizones (and cell types) showing the percent area covered as a function of Allen regions included; whole-brain and separately for forebrain, midbrain and hindbrain. Lipizones shown are those not related to connectivity; see Extended Data Fig. 3g for full analysis. Mean curve across lipizones (or cell-type supertypes) within each division, with the ±1 s.d. across entities (shaded). **l**, Spatial plots of cortical lipizones. Left, close-up; right, examples, displayed in groups spanning the same anatomical regions.

We next asked whether the lipidome spatial heterogeneity relates to metabolic enzyme expression patterns. We integrated the spatially-resolved brain cell atlases of Yao and colleagues^4^ (Supplementary Fig. 7a) and Langlieb and colleagues^1^ with our lipid atlas based on the CCF registration. We investigated whether enzymes and the membrane lipids they produce are correlated at the cell-type level. We used single-cell RNA sequencing enzyme transcripts as proxies. Except for 81 of 338 enzyme–product pairs correlating *R* > 0.5, enzyme transcripts were not trivially positively correlated (*R* = −0.13 ± 0.57; *P* < 0.0001, two-sided one-sample *t*-test against zero) with their lipid products (Fig. 3a).

Lipid distributions thus cannot be inferred reliably from individual enzyme expression. Reasoning that a more holistic view of the metabolic network might improve predictions, we tested whether supervised models could leverage covariation between metabolic genes and lipid distributions. We used canonical correlation analysis and redundancy analysis with more than 500 Allen Brain MERFISH-imputed metabolism-related genes^4^, finding that metabolic genes linearly captured approximately 36.6% of the total variance in lipid profiles (Supplementary Fig. 8a–e).

Finally, to assess the information relationship between gene expression and lipidome, we quantified the predictability of lipid distributions from the Allen Brain full transcriptome^4^ using XGBoost^19^ to capture non-linear relationships (Supplementary Methods). Broad lipid distributions (*R*^2^ > 0.25) could be predicted for 86.7% of lipids, with 35 of 172 lipids being predicted with good accuracy (*R*^2^ > 0.60) (Fig. 3b). Considering a conservative upper bound on prediction performance of *R*^2^ ≈ 0.81 based on empirically estimated irreducible noise in lipid-to-lipid prediction, the gene-based model (test-set average Pearson’s *R* = 0.68) captured 56% of the explainable lipid variance (Supplementary Methods). These results suggest that the brain lipidome is irreducible to the transcriptome.

Feature importance analysis found that predictive genes for 91% of 251 anatomical regions and 172 lipid pairs were enriched significantly for cell-type markers (one-sided permutation test, 2,000 permutations, BH-FDR < 0.05; Supplementary Fig. 8b,c). This indicates that reliable but imperfect lipid predictions derive from cell-type identity, suggesting that lipid distributions reflect properties intrinsic to cellular lineages.

## Lipizones map cell-type bodies and terminals

MALDI–MSI pixels mix cell bodies, projections and extracellular matrix, all of which may contribute to lipizones. To test how lipizones corresponded to cell-type territories in terms of cell bodies, we measured co-localization at all hierarchical levels by reciprocal enrichment analysis (Supplementary Methods). We found a striking correspondence (Fig. 3c–e): excluding the lipizones whose modal division was fibre tracts (67) or cerebellum (73), 76% (304 of 399; Supplementary Table 7), lipizones discovered by our clustering algorithm overlapped cell-type territories. Specific cell types were rediscovered by our lipid-only clustering method (Fig. 3e and Supplementary Fig. 8d).

**Extended Data Fig. 4:**
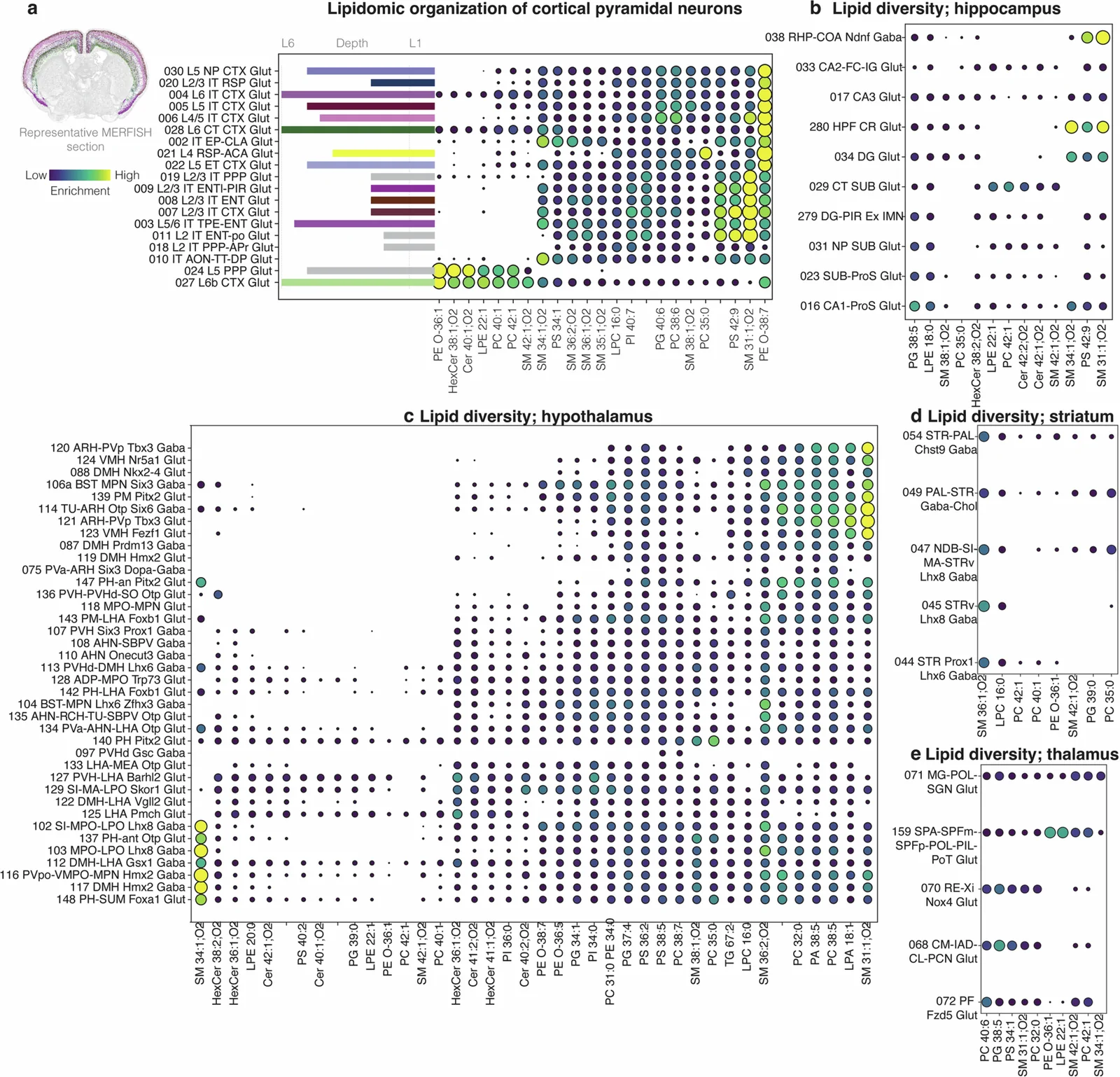
Lipid markers of cell types. (**a**) Dot plot of lipid enrichment scores from the deconvolution analysis for cortical pyramidal neurons (rows) and selected lipids (columns). Horizontal bars indicate the cortical depth of each cell type from layer 6 to layer 1, coloured by Allen MERFISH subclass. Inset: spatial distribution of pyramidal cortical neuron subclasses in a representative MERFISH section, using the same colour code (**b**–**e)**, Dot plots as in (a), showing lipid enrichment for hippocampal (**b**), hypothalamic (**c**), striatal inhibitory (**d**) and thalamic (**e**) neurons.

We used CCF-registered cell-type information to estimate the lipidome of individual cell types by deconvolution (Fig. 3d, Supplementary Fig. 9a–d*,* Extended Data Fig. 4a–e and Supplementary Table 8). Deconvolution-based lipid signatures were broadly consistent with in vitro cell-type signatures from bulk lipidomics in a study by Fitzner and colleagues^9^, yet 8–15% of lipids were discordant per cell type.

We next asked whether similar lipidomic entities appear in single-cell data and other species. We mapped single-cell lipidomics data from adult rat brain^12^ and developing human brain cortex^24^ onto our atlas, finding that lipid profiles aligned with lipizones (Supplementary Fig. 10a–d). This raises the possibility that this organization is conserved.

**Extended Data Fig. 5:**
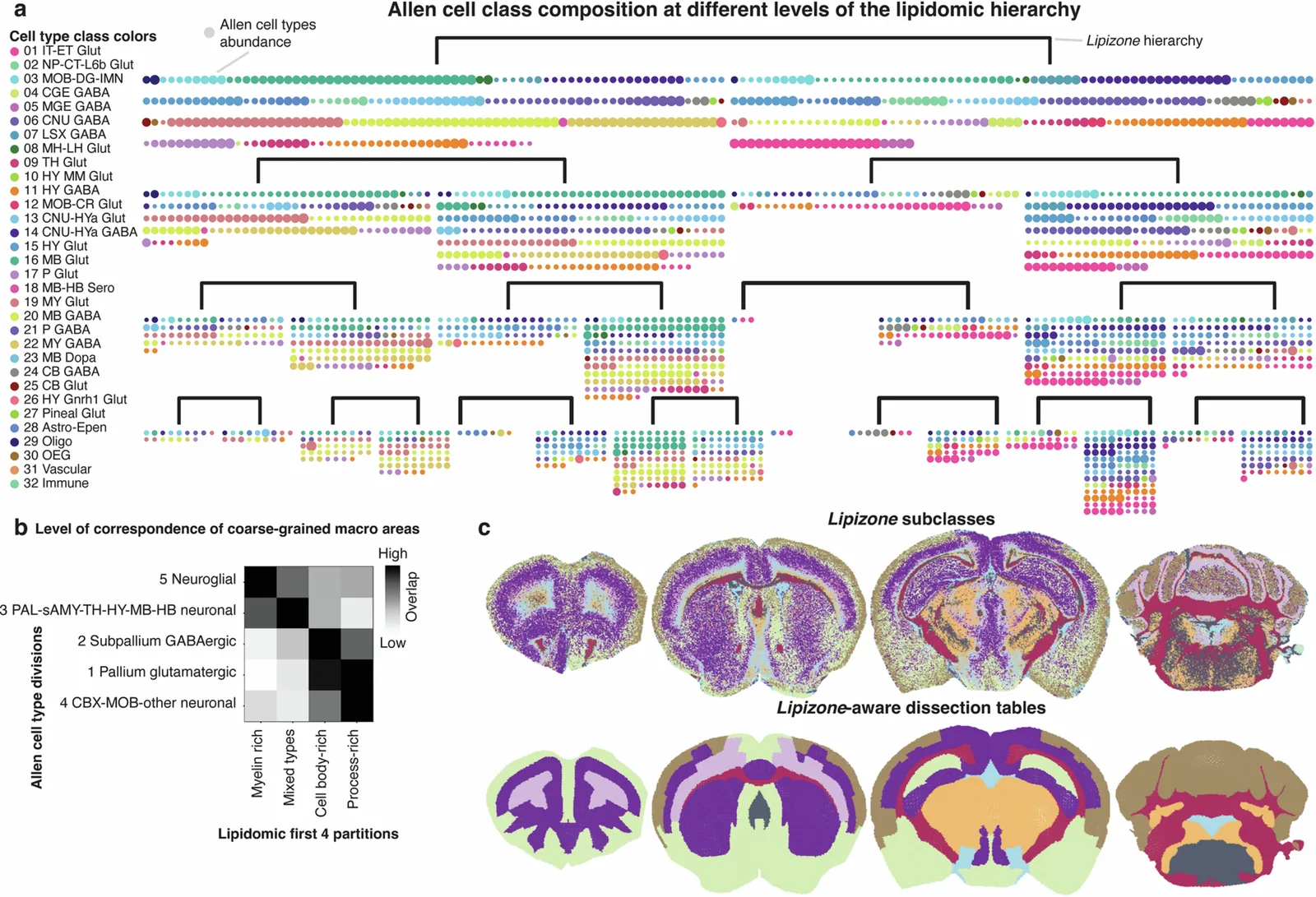
Comparison of lipidomic and transcriptomic brain hierarchies, and *lipizone*-informed dissection tables. (**a**) Dendrogram of the *lipizone* binary hierarchy (first 4 splits) with sorted dots for Allen supertype cell types, colored by the Allen cell type class (see legend). The dot size is proportional to their abundance, and dots are assigned to the side where they colocalize the most. (**b**) Heatmap of the spatial overlap between the five Allen cell type divisions (rows) and the first four lipidomic partitions (columns), showing the degree of correspondence between coarse-grained transcriptomic and lipidomic brain parcellations. (**c**) Representative brain sections colored by *lipizone* subclass (top) and the corresponding *lipizone*-aware dissection scheme (bottom), proposed as a reference for region selection in future targeted lipidomic studies.

The transcriptional hierarchy of cell types first separates neurons from glia, then developmental and neurotransmitter-driven divisions^25^. The lipidomic hierarchy does not recapitulate these axes (Extended Data Fig. 5a,b) and, instead, cuts across neuromeric origin. We interpret both the divergence of the hierarchies and their terminal-level matching as consistent with a single picture: the lipidome does not follow the same organizing principles as gene expression, yet distinct lipidomic profiles are modulated at the level of individual cell types—the finest entity with unique gene-regulation programmes.

The mismatch between traditional parcellations and the lipidomic architecture implies that analyses dissecting the brain regions spanning several lipizone classes risk being confounded. Therefore, to empower future experiments, we provide tables (with ABA coordinates) to guide lipizone-informed anatomical dissections (Extended Data Fig. 5c and Supplementary Table 9).

A total of 66 cell-type territories were matched by more than one lipizone. Given their spatial coherence, these lipizones might represent enrichments of different subcellular regions, processes and extracellular matrix, myelination levels or ‘lipotypes’^26^. One example is the cerebellar granule cell layer (Fig. 3f). The inner sublayer (proximal to the arbor vitae), where myelinated mossy fibres form synaptic glomeruli, was enriched in myelin-related lipids, whereas the outer layer was relatively enriched in phospholipids (Supplementary Fig. 8e). The lipizone hierarchy grouped the inner granule cells layer with regions rich in cell bodies, including cortical layer 6. In contrast, the outer granule cells layer clustered with axon-rich regions, including striatal, hypothalamic and thalamic areas, suggesting that the double-layer might reflect compositional differences between neuronal cell bodies and axonal projections. Lipizones that did not overlap cell-type territories localized largely to the hindbrain, striatum and midbrain (myelin-rich regions), and hypothalamus—33% of which is covered by only two lipizones.

**Extended Data Fig. 6:**
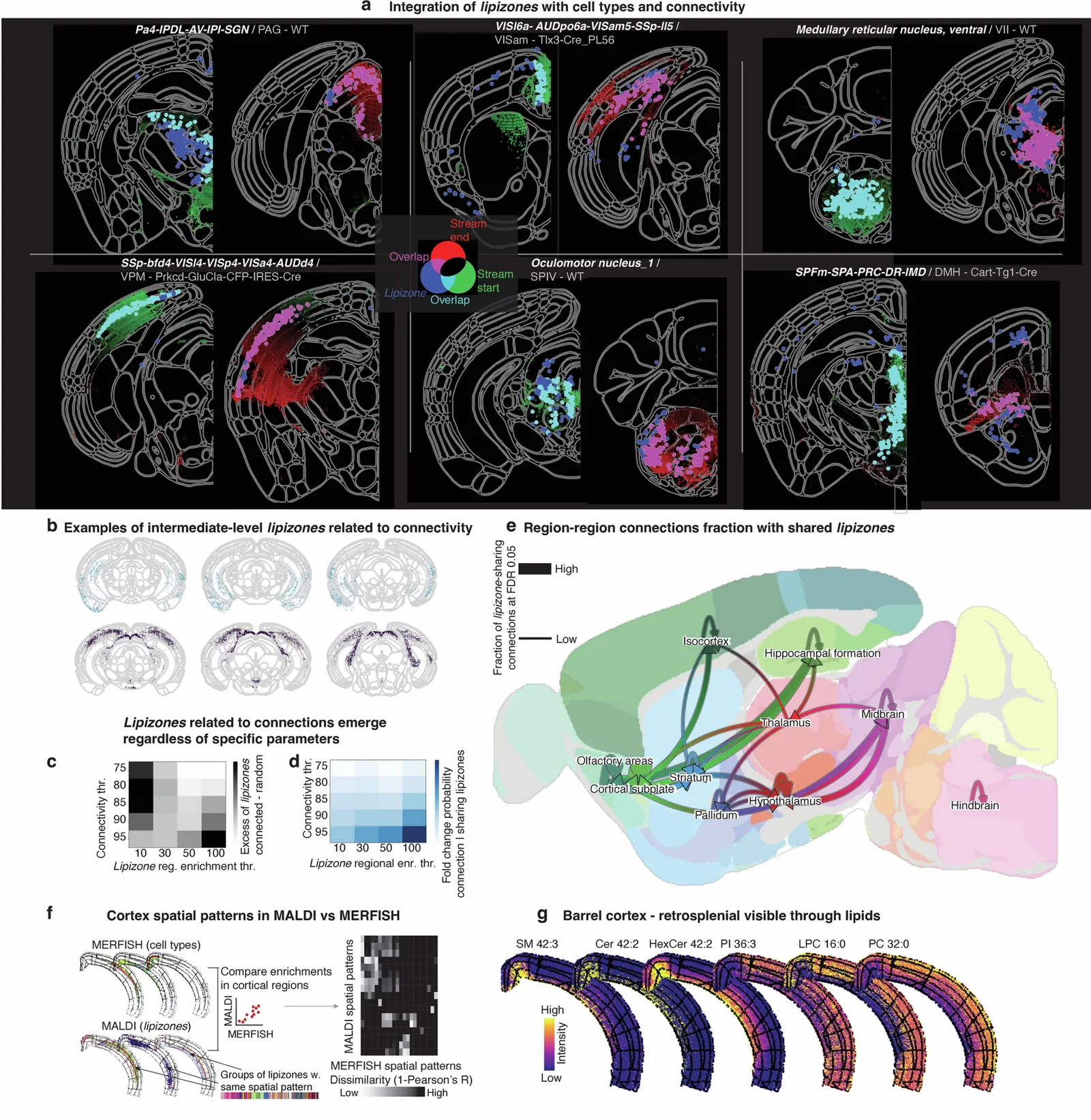
Integrative analysis of cell types, *lipizones*, and connectivity. (**a**) 2D visualizations of six further representative *lipizones* that likely include cell bodies (cyan) and their distal terminals (magenta). The associated connectomic streams are represented in background, colored from green to red, passing through black. The pixels of the *lipizones* that do not overlap with the connectomic streams are colored in blue. The gray text in the titles, after the white *lipizone* names, contains the connectome experiment injection site and mouse line. (**b**) Spatial plots of representative intermediate-level *lipizones* whose territory spans pairs of regions that are canonically connected. (**c**) Heatmap showing the excess of shared *lipizones* between connected versus null-model region pairs (*see* Methods), across varying thresholds for calling a connectomic connection (y-axis) and a *lipizone* regional enrichment (x-axis). (**d**) Heatmap showing the fold change in connection frequency for region pairs that share *lipizones* versus null-model region pairs, varying the threshold for calling a connection from the Allen mesoscale connectome (y-axis) and the threshold for calling a *lipizone* regional enrichment in the Lipid Brain Atlas (x-axis). (**e**) Spatial schematic of a sagittal mouse brain section, showing the fraction of *lipizone*-sharing connections at FDR 0.05 (curve thickness) stratified by brain division pairs. The coloring uses a gradient from the source region color to the target region color. (**f**) Left, schematic of the experiment comparing the unique spatial patterns delineated by MERFISH-derived cell types and MALDI-derived *lipizones*. We assessed the enrichment of each cell type and *lipizone* across cortical Allen regions, clustered cell types and *lipizones* based on these regional enrichments to detect unique spatial patterns, and compared these clusters between MERFISH and MALDI. Right, corresponding heatmap quantifying the pairwise dissimilarity of MERFISH cell type spatial patterns and MALDI *lipizone* spatial patterns in the cortex, evaluated as 1 - Pearson’s R calculated on the regional enrichments. (**g**) Spatial plots restricted to half section cortex for representative lipids highlighting the barrel and retrosplenial common lipidome captured by *lipizones*.

Lipizones that recapitulated cell-type territories often extended to physically distant regions, suggesting that individual lipizones might capture cell bodies and their distal axon terminals. We therefore sub-clustered lipizones by spatial coordinates and used Allen cell-body locations to classify subclusters as cell-body- or terminal-related (Supplementary Methods). To support a soma- or terminal-related role, we examined Allen connectomic streams^3^ overlapping both subclusters, testing whether they localized at opposite extremes of at least one stream. On the basis of these data, for 106 lipizones, we found two subclusters separated in space, directionally connected, with one subcluster co-localizing with the cell bodies (Fig. 3g and Extended Data Fig. 6a). Unlike cell-body-based spatial transcriptomics, lipidomic profiles are therefore able to capture long-range projections within individual cell types.

Notably, regions with known functional connections often clustered together in our data-driven hierarchy despite their spatial separation (Extended Data Fig. 6b). For example, ectorhinal, temporal association and motor cortices, all forming predominantly corticocortical connections, shared similar lipizones^3^. Similarly, the hippocampus clustered with its known input and output regions, including lateral septal nucleus, cortical amygdala, entorhinal cortex and cortical motor areas^27^. Anterior cingulate and hypothalamic nuclei^28^ have similar lipidomes, as well as thalamic nuclei and cortical layer 6, which form established corticothalamic circuits^29^ (Fig. 3h). Nonetheless, some connections, such as those between the thalamus and the thalamic recipient cortical layer 4, are not exposed by our lipidomic hierarchy. We also found spatial correspondence between some specific lipizone distributions and axon streams, such as in the striatum (Fig. 3i).

Compelled by these observations, we compared the mesoscale brain connectivity^3^ with lipizones. We found that connected brain regions shared one or more lipizones 2.28× more frequently than non-connected pairs (33.8% versus 14.8%, one-sided structured permutation test accounting for brain region distances; *P* < 0.001) (Fig. 3j and Extended Data Fig. 6c,d). These findings suggest that lipizone organization reflects aspects of brain connectivity architecture (Extended Data Fig. 6e and Supplementary Fig. 11a).

Lipizones were localized as anatomically as cell types^4^. For each lipizone and cell type, we counted the unique anatomical regions needed to reach 75% coverage. Distributions did not differ significantly between the two in any brain division (two-sided Mann–Whitney *U*; BH-FDR at 0.05; Fig. 3k, Extended Data Fig. 3g–i and Supplementary Methods). A non-stratified analysis pooling divisions, which captures processes spanning several regions, showed lipizones predictably covering more regions than cell types (8.3 versus 4.4 to reach 75%), consistent with their tracking of long-range connectivity.

In the cortex, we identified spatial distributions of lipizones that recapitulated cortical layering while revealing patterns not observed in the Allen cell-type atlas^4^ (Fig. 3l and Extended Data Fig. 6f). Notably, we discovered 14 layer 5 barrel field lipizones that were not detected by gene expression. In layer 5, these lipizones were also enriched in the retrosplenial cortex—a region that was proposed to connect with the barrel cortex^30^. The barrel field and the retrosplenial cortex were jointly enriched in specific lipids, such as Cer 42:2;O2 and SM 42:3;O2 (Extended Data Fig. 6g). We also identified lipizones restricted to the barrel field and somatosensory cortex in layer 4, whereas others spanned physically distant regions, such as the lipizones spanning the somatosensory and visual cortices. These findings suggest that lipid distributions might capture function-correlated cortical specializations beyond the cell-type composition.

**Extended Data Fig. 7:**
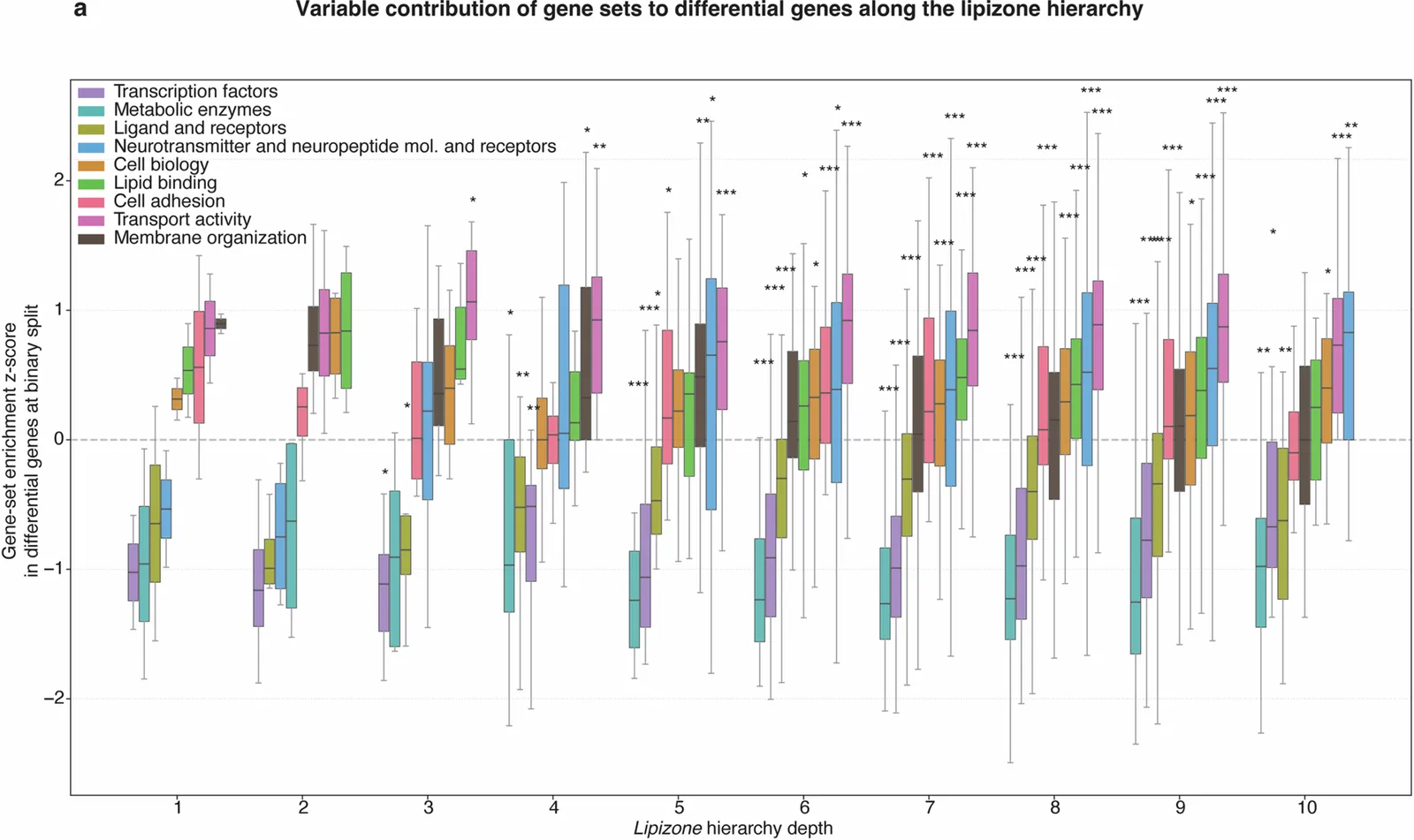
Gene set enrichment among differentially expressed genes at each level of the *lipizone* hierarchy. (**a**) Box plots showing the enrichment z-score of nine gene sets among differentially expressed genes at each binary split of the *lipizone* hierarchy (x-axis). At each hierarchy depth, boxes are sorted by ascending median. Asterisks denote significant deviation of the median z-score from zero (two-sided Wilcoxon signed-rank test, Benjamini–Hochberg-corrected): *q < 0.05, **q < 0.01, ***q < 0.001.

We next asked which biological processes distinguish lipizones along their hierarchy. Using the imputed Yao atlas^4^, we identified differentially expressed genes at each binary split (two-sided Welch’s *t*-test; Extended Data Fig. 7a) and computed their percent gene set coverage (Supplementary Methods). Cell adhesion and membrane organization-related genes led the second split of the hierarchy; genes with transporter activity led from the third split, followed by genes related to lipid binding and membrane organization. These gene sets stayed dominant, joined at deeper splits by neurotransmitter and neuropeptide genes, which eventually dominated the differentially expressed genes. Finally, transcription factors had low enrichment across all splits, suggesting that lipid configurations may relate to cellular processes beyond core-cell-identity programmes.

In conclusion, lipizones capture biomolecular variability tracking cell-type composition, cytoarchitecture, cellular processes and circuitry (Fig. 1k).

## Diversity of myelinated regions

**Fig. 4:**
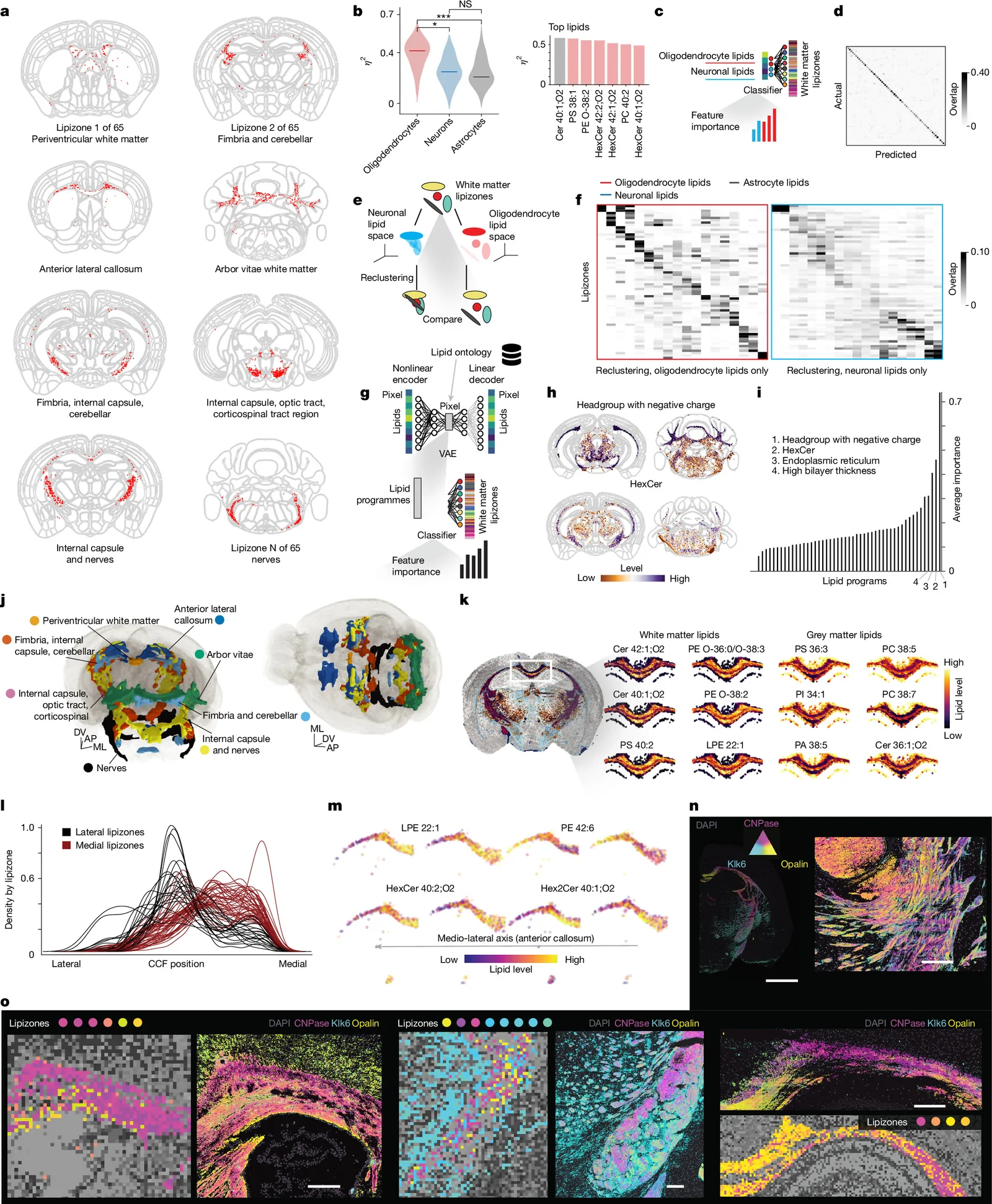
White matter lipidomic heterogeneity. **a**, Regionalized heterogeneity of fibre tract lipizones, showing spatial plots of eight white matter lipizones. **b**, Left, violin plot of lipid-wise *η*^2^ (SS_between/SS_total) using the 65 terminal white matter lipizones as groups. Lipids restricted to oligodendrocytes, neurons and astrocytes^9^; *n* = 60 lipids (16, 8 and 36, respectively). *P < 0.05; ***P < 0.001; NS, not significant; two-sided permutation test on median *η*^2^ difference (10,000 permutations; unadjusted). Right, bar plot of top *η*^2^ lipids, coloured by category. **c**, Schematic of the lipizone classification task extracting the relative importances of neuronal and oligodendrocyte lipids for white matter heterogeneity. **d**, Normalized confusion matrix (true versus predicted) for the 65-class lipizone classification task on held-out data. **e**, Schematic of pixel reclustering using only neuronal or oligodendrocyte lipids. **f**, Normalized confusion matrices comparing the 65 lipizones with oligodendrocyte-specific (left) and neuron-specific (right) lipid clusters. **g**, Top, schematic of lipiMap—a biochemically constrained variational autoencoder (VAE) learning latent biological programmes. Bottom, classification task extracting lipid programme feature importances for white matter heterogeneity. **h**, Spatial plots of two lipid programmes with regional heterogeneity within white matter. **i**, Bar plot of feature importances of lipid programmes for the white matter classification task, highlighting top programmes. **j**, Three-dimensional renderings of eight white-matter-related lipizones. **k**, Lipidomics of neural projections in white matter. Spatial plot of lipizones forming a bundle through the fibre tracts in the anterior callosum; zoom-ins coloured by differential lipids between bundle and surroundings. **l**, Kernel density estimates of white-matter-related lipizone abundances along the medio-lateral axis, one curve per lipizone; colours highlight medial versus lateral. **m**, Zoom-in spatial plots of lipids with a medio-lateral gradient in the anterior callosum (one hemisphere). **n**, Immunostainings for Opalin, Klk6 and CNPase—three myelin structure-related proteins.Pixel colour reflects position in the simplex (three pure colours as vertices); experiment replicated twice. **o**, Selected lipizones compared with immunostained white matter microstructure. For clarity, one lipizone was assigned manually per cyan, magenta or yellow channel; others using the simplex. Scale bars, 1,735 μm (**n**) and 200 μm (**n** (inset) and **o**).

We hypothesized that lipizones could reveal unrecognized heterogeneity in regions where specialized plasma membranes dominate over cell bodies, such as highly myelinated areas. One branch of the hierarchy contained 65 lipizones localized to fibre tracts, arbor vitae and the hindbrain, and another branch contained 70 myelin-rich lipizones. These regions were enriched for HexCer and an oligodendrocyte membrane-lipid score^9^ (Supplementary Fig. 12a–l; Methods). Individual lipizones were restricted to structures such as the periventricular white matter, fimbria, arbor vitae, internal capsule, optic tract, corticospinal tract and antero-lateral callosum (Fig. 4a and Supplementary Fig. 12b,c). This structured diversity is notable given that myelin has been described as homogeneous, with fewer than ten oligodendrocyte subtypes recognized transcriptomically^4,31^.

We therefore asked whether this heterogeneity depends on myelin, astrocytes or axon composition. For each lipid, we computed *η*^2^—the fraction of its white matter variance explained by lipizone membership—and compared the *η*^2^ distributions across oligodendrocyte, neuron and astrocyte-restricted lipid sets^9^. Oligodendrocyte-specific lipids had higher *η*^2^ than both neuronal lipids (*P* = 0.004) and astrocyte lipids (*P* < 0.001; two-sided permutation test on median *η*^2^ differences, 10,000 permutations; Fig. 4b and Supplementary Fig. 12d). This result held across alternative clustering regimes. We then trained a classifier on oligodendrocyte- and neuron-enriched lipids^9^ and used Shapley additive explanations^32^ to extract the feature importances for each lipizone (Fig. 4c,d and Supplementary Methods). Oligodendrocyte-restricted lipids were more predictive than neuronal lipids (gene set enrichment analysis, *P* = 0.048 for oligodendrocyte lipids, non-significant for neuronal lipids; Supplementary Fig. 12e). To confirm, we restricted features to oligodendrocyte or neuronal lipids, reduced dimensionality to ten principal components and reclustered (Fig. 4e). The oligodendrocyte lipids space better recovered the fine-grained lipizones (normalized mutual information = 0.24 versus 0.12; adjusted Rand index = 0.057 versus 0.023) (Fig. 4f).

**Extended Data Fig. 8:**
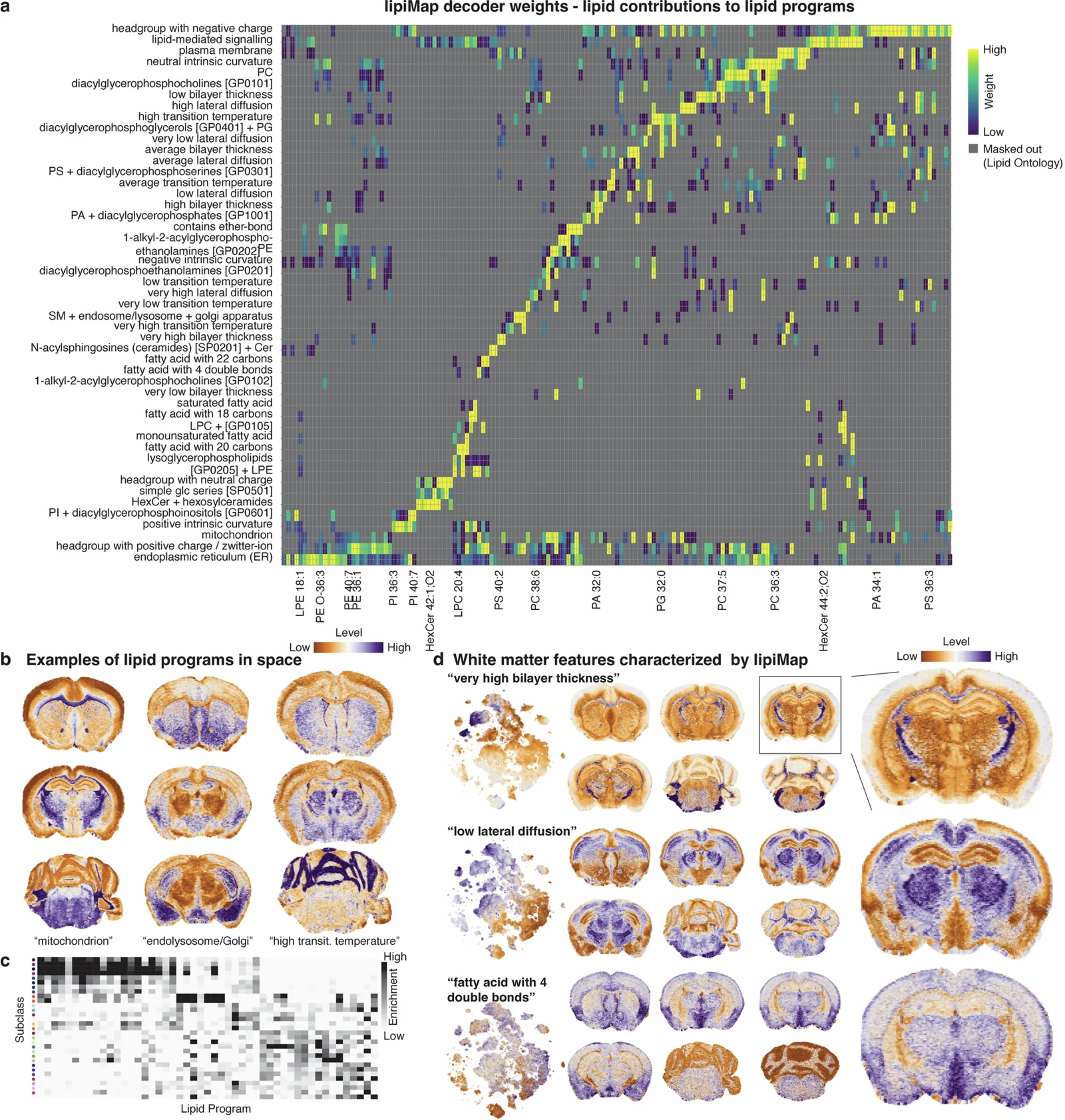
A biochemically-constrained variational autoencoder to disentangle lipid programs. (**a**) Heatmap of the decoder weights of the variational autoencoder, for each lipid program and lipid pair, gray where a lipid does not belong to a program; color intensity otherwise represents the weight of a lipid program to reconstruct the abundance of a given lipid. Columns are individual lipids; only few lipids are labelled for readability. (**b**) Spatial plots for example lipid programs, representative sections. (**c**) Heatmap of lipid program enrichments by *lipizone* subclass. (**d**) tSNEs and spatial plots of representative sections, with zoom-ins, colored by lipid programs that characterize the white matter and its heterogeneity.

To reveal the biological processes underpinning the observed heterogeneity, we designed lipiMap, a biochemically constrained variational autoencoder (Fig. 4g and Supplementary Methods). We trained the model on brain 1, enriching each pixel with interpretable lipid programmes^33^ (Fig. 4h and Extended Data Fig. 8a–c). We inferred thicker membrane properties in the white matter, accompanied by a low lateral diffusion, aligning with the notion that white matter membranes are stable and compact (Extended Data Fig. 8d). We found that known myelin-related characteristics had the highest feature importance to distinguish the white matter-rich lipizones, including the programmes ‘headgroup with negative charge’, ‘hexosylceramides’, and ‘high bilayer thickness’^34^ (Fig. 4i). Collectively, these observations suggest that the lipidome of oligodendrocytes is the main driver of the regional heterogeneity of white matter-rich lipizones (Fig. 4j).

We next asked whether axonal lipid composition contributes to residual heterogeneity in fibre tract lipizones outside the main myelin-rich branch. We focused on a specific part of the white matter that carries interhemispheric axons within the corpus callosum^35^. Lipizones in this area identified a bundle crossing between the two hemispheres (Fig. 4k). Differential analysis showed that this bundle was enriched in the neuronal marker Cer 36:1;O2 (ref. ^9^) and phospholipids, whereas the surrounding white matter was enriched in myelin lipids, including other Cers, PSs and ether lipids (Fig. 4k and Supplementary Fig. 12f). This pattern with lightly myelinated axons in the middle^36^ suggests that variable myelination is a source of heterogeneity.

Given the stability of myelin and its retention of ontogenic marks^37^, we hypothesized that white matter lipizone diversity might reflect developmental origin. Specifically, we reasoned that as myelination progresses postnatally in waves reaching the medial callosum later than the lateral, this temporal sequence might leave enduring lipid signatures in the adult^38^. Consistent with this prediction, we found that anterior fibre tract lipizones grouped into lateral and medial pools (Fig. 4l and Supplementary Fig. 12g) (average CCF 3.72 versus 4.33, two-sided *t*-test; *P* < 0.0001). Specific lipids, including Hex2Cer 40:1;O2 and HexCer 40:2;O2, LPE 22:1 and PE 42:6, correlated with the medio-lateral axis in the anterior callosum (Fig. 4m), suggesting that myelin lipid signatures retain chronotopic developmental features.

We hypothesized that this regional heterogeneity of myelin lipidomes might correspond to local variations of myelin structure, such as compactedness or density. We therefore conducted immunofluorescence experiments, staining for Opalin, Klk6 and CNPase. White matter territories showed zonation based on these three structural markers (Fig. 4n), with the resulting pattern resembling lipizones (Fig. 4o).

## The ventricles are zoned metabolically

**Extended Data Fig. 9:**
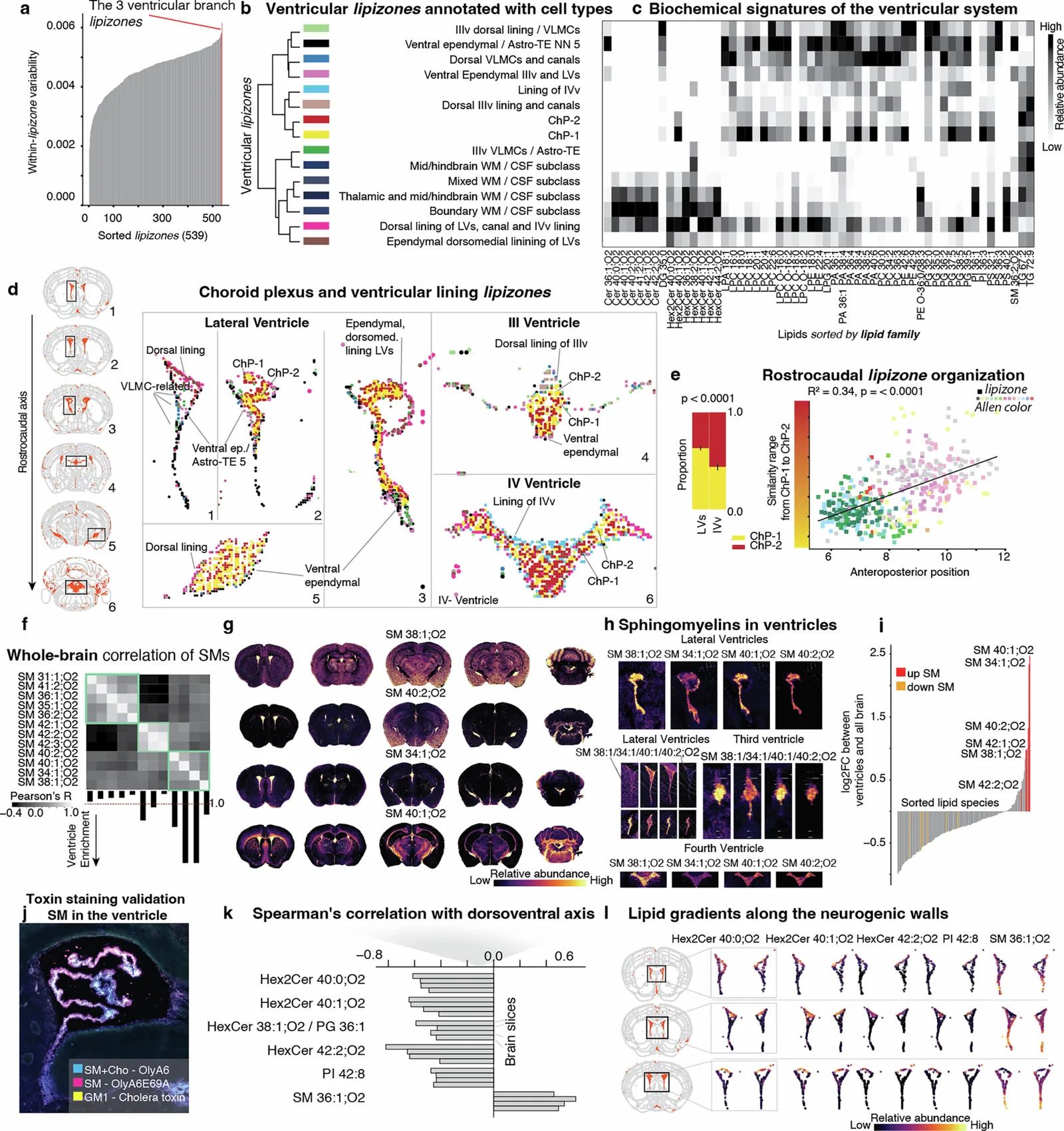
Metabolic zonation in the ventricular system. (**a**) Lineplot of within-*lipizone* variability for all the 539 *lipizones*. Lines are sorted in ascending order. Ventricular *lipizones* are indicated in red. (**b**) Annotated dendrogram representing the hierarchy of fine ventricular *lipizones*, with (**c**) matched heatmap of lipid relative abundances. Lipids are ordered by lipid class. (**d**) Close up on ventricles, and their *lipizones*. *Lipizones* are colored as in (**b**). (**e**) Left: stacked barplots of the proportions of the two core choroid plexus *lipizones*, in the lateral (LVs) and fourth (IVv) ventricles; two-sided Pearson correlation (p < 0.0001), using *lipizones* as statistical units. Right, scatter plot of position along the anteroposterior axis (x axis) against the similarity with the two choroid plexus *lipizones* (y axis); each point is a *lipizone* and is colored by its Allen Brain color. (**f**) Heatmap of the brain-wide correlation of sphingomyelins, highlighting three blocks (gray, white matter, and ventricular). The falling barplot at the bottom highlights the ventricular enrichment of each sphingomyelin. The red dashed line represents no enrichment (= 1.0). (**g**) Spatial plot of the four ventricular sphingomyelin markers, across the entire brain, for 5 representative sections. (**h**) Spatial zoom-in on the ventricles colored by the four ventricular sphingomyelin markers. (**i**) Barplot of the log2 fold changes of lipids in the ventricular systems compared to the rest of the brain. Sphingomyelins are highlighted in red (increased in ventricles) and orange (decreased). (**j**) Toxin staining validation of sphingomyelin in the choroid plexus. The toxins and the lipids they mark are indicated and color-coded. (**k**) Barplots of the Spearman’s correlations between lipid abundances and the dorsoventral axis in the lateral ventricle-lining *lipizone* (one bar per brain section; bars correspond to all sections where the lateral-ventricle-lining *lipizone* was detected), with (**l**) Spatial view of the lateral ventricle linings colored by individual lipids having gradients along the dorsoventral axis.

As the ventricles harboured high residual variability unexplained by terminal lipizones (Extended Data Fig. 9a), we reclustered ventricular and periventricular lipizones into 15 subzones (Extended Data Fig. 9b–d). Some recapitulated the organization of vascular leptomeningeal cells, ependymal cells and telencephalic astrocytes.

We identified two distinct clusters in the choroid plexus (ChP), which we termed ChP1 and ChP2 (Extended Data Fig. 9d and Supplementary Fig. 12h). Whereas ChP1 was enriched in phospholipids and ether lipids, ChP2 contained relatively more HexCers (Supplementary Fig. 12i). The differential lipid profiles suggested functional specialization: the sphingolipid enrichment of ChP2 may indicate proinflammatory signalling capacity^39^, whereas the phospholipid abundance of ChP1 suggests secretory functions. Because the adult ChP retains ventricle-specific gene expression mirroring embryonic origins^40^, we asked whether ChP1, enriched in the lateral ventricles (Extended Data Fig. 9e), had a more anterior lipid signature than ChP2, enriched in the fourth ventricle. Correlating each cluster’s similarity to all lipizones with their average antero-posterior position, we found more posterior lipizones were significantly more similar to ChP2 (two-sided Pearson correlation, *P* < 0.0001; Extended Data Fig. 9e and Supplementary Fig. 12j). This suggests that developmental positional identity is retained in the ChP lipidome.

Although sphingomyelins are typically classified as white matter lipids^10^, we found them enriched in the ventricles. A whole-brain SM correlation matrix revealed three distinct clusters (Extended Data Fig. 9f): one broader in the grey matter, with SM 36:2;O2 and SM 36:1;O2; one restricted to the white matter (42-long SMs); and another strictly ventricular. We propose SM 38:1;O2, SM 34:1;O2, SM 40:1;O2 and SM 40:2;O2 as ventricular markers, as they highlight the ventricular system in whole-brain views (Extended Data Fig. 9g–i and Supplementary Fig. 12k). SM 40:1;O2 and SM 34:1;O2 are also over-represented in blood lipidomes^41^, but our results held when omitting them. Staining with toxins that specifically bind to SMs (and GM1 as a negative control)^42–44^ validated SMs as ventricular markers (Extended Data Fig. 9j).

We identified six lipids, including the neuronal SM 36:1;O2, that displayed pronounced dorso-ventral gradients along the walls of the lateral ventricles (two-sided Spearman correlation, BH-FDR corrected; |R| > 0.4, adjusted *P* < 0.0001; Extended Data Fig. 9k,l and Supplementary Fig. 12l). The walls of the lateral ventricles host the adult brain’s largest neural stem cell pool^45^, which produces distinct neuron progeny depending on their dorso-ventral position. These lipid gradients may therefore track the patterning of the neurogenic niche. These findings reveal lipid-based zonation within the ventricular system.

## Pregnancy remodels brain lipizones

We used MALDI–MSI to characterize the brain lipidome of three male and three female mice with approximately matched coronal sections (29 acquisitions in total included in the atlas; Supplementary Fig. 13a–l). The effect of sex on the lipidome was generally limited (Supplementary Fig. 13b,d and Supplementary Methods). Of the 222 supertypes, only 29 displayed a sex-related log_2_ fold change of at least 0.2 for more than 5 of 172 membrane lipids (Supplementary Fig. 13b,c).

**Fig. 5:**
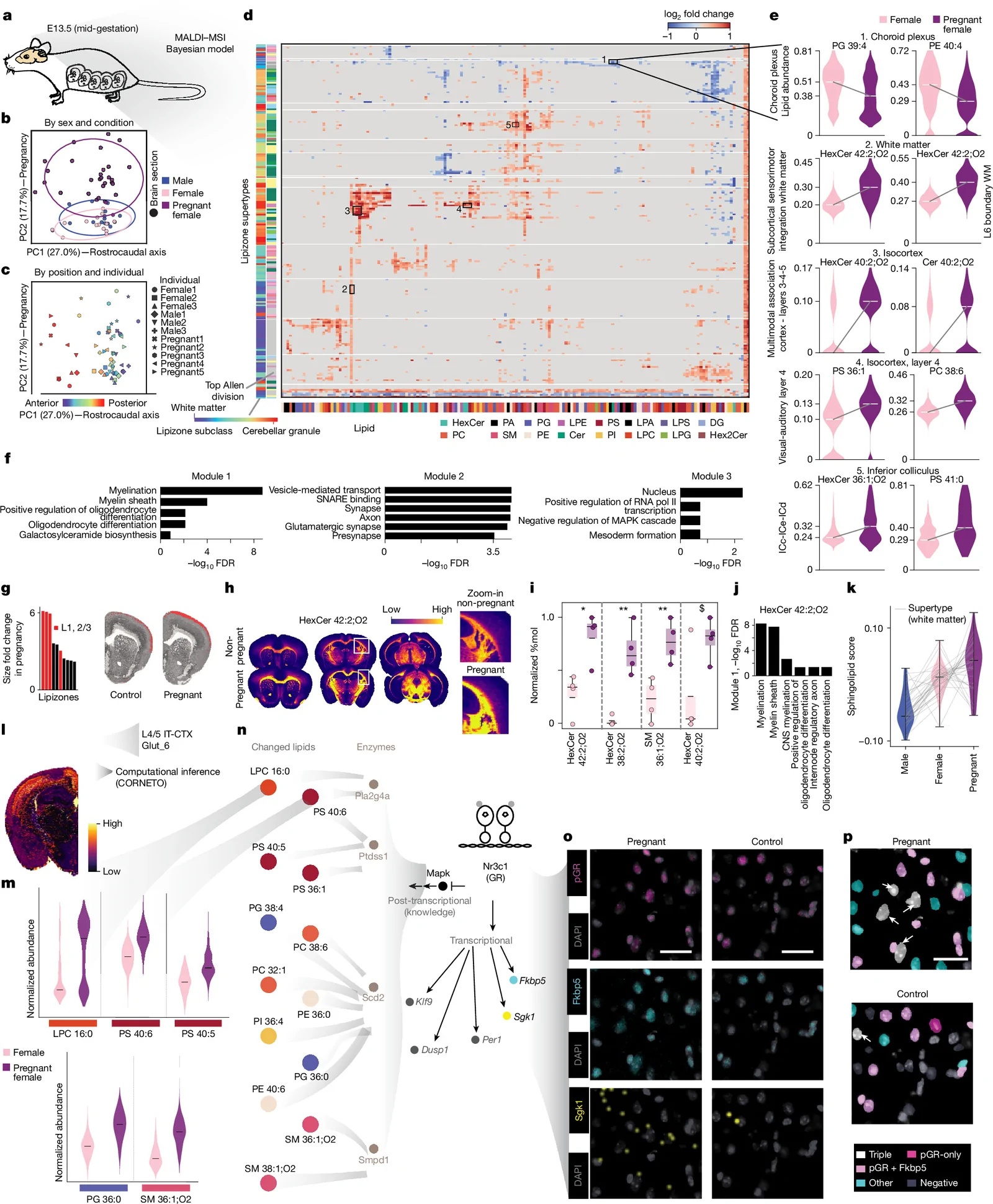
Lipidomic reorganization in the brain of pregnant mice. **a**, Schematic of pregnant mouse. **b**, PCA scatterplot of section-wise brain lipidomes for male and female mice and pregnant female mice; ellipses, data ranges; axes label, correlated covariate. *n* = 5 pregnant female mice (6 sections each); *n* = 3 male and *n* = 3 female mice (29 sections total). **c**, As in **b**, coloured by rostrocaudal position; marker shapes indicate individual mice. **d**, Lipid log_2_ fold changes in pregnancy by lipizone supertype; row colours, lipizone subclass and Allen division; column colours, lipid class. Grey color indicates non-significant. **e**, Violin plots of selected lipids for six lipizones; lines, medians. **f**, GO importance analysis, showing top GO terms for three gene-expression modules predictive of lipid changes. MAPK, mitogen-activated protein kinase. **g**, Barplot and spatial plot of size fold change for most altered lipizones; two-sided *t*-test across individual mice (*P* = 0.002 for the most expanded). **h**, Myelin marker HexCer 42:2;O2 in non-pregnant (control) mice versus pregnant mice. **i**, Box plots of (total-lipid-normalized) %mol LC–MS abundances for selected sphingolipids, control versus pregnant (dots, samples; *n* = 4 versus 4); Welch’s *t*-test, two-sided, unadjusted (HexCer 42:2;O2 *P* = 0.011; HexCer 38:2;O2 *P* = 0.0095; SM 36:1;O2 *P* = 0.0075; HexCer 40:2;O2 *P* = 0.075, $); median, box, 25–75 percentiles, 1.5 × IQR. **j**, Top GO terms for module predictive of HexCer 42:2;O2 change. **k**, Violin plots of sphingolipid score across core white matter supertypes in male, non-pregnant female and pregnant female mice; lines, matched supertypes; *n* = 3 brains per condition: median, minimum and maximum shown. **l**, Large change in layer 4 shown as a spatial plot; colour bar, number of lipids changed. **m**, Violin plots of selected lipids. **n**, CORNETO schematic, lipid changes to putative signalling (L4/5 IT-CTX Glut). In **l**–**n**, pixels pooled from *n* = 3 control and *n* = 3 pregnant mice. **o**, Immunostaining for phospho-glucocorticoid receptor (p-GR) and its targets in cortical layer 4 (VISal4 brain region); *n* = 2 animals per condition. DAPI, 4′,6-diamidino-2-phenylindole (grey background). **p**, Same micrographs as in **o**, showing overall response state; cells categorized by phospho-glucocorticoid receptor, Fkbp5 intensity and Sgk1 puncta density; arrows, triple positives; *n* = 2 animals per condition. Scale bars (**o**,**p**), 25 μm.

We next asked whether physiological adaptations can modulate the lipidome dynamically. Brain remodelling during pregnancy includes volumetric changes and circuit restructuring^46^ to support behavioural modifications and fetal metabolic demands^47^, which might involve lipid changes. We measured the brain lipidome of first-pregnancy female mice (mid-gestation (E13.5); Fig. 5a) by MALDI–MSI alongside non-pregnant female mice, and label-transferred 215 of 222 lipizone supertypes.

A first global variance analysis revealed that pregnancy is associated with more extensive changes in brain membrane lipids than sex (Fig. 5b,c). Using a Bayesian hierarchical model controlling for batch effects and inter-individual variation (Supplementary Fig. 13e–k and Supplementary Methods), we quantified pregnancy-induced changes for each lipizone supertype (log_2_ fold changes were between −2 and +2).

To assess whether lipizones offer a genuine advantage over conventional parcellations, we trained an analogous model using anatomical regions instead of lipizones. Despite nearly twice as many categories, the anatomical model yielded significantly worse fits (two-sided Wilcoxon test, *P* < 0.0001; Supplementary Fig. 13l).

**Extended Data Fig. 10:**
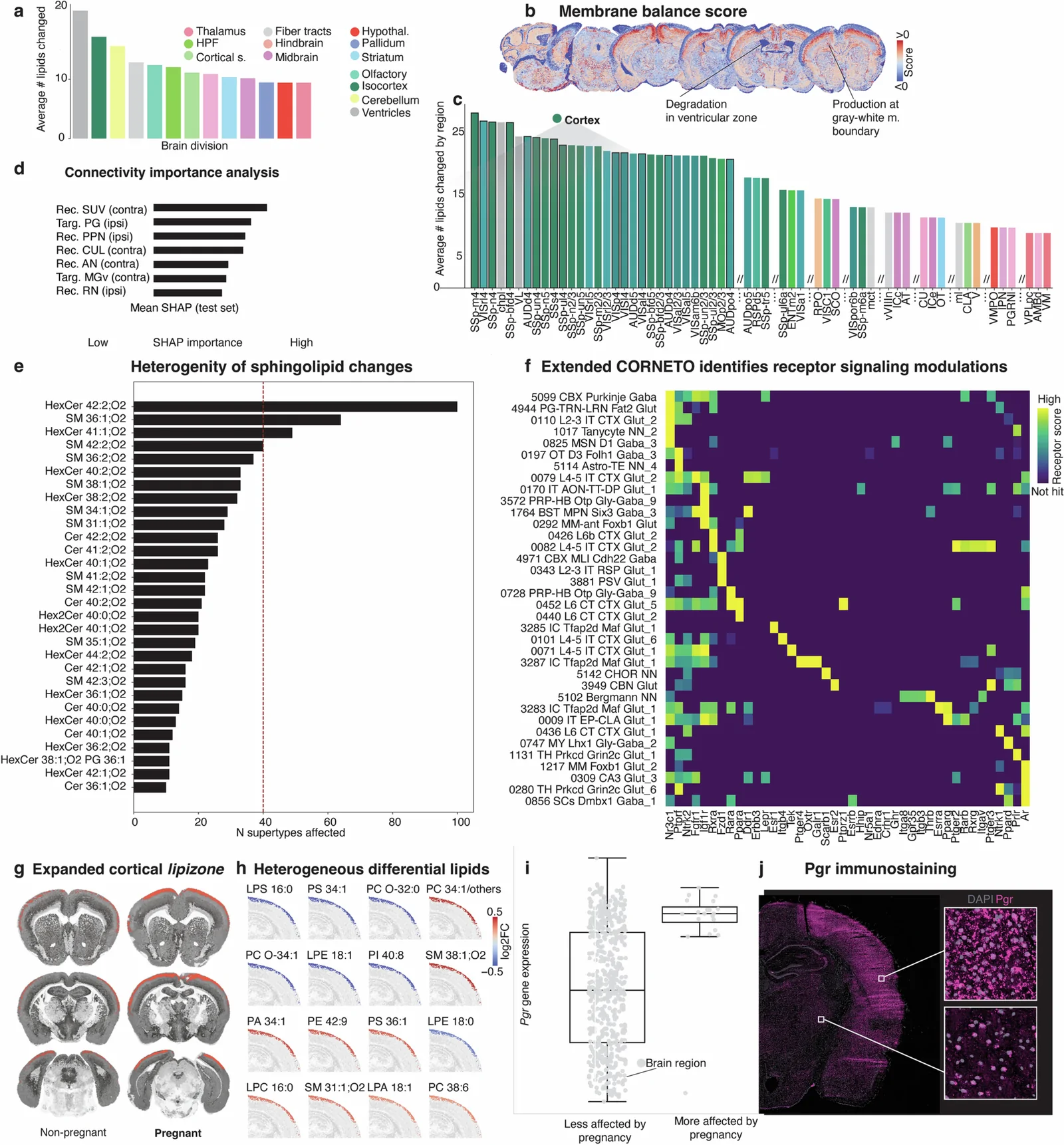
Spatial lipidomic changes in pregnancy and their relationship to signalling pathways and myelin. (**a**) Barplot of the average number of lipids changed in pregnancy, subdivided and colored by Allen Brain division. (**b**) Spatial plot of the membrane balance score for representative sections, which quantifies the supertype-resolved overall lipid variation in pregnancy, by summing the net shifts inferred via Bayesian modeling across all lipids, charting the relative prevalence of biosynthesis or turnover. (**c**) Bar plot of the average number of lipids changed by pregnancy, subdivided and colored by Allen Brain fine-grained region. The x axis is broken multiple times to show representative regions from highly to minimally changed; the cortex is highlighted. (**d**) Bar plot of mean absolute SHAP feature importance extracted from a model predicting the number of lipids affected in pregnancy from the regional connectome. (**e**) Barplot showing the number of *lipizone* supertypes in which a given sphingolipid (rows) is changed in pregnancy. The dark red dashed line divides sphingolipids between altered in <40 and > 40 supertypes. (**f**) Heatmap showing, for each *lipizone* supertype (rows), the receptor pathways (columns) predicted by the extended CORNETO analysis to be activated in pregnancy in the corresponding cell type territory. (**g**) Spatial plot of the *lipizone* whose proportion was increased in pregnancy. (**h**) Spatial plots of selected lipids with differential abundance in pregnancy in the outer cortex, coloured by log_2_ fold change. (**i**) Box plots with overlaid strip plot of *Pgr* expression across Allen Brain Atlas regions, measured by MERFISH spatial transcriptomics, comparing the 20 *lipizone* supertypes most affected by pregnancy against the rest of the brain. Two-sided Mann–Whitney U test, p = 7.9 × 10^−7^. Centre line, median; box, 25th-75th percentile; whiskers, 1.5 × IQR; individual data points are overlaid. (**j**) Immunostaining for PGR protein in a representative female brain section, with insets showing cortex and thalamus.

For the top 20 most altered supertypes, the average pregnancy-versus-control change was 120% of the mean variation between sister supertypes, with 16 lipids modulated per supertype. Overall, 139 of 215 supertypes were affected (>5% differential lipids; Fig. 5d). The ventricular system had the most differential lipids, followed by the isocortex, fibre tracts and olfactory areas; whereas pallidum, thalamus and hypothalamus had the fewest (Extended Data Fig. 10a–j). Within the cortex, the most altered supertypes localized to layer 4 (Extended Data Fig. 10c).

Next we scored the overall membrane-lipid change across regions (Extended Data Fig. 10b), revealing region-specific remodelling: negative scores (prevalent turnover or inhibited biosynthesis) in ventricles, hindbrain, white matter, cerebellum, outer cortex and hypothalamus, and positive scores (prevalent biosynthesis or inhibited turnover) at grey–white matter boundaries and in deep cortical layers. The ChP supertype had 36 decreased and only 2 increased lipids (Extended Data Fig. 10b).

Differential lipids were highly heterogeneous across supertypes (a median of 14 affected supertypes per lipid, log_2_ fold change > 0.2 or < −0.2 for greater than 98% posterior samples), further pointing to local rather than global adaptations (Fig. 5e).

To understand what makes certain populations susceptible to pregnancy remodelling, we trained a predictive model of lipid changes from gene expression (Allen Brain Atlas^4^) and examined the driving features (Supplementary Methods). The most predictive genes related to myelin structural constituents, presynapses, lipid metabolism, histone deacetylation, nucleus, SNARE binding and vesicles (Fig. 5f). These genes were not trivially the markers of the most affected regions; their convergence on membrane-related processes suggests that a cell’s lipidomic response to pregnancy is shaped by its baseline membrane biology.

We also asked whether the mesoscale connectome^3^ could contribute to explain the pattern of differential lipids, using the same predictive feature analysis approach. The most predictive connectivity streams converged on vestibulo-cerebellar, brainstem state control^48^; limbic–thalamic–retrosplenial and projections onto the ventral medial geniculate^49^ (Extended Data Fig. 10d). The last two are related to auditory salience circuits, relevant to maternal behaviour.

Beyond composition, some lipizones also changed size. Three supertypes in cortical layers 1 and 2/3 expanded during mid-gestation, the most reaching 6-fold higher representation (*P* = 0.002, two-sided *t*-test across individual mice; Fig. 5g and Extended Data Fig. 10g). This is consistent with the cortical hypertrophy previously reported during pregnancy^50^. Their lipidomic changes were nonetheless modest: 16 lipids modulated at absolute log_2_ fold change > 0.2 (Extended Data Fig. 10h). Other lipizones did not change size but were remodelled in composition. The inferior colliculus lipizone—a subcortical hub integrating ascending auditory input with descending cortical signals^51^—increased myelin-related lipids, whereas the LM-VISC1-PVp-MMm-MMp lipizone (including Papez circuit nuclei involved in emotional regulation^52^) and commissural branch of stria terminalis–substantia nigra (a myelinated fibre bundle carrying amygdala outputs) decreased them (Fig. 5d), suggesting that pregnancy fine-tunes myelin composition in a region-specific manner.

**Extended Data Fig. 11:**
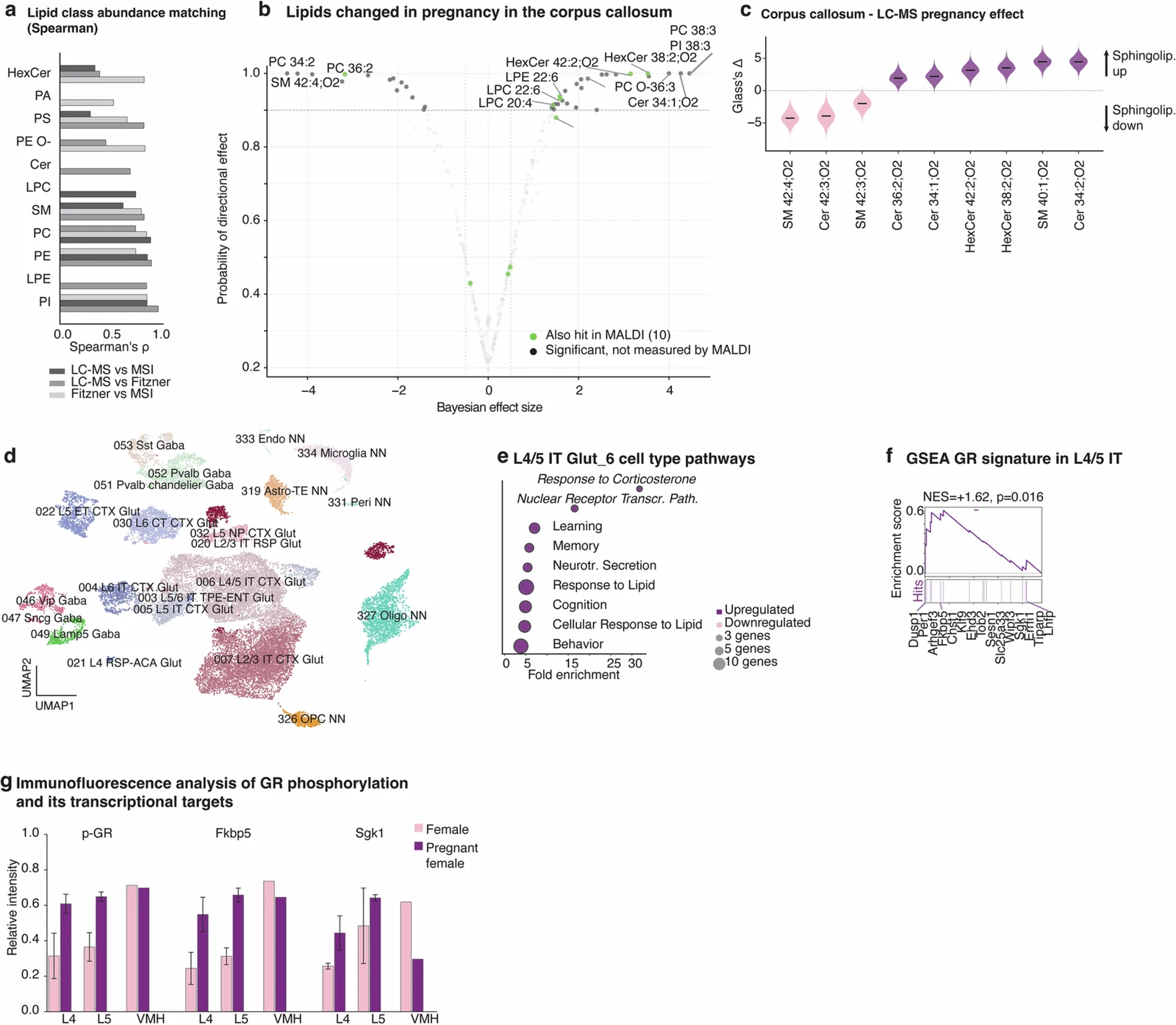
Validation of pregnancy-associated lipidomic changes by LC-MS, snRNA-seq and immunostaining. (**a**) Bar plots showing, for each lipid family, the Spearman correlation among individual lipids within a family across dataset pairs: microdissection-based bulk lipidomics, reference bulk lipidomics from Fitzner et al.^9^, and the spatial MSI dataset. (**b**) Volcano plot of bulk lipidomics data showing the effect size (x axis) and the significance (from the integrated Bayesian posterior); lipids with supporting evidence in callosal *lipizones* from the MALDI dataset are highlighted in green; grey dots represent lipids detected by LC-MS but not by MALDI. (**c**) Violin plots of the Bayesian posterior of the pregnancy effect (in units of standard deviations from non-pregnant controls), showing LC-MS changes for selected sphingolipids. (**d**) UMAP of the single-nucleus RNA-sequencing dataset (pregnancy and control samples), colored by Allen Brain Atlas cell types at the subclass level. (**e**) Over-representation analysis for the genes changed in the most affected cell type (L4/5 IT-CTX Glut), showing fold enrichment for selected significant terms. Dot size is proportional to the number of differentially expressed genes contributing to that term, whereas color distinguishes enrichment versus depletion in pregnancy. The analysis is based on snRNA-seq. (**f**) Gene set enrichment analysis (GSEA) enrichment plot for the glucocorticoid receptor gene signature, based on snRNA-seq. (**g**) Bar plot showing the relative signal intensity for p-GR and Fkbp5 and relative average puncta per cell for Sgk1, in pregnant and control brain sections. Each bar represents the per-condition average; the standard error of the mean across bregmas is shown. N = 2 biologically independent animals per condition (n = 2 bregmas per animal). L4 = cortical layer 4, L5 = cortical layer 5, VMH = ventromedial nucleus of the hypothalamus.

Despite the predominantly region-specific nature of the response, some lipids were modulated recurrently across many supertypes. The myelin marker HexCer 42:2;O2 (most probably galactosylceramide 42:2;O2)^9^ was increased in 100 supertypes at mid-gestation, especially across the white matter (average log_2_ fold change = 0.38; log_2_ fold change > 0.2 for greater than 98% posterior samples; Fig. 5h). Microdissection and bulk LC–MS of the corpus callosum confirmed increased HexCer 42:2;O2 and other sphingolipids (Fig. 5i, Extended Data Fig. 11a–c and Supplementary Table 1). Feature importance analysis attributed this increase to genes involved in myelination and oligodendrocyte differentiation (*Aspa*, *Nkx6-2*, *Myrf*, *Olig2*, *Il33*; Fig. 5j and Supplementary Methods). Sphingolipids changed more than phospholipids across most supertypes (135 of 215, one-sided binomial test; *P* < 0.001), and were generally increased (667 of 791 lipid-supertype pairs, one-sided binomial test; *P* < 0.0001) although this response was not uniform: 26 of 30 sphingolipids changed in fewer than 40 supertypes each (Extended Data Fig. 10e). Together, these observations point to widespread but regionally selective adaptive myelination in pregnancy, consistent with prolactin-mediated re-myelination during mouse gestation^53^. A sphingolipid signature also showed a graded increase across white matter supertypes from male to non-pregnant female to pregnant female mice (Fig. 5k).

Having established the landscape of lipidomic remodelling, we next focused on the cortex, where six supertypes showed more than 25 changed lipids. These spanned cortical layer 4, encompassing the layer 4–layer 5 intratelencephalic glutamatergic neuron territory (Fig. 5l). The most affected lipizone, ‘VISal4-VISrl4-AUDd4-VISp4-AUDpo4’ (cell-type territory 0100/0101 layer 4–layer 5 intratelencephalic cortical glutamatergic 6 (L4/5 IT-CTX Glut_6)), had 34 differential lipids, including four increased phosphatidylserines (PS 36:1, 40:6, 40:5, 34:1; Fig. 5m), an anionic class that regulates vesicle docking, fusion and presynaptic organization^54^. These changes in the thalamorecipient layer of visual and auditory cortex may tune sensory–perceptual circuits for infant cue detection^55^.

To identify extrinsic cues driving these regional changes, we examined the baseline receptor landscape: the 20 most affected supertypes had higher progesterone receptor (*Pgr*) expression than the rest of the brain (two-sided Mann–Whitney; *P* < 0.0001; Extended Data Fig. 10i), revealing high cortical *Pgr* expression that we confirmed by an independent spatial transcriptomics dataset^1^ and immunostaining (Extended Data Fig. 10j).

To move beyond this first-order analysis, we adapted CORNETO^56^ and the Rhea database^57^ to identify receptor–signalling–enzyme–lipid paths parsimoniously explaining joint lipid changes within each lipizone (Fig. 5n and Supplementary Methods). Across lipizones, 42 receptors were prioritized, including *Esr1*, *Esr2*, *Prlr*, *Lepr*, *Oxtr*, *Ghr* and *Crhr1* (Extended Data Fig. 10f). The receptors accounting for the most lipizone changes were *Nr3c1*, *Ptprf*, *Ntrk2*, *Rxra*, *Igf1r*, *Ar* and *Fgfr1*. For the most affected lipizone, 0101 L4/5 IT-CTX Glut_6, the changes were best explained by transduction from *Nr3c1*—the glucocorticoid receptor—with the most parsimonious model predicting a blockade of *Mapk* signalling, converging on lipid biosynthesis through *Smpd1* (affecting SMs), *Ptdss1* (modulating PSs) and *Scd2* (governing the PC/PE pool and PGs).

We corroborated this prediction experimentally. Using single-nucleus RNA sequencing (snRNA-seq) (Extended Data Fig. 11d), we found that the layer 4–layer 5 intratelencephalic glutamatergic population had consistent transcriptional changes: genes marked by the ontology terms ‘response to corticosterone’ and ‘nuclear receptor transcription pathway’ were significantly over-represented (Extended Data Fig. 11e); furthermore, a curated signature of 15 glucocorticoid response genes was significantly enriched^58^ (Extended Data Fig. 11f and Supplementary Methods). *Per1*, a sensitive glucocorticoid receptor target^59^ and circadian regulator, was upregulated. The glucocorticoid receptor itself was upregulated. Two canonical glucocorticoid receptor targets (Fkbp5 and Sgk1) and glucocorticoid receptor phosphorylation state (Ser211) seemed increased, as did the fraction of triple-positive cells, by immunostaining (*n* = 2 pregnant, *n* = 2 control animals) (Fig. 5o,p and Extended Data Fig. 11g). These observations suggest a connection between a lipidomic signature and a defined hormonal signalling axis.

## Discussion

Using high-resolution MALDI–MSI and computational methods, we mapped the lipidomic architecture of the adult mouse brain. We identified more than 500 biochemically distinct territories that align with anatomical regions and correlate with cell types^1,4,5^ and their connections.

Lipizones were able to capture cell bodies and their distant axon terminals, suggesting their potential use to support the investigation of a dimension of the brain architecture not measured by spatial transcriptomic methods.

We showed that connected brain regions, even those physically distant, share distinct lipid signatures. This observation suggests that lipidomic profiling could contribute to building a scalable label-free proxy for connectivity mapping, and positions lipids as a meaningful new layer to interrogate brain function beyond gene expression.

Our lipid analysis reveals striking^4,31^ regional diversity in white matter composition, driven predominantly by oligodendrocyte-specific lipids. We speculate that part of this diversity might relate to the chronotopic unfolding of myelin development^38^, an explanation compatible with the reported stability of myelin^37^.

We found large-scale lipid adaptations in the brains of mid-gestation pregnant female mice, with changes in the cortex possibly related to signalling and myelin production, corroborated by orthogonal experimental methods.

The enrichment of sphingomyelins in ventricles and the unexpected diversity in white matter composition suggest specialized functional roles that remain to be characterized. A further direction would be to ask whether the lipid changes induced by pregnancy persist over time, which could explain physiological differences between females that have been pregnant and virgin females. This is relevant because pregnancy is associated with lower risk of Alzheimer disease^60^, and Alzheimer disease is intimately linked to lipids^61^. Finally, separating adaptations that support maternal behaviour from those meeting fetal metabolic demands could reveal regulatory mechanisms specific to maternal-fetal neurometabolic interactions.

## Methods

### Animal studies and cryosectioning

C57BL/6J adult mice (8 weeks old or 12 weeks old in pregnancy-related studies) were used in this study. Mice were group-housed in the École Polytechnique Fédérale de Lausanne (EPFL) animal facility on 12-h light/dark cycles, at ambient temperature and humidity, with ad libitum access to food and water; pregnant female mice were maintained group-housed throughout gestation. For MALDI–MSI analysis, animals were anesthetized using isoflurane and euthanized by cervical dislocation. Brains were dissected, embedded immediately in 2% carboxymethylcellulose in deionized water and frozen on dry ice plus isopentane. Samples were stored at −80 °C until cryosectioning, during which coronal sections were cryo-sectioned with a cryotome into 10-µm sections. Sections were collected at 200-µm intervals throughout the entire brain. For sex and pregnancy comparisons, six representative regions were sampled at approximately the following stereotaxic coordinates (in millimetres, relative to interaural and bregma): (1) 5.14; 1.34; (2) 3.22; −0.58; (3) 2.34; −1.46; (4) 1; −2.80; (5) −0.92; −4.72; (6) −2.84; −6.64. All animal care and treatment procedures were performed in accordance with the Swiss guidelines and were approved by the animal ethics committee of the Canton of Vaud, Switzerland (authorization VD 3730.c).

For LC–MS and snRNA-seq, further healthy adult female and pregnant female mice (at embryonic day E13.5) were euthanized by cervical dislocation followed by decapitation to preserve lipid integrity and ensure consistency with the experimental procedures detailed in this manuscript. Brains were removed rapidly and transferred to ice-cold phosphate-buffered saline (PBS). Each brain was divided into two hemispheres for lipidomic and transcriptomic sampling. Tissue allocated to lipidomics was handled exclusively in ice-cold PBS. Tissue designated for transcriptomic analyses was processed in fresh, ice-cold, carbogenated artificial cerebrospinal fluid (93 mM N-methyl-d-glucamine, 2.5 mM KCl, 1.2 mM NaH_2_PO_4_, 30 mM NaHCO_3_, 20 mM HEPES, 25 mM D-glucose, 5 mM Na-ascorbate, 2 mM thiourea, 3 mM Na-pyruvate, 10 mM MgSO_4_ and 0.5 mM CaCl_2_; pH 7.3–7.4 adjusted with concentrated HCl). All procedures were performed using molecular-biology–grade reagents and nuclease-free water. Hemispheres were sectioned separately using a 1-mm coronal brain matrix and maintained under cold conditions throughout the procedure. After removal of the meninges, the isocortex and corpus callosum were microdissected carefully. The isocortex was separated from underlying subcortical structures along the external capsule and adjacent white matter tracts. The corpus callosum was identified along the midline as the dense dorsal commissural fibre tract located above the lateral ventricles. Corpus callosum tissue from the right hemisphere was allocated to bulk lipidomic analysis, and isocortex from the left hemisphere was allocated to transcriptomic analysis. Lipidomic samples were processed immediately. Tissue designated for transcriptomic analysis was collected into tubes containing 10 mM Tris, 250 mM sucrose, 25 mM KCl, and 5 mM MgCl_2_ (pH 8.0) supplemented with 40 U μl^−1^ RiboLock RNase inhibitor (Thermo Scientific, catalogue no. EO0281), snap-frozen, and stored at −80 °C until nuclei isolation for snRNA-seq. All procedures were conducted in accordance with Swiss guidelines and approved under authorization VD3835g by the animal ethics committee of the Canton of Vaud, Switzerland.

### MALDI–MSI experimental acquisition

Sections were dried at room temperature and coated with 2,5-dihydroxybenzoic acid (30 mg μl^−1^ in 50:50 acetonitrile:water and 0.1% trifluoroacetic acid) using the automatic SMALDIPrep (TransMIT GmbH) at 350 rpm and flow rate 5 μl min^−1^ for 30 min. Acquisitions were performed with an AP-SMALDI5 AF system coupled to a Q Exactive orbital trapping mass spectrometer. Calibration maintained mass error within ±2 ppm. For each pixel, the spectrum was accumulated from 50 laser shots at 100 Hz. MS parameters in the Tune software (Thermo Fisher Scientific) were set to the spray voltage of 4 kV, S-Lens 100 eV, capillary temperature to 250 °C. The step size of the sample stage was set to 25 μm. Positive ion mode measurements were performed in full scan mode (*m*/*z* 400–1,600) with resolving power *R* = 240,000 at *m*/*z* = 200, using internal calibration using lock mass.

### Whole-brain LC–MS/MS analysis

Four male and four female mice were euthanized with CO_2_ and decapitated. Brains were extracted rapidly and snap-frozen in liquid nitrogen, then ground in a pre-cooled mortar while adding liquid nitrogen continuously until reaching a homogeneous powder (around 5 min). The powder was aliquoted and weighed to determine wet weight. Brains were analysed by high-throughput targeted hydrophilic interaction chromatography (HILIC) tandem MS (MS/MS) lipidomics. Complex lipids were extracted with isopropanol pre-spiked with internal standards containing 75 isotopically labelled lipid species. Extracts were analysed by HILIC coupled to electrospray ionization (ESI) MS/MS (HILIC ESI–MS/MS) in positive and negative ionization modes using a TSQ Altis LC–MS/MS system. Using a dual-column setup, several lipid classes (glycerolipids, glycerophospholipids, cholesterol esters, sphingolipids and free fatty acids) were quantified. HILIC separation allowed endogenous lipids to co-elute with corresponding internal standards for matrix effect correction. Data acquisition used timed selected reaction monitoring mode with lipid-class-dependent parameters. Raw data were processed using Trace Finder Software (v.4.1) with abundance reported as estimated concentration.

### Regional LC–MS bulk lipidomics

Lipids were extracted using a methyl tert-butyl ether (MTBE)–based protocol modified from that of Matyash and colleagues^62^. In brief, 2 mg of mouse brain tissue was resuspended in 100 µl MS-grade water and 360 µl methanol and homogenized using a handheld homogenizer. An internal lipid standard mixture (5 μl; SPLASH II LIPIDOMIX, Avanti Polar Lipids), supplemented with 0.5 nmol Cer (d18:1/17:0) and 0.1 nmol glucosylceramide (GlcCer d18:1/8:0) (Avanti Polar Lipids) was added before extraction. Subsequently, 1.2 ml MTBE was added, and samples were vortexed vigorously for 1 h at 4 °C. Phase separation was induced by adding 200 µl MS-grade water, followed by centrifugation at 1,000*g* for 10 min. The upper (organic) phase was collected, and the lower (aqueous) phase was re-extracted with 400 µl MTBE/methanol/H_2_O (10:3:1.5, v/v/v). The combined organic phases were dried under a gentle stream of N_2_ gas at 40 °C and stored at −80 °C until analysis. For LC–MS analysis, dried lipid extracts were resuspended in 20% chloroform/methanol (1:1, v/v) and 80% solvent B. Mobile phase A consisted of 5 mM ammonium acetate and 0.1% formic acid in water, and mobile phase B consisted of isopropanol/acetonitrile (2:1, v/v) containing 5 mM ammonium acetate and 0.1% formic acid. Lipid separations were performed on a Shimadzu Prominence UFPLC XR system equipped with a reversed-phase Accucore C30 column (150 × 2.1 mm, 2.6 µm particle size) and a 20 mm guard column (Thermo Fisher Scientific) maintained at 40 °C, with a flow rate of 350 µl min^−1^. The injection volume was 5 µl. The gradient was linear from 50% solvent B at 0 min to 100% solvent B at 15 min, held at 100% solvent B until 21 min and then re-equilibrated at 50% solvent B for 5 min. The LC system was coupled to a hybrid Orbitrap Elite mass spectrometer (Thermo Fisher Scientific) operated with a heated ESI source. Positive- and negative-ion mode data were acquired in separate runs. MS1 spectra were acquired at 240,000 resolution over an *m*/*z* range of 350–1,700. Lipid identification and quantification were carried out using Skyline^63^ (v.21.1.0.146; MacCoss Laboratory, University of Washington). An initial transition list was generated with LipidCreator^64^ and refined manually on the basis of experimental data. The transition list included lipid species name, precursor ion, *m*/*z*, chemical formula and adduct type. Peak areas were normalized to the corresponding internal standards.

### Immunostainings and toxin staining

Fresh frozen brain sections of 10-μm thickness were dried and fixed with 4% paraformaldehyde at room temperature for 15 min, washed with 1× tris-buffered saline (TBS) for 5 min three times, followed by a secondary wash with 1× TBS + 0.3% Triton-X for 10 min three times. Next, the sections were permeabilized and blocked with 3% bovine serum albumin + 0.3% Triton-X in 1× TBS for 1 h at room temperature. Sections were stained with primary antibodies (1:200 rabbit anti-p-GR S211, Invitrogen, catalogue no. PA5-17668; 1:100 goat anti-Klk6, Invitrogen catalogue no. PA5-143104; 1:300 rabbit anti-Opalin, Alomone Labs catalogue no. ANR-040) overnight at 4 °C. After primary antibody staining, sections were washed with 1× TBS for 5 min three times. The corresponding secondary antibody (1:1,000 anti-rabbit Alexa Fluor 488, Invitrogen catalogue no. A11034; 1:1,000 anti-rabbit Alexa Fluor 647, Invitrogen catalogue no. A32795; 1:1,000 anti-goat Alexa Fluor 568, Invitrogen catalogue no. A11057) treatment was performed at room temperature for 1 h. Sections were washed with 1× TBS for 5 min three times, then probed with conjugated antibodies (1:200 mouse anti-Pgr aPR6, Invitrogen catalogue no. MA1-411 and 1:100 mouse anti-Sgk1, Abnova catalogue no. H00006446-M02 with FlexAble 2.0 CoraLite Plus 555 antibody labelling kit for Mouse IgG, Proteintech catalogue no. KFA 522; 1:250 rabbit anti-Fkbp5, Invitrogen catalogue no. 702260 with FlexAble 2.0 CoraLite Plus 647 for Rabbit IgG, Proteintech catalogue no. KFA503) for 1 h at room temperature with subsequent washing for 5 min three times. We also used mouse anti-CNPase, clone 11-5B, Alexa Fluor 488-conjugated (Abcam catalogue no. ab313960). The slides were mounted with SlowFade Glass Antifade Mountant with 4′,6-diamidino-2-phenylindole (Invitrogen, catalogue no. S36920) and scanned immediately at ×20 magnification using an Olympus VS200 whole-slide scanner. For toxin staining, fresh frozen brain sections 10 µm in thickness were brought to room temperature for 5 min, fixed with 4% paraformaldehyde at room temperature for 10 min, and washed with PBS for 5 min three times. Sections were blocked with 5% bovine serum albumin in PBS for 30 min at room temperature and then incubated overnight at 4 °C in a humidified chamber, in the dark, with fluorescently labelled lipid-binding toxins diluted 1:1,000 in blocking solution: ostreolysin A6 (OlyA6) conjugated to Alexa Fluor 488, which binds sphingomyelin–cholesterol complexes; the OlyA6 E69A mutant conjugated to Alexa Fluor 568, which binds sphingomyelin independently of cholesterol; and cholera toxin B subunit conjugated to Alexa Fluor 647, which binds GM1 and served as a negative control. Sections were washed with PBS three times, counterstained with Hoechst (1:1,000 in PBS) for 10 min at room temperature in the dark, washed with PBS three times, rinsed in distilled water, mounted and imaged by confocal microscopy.

### Registration with ABA-CCF

We registered with the ABA-CCF (CCFv.3 (ref. ^16^)). First, manual registration with Aligning Big Brains and Atlases (ABBA^65^) software used affine transforms and non-linear warping with anatomical borders guiding key feature registration. For improved accuracy, we further processed sections with STAlign^66^, using the Allen density images as reference and peak *m*/*z* = 845.528 as target (which is the most correlated with density). The resulting diffeomorphism was interpolated to reposition lipidomic pixels.

### Nuclei isolation and snRNA-seq

Nuclei were isolated from the cortex of non-pregnant and pregnant female mouse brains, using ice-cold materials and buffers. In brief, microdissected cortex tissue pieces were resuspended in 2 ml of nuclear isolation medium (NIM; 250 mM sucrose, 25 mM KCl, 5 mM MgCl_2_, 10 mM Tris-HCl pH 7.5, 5 ng ml^−1^ actinomycin D, 1 mM dithiothreitol, 100 U ml^−1^ mouse RNase inhibitor (New England Biolabs) supplemented with NP-40 (0.1% v/v). The punches were transferred into C-tubes (Miltenyi) and homogenized on a MACS OctoDissociator (Miltenyi) using the ‘4C_nuclei_1’ programme. Next, the lysate was passed through a 100-µm strainer (Miltenyi) and the strainer was washed with 1.5 ml NIM. The lysate was centrifuged at 1,000*g* for 8 min and supernatant was removed. The pellet was resuspended in 500 µl of NIM and transferred to a chilled 1.5-ml Eppendorf tube. Then, 600 µl of 50% iodixanol was added to a final gradient concentration of 30% and mixed well. The samples were centrifuged at 10,000*g* for 20 min to pellet the nuclei. The supernatant was removed completely, and nuclei were resuspended in the storage buffer (0.5% w/v bovine serum albumin, 2 mM MgCl_2_, 1 mM dithiothreitol, 200 U ml^−1^ mouse RNAse inhibitor, 5 ng ml^−1^ actinomycin D) to get a concentration of approximately 1,200 nuclei µl^−1^. The nuclei were then filtered through a 40-µm cell strainer (Flowmi, Sigma). After filtration through a 40-µm Flowmi strainer (Bel-Art), nuclei were checked for the absence of substantial doublets or aggregates and were loaded into a Chromium X (10x Genomics) chip together with beads, reverse transcription master mix reagents and oil to generate single-nuclei-containing droplets. Single-nucleus encapsulation and library preparation were performed by the Gene Expression Core Facility at EPFL. Libraries were prepared using the Chromium Next GEM Single Cell 3’ Reagent Kits v.4, following the manufacturer’s official protocol (CG000731 Rev B). The loading concentration was targeted to recover approximately 8,000 nuclei per sample. cDNA amplification was performed using 11 PCR cycles. Following quality control (assessed by Qubit and Fragment Analyser/TS4200), the final indexing and library construction were completed using 12–13 PCR cycles with the 10x Genomics Dual Index Kit TT Set A. The final snRNA-seq libraries were pooled and sequenced on an Element Biosciences AVITI24 system. Sequencing was performed to a depth of roughly 250 million reads per sample, which corresponded to an average of approximately 50,000 reads per nucleus. Read 2 length was set to 90 bp.

### Lipid brain atlas: MALDI raw data processing with uMAIA

Raw MALDI–MSI data (.raw/.udp) were first converted to imzML format using RAW2IMZML (v.1.0r47). We used uMAIA^15^ (github.com/lamanno-epfl/uMAIA/) with default settings to process all raw data collectively (peak calling, peak matching, feature normalization), including two complete individual male brains (73 sections), male and female control sections from three individuals each (29 sections) and brains of three pregnant mice (18 sections). We identified 26,874 features initially. Quality control restricted the feature space to [M + 0] isotopologues, biological compounds (greater intensity within tissue than matrix) and features present in at least four consecutive sections. Before normalization, we reduced the feature space by removing peaks not annotated in any database, peaks with Moran’s *I *< 0.4, and redundant adducts. For redundant adducts mapping to the same lipid, we kept the peak with the least dropout and the highest total signal after confirming a high correlation between different adducts on non-dropout sections. This procedure yielded 1,400 *m*/*z* peaks for normalization with uMAIA.

### Data preparation and feature selection

We extracted uMAIA-normalized *m*/*z* peak values for all pixels and acquisitions, transformed them back to linear scale, and stored them in a dataframe with spatial coordinates and ABA metadata (anatomical regions, colours and contours). We refined tissue masks using CCF borders, removing pixels mapping outside the brain volume. To completely remove features (*m*/*z* peaks) with residual batch effects, we performed the following feature selection procedure. We computed three scores: (1) difference between intra- and inter-acquisition variance, (2) spatial autocorrelation computed as Moran’s *I* (squidpy^67^) and (3) number of sections the lipid measurement dropped out. First, we removed features with dropout of more than four sections (retaining 247 of 1,400 features), then we clustered lipids in the space of these scores (standardized) with kMeans (*k* = 10), and removed the features belonging to five out of ten clusters, corresponding to visibly corrupted distributions across acquisitions, retaining 105 of 247 features. Further technical explanations of computational methods used in this study can be found in Supplementary Methods.

### Dimensionality reduction and batch integration

All differential testing and downstream analyses of the manuscript were performed directly on the uMAIA output. The dimensionality reduction and harmonization described below was used solely for clustering where strong coembedding is desired.

Dimensionality reduction was performed fitting NMF on brain 1, providing interpretable ‘global’ embeddings.

NMF seeks two low-rank non-negative matrices,

$$W\in {R}_{\ge 0}^{m\times k}$$

and

$$H\in {R}_{\ge 0}^{k\times n},$$

whose product approximates the original non-negative data matrix

$$V\in {R}_{\ge 0}^{m\times n}:$$

$$V\approx {WH}$$

Here *m* is the number of pixels, *n* is the number of lipids and *k* is the number of NMF factors. The number of NMF factors was selected as the number of lipid Leiden clusters maximizing the score:

$${Q}^{\alpha }\log (1+C)$$

where *Q* is the modularity, which quantifies the strength of division of a network into clusters, *C* the number of clusters and *α* a tunable parameter that controls the relative influence of modularity versus having a large number of clusters (default = 0.7). This yielded 16 lipid Leiden clusters, that is, 16 NMF factors. The NMF component matrix (left-hand side, W matrix) was seeded^68^ with the lipid closest to the centroid for each cluster of lipids and the coefficient matrix with the zero-clipped cosine similarity between each lipid and the central one. We verified embedding robustness to random feature downsampling. Once the *W* and *H* matrices were learnt from brain 1, the *H* matrix was kept fixed and the *W* matrices were determined for the other brains; Harmony^18^ was applied on the *W* matrices. We also produced a low-rank, batch effect-corrected dataset by multiplying the component with the coefficient matrix. This was used for the local embedding recomputation during the clustering procedure described below. The coefficient matrix was *z*-standardized.

### Top-down binary splitting algorithm and label transfer

We implemented a top-down hierarchical binary splitter. The algorithm splits the set of input pixels in two recursively. To perform each of the split balancing local and global variations, the globally computed *W* matrix (NMF coefficients for each pixel) is concatenated with the *W* matrix of the pixels of the parent split and of the current split, that is, the NMF is recomputed iteratively at each split. The optimal binary split is found using the backSPIN^69^ approach, which is sped up by using aggregated pixel clusters instead of individual pixels (*k* = 60 clusters using kMeans).

A stop condition is implemented so that the splitting is halted if one of the following conditions is not satisfied: (1) differentially expressed features (inspired by Yao and colleagues^4^); (2) minimal size of clusters; or (3) coherence between consecutive sections. For differential expression, two-sided Mann–Whitney *U* test with BH-FDR-correction required at least 2 of 105 differential peaks at 0.2 absolute fold change and an adjusted *P* value of 0.05. The minimal size of a cluster was set to 200 pixels. For coherence between consecutive sections, we impose that each cluster must span at least two consecutive or four total acquisitions.

To make the split robust (that is, not instance-dependent) and to allow accurate label transfer of new datasets, at each split we trained an XGBoost binary classifier to perform the split from the embeddings. XGBoost classifiers (class rebalancing, 1,000 estimators, maximum depth 8, learning rate 0.02, 0.8 subsampling and colsample, and gamma 0.5) used early stopping on validation set (a set of held-out sections spanning the antero-posterior axis) accuracy. The XGBoost classifications on the training, validation and test sets were used as cluster labels. By this procedure, our clustering method inherently builds a transferrable annotation tree: a new pixel can be annotated by applying the local NMF to obtain its embeddings, followed by XGBoost classification, moving iteratively from the root to a terminal leaf (lipizone) of the binary tree.

To assess lipizone reproducibility and label transfer accuracy across brains, we compared the regional distributions of lipizones against the reference atlas using Pearson’s *R*.

### Lipizone assessment, naming and visualization

We compared our clustering approach with Leiden clustering (resolution = 14.0) inspecting the confusion matrix. Results were qualitatively confirmed by visualizing the clusters with matching colours.

Lipizones were named on the basis of the ABA anatomical acronyms where they were found. Specifically, we calculated a reciprocal enrichment score from the confusion matrix of lipizones and ABA acronyms by element-wise multiplication of the enrichment of lipizones in acronyms and the enrichment of acronyms in lipizones. Lipizone colours were chosen to allow the visualization of several lipizones in the same view. To maximize contrast, we coloured the lipizones concatenating eight colourmaps, one for each of the eight lipizone classes, and assigned the colours based of the lipizone ‘leaves’ of the hierarchy. Contiguous leaf lipizones were distanced along the colourmap proportionally to their cosine similarity.

### Feature restoration

We used XGBoost regression to impute features discarded during selection (the features for which measurement dropped out in a non-negligible number of sections). Features with Moran’s *I* ≥ 0.4 in at least four sections were candidates for restoration. For each feature, we selected the three best sections by Moran’s I for training and the fourth for testing. XGBoost models were trained to predict discarded features from the 16 Harmony-corrected NMF embeddings. Models yielding Pearson’s *R* ≥ 0.4 on held-out test sections were accepted and applied to all sections, resulting in 692 *m*/*z* peaks restored.

### Lipid annotation

For an accurate annotation of *m*/*z* peaks, we integrated several reference sources: three bulk lipidomic experiments (ESI LC–MS with two different chromatographic columns, HILIC and Reverse Phase, and LC–MS/MS), METASPACE annotations (using the Human Metabolome Database), the LIPIDMAPS database and two published brain MS datasets (those of Fitzner and colleagues^9^ (LC–MS) and Vandenbosch and colleagues (MALDI–MSI^11^)). We matched raw peak *m*/*z* values to reference annotations within 5 ppm, harmonizing nomenclature with goslin^70^ when needed. For MALDI–MSI, we considered [M + H]^+^, [M + Na]^+^, [M + K]^+^ and [M + NH_4_]^+^ adducts; for in-house LC–MS/MS, we used [M + H]^+^ and [M + NH_4_]^+^ in positive mode and [M+AcO]^−^, [M − 2H]^−^ and [M − H]^−^ in negative mode.

We assigned confidence-weighted scores to each annotation: LC–MS/MS (8), ESI LC–MS (2), published studies (1), METASPACE (1 if FDR < 0.05, otherwise 0.5) and LIPIDMAPS (0, annotation transferred but unconfirmed in brain). Peak names were further prioritized using an independent quantitative LC–MS/MS dataset for sex-specific brain lipidomics (Supplementary Table 1). In cases of naming ambiguity, we prioritized lipid names covering more than 80% molar fraction among candidates; otherwise, we reported several names as the species designation. We provide detailed annotation tables (Supplementary Table 2a,b) motivating every lipid name where an overlap of an abundant and a less abundant species is possible.

We note that, in positive ion mode, a phosphatidylcholine that loses its entire choline moiety through in-source fragmentation yields an ion isobaric with the protonated phosphatidic acid of the same acyl composition; at the MS1 resolution of imaging experiments the PC in-source fragment and a genuine PA are therefore indistinguishable^71^. PG and PI species are detected and quantified in negative-mode LC–MS/MS in this study; we note that their negative-mode detection corroborates that the species exist in brain tissue but does not, on its own, establish that any specific positive-mode MALDI–MSI peak at a PG/PI *m*/*z* represents that species.

Peaks with annotation scores greater than four (172 nonredundant lipids) were considered unambiguous and used for the analysis in this work. The final dataset includes several lipid classes (LPC, LPE, LPA, LPS, LPG, LPI, PC, PE, PI, PS, PA, PG, SM, Cer, HexCer, Hex2Cer, TG, DG) with variable chain lengths and unsaturation degrees. As MALDI–MSI cannot distinguish isomers, we report the sum composition of carbon atoms and unsaturations, meaning an ‘individual’ lipid may comprise several isomers. The PI and PG annotations are only suggestive (based on the *m*/*z*) but should not be considered reliable in our positive ion mode MALDI–MSI data.

### Data analysis and visualization methods

For visualization, we embedded our dataset in *t*-SNE space (perplexity 30) using *z*-standardized, harmonized NMF embeddings.

The 3D interpolation used radially exponentially decaying weighted neighbours with ABA regions as anatomical guides. To render, explore and film 3D maps, we used Napari^72^ and napari_animation (Supplementary Data File 1).

We assessed the residual within-cluster variability of each lipizone on brain 1 as the log determinant of the covariance matrix of that lipizone, after *z*-scaling lipids across the entire brain. We processed cell-type-specific bulk lipidomics data from Fitzner and colleagues^9^ using minimum–maximum normalization. For the oligodendrocyte score used in the white matter analysis, cell-type deconvolution used linear regression by non-negative least squares, with coefficients normalized to sum to 1 across cell types per lipizone. Differential lipid testing between groups used two-sided Mann–Whitney *U* test with BH correction (*α* = 0.05). A two-sided permutation test was used to evaluate lipid class enrichment.

For the similarity range score comparing the two ChP principal lipizones (ChP1–ChP2) against all other brain lipizones, for each lipizone centroid, we computed the Euclidean distances to both ChP lipizone centroids in lipidomic space restricting to differential lipids. We then expressed this as a relative distance ratio, where values below 0.5 indicated greater similarity to the first ChP lipizone and values above 0.5 to the second.

### Lipid brain atlas explorer

The Lipid Brain Atlas Explorer (https://lbae-v2.epfl.ch/) is a Python-based interactive web application built using the Dash^73^ framework for visualizing and analysing high-resolution mass spectrometry imaging data of lipids across the mouse brain.

### Sample size, randomization and blinding

No statistical method was used to predetermine sample size. For the atlas backbone, two C57BL/6J male mice were densely sampled at 200-μm intervals throughout the entire brain (73 sections overall), with brain 1 used as the primary reference and brain 2 as an independent replicate to verify cluster and discovery reproducibility and label transfer performance. Sample sizes for the sex and pregnancy comparisons (*n* = 3 male mice, *n* = 3 non-pregnant female mice, and *n* = 3 pregnant female mice at E13.5; 29 control sections and 18 pregnancy sections) follow current conventions for comprehensive sampling in spatial transcriptomic mouse brain atlas studies. Sufficiency is supported by the successful reproduction of cluster spatial organization across all 11 brains as biological replicates (Supplementary Fig. 4d). Two additional sparsely sampled pregnant female mice were used for the PCA of pregnancy versus between-sex differences; a further four pregnant and four non-pregnant female mice were used for bulk LC–MS, two pregnant and two non-pregnant female mice were used for for snRNA-seq, and two pregnant and two non-pregnant female mice were used for immunostaining. Sections that were damaged or had low MALDI yield were excluded; these remain available as raw data on METASPACE.

The main covariates studied were sex and pregnancy, and mice were homogeneous in age. Samples were processed in a random order on MALDI–MSI; all pregnancy samples were processed after the male and female samples.

Formal blinding was not implemented in this study, in line with ARRIVE v.2.0 reporting recommendations for atlas-scale observational designs. However, different investigators performed distinct experimental stages: tissue collection and sectioning; MALDI–MSI acquisition; and downstream computational analyses. Tissue processing and acquisition used standardized protocols and predefined acquisition parameters, and downstream analyses relied on objective, quantitative lipidomic outputs generated by computational pipelines rather than on subjective scoring. Group assignments (sex, pregnancy) are intrinsic biological properties of the animals rather than experimental treatments allocated by the investigators, and are visibly apparent at the time of dissection; for the immunostaining quantification (Figs. 4n–o and 5o–p and Extended Data Fig. 11g), per-image segmentation and intensity readouts were generated by fixed Cellpose3, Spotiflow and Otsu pipelines with parameters set on a single representative sample before group identity entered any analysis.

### Use of large language models

Large language models were used for code writing, manuscript drafting and editing.

### Reporting summary

Further information on research design is available in the Nature Portfolio Reporting Summary linked to this article.

## Online content

Any methods, additional references, Nature Portfolio reporting summaries, source data, extended data, supplementary information, acknowledgements, peer review information; details of author contributions and competing interests; and statements of data and code availability are available at https://doi.org/10.1038/s41586-026-11050-0.

## Supplementary information

Supplementary Information Supplementary Methods and Figs. 1–13.

Reporting Summary

Supplementary Tables Supplementary Tables 1–11.

Supplementary Video 1 Supplementary Data File 1. 3D rendering of SM 34:1;O2 (whole-brain reconstruction video).

Supplementary Data File 2 Validation of lipizones by alternative clustering methods. Multi-page PDF report. Each page documents one alternative clustering regime, replicating the main findings of the paper with confusion matrices, spatial plots and replications of key figure panels (including Figs. 3j, 4b,k,l and 5e).

Peer Review File

## Data Availability

All data are granted public access. Raw data are available at Zenodo (for Zenodo repository numbers, see Supplementary Table 11); iMZML and IBD datasets are available on METASPACE (https://metaspace2020.org/project/mlba-2025; click on ‘See all datasets’ at the bottom of the page to reveal all 132 datasets); and processed data are available at Zenodo (https://doi.org/10.5281/zenodo.15379499, https://doi.org/10.5281/zenodo.15379534 and https://doi.org/10.5281/zenodo.15379565)^74–76^ in various formats including all metadata and in AnnData h5ad format for integration with the scverse ecosystem^77^. Raw confocal stacks for the immunofluorescence experiments (Figs. 4n–o and 5o–p and Extended Data Fig. 11g) are available at Zenodo (https://zenodo.org/records/20702309 and https://zenodo.org/records/20703205)^78,79^. The snRNA-seq dataset is available on GEO under accession code GSE325586. Interactive visualizations are available on our website (https://lbae-v2.epfl.ch/). Public databases used in this study are as follows: LIPID MAPS Structure Database (https://www.lipidmaps.org/databases/lmsd/), Human Metabolome Database (https://hmdb.ca/), METASPACE (https://metaspace2020.org/), LION/web Lipid Ontology v.2020-08-07 (http://www.lipidontology.com/), LINEX2 (https://gitlab.lrz.de/lipitum-projects/linex), Rhea reaction knowledgebase (https://www.rhea-db.org/), adapted: see Supplementary Table 10, Omnipath (https://omnipathdb.org/), SIGNOR (https://signor.uniroma2.it/), LIANA (https://liana-py.readthedocs.io/en/latest/), SCENIC+ (https://scenicplus.readthedocs.io/en/latest/), Mouse Genome Informatics (http://www.informatics.jax.org/), and MSigDB v.2025.1 (https://www.gsea-msigdb.org/gsea/msigdb/). The following published datasets were reused: Yao and colleagues^4^ (10x single-cell RNA sequencing and MERFISH, available through the Allen Brain Cell Atlas https://portal.brain-map.org/atlases-and-data/bkp/abc-atlas), Langlieb and colleagues^1^ (Slide-seq2, https://www.braincelldata.org/), Oh and colleagues^3^ (Allen Mouse Brain Connectivity Atlas, https://connectivity.brain-map.org/), Wang and colleagues^16^ (Allen Mouse Brain CCFv3, https://atlas.brain-map.org/), Fitzner and colleagues^9^ (bulk lipidomics; accession in original publication), Xie and colleagues^12^ (single-cell rat lipidomics; accession in original publication), and Bhaduri and colleagues^24^ (single-cell human cortex lipidomics; accession in original publication).

## Code availability

EUCLID methods and tutorials are available as a PyPI package and a GitHub repository at https://github.com/lamanno-epfl/EUCLID. The package is in active development and currently tested on a limited number of scenarios. The code to replicate the analysis of the paper and explore the dataset is available at GitHub https://github.com/lamanno-epfl/lipidbrainatlas. The code for lipiMap and the code to build the explorer are also available as separate GitHub repositories (https://github.com/lamanno-epfl/lipiMap and https://github.com/lamanno-epfl/lbae). All code is available at Zenodo (https://doi.org/10.5281/zenodo.20702555)^80^.
