# Supplementary material for: The lipidomic architecture of the mouse brain
Source: Nature. 2026 Sep 23;658(8134):205–17. doi: 10.1038/s41586-026-11050-0 (PMC13626954; doi:10.1038/s41586-026-11050-0)
Supplement: Supplementary file 1 [file 41586_2026_11050_MOESM2_ESM.pdf]

nature portfolio

Corresponding author(s): Gioele La Manno, Giovanni D'Angelo

Last updated by author(s): 16/05/2026

Reporting Summary

Nature Portfolio wishes to improve the reproducibility of the work that we publish. This form provides structure for consistency and transparency in reporting. For further information on Nature Portfolio policies, see our Editorial Policies and the Editorial Policy Checklist.

Statistics

For all statistical analyses, confirm that the following items are present in the figure legend, table legend, main text, or Methods section.

n/a	Confirmed	
☐	☒	The exact sample size (*n*) for each experimental group/condition, given as a discrete number and unit of measurement
☐	☒	A statement on whether measurements were taken from distinct samples or whether the same sample was measured repeatedly
☐	☒	The statistical test(s) used AND whether they are one- or two-sided<br>*Only common tests should be described solely by name; describe more complex techniques in the Methods section.*
☐	☒	A description of all covariates tested
☐	☒	A description of any assumptions or corrections, such as tests of normality and adjustment for multiple comparisons
☐	☒	A full description of the statistical parameters including central tendency (e.g. means) or other basic estimates (e.g. regression coefficient) AND variation (e.g. standard deviation) or associated estimates of uncertainty (e.g. confidence intervals)
☐	☒	For null hypothesis testing, the test statistic (e.g. *F*, *t*, *r*) with confidence intervals, effect sizes, degrees of freedom and *P* value noted<br>*Give P values as exact values whenever suitable.*
☐	☒	For Bayesian analysis, information on the choice of priors and Markov chain Monte Carlo settings
☒	☐	For hierarchical and complex designs, identification of the appropriate level for tests and full reporting of outcomes
☐	☒	Estimates of effect sizes (e.g. Cohen's *d*, Pearson's *r*), indicating how they were calculated

Our web collection on statistics for biologists contains articles on many of the points above.

Software and code

Policy information about availability of computer code

Data collection	Raw MALDI-MSI data were converted from .raw/.udp to imzML using RAW2IMZML (v1.0r47). MALDI-MSI acquisition used Thermo Tune (v2.13) on the AP-SMALDI5 AF / Q Exactive system and Bruker timsControl (v7.0.5) on the TimsTOF flex. Bulk LC-MS/MS data were acquired with Thermo Xcalibur and processed with Trace Finder Software (v4.1). Regional bulk LC-MS data were acquired on a Shimadzu Prominence UFPLC XR system coupled to a Thermo Orbitrap Elite. snRNA-seq libraries were prepared with the Chromium Next GEM Single Cell 3' Reagent Kit v4 (GEMX) and sequenced on an Element Biosciences AVITI24. Immunofluorescence images were acquired on an Olympus VS200 whole slide scanner (Olympus VS200 ASW).
Data analysis	MALDI data preprocessing: uMAIA (https://github.com/lamanno-epfl/uMAIA/). CCF registration: ABBA (https://abba-documentation.readthedocs.io/) and STalign (https://github.com/JEFworks-Lab/STalign). Lipid annotation: METASPACE, LIPID MAPS, LION/web (v2020-08-07), goslin, LINEX2. LC-MS/MS data analysis: Skyline (v21.1.0.146), LipidCreator. snRNA-seq data analysis: Cell Ranger (v10.0.0, 10x Genomics) with GRCm39-2024-A reference; Scrublet; Seurat (v3); scanpy; scIB; MapMyCells; CellBender (v0.3.0); gseapy; MSigDB v2025.1. Probabilistic modeling: NumPyro, PyTorch (v2.2.1). Machine learning: XGBoost, SHAP (SHapley Additive exPlanations), scikit-learn. Network inference: CORNETO, Omnipath, SIGNOR, Dorothea, Rhea database. Visualization: Napari, napari_animation, Dash. Image analysis: Cellpose3, Spotiflow. Custom pipeline (this study): EUCLID (https://github.com/lamanno-epfl/EUCLID), lipiMap (https://github.com/lamanno-epfl/lipiMap). Code to reproduce all analyses: https://github.com/lamanno-epfl/lipidbrainatlas. Web explorer: LBAE (https://github.com/lamanno-epfl/lbae).

For manuscripts utilizing custom algorithms or software that are central to the research but not yet described in published literature, software must be made available to editors and reviewers. We strongly encourage code deposition in a community repository (e.g. GitHub). See the Nature Portfolio guidelines for submitting code & software for further information.

Data

Policy information about availability of data

All manuscripts must include a data availability statement. This statement should provide the following information, where applicable:

- Accession codes, unique identifiers, or web links for publicly available datasets
- A description of any restrictions on data availability
- For clinical datasets or third party data, please ensure that the statement adheres to our policy

All data generated in this study is granted public access. Raw data are available on Zenodo (see Supplementary Table 11); iMZML and IBD datasets are available on METASPACE at https://metaspace2020.org/project/mlba-2025 (click on "See all datasets" at the bottom of the page to reveal all 132 datasets); processed data is on Zenodo at https://doi.org/10.5281/zenodo.15379499, https://doi.org/10.5281/zenodo.15379534, https://doi.org/10.5281/zenodo.15379565, in various formats including all metadata. The snRNA-seq dataset is available on GEO at (GSE325586). Interactive visualizations are available on our website at https://lbae-v2.epfl.ch/. Public databases used in this study: LIPID MAPS Structure Database (https://www.lipidmaps.org/databases/lmsd/), Human Metabolome Database (HMDB, https://hmdb.ca/), METASPACE (https://metaspace2020.org/), LION/web Lipid Ontology v2020-08-07 (http://www.lipidontology.com/), LINEX2 (https://exbio.wzw.tum.de/linex/), Rhea reaction knowledgebase (https://www.rhea-db.org/), Omnipath (https://omnipathdb.org/), SIGNOR (https://signor.uniroma2.it/), LIANA (https://liana-py.readthedocs.io/en/latest/), SCENIC+ (https://scenicplus.readthedocs.io/en/latest/), Mouse Genome Informatics (MGI, http://www.informatics.jax.org/), MSigDB v2025.1 (https://www.gsea-msigdb.org/gsea/msigdb/). The following published datasets were used: Yao et al. 2023 (10x scRNA-seq and MERFISH, available via Allen Brain Cell Atlas https://portal.brain-map.org/atlases-and-data/bkp/abc-atlas), Langlieb et al. 2023 (Slide-seq2, https://www.braincelldata.org/), Oh et al. 2014 (Allen Mouse Brain Connectivity Atlas, https://connectivity.brain-map.org/), Wang et al. 2020 (Allen Mouse Brain CCFv3, https://atlas.brain-map.org/), Fitzner et al. 2020 (bulk lipidomics; accession in original publication), Xie et al. 2024 (single-cell rat lipidomics; accession in original publication), Bhaduri et al. 2021 (single-cell human cortex lipidomics; accession in original publication).

Research involving human participants, their data, or biological material

Policy information about studies with human participants or human data. See also policy information about sex, gender (identity/presentation), and sexual orientation and race, ethnicity and racism.

Reporting on sex and gender	N/A
Reporting on race, ethnicity, or other socially relevant groupings	N/A
Population characteristics	N/A
Recruitment	N/A
Ethics oversight	N/A

Note that full information on the approval of the study protocol must also be provided in the manuscript.

Field-specific reporting

Please select the one below that is the best fit for your research. If you are not sure, read the appropriate sections before making your selection.

☒ Life sciences ☐ Behavioural & social sciences ☐ Ecological, evolutionary & environmental sciences

For a reference copy of the document with all sections, see nature.com/documents/nr-reporting-summary-flat.pdf

Life sciences study design

All studies must disclose on these points even when the disclosure is negative.

Sample size	No statistical method was used to predetermine sample size. For the atlas backbone, two C57BL/6J male mice were densely sampled at 200 µm intervals throughout the entire brain (73 sections overall) with brain #1 used as the primary reference and brain #2 as an independent replicate to verify cluster and discovery reproducibility, and label-transfer performance. Sample sizes for the sex and pregnancy comparisons (n=3 males, n=3 non-pregnant females, n=3 pregnant females at E13.5; 29 control sections and 18 pregnancy sections) follow the current conventions on order of magnitude for comprehensive sampling in spatial transcriptomic mouse brain atlas studies. Sufficiency is supported by successful cluster spatial organization reproduction across all 11 brains as biological replicates (Supplementary figure 4d). 2 extra sparsely sampled pregnant females samples were used for PCA analysis of pregnancy vs between-sex differences; an additional 4 pregnant + 4 non-pregnant females for bulk LC-MS, and 2 pregnant + 2 non-pregnant females for snRNA-seq and 4 pregnant + 4 non-pregnant females for immunostaining as described in the Methods.
Data exclusions	Sections that were damaged or with low MALDI yield were excluded, but are available on METASPACE as raw data.
Replication	MALDI-MSI clustering was developed on densely-sampled brain #1 and independently validated on densely-sampled brain #2; reclustering brain #2 from scratch and label-transferring from brain #1 produced broadly concordant results (Supplementary figure 3e). Lipizone reproducibility was further assessed across 11 biological replicates by Allen-region-resolved Pearson correlation of relative lipizone

	abundance, with consistent regional distributions across animals (Supplementary figure 4d). All 25+ alternative clustering regimes tested reproduced the major findings reported in the paper (Supplementary File 2).
Randomization	The main covariates studied were sex and pregnancy. Mice were homogeneous in terms of age. Samples were processed in a random order on MALDI-MSI; all pregnancy samples were processed after the male and female samples.
Blinding	Formal blinding was not implemented in this study, in line with current recommendations for atlas-scale observational designs. Different investigators performed distinct experimental stages, tissue collection and sectioning, MALDI-MSI acquisition, and downstream computational analyses, which limits observer-dependent influence across the workflow. Tissue processing and acquisition used standardised protocols and predefined acquisition parameters, and downstream analyses relied on objective, quantitative lipidomic outputs generated by computational pipelines rather than on subjective scoring. Group assignments (sex, pregnancy) are intrinsic biological properties of the animals rather than experimental treatments allocated by the investigators, and are visibly apparent at the time of dissection.

Reporting for specific materials, systems and methods

We require information from authors about some types of materials, experimental systems and methods used in many studies. Here, indicate whether each material, system or method listed is relevant to your study. If you are not sure if a list item applies to your research, read the appropriate section before selecting a response.

Materials & experimental systems

n/a	Involved in the study
☐	☒ Antibodies
☒	☐ Eukaryotic cell lines
☒	☐ Palaeontology and archaeology
☐	☒ Animals and other organisms
☒	☐ Clinical data
☒	☐ Dual use research of concern
☒	☐ Plants

Methods

n/a	Involved in the study
☒	☐ ChIP-seq
☒	☐ Flow cytometry
☒	☐ MRI-based neuroimaging

Antibodies

Antibodies used	Primary antibodies. Anti-phospho-Glucocorticoid Receptor (Ser211), rabbit polyclonal (Invitrogen #PA5-17668), 1:200, raised against a synthetic phosphopeptide corresponding to residues surrounding Ser211 of human GR. Anti-Kallikrein 6, goat polyclonal (Invitrogen #PA5-143104), 1:100, raised against peptide C-KTADGDFPDTIQ. Anti-Opalin, rabbit polyclonal (Alomone Labs #ANR-040), 1:300, raised against peptide (C)DNPKISENPRRSPTHE corresponding to residues 79–94 of mouse Opalin (intracellular C-terminus). Anti-Progesterone Receptor, mouse monoclonal clone Alpha PR6 (Invitrogen #MA1-411), 1:200, raised against progesterone receptor purified from chick oviduct cytosol; subsequently conjugated in-house with FlexAble CoraLite Plus 555 (Proteintech #KFA522). Anti-SGK1, mouse monoclonal clone 3E3 (Abnova #H00006446-M02), 1:100, raised against a partial human SGK1 recombinant protein (a.a. 1–~90); conjugated in-house with FlexAble CoraLite Plus 555 (Proteintech #KFA522). Anti-FKBP5, rabbit recombinant monoclonal clone 6H8L4 (Invitrogen #702260), 1:250; conjugated in-house with FlexAble CoraLite Plus 647 (Proteintech #KFA503). Anti-CNPase, mouse monoclonal clone 11-5B directly conjugated with Alexa Fluor 488 (Abcam ab313960). Secondary antibodies. Goat anti-rabbit Alexa Fluor 488 (Invitrogen #A11034, 1:1000); goat anti-rabbit Alexa Fluor 647 (Invitrogen #A32795, 1:1000); donkey anti-goat Alexa Fluor 568 (Invitrogen #A11057, 1:1000).
Validation	PA5-17668 (anti-pGR Ser211): manufacturer-validated for WB, ICC/IF and IP in human and mouse samples. Specificity is restricted to glucocorticoid receptor phosphorylated at Ser211 (the canonical activity marker) and the antibody is non-cross-reactive with other unrelated phosphorylated proteins. PA5-143104 (anti-Klk6, goat polyclonal): manufacturer-tested in ICC/IF, IHC-P and Flow; reactivity officially listed as human. We used it on mouse tissue, where the immunogen peptide (C-KTADGDFPDTIQ) is conserved with mouse KLK6. ANR-040 (anti-Opalin, rabbit polyclonal): raised against mouse Opalin and validated by the manufacturer in mouse brain by WB (rat and mouse brain lysates) and IF on free-floating frozen mouse brain sections, with specificity confirmed by blocking-peptide pre-incubation. MA1-411 (anti-PR Alpha PR6, mouse monoclonal): cited in >50 publications across human, mouse, rat, rabbit, bovine, ovine, chicken, and guinea pig samples; non-cross-reactive with estrogen receptor or glucocorticoid receptor. Validated for IF/ICC and IHC-F by the manufacturer. H00006446-M02 (anti-SGK1 clone 3E3, mouse monoclonal): manufacturer-tested in WB, ICC/IF, IHC-P and ELISA; reactivity officially listed as human. 702260 (anti-FKBP5 clone 6H8L4, rabbit recombinant monoclonal): manufacturer-tested in WB; recombinant-rabbit format provides lot-to-lot consistency. Manufacturer-predicted to react with monkey, pig, rabbit, and sheep. anti-CNPase clone 11-5B, mouse monoclonal: validated by the manufacturer in human HAP1 cells, with >140 published uses across human, mouse and rat.

Animals and other research organisms

Policy information about [studies involving animals](); [ARRIVE guidelines]() recommended for reporting animal research, and [Sex and Gender in Research]()

Laboratory animals	C57BL/6J mice, 8 weeks old for the male/female atlas backbone and sex comparison; 12 weeks old at sacrifice for the pregnancy cohort (mid-gestation, embryonic day E13.5; first pregnancy).

Wild animals	No wild animals were used in this study.
Reporting on sex	Main clustering was performed on male animals, differences between sexes are studied in a dedicated part of the manuscript.
Field-collected samples	No field-collected samples were used in this study.
Ethics oversight	All animal care and treatment procedures were performed in accordance with the Swiss guidelines and were approved by the Canton of Vaud SCAV (authorization VD 3730.c). Mice for LC-MS/MS were donated from Professor Johannes Gräff's laboratory (license #3954).

Note that full information on the approval of the study protocol must also be provided in the manuscript.

Plants

Seed stocks	N/A
Novel plant genotypes	N/A
Authentication	N/A
