# Supplementary material for: The lipidomic architecture of the mouse brain
Source: Nature. 2026 Sep 23;658(8134):205–17. doi: 10.1038/s41586-026-11050-0 (PMC13626954; doi:10.1038/s41586-026-11050-0)

1 Leiden on harmonized NMF

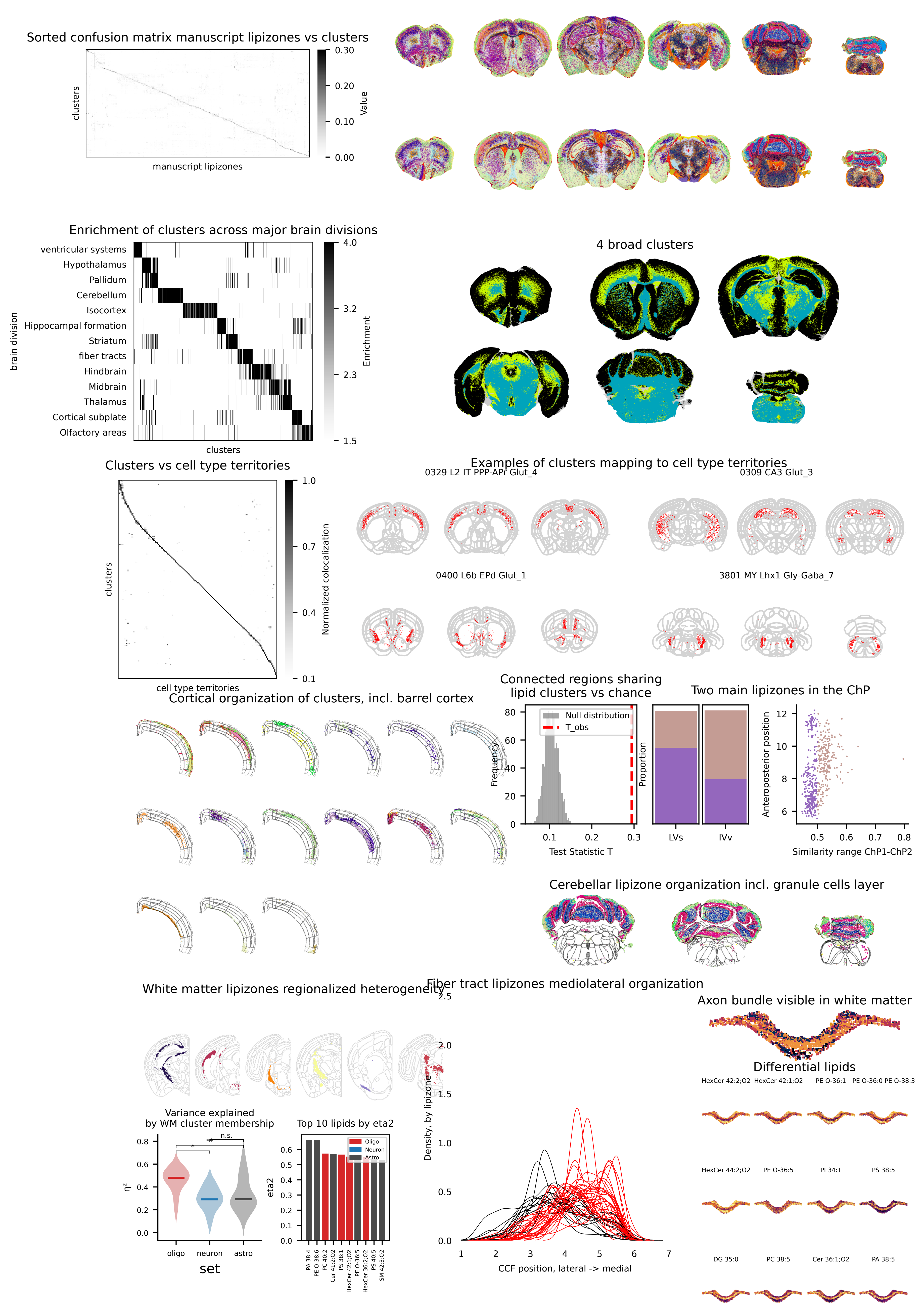

2 Louvain on harmonized NMF

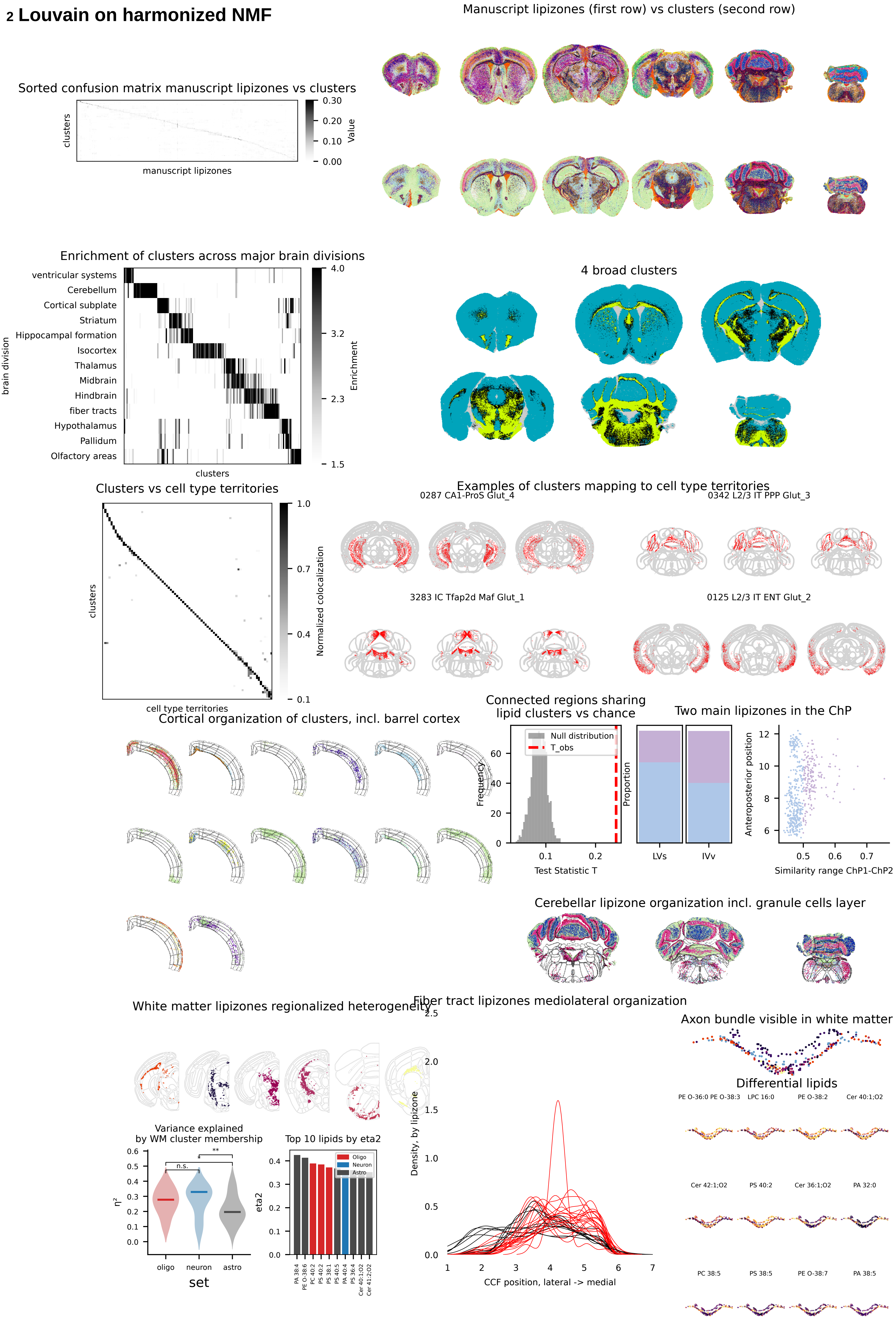

3 Splitter feature downsampling 75 iter 56

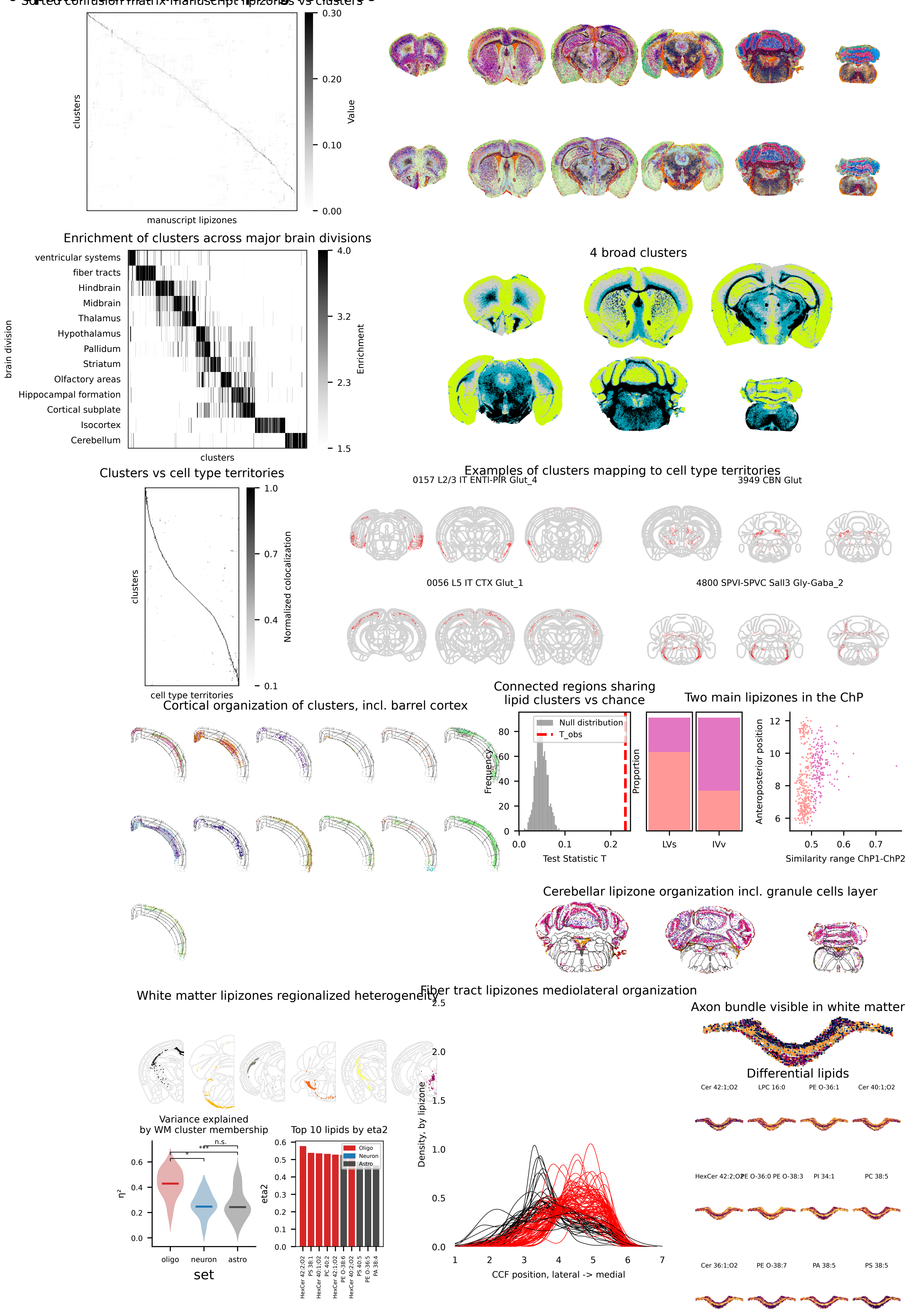

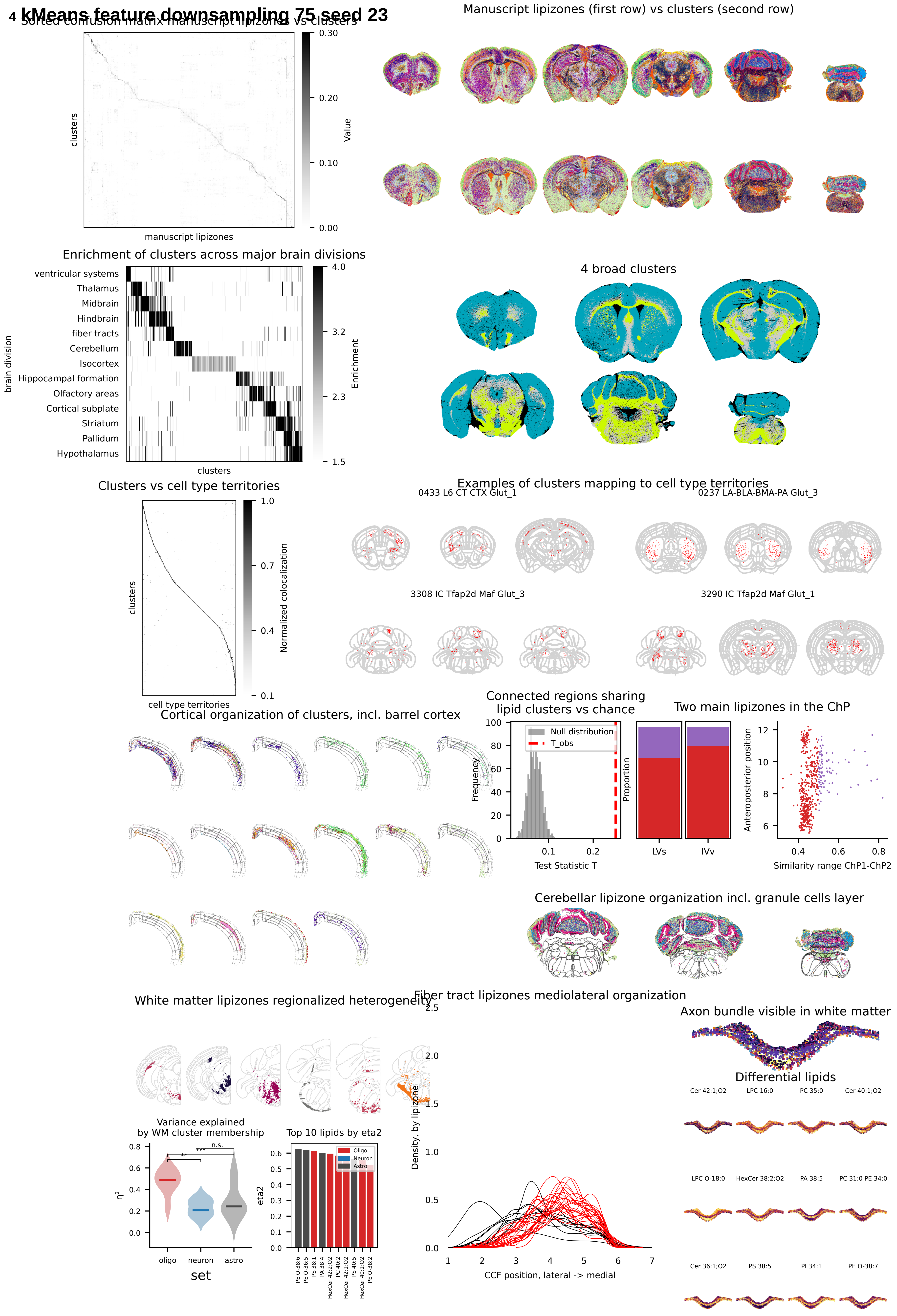
4 kMeans feature downsampling 75 seed 23
Sorted confusion matrix manuscript lipizones vs clusters
clusters
manuscript lipizones
Value
Manuscript lipizones (first row) vs clusters (second row)
Enrichment of clusters across major brain divisions
ventricular systems
Thalamus
Midbrain
Hindbrain
fiber tracts
Cerebellum
Isocortex
Hippocampal formation
Olfactory areas
Cortical subplate
Striatum
Pallidum
Hypothalamus
brain division
clusters
Enrichment
4 broad clusters
Clusters vs cell type territories
cell type territories
Normalized colocalization
Examples of clusters mapping to cell type territories
0433 L6 CT CTX Glut_1
0237 LA-BLA-BMA-PA Glut_3
3308 IC Tfap2d Maf Glut_3
3290 IC Tfap2d Maf Glut_1
Cortical organization of clusters, incl. barrel cortex
Connected regions sharing lipid clusters vs chance
Null distribution
T_obs
Frequency
Test Statistic T
Proportion
LVs
IVv
Two main lipizones in the ChP
Anteroposterior position
Similarity range ChP1-ChP2
Cerebellar lipizone organization incl. granule cells layer
White matter lipizones regionalized heterogeneity
Fiber tract lipizones mediolateral organization
Density, by lipizone
CCF position, lateral -> medial
Axon bundle visible in white matter
Differential lipids
Cer 42:1;O2
LPC 16:0
PC 35:0
Cer 40:1;O2
LPC O-18:0
HexCer 38:2;O2
PA 38:5
PC 31:0 PE 34:0
Cer 36:1;O2
PS 38:5
PI 34:1
PE O-38:7
Variance explained by WM cluster membership
η²
oligo
neuron
astro
set
n.s.
Top 10 lipids by eta2
Oligo
Neuron
Astro
eta2
PE O-38:6
PE O-36:5
PS 38:1
PA 38:4
HexCer 42:2;O2
PC 40:2
HexCer 42:1;O2
PE 40:5
HexCer 40:1;O2
PE O-38:2

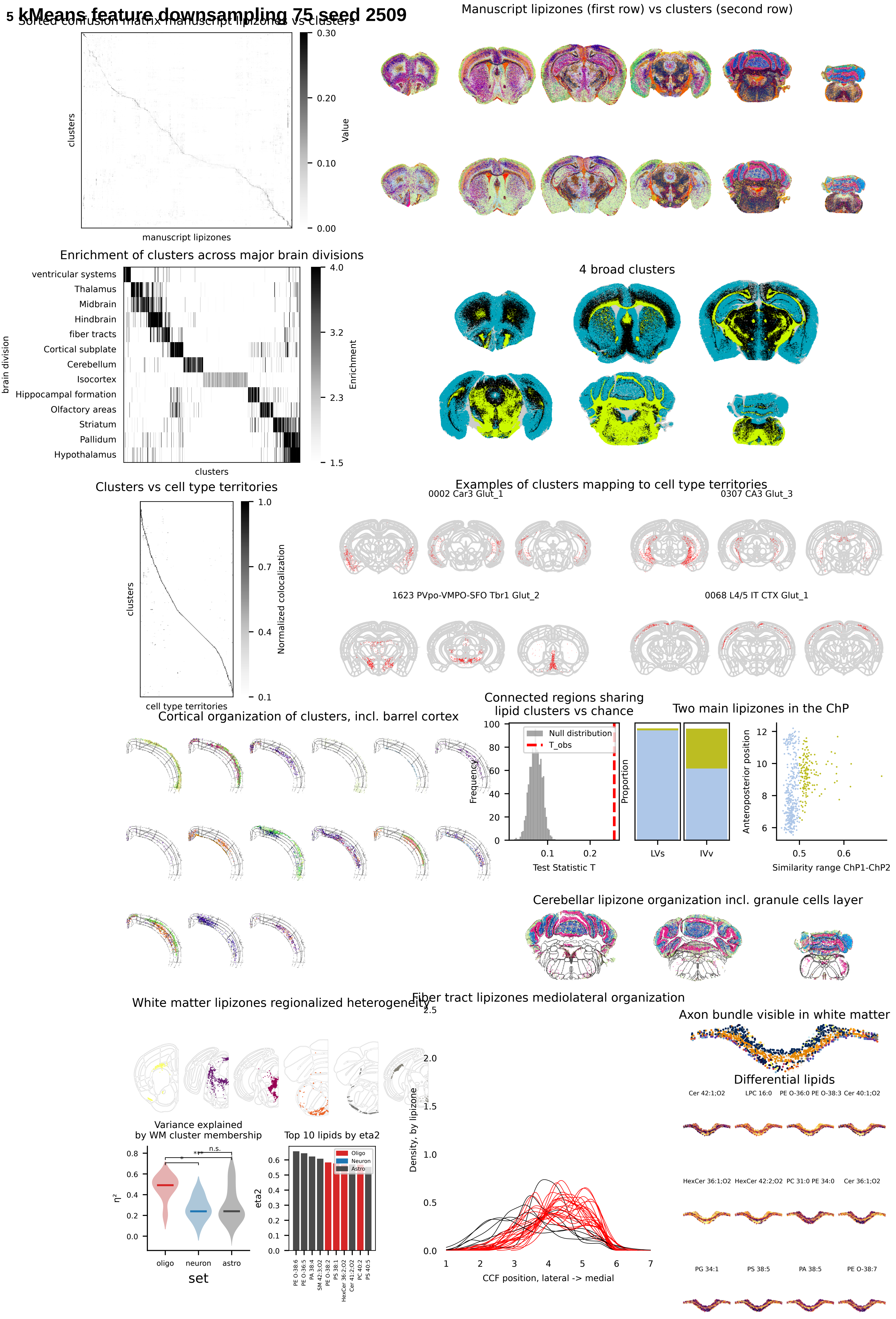

5 kMeans feature downsampling 75 seed 2509
Sorted confusion matrix manuscript lipizones vs clusters
clusters
manuscript lipizones
Value
0.30
0.20
0.10
0.00
Manuscript lipizones (first row) vs clusters (second row)
Enrichment of clusters across major brain divisions
ventricular systems
Thalamus
Midbrain
Hindbrain
fiber tracts
Cortical subplate
Cerebellum
Isocortex
Hippocampal formation
Olfactory areas
Striatum
Pallidum
Hypothalamus
brain division
clusters
Enrichment
4.0
3.2
2.3
1.5
4 broad clusters
Clusters vs cell type territories
clusters
cell type territories
Normalized colocalization
1.0
0.7
0.4
0.1
Examples of clusters mapping to cell type territories
0002 Car3 Glut_1
0307 CA3 Glut_3
1623 PVpo-VMPO-SFO Tbr1 Glut_2
0068 L4/5 IT CTX Glut_1
Cortical organization of clusters, incl. barrel cortex
Connected regions sharing
lipid clusters vs chance
Null distribution
T_obs
Frequency
Test Statistic T
Proportion
LVs
IVv
Two main lipizones in the ChP
Anteroposterior position
Similarity range ChP1-ChP2
Cerebellar lipizone organization incl. granule cells layer
White matter lipizones regionalized heterogeneity
Fiber tract lipizones mediolateral organization
Axon bundle visible in white matter
Differential lipids
Cer 42:1;O2
LPC 16:0
PE O-36:0
PE O-38:3
Cer 40:1;O2
HexCer 36:1;O2
HexCer 42:2;O2
PC 31:0
PE 34:0
Cer 36:1;O2
PG 34:1
PS 38:5
PA 38:5
PE O-38:7
Variance explained
by WM cluster membership
η²
n.s.
***
oligo
neuron
astro
set
Top 10 lipids by eta2
eta2
Oligo
Neuron
Astro
PE O-38:6
PE O-36:5
PA 38:4
SM 42:3;O2
PE O-38:2
PS 38:1
HexCer 36:2;O2
Cer 41:2;O2
PC 40:2
PS 40:5
Density, by lipizone
CCF position, lateral -> medial

6 **kMeans feature downsampling 75 seed 2**

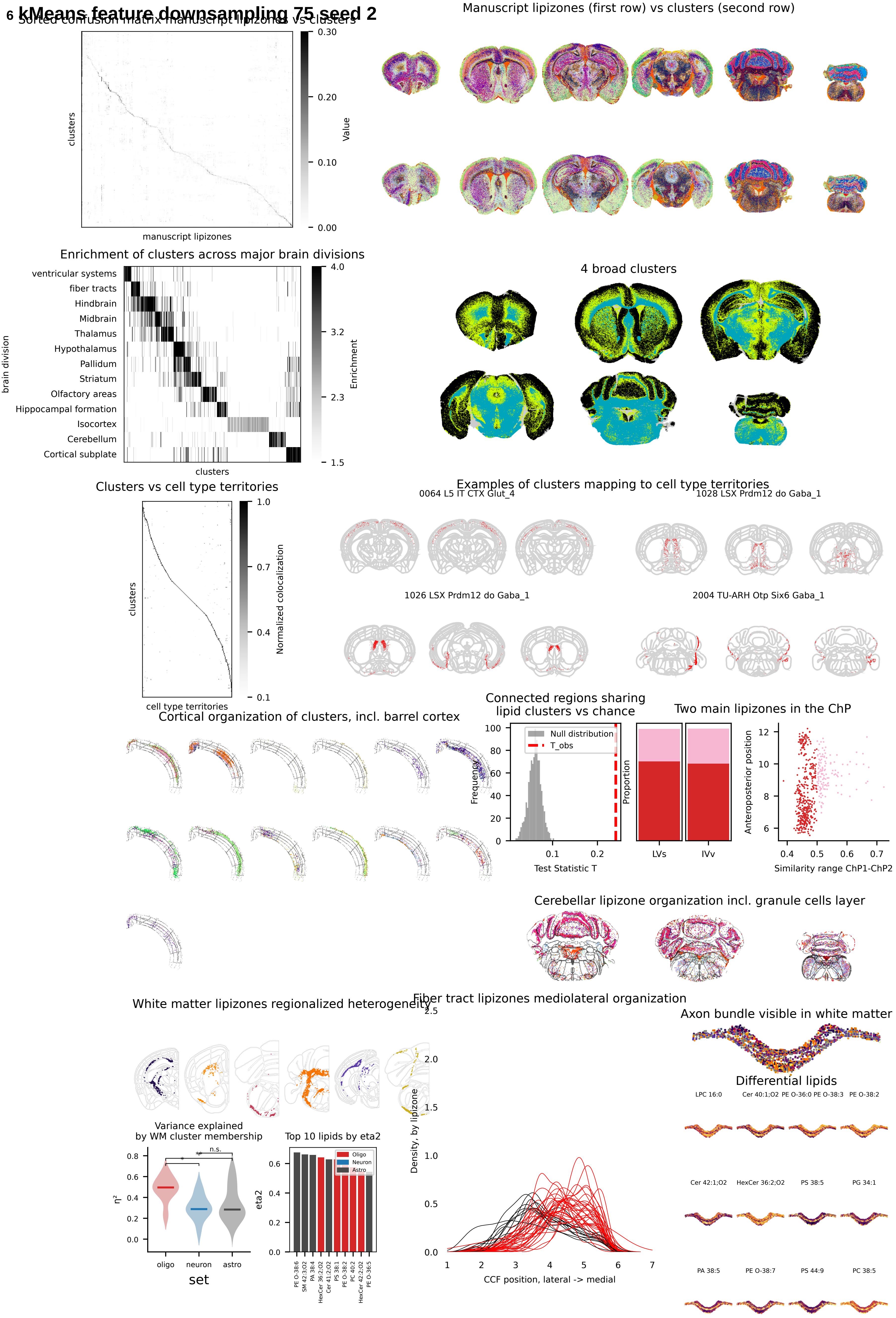

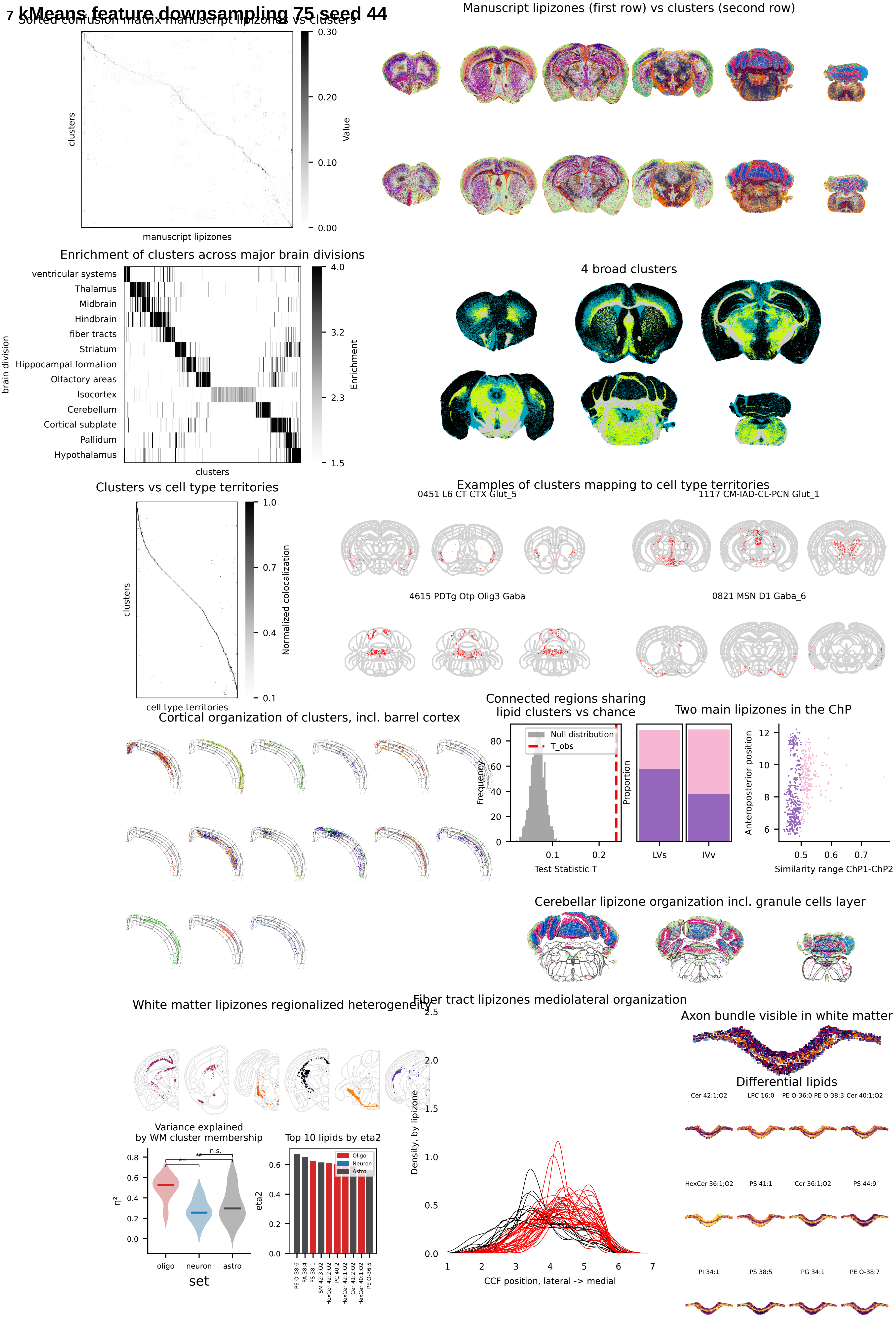
7 kMeans feature downsampling 75 seed 44
Sorted confusion matrix manuscript lipizones vs clusters
clusters
manuscript lipizones
Value
0.30
0.20
0.10
0.00
Manuscript lipizones (first row) vs clusters (second row)
Enrichment of clusters across major brain divisions
ventricular systems
Thalamus
Midbrain
Hindbrain
fiber tracts
Striatum
Hippocampal formation
Olfactory areas
Isocortex
Cerebellum
Cortical subplate
Pallidum
Hypothalamus
brain division
clusters
Enrichment
4.0
3.2
2.3
1.5
4 broad clusters
Clusters vs cell type territories
clusters
cell type territories
Normalized colocalization
1.0
0.7
0.4
0.1
Examples of clusters mapping to cell type territories
0451 L6 CT CTX Glut_5
1117 CM-IAD-CL-PCN Glut_1
4615 PDTg Otp Olig3 Gaba
0821 MSN D1 Gaba_6
Cortical organization of clusters, incl. barrel cortex
Connected regions sharing lipid clusters vs chance
Null distribution
T_obs
Frequency
Test Statistic T
Two main lipizones in the ChP
Proportion
LVs
IVv
Anteroposterior position
Similarity range ChP1-ChP2
Cerebellar lipizone organization incl. granule cells layer
White matter lipizones regionalized heterogeneity
Fiber tract lipizones mediolateral organization
Density, by lipizone
CCF position, lateral -> medial
Axon bundle visible in white matter
Differential lipids
Cer 42:1;O2
LPC 16:0
PE O-36:0
PE O-38:3
Cer 40:1;O2
HexCer 36:1;O2
PS 41:1
Cer 36:1;O2
PS 44:9
PI 34:1
PS 38:5
PG 34:1
PE O-38:7
Variance explained by WM cluster membership
η²
set
oligo
neuron
astro
n.s.
Top 10 lipids by eta2
eta2
Oligo
Neuron
Astro
PE O-38:6
PA 38:4
PS 38:1
SM 42:3;O2
HexCer 42:2;O2
PC 40:2
HexCer 42:1;O2
Cer 41:2;O2
HexCer 40:1;O2
PE O-36:5

8 Leiden feature downsampling 75 seed 23

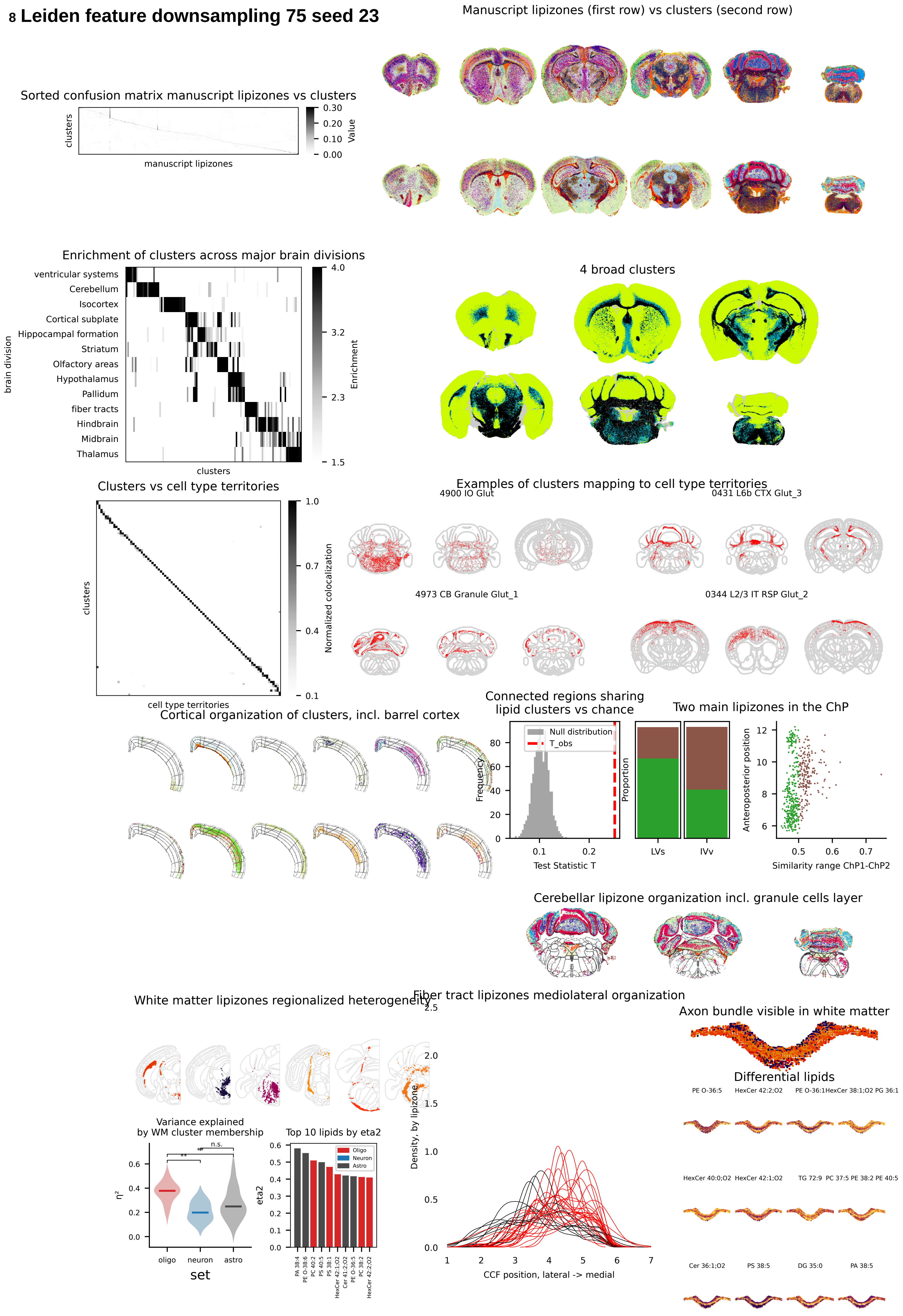

9 Leiden feature downsampling 75 seed 44

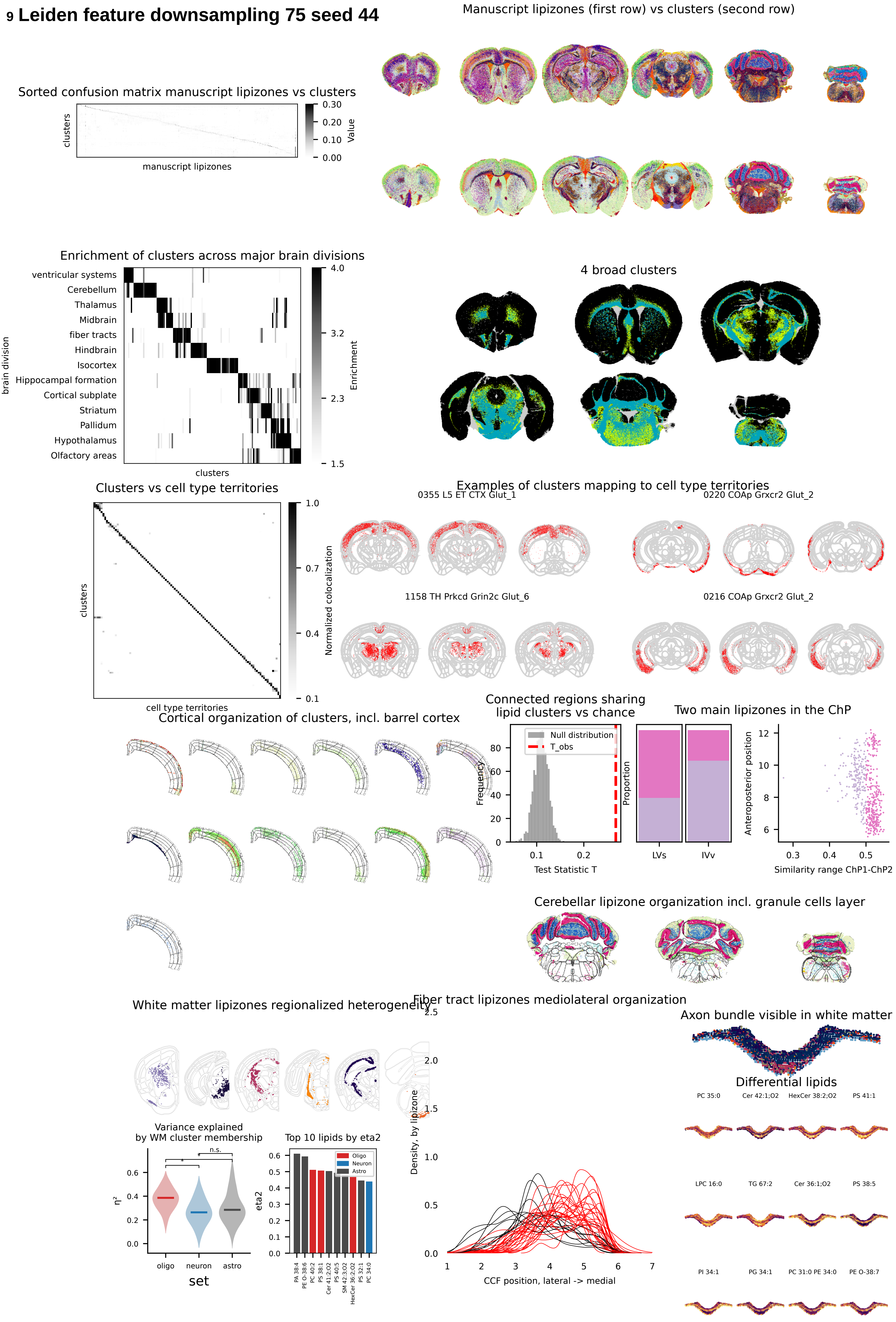

10 Leiden on lipiMap

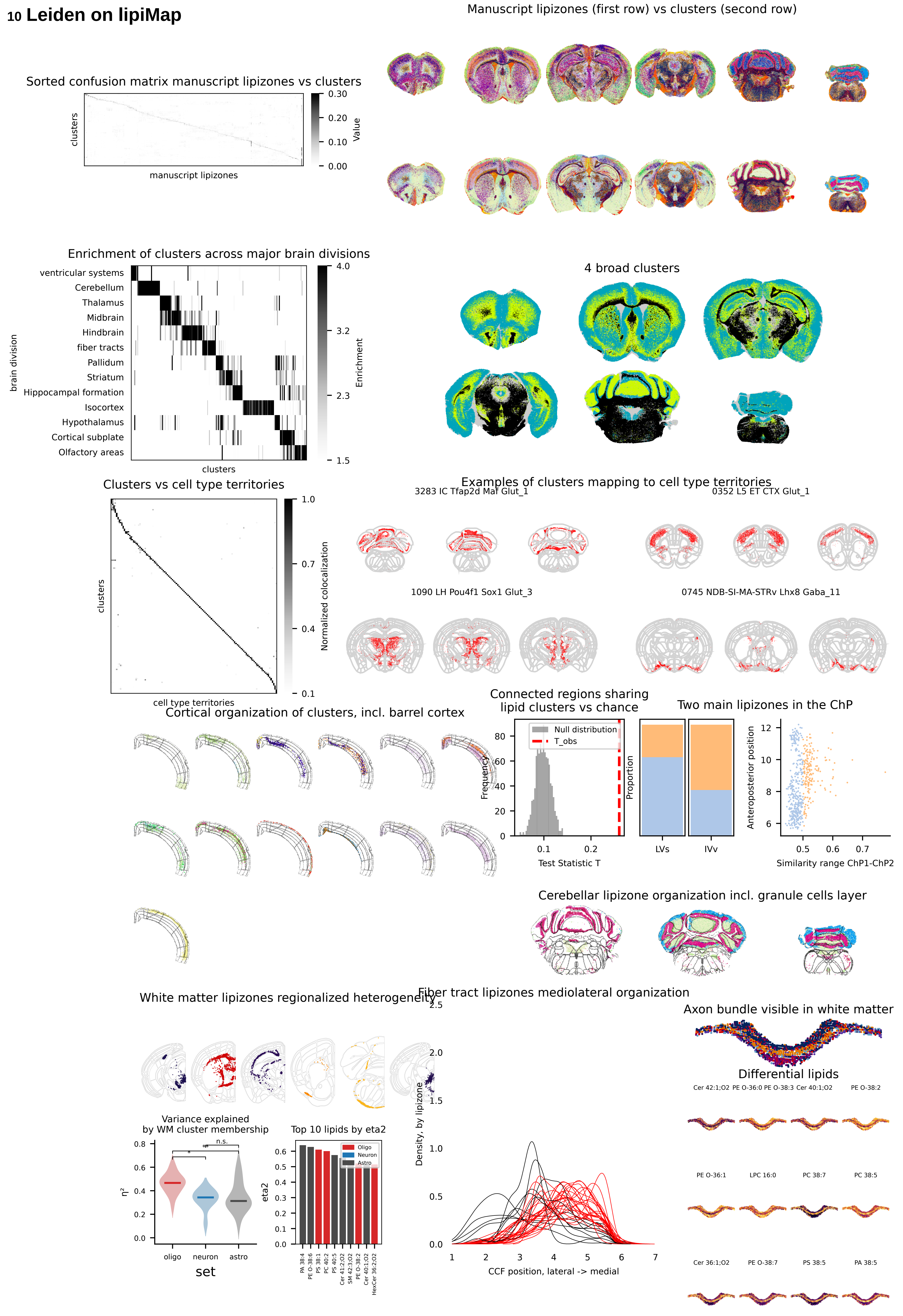

11 Spectral on Harmony

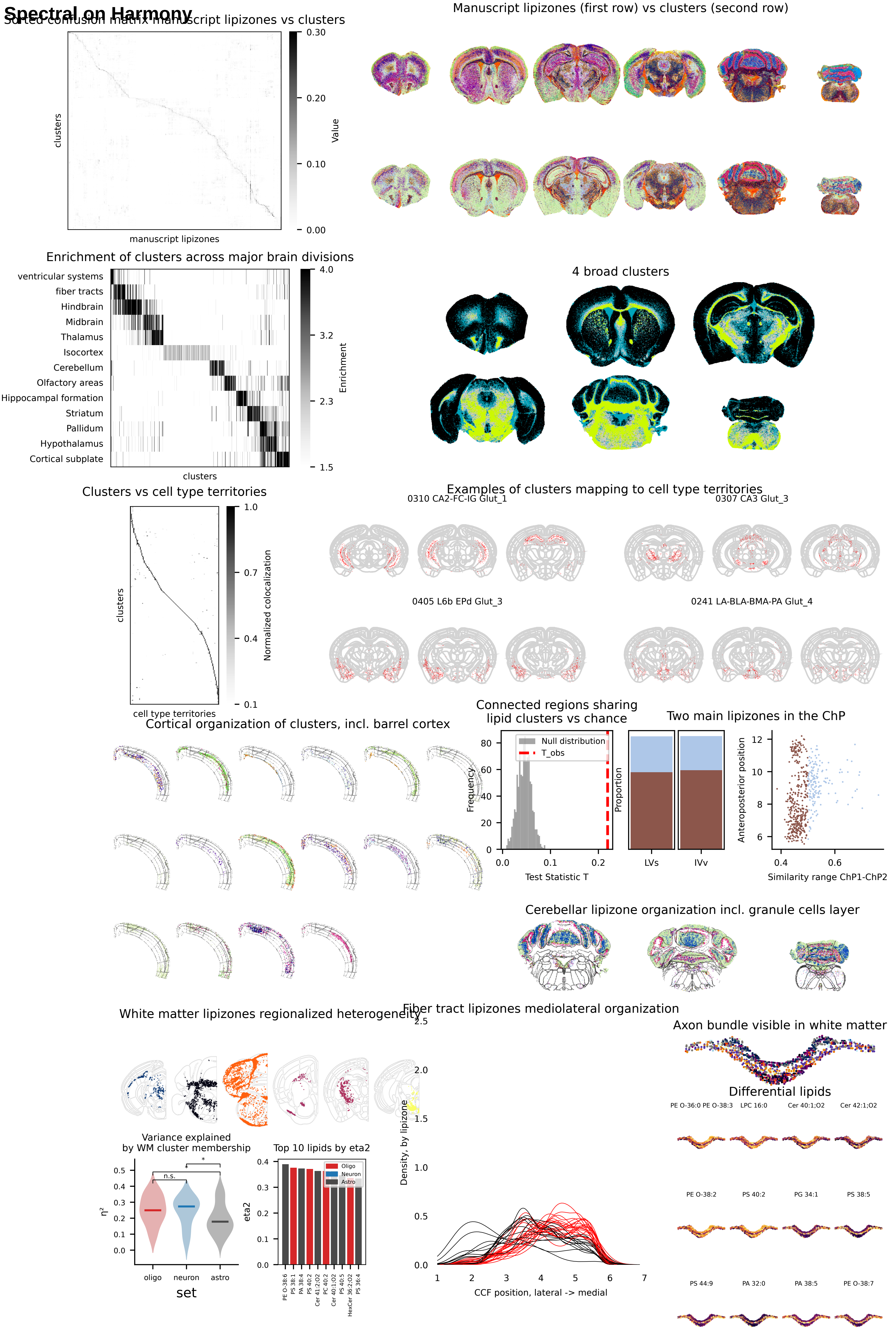

Transferred from splitter trained on second atlas

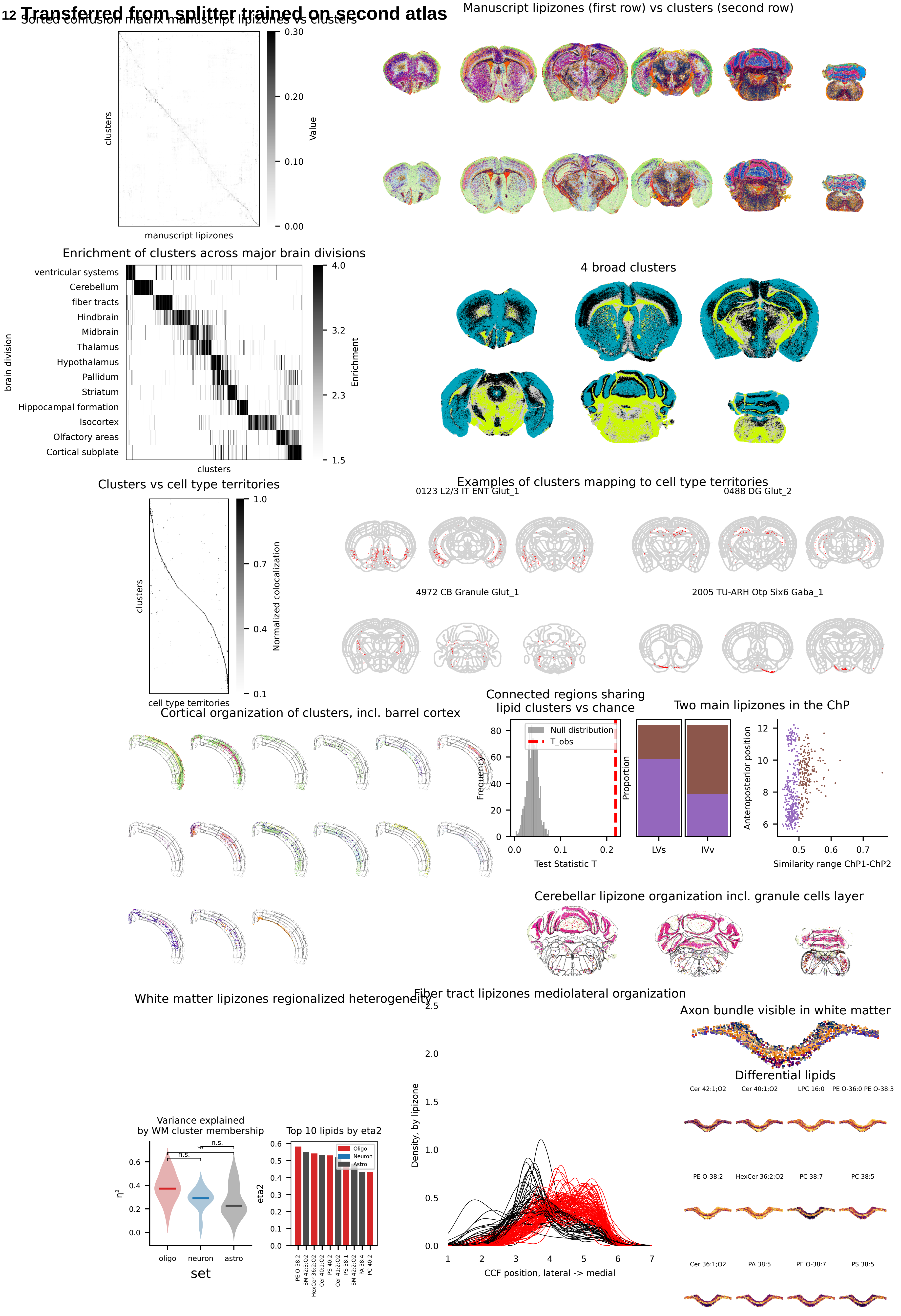

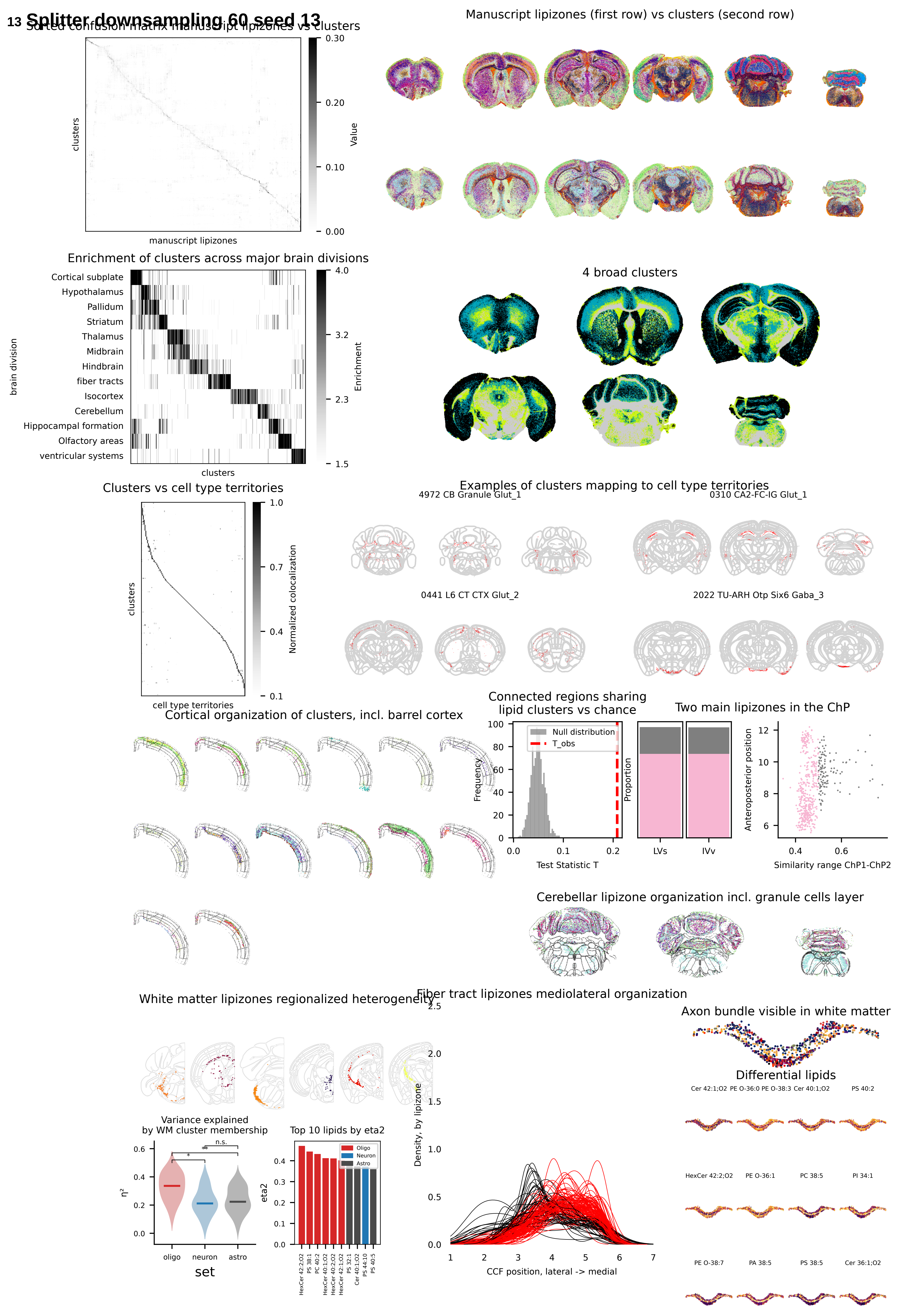
13 Splitter downsampling 60 seed 13
Sorted confusion matrix manuscript lipizones vs clusters
clusters
manuscript lipizones
Value
0.30
0.20
0.10
0.00
Manuscript lipizones (first row) vs clusters (second row)
Enrichment of clusters across major brain divisions
Cortical subplate
Hypothalamus
Pallidum
Striatum
Thalamus
Midbrain
Hindbrain
fiber tracts
Isocortex
Cerebellum
Hippocampal formation
Olfactory areas
ventricular systems
brain division
clusters
Enrichment
4.0
3.2
2.3
1.5
4 broad clusters
Clusters vs cell type territories
clusters
cell type territories
Normalized colocalization
1.0
0.7
0.4
0.1
Examples of clusters mapping to cell type territories
4972 CB Granule Glut_1
0310 CA2-FC-IG Glut_1
0441 L6 CT CTX Glut_2
2022 TU-ARH Otp Six6 Gaba_3
Cortical organization of clusters, incl. barrel cortex
Connected regions sharing lipid clusters vs chance
Null distribution
T_obs
Frequency
Test Statistic T
Two main lipizones in the ChP
Proportion
LVs
IVv
Anteroposterior position
Similarity range ChP1-ChP2
Cerebellar lipizone organization incl. granule cells layer
White matter lipizones regionalized heterogeneity
Fiber tract lipizones mediolateral organization
Density, by lipizone
CCF position, lateral -> medial
Axon bundle visible in white matter
Differential lipids
Cer 42:1;O2
PE O-36:0
PE O-38:3
Cer 40:1;O2
PS 40:2
HexCer 42:2;O2
PE O-36:1
PC 38:5
PI 34:1
PE O-38:7
PA 38:5
PS 38:5
Cer 36:1;O2
Variance explained by WM cluster membership
η²
set
oligo
neuron
astro
n.s.
Top 10 lipids by eta2
eta2
Oligo
Neuron
Astro
HexCer 42:2;O2
PS 38:1
PC 40:2
HexCer 40:1;O2
HexCer 40:2;O2
HexCer 42:1;O2
PS 32:1
Cer 40:1;O2
PS 44:10
PS 40:5

14 **Splitter downsampling 60 seed 52**

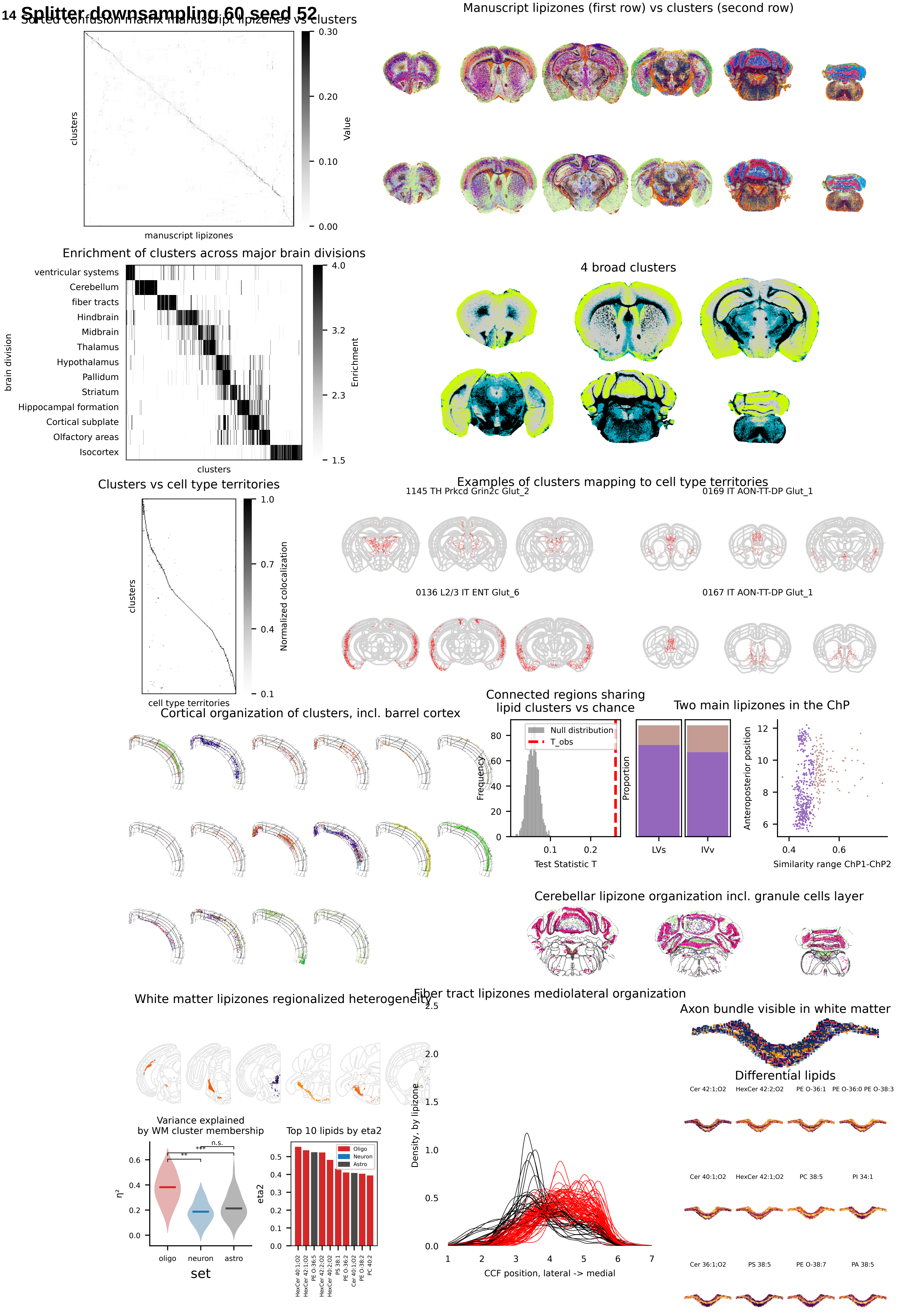

Splitter downsampling 60 seed 73

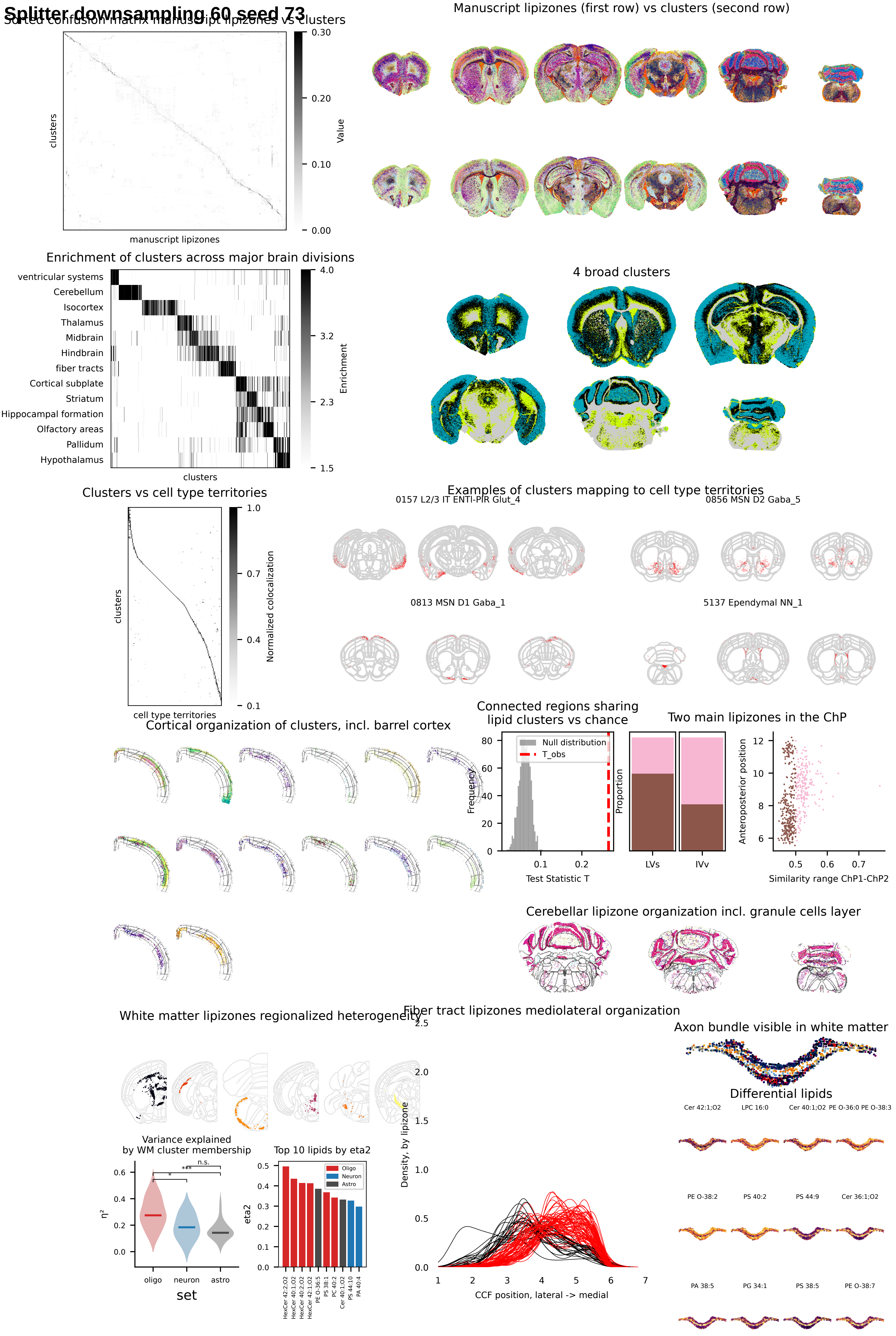

16 Splitter downsampling 75 seed 15

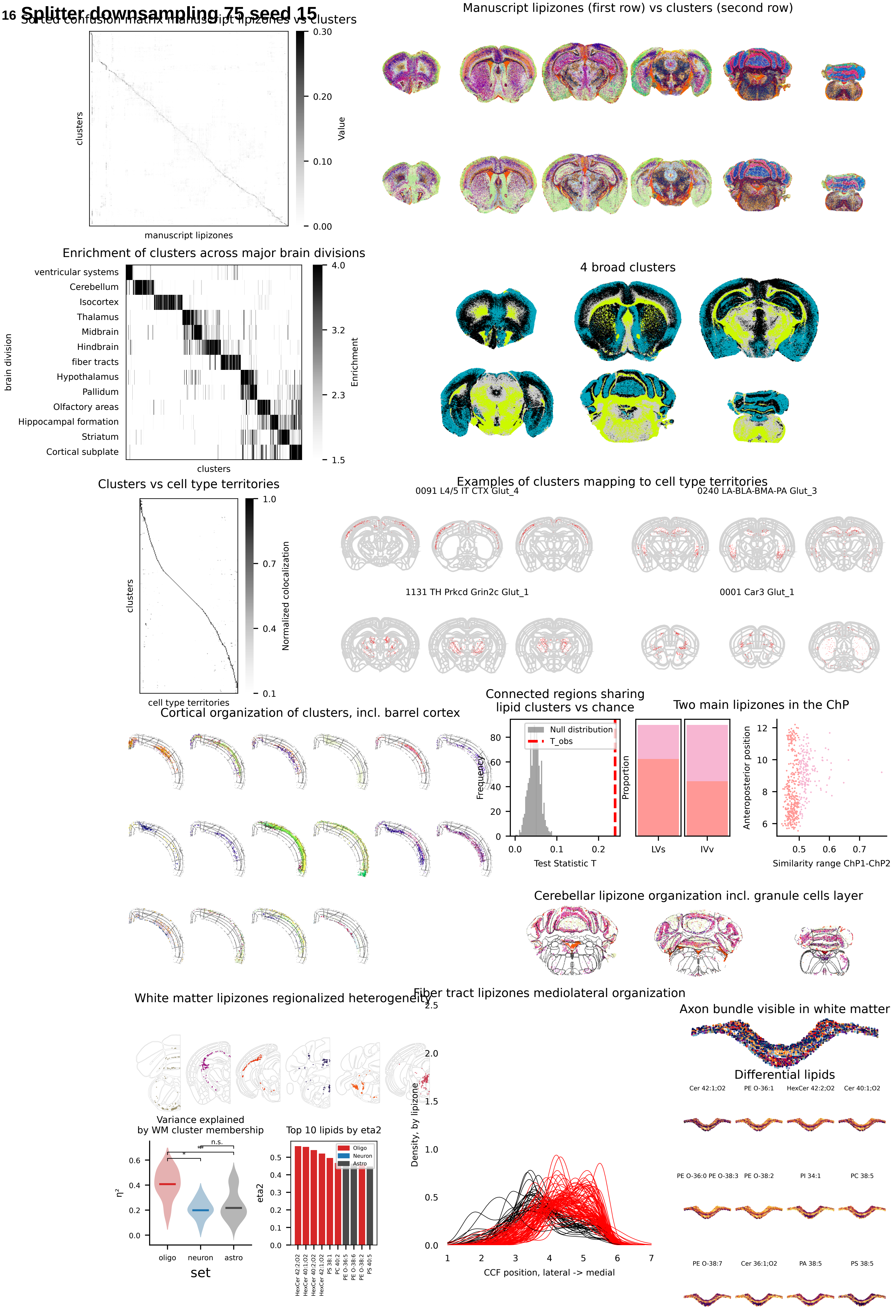

17 Splitter downsampling 75 seed 32

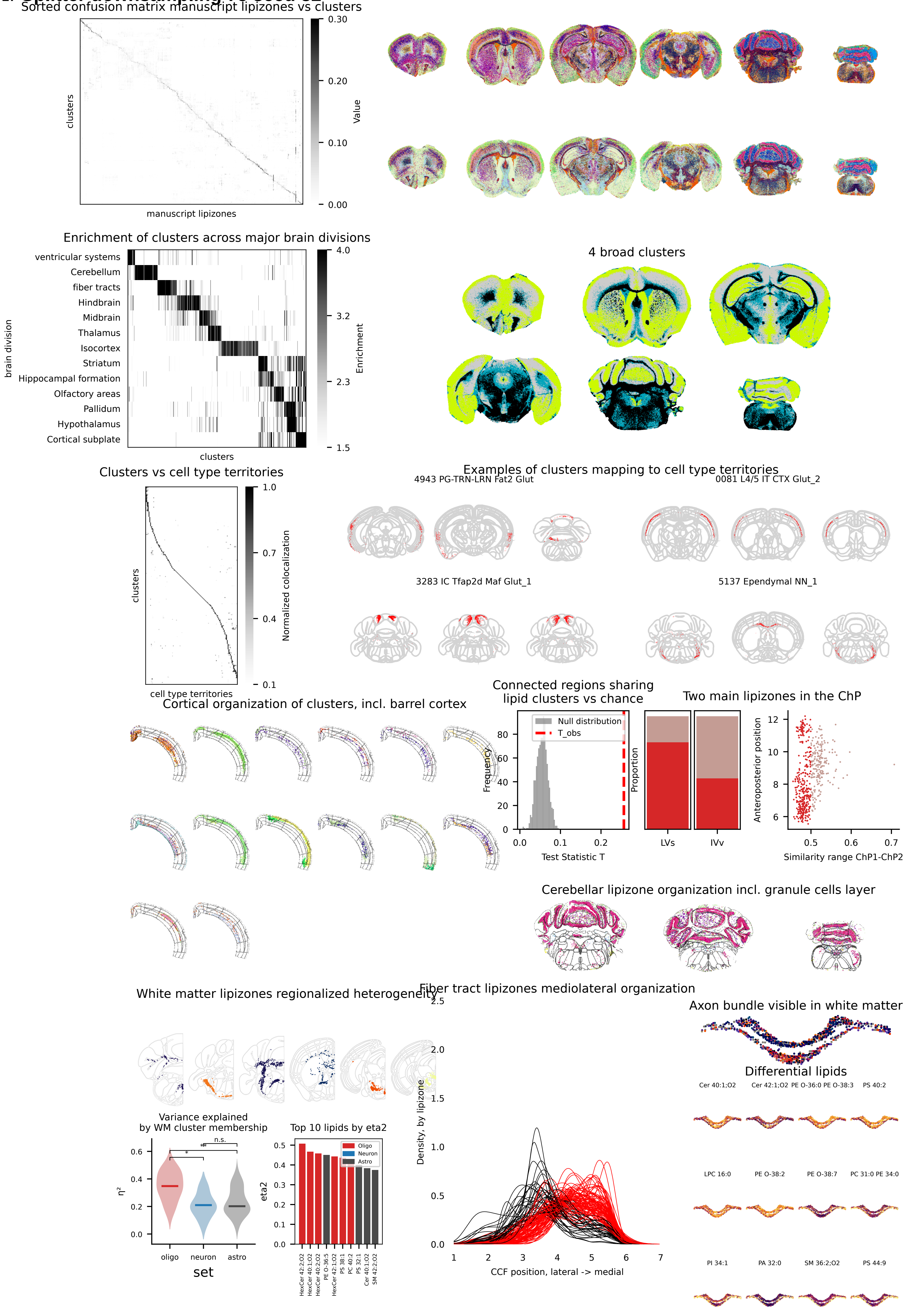

Splitter downsampling 75 seed 42

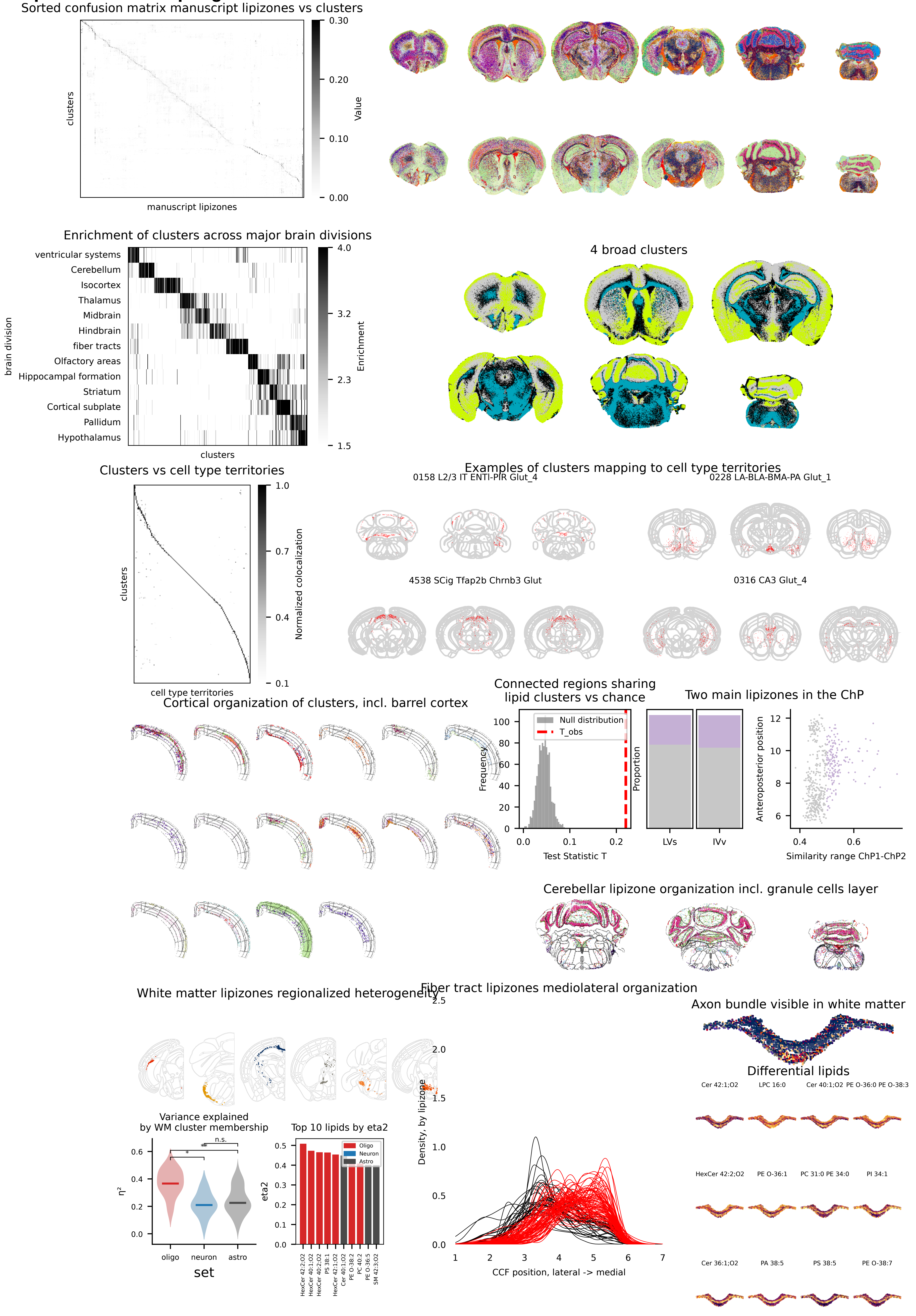

19 Splitter downsampling 75 seed 52

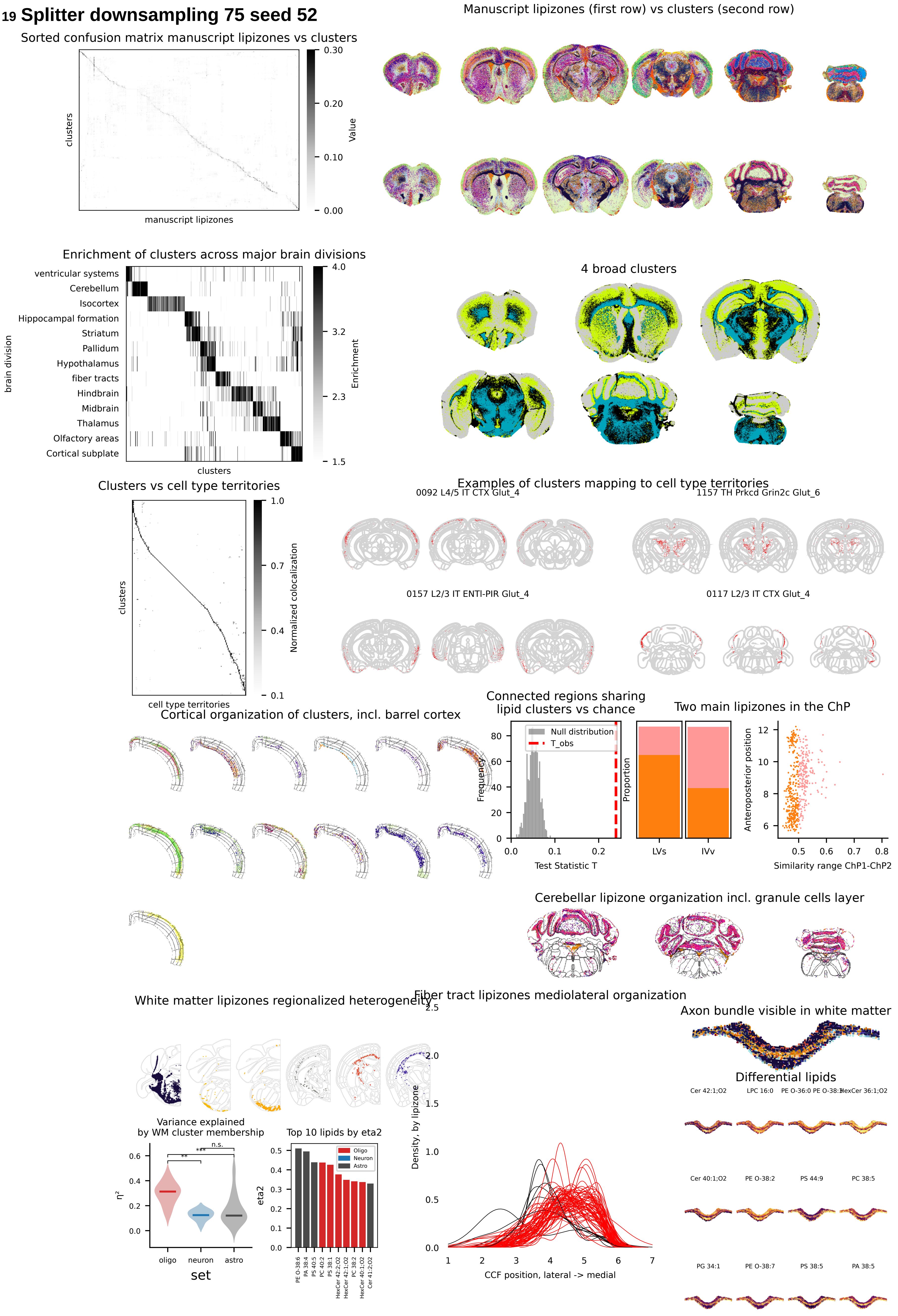

Splitter downsampling 90 seed 66

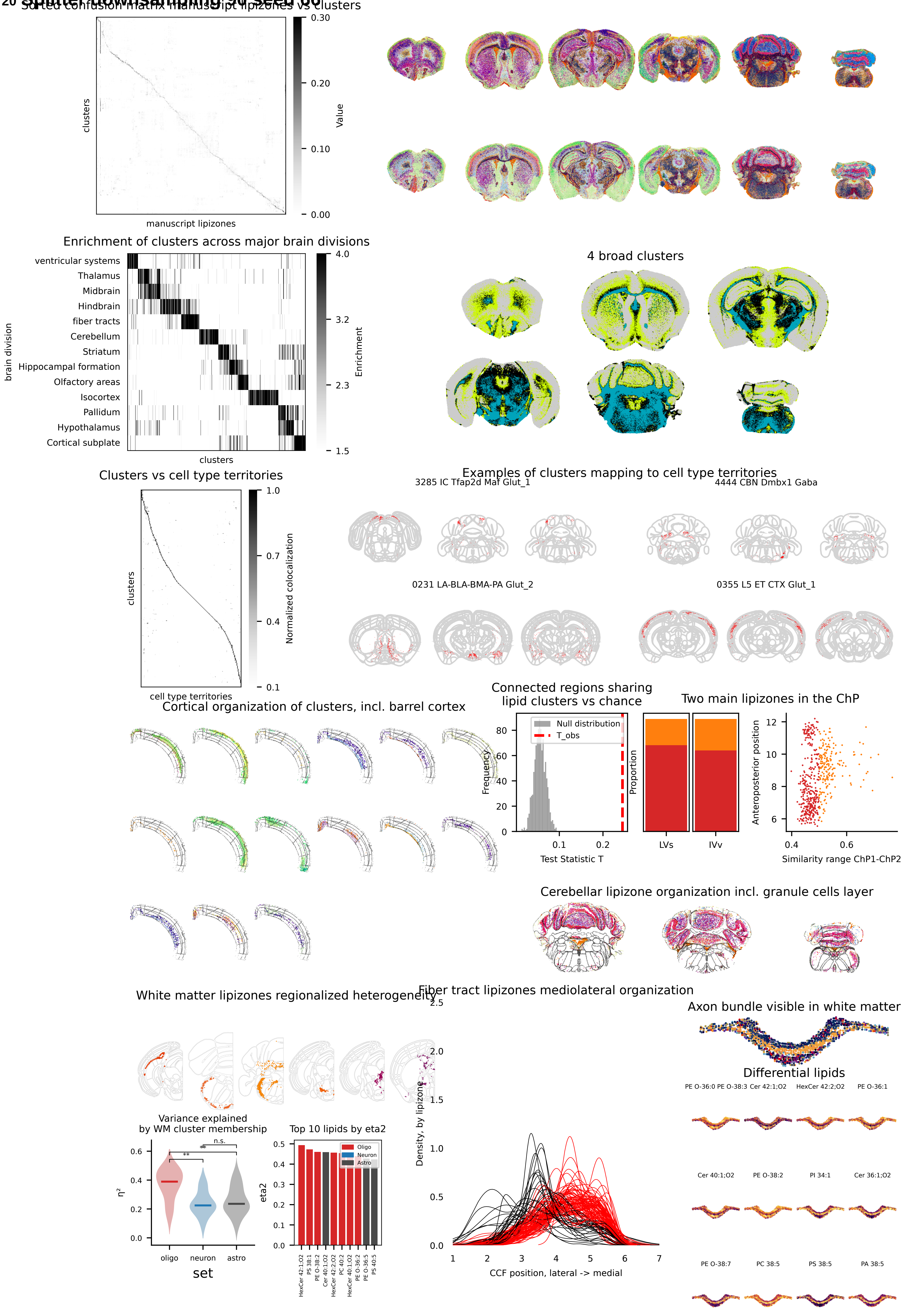

21 Splitter incl. Imputed lipids

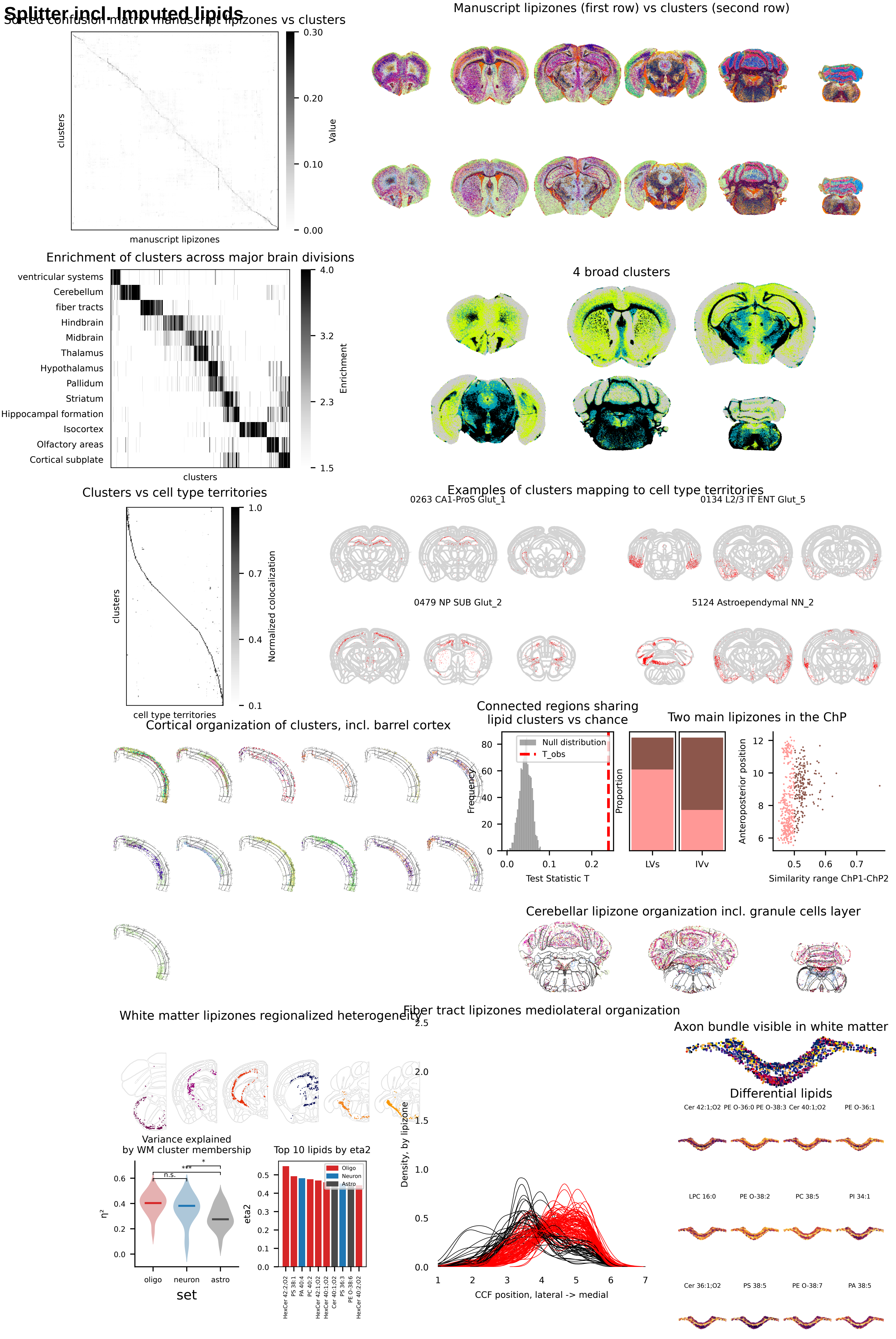

22 Splitter parameter set 1

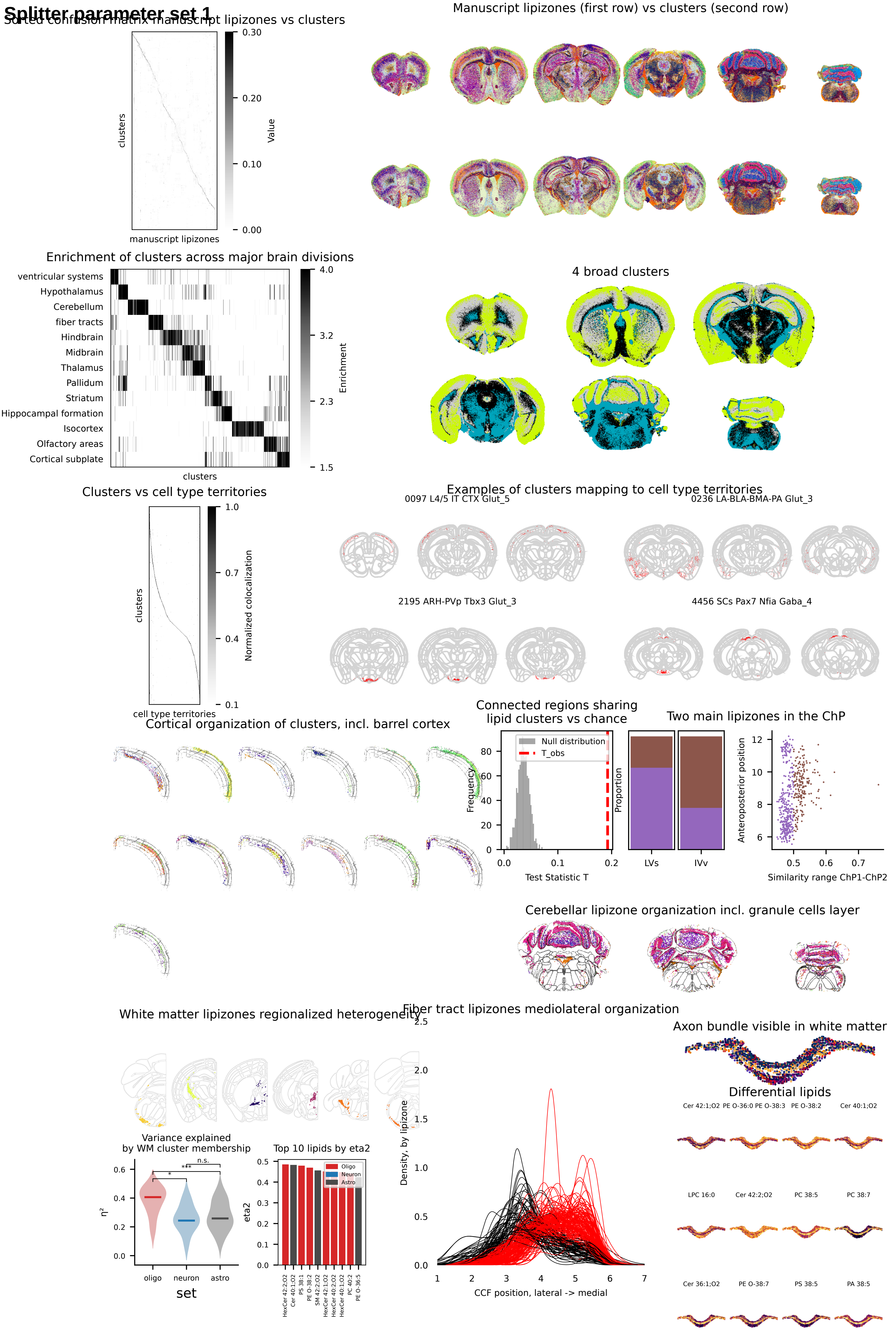

23 Splitter 24 sections

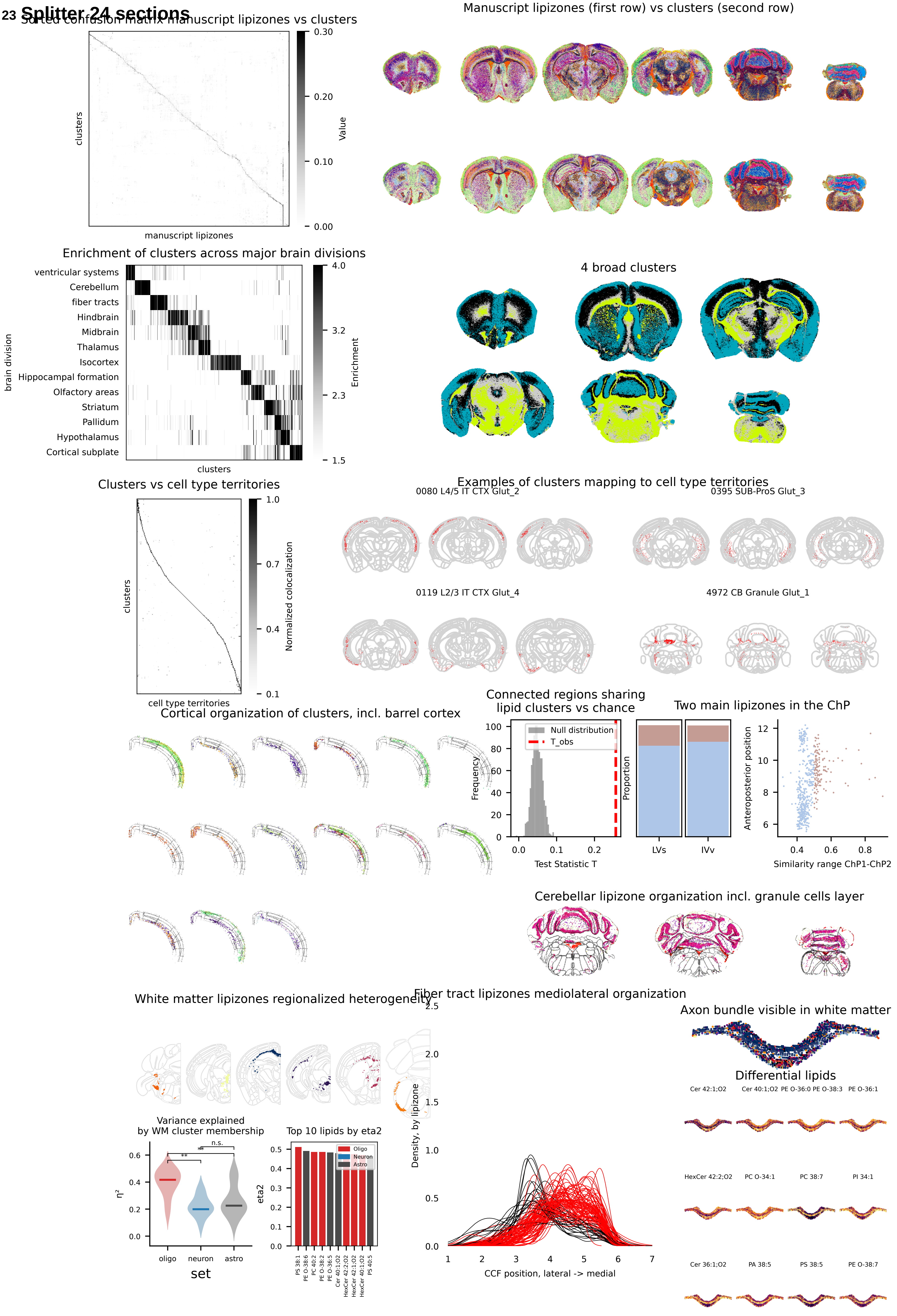

24 Splitter 18 sections

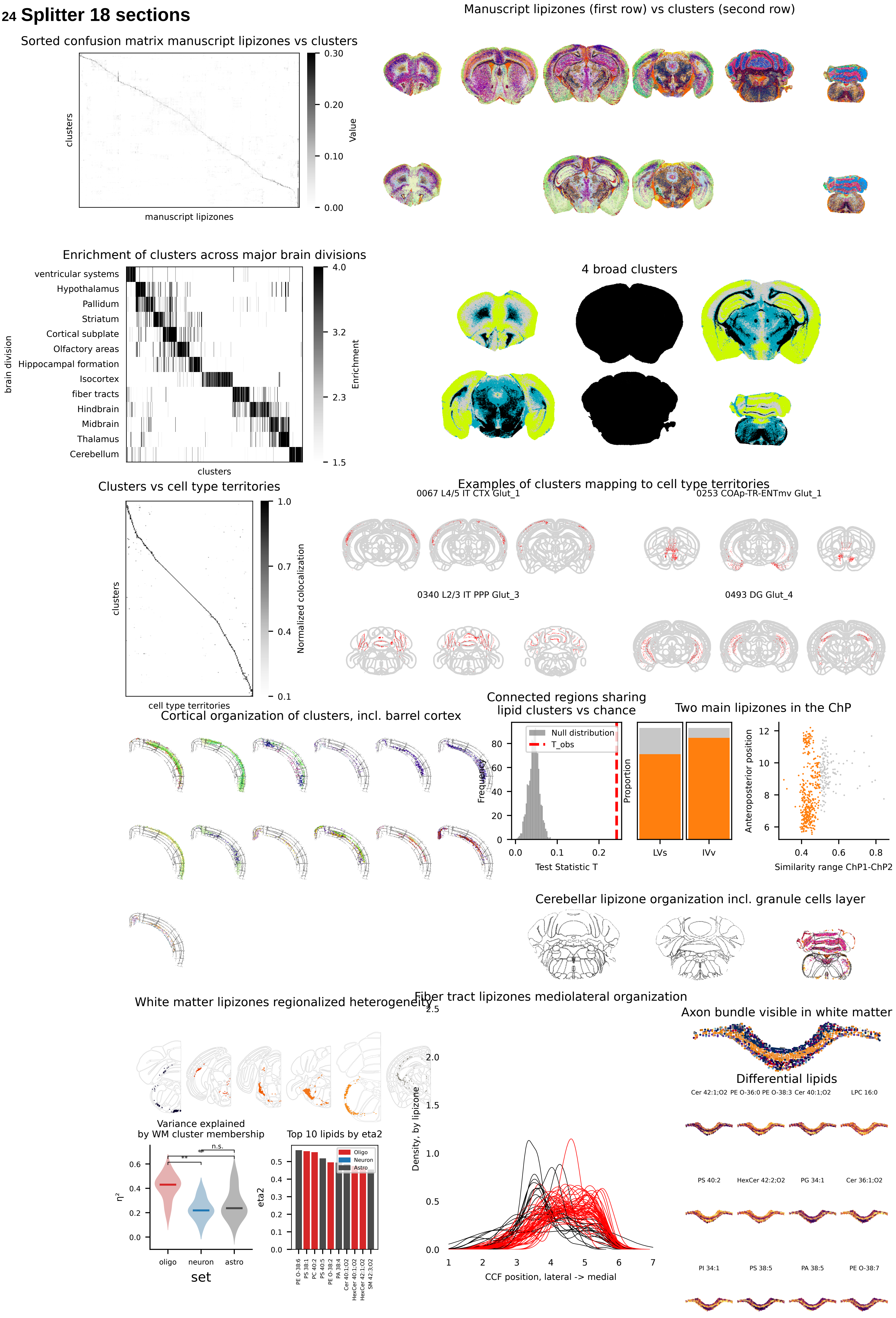

Splitter 12 sections

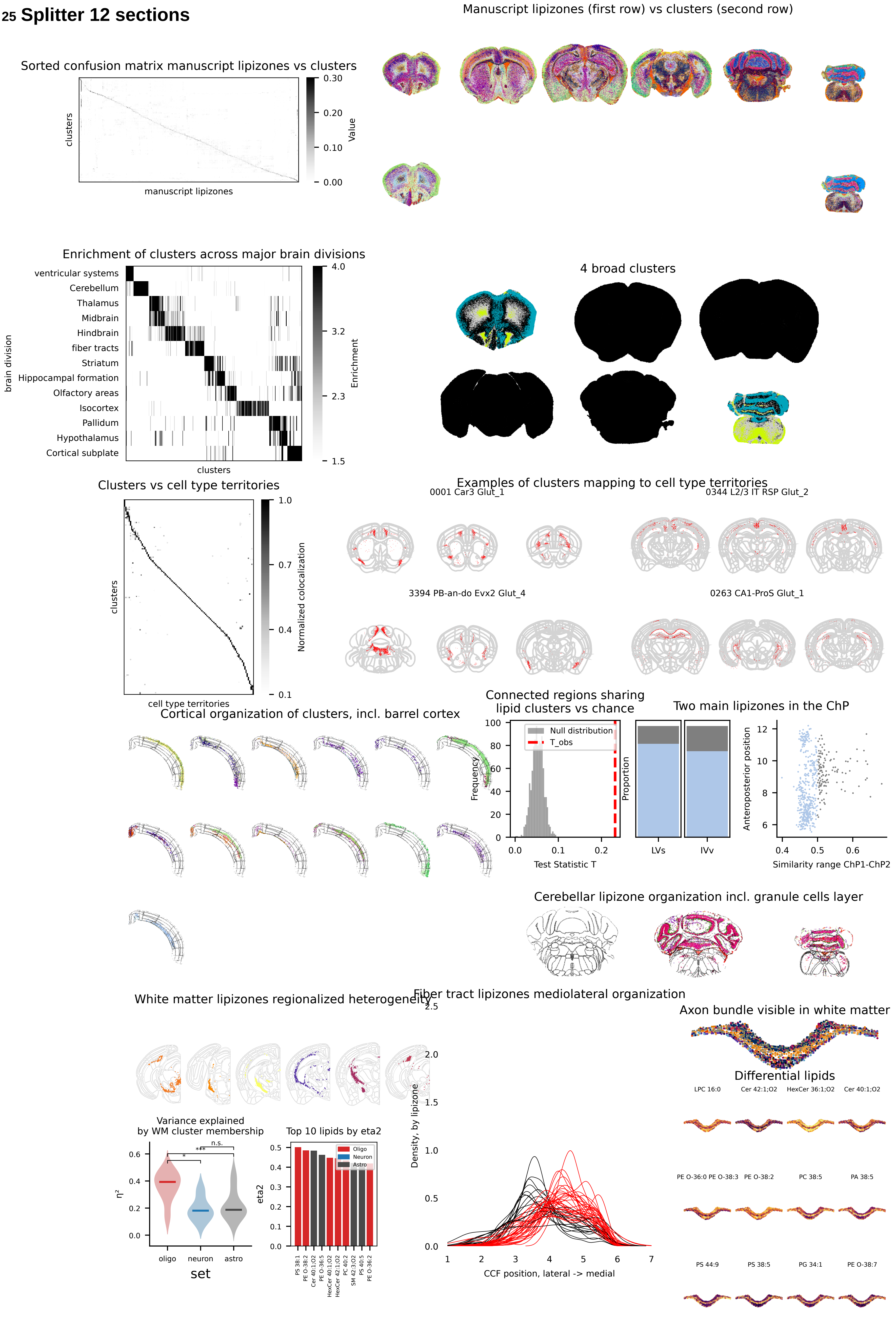

26 Leiden feature downsampling 75 seed 1111

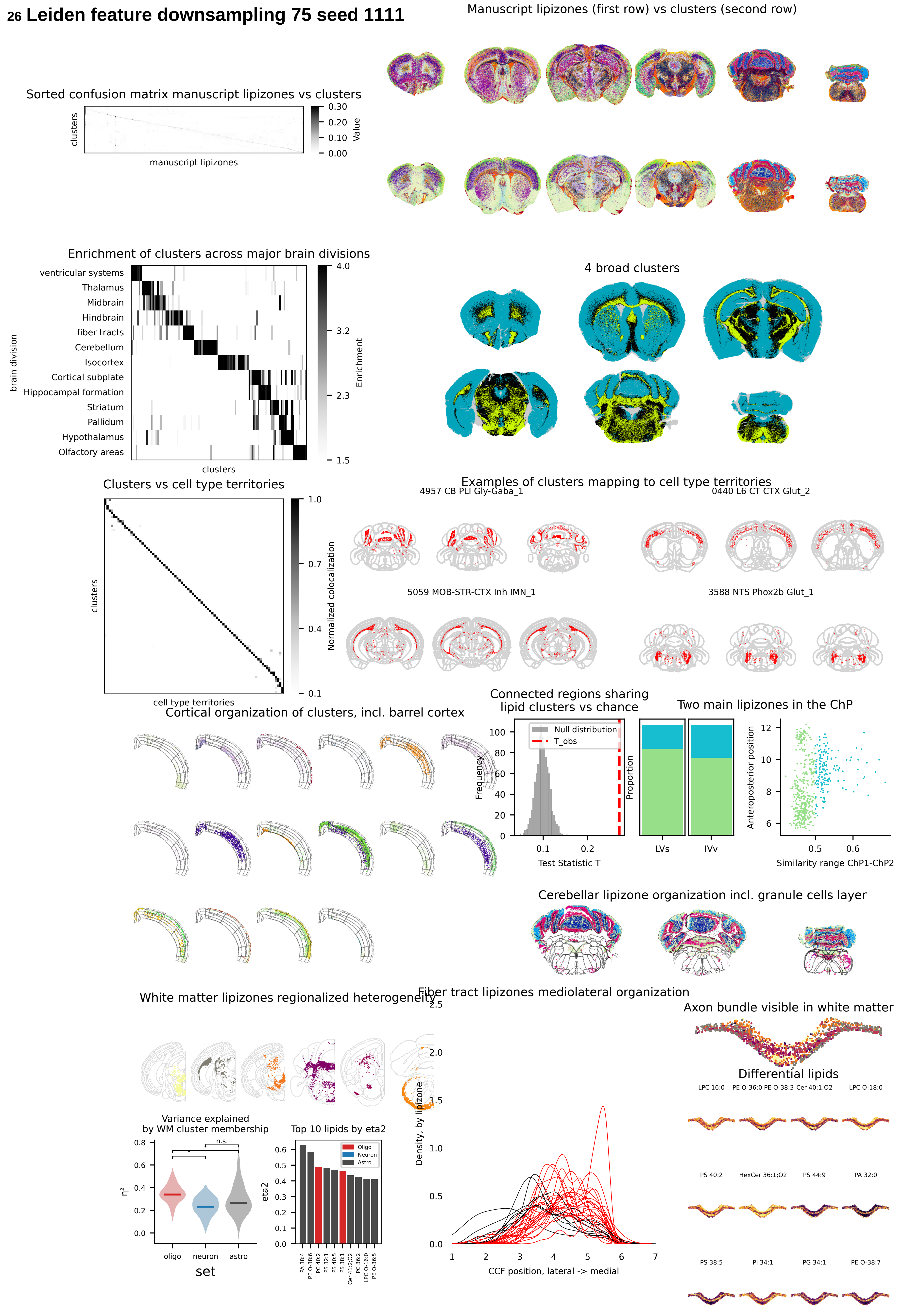

27 kMeans feature downsampling 75 seed 1111

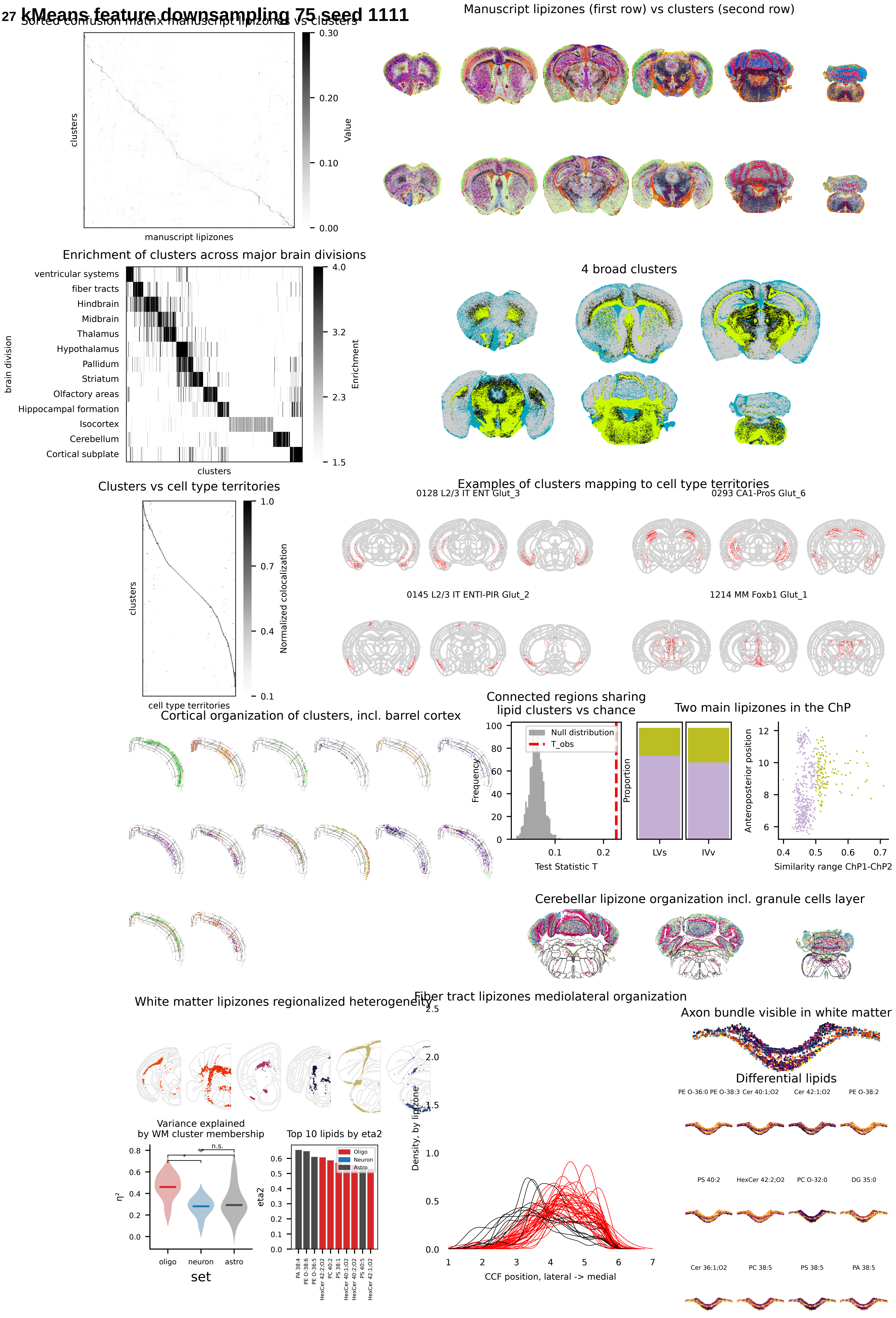

Supplement: Supplementary file 2 [file 41586_2026_11050_MOESM5_ESM.pdf]
