# Supplementary material for: The lipidomic architecture of the mouse brain
Source: Nature. 2026 Sep 23;658(8134):205–17. doi: 10.1038/s41586-026-11050-0 (PMC13626954; doi:10.1038/s41586-026-11050-0)
Supplement: Supplementary file 3 [file 41586_2026_11050_MOESM6_ESM.pdf]

nature portfolio

# Peer Review File

# The lipidomic architecture of the mouse brain

Corresponding Author: Professor Gioele La Manno

Any redactions in this file are there to maintain patient confidentiality, the confidentiality of unpublished data, or to remove third-party material.

**This file contains all reviewer reports in order by version, followed by all author rebuttals in order by version.**

Version 2:

Reviewer comments:

Referee #1

(Remarks to the Author)
This manuscript by Bassini et al present an elegant and detailed map of the lipidomics architecture of the mouse brain, far beyond any previous studies. While the brain has been mapped at great detail to reveal distinct regions, this has not been achieved on the lipidome level at the depth presented here. The study employs MALDI-MSI and advanced machine learning to map the localization of lipids across serial mouse brain sections across several model systems (e.g. age, sex and pregnancy), to demonstrate how localized lipid environments can adapt to different requirements. Not only do lipid microenvironment (called lipozones) match to cell types and previously identified genetic zones, but new zones are also identified based on lipid composition not previously identified were ground, highlighting the critical, and as yet poorly understood, role of lipid metabolism in defining brain architecture and connectivity. This includes novel information such as oligodendrocyte heterogeneity in the white matter and biochemical compartmentalization within the choroid plexus. The work is highly novel, data analysis is sound and the findings hold significant value across the biological sciences, opening up new means to understand brain metabolism (and dysregulation) and connectivity. The work reveals some very exciting new insights about brain lipid biochemistry. The manuscript has a broad readership appeal to many fields of science and is very timely in regard to interest in brain lipid biochemistry within neurosciences. Based on the findings and their broader implications, the quality of the work, and the excellent presentation of data I am supportive of publication in Nature. I do have several questions and comments that the authors should consider.

The introduction should acknowledge that MALDI-MSI has already been employed across many studies to map lipids in brain, both in 2D and 3D. This does not detract from the novelty of this work, but should be acknowledged with relevant citations.

The authors have done a good job and annotated the lipids based on the MSI data – including comparisons with LC-MS/MS. This is probably as good as is possible. This leaves overall high confidence in many lipid annotations. As key conclusions are based on patterns of lipid species throughout the lipozones, some annotations raise questions.

1. The MSI is performed in positive ion mode. The authors present a variety of lipids detected that are not typically detected in positive mode MALDI-MSI, such as PG, PI, and PA. While these lipids are likely present in brain, it is not clear if they are truly in the data or just happen to overlap within the ppm tolerance with an unrelated signal. The studies used to validate the assignments both used negative ion mode to detect these lipids. Do the authors have MS/MS confirmation of these. If not I suggest the authors add caveat statement round the detection of lipids that are not typically detected in positive mode MALDI-MSI so non-MS expert can have the knowledge to interpret the data and understand limitations. One way to check is whether the acyl chain distributions match those revealed by LC-MS where presumable lipids elute on a class basis.

2. Moreover for PA lipids it is more likely these arise from loss of NC3H9 from PC lipids which happens to a reasonable extent in MALDI and leads to a lipid isomeric with a PA. This can be seen when viewing the spectra on METASPACE. Eg. The PC34:1+K ion at 798.54 fragments to the 739.46 which is incorrectly assigned as a PA. Is there MS/MS confirmation that these are PAs? In Supplementary Fig. 1 lipid class compositional breakdown is shown as pie charts for LC-MSMS/MS and MALDI-MSI. There is a rather sizeable black wedge in the MALDI data set that corresponds to PA, while the LCMS does not display this, further suggesting over representation of Pas in the MALDI data. How does the PA profile by LSMS compare to that revealed by MALDI annotations? Al least limitations of PA detection should be acknowledged and

discussions around PA lipids be amended,

Throughout the manuscript sphingolipids do not conform with standardized LIPIDMAPS Consortium nomenclature, e.g., SM 34:1 should be SM 34:1;1 etc. For MALDI the inclusion of the ";2" OH number is particularly important for ceramides as cations can arise as either/both [M+H]+ and [M-H2O+H]+, which can subsequently lead to species misidentifications. In particular, the reviewer draws note to the in-text sphingolipid annotations of Page 10, column 2 where both types of nomenclature are used for HexCer's. The reviewer suggests that the all sphingolipid shorthand nomenclature be standardized throughout and always display the OH number (e.g., either XX 42:2;2 or XX 42:2;O2).

Figure 3A- can the authors comment on how this correlation is calculated. Is the MSI data based on normalized intensities

I am very happy to see this study provides strong evidence that "lipid-based partitions are driven by combinatorial patterns rather than single-lipid dominance.". I think this demonstration and call out is very significant for the field.

Figure 5 and text – when discussing ventrical markers. Is it possible some this arises from residual blood? Were the mice perfused?

For the comparison of sex differences, how many replicates were analyzed?

The pregnancy results are fascinating, especially when the authors begin to speculate how this could relate to neuropathophysiology. Interestingly, there is a paper that found a negative correlation between pregnancy and AD – this could be a good addition to your discussion. https://doi.org/10.1177/1533317518786447

Data availability – The authors have done a thorough job in making data available. The website and interactive visualizations are great, However there are some gaps. It is stated that all raw data is on metaspace. However when clicking on the link there are only a handful of individual sections. Further METASPACE is already processed data as it is centroided and converted to imzML. For such a paper, providing access to the underlying raw data would be helpful to the community. Can the authors also provide access to .raw files from the orbitrap.

Something to note about the web-application, is that sometimes when selecting a new rostro-caudal axis section it doesn't always change the display to that section depth – instead displaying the previously selected section depth. Moving away from and back to appears to fix it, but it may be worthwhile checking to see if this can be resolved. I do note that there is a message about refreshing the page, but this doesn't always work.

(Remarks on code availability)

Referee #2

(Remarks to the Author)
This manuscript describes a large-scale spatial lipidomic profiling effort in the mouse brain using high-resolution MALDI-MSI that delivers 5 micron spatial resolution at 25 micron intervals. The dataset spans 11adult brains that includes 2 densely sampled males (32 coronal sections each, corresponding to about a third the frequency of sampling of current neuroanatomical and spatial transcriptomics atlases), and six sections each from 3 additional males, 3 females, and 3 pregnant females. The main finding of the paper is the division of the brain into hundreds of discrete lipid-defined zones ("lipizones")
- Especially for all main findings regarding lipizones—e.g. the barrel cortex-specific lipizones, and "inside vs outside" lipizones, it would be reassuring to do some additional pressure testing of clustering reproducibility across brains. This would include transfer accuracy reporting across all brains (not just brain 2) and an assessment of cluster stability using resampling and feature downsampling.
- One somewhat interesting and admittedly confusing aspect of the lipizones is how they distribute across regions. Compared with the single-cell and spatial atlases, there are far more lipizones in larger regions (e.g. cortex and cerebellum) and comparatively few
- The claim that some lipizones correspond to patterns of connectivity or functionally related cells could be better supported. For every pairing of two cell types—like layer 6 paired with thalamic cells—there are plenty of other canonically connected cells (like thalamic cells to layer 4 cells, for example!) that do not seem to be paired. The main support is a permutation test controlling for inter-regional distance that shows connected regions share lipizones more often than expected (this is just a single suggest correlation). It would be helpful to also report the absolute differences in sharing probability, rather than just a p-value for this analysis. The test is also binarized between "high" and "low" connectivity; it would be helpful to motivate the selection of this binarization by examining the distribution and, if appropriate, showing robustness to alternative thresholds. Lastly on this topic, given that only some connected pairs show lipizone sharing, it might be helpful to do a specificity analysis—for example, in cortex, where inputs and outputs are well established, the authors could examine whether specific

pairings (e.g. corticothalamic versus corticocortical or corticostriatal) drive clustering behavior.
- I found the pregnancy figure to be almost completely impenetrable, beyond the assertion that "pregnancy changes the lipidome." Many panels convey little information—like panel b—or they convey information that is not supporting the key assertions that are returned to in the discussion, namely that the outer cortex and white matter are most affect. If the authors want to argue for these ideas specifically, they need clear, simple analyses that show that these regions are more perturbed than others. The sample size is also precariously low for these assertions, and incudling an individual pregnant replicate into the PCA analysis in fig 6e would really help bolster the claim that pregnancy is a huge contributor to variance, rather than batch effect (which is mentioned as being important to control for in these analyses though I am not entirely clear where or how these batch effects arise). It would also be helpful to independently validate a few of the strongest results orthogonally, such as showing white matter has higher HexCer 42:2 by doing microdissection and bulk lipipomics.

Smaller issues:

- The dataset is mentioned to be 109 sections, but Supp Fig 1b totals 118 sections.
- Regarding the validation staining in figure 5, the citation 61 is to cholera toxin, which doesn't bind sphingomyelins.
- I am not sure that the assertion in the text about Supp Fig 7g—that these finer-grained Allen anatomical regions nicely overlap liposomes—is supported by the figure itself, which is a very hard-to-read, dense confusion matrix. For characterization of the anatomical correlation with the lipizones, it might be helpful to generate a cumulative distribution plot, showing the number of unique anatomical regions (at a given resolution level) needed to reach some reasonable percentage of the total lipizone area (say, 90% or 95%).

(Remarks on code availability)

Referee #3

(Remarks to the Author)
The spatial organization of lipids has been underexplored within in intact tissue. This study presents a high-resolution lipid brain atlas of the adult mouse brain, created using MALDI-MSI and computational methods, mapping lipid composition across sexes and during pregnancy. The authors identified over 500 distinct biochemical regions, termed "lipizones," which align with anatomical structures, cell types, and neural connectivity. Lipizones reveal a fine-grained biochemical architecture that correlates with functional brain anatomy, including both cell bodies and distant axon terminals, something not captured by spatial transcriptomics. Key discoveries include a new axis of oligodendrocyte heterogeneity in white matter and distinct biochemical zonation in the choroid plexus and ventricular walls. Furthermore, the lipidomic architecture demonstrates adaptability during pregnancy, the white matter becomes metabolically activated and the outer cortex undergoes structural reorganization. Overall, this atlas establishes a comprehensive spatially resolved lipid map of the mouse brain.

Major criticism:
There is a degree of overinterpretation and overselling of the data. The authors claim to have identified "lipizones" as fundamental units of lipid composition. However, this appears to be an artificial classification largely driven by the methodology employed. A comparable categorization could likely be derived using other omics data, suggesting the approach may not uniquely reveal such structures. Furthermore, the authors imply that they have achieved single-cell or even subcellular spatial resolution (e.g., in the Introduction), but the method is constrained by the 5 µm spot size of MALDI-MSI, which does not support this level of detail.

Key analyses are also lacking. While the authors describe their spatial resolution as "quasi-cellular," they do not provide lipid composition profiles for distinct brain cell types. Such data are essential to substantiate their claims and should be included, ideally alongside a comparison to lipidomics from cultured cells.

Thus, overall this study represents the first comprehensive spatially resolved lipid map of the mouse brain. However, biological insights provided are limited. Moreover, it is unclear whether this analysis will serve as a valuable resource without identifying the lipid compositions of the various cell types. The concept of "lipizones" appears artificial and may prove difficult to apply in future studies.

(Remarks on code availability)

Referee #4

(Remarks to the Author)
I co-reviewed this manuscript with one of the reviewers who provided the listed reports.

(Remarks on code availability)

Version 3:

Reviewer comments:

Referee #1

(Remarks to the Author)
I would like to commend the authors on providing an extremely thorough and detailed revision. I believe this addresses the reviewer comments for all reviewers, and other than point below, I can recommend publication.

The confirmation of PI and PG by comparing to negative mode data is not necessarily supporting their detection in positive ion mode, as they ionize much better in negative mode. This is evident from Figure 2d where distributions of thinks like PI 38:7, PI 40:7 and PG 40:4 are different across positive and negative ion mode. The authors do acknowledge these are likely not detected in positive ion mode, but I feel this should be stronger to really make clear these identifications are likely something else. The best way to confirm would be with on-tissue MS/MS in positive ion mode where class specific headgroup losses should be observed. Ideally this should be done for a at least 1 or 2 representative PI and PG lipids to support their presence in the data used to define lipozones. In the absence of this a stronger caveat statement would be good

The new data to suggest PC fragments lead to the “PA” identifications is good to include. The revised text on page 5 of the response suggests the PA 38:5 does not rise from PC fragmentation. Do the authors know where this comes from, as if it was a PA it should show up in the negative mode LC-MS in Table S2 ?It is also possible they arise from fragmentation of other classes like PS. If the authors believe some PAs are detected by MALDI this should be confirmed with MS/MS on tissue in positive mode. If a loss of 124 is observed, this will confirm they are instead PC fragments.

As the text now includes the caveat that many PAs arise from PC fragmentation it would be good to include to the reader how this is the case. Essentially loss of NC3H9 from PC leads to an ion isomeric with PA.

It is not clear how the correlation of MALDI and LC-MS was performed as this cannot be done using ion abundances as they will differ across the two methods. Given the absence of support for many PAs in the LC-MS/MS I wonder if this data is needed or rather the MSI vs LC correlations will just lead to confusion when the point that PAs are largely an artefact should be clear.

I release this is a pedantic point and it should not detract from the excellent and insightful study that I do recommend for publication. For such a possible high impact publication for the lipidomics field, it is important to present the annotated lipids as confidently as possible. The authors have a done a good job of doing this in the revision, I just feel it can be improved a bit.

(Remarks on code availability)

Referee #2

(Remarks to the Author)
The authors have thoroughly addressed all concerns, especially in addressing the pregnancy analyses.

(Remarks on code availability)

Referee #3

(Remarks to the Author)
The authors have satisfactorily addressed all the issues raised

(Remarks on code availability)

Referee #4

(Remarks to the Author)
I co-reviewed this manuscript with one of the reviewers who provided the listed reports.

(Remarks on code availability)

Version 4:

Reviewer comments:

Referee #1

(Remarks to the Author)
I am satisfied that all comments have been addressed and am happy to recommend publication. I would also like to congratulate the authors on the pioneering work

(Remarks on code availability)

**Open Access** This Peer Review File is licensed under a Creative Commons Attribution 4.0 International License, which permits use, sharing, adaptation, distribution and reproduction in any medium or format, as long as you give appropriate credit to the original author(s) and the source, provide a link to the Creative Commons license, and indicate if changes were made.
In cases where reviewers are anonymous, credit should be given to 'Anonymous Referee' and the source.
The images or other third party material in this Peer Review File are included in the article's Creative Commons license, unless indicated otherwise in a credit line to the material. If material is not included in the article's Creative Commons license and your intended use is not permitted by statutory regulation or exceeds the permitted use, you will need to obtain permission directly from the copyright holder.
To view a copy of this license, visit https://creativecommons.org/licenses/by/4.0/

# The lipidomic architecture of the mouse brain

Fusar Bassini et al., 2025
Answers to the referee comments

*We thank the reviewers for their detailed and constructive evaluation. In response, we have revised the manuscript, adding new experimental data, expanded computational analyses, and deeper contextualization of the findings with the pregnancy model. The revision includes over 70 new figure panels (of which 18 in the main figures), 6 new Supplementary Tables, 3 new experimental modalities (microdissection bulk LC-MS, immunofluorescence, single-nucleus RNA-seq).*

*First, we validated the robustness of lipizones by testing over 25 alternative clustering regimes and showing that the core discoveries are replicable. Second, we strengthened the interpretability of lipizones in relation to brain cell types and their projections by estimating cell type lipidomes through deconvolution, comparing with published bulk and single-cell lipidomics data, and performing a systematic connectivity–lipizone specificity analysis. Third, we improved lipid annotation rigor by acquiring Tims-TOF MALDI2 data in negative ion mode, performing systematic PA–PC fragmentation analysis, standardizing sphingolipid nomenclature to LIPIDMAPS conventions, comparing within-class regional abundances in MALDI and LC-MS, and depositing all raw files on Zenodo. Fourth, we reported additional biological insights: inside the major brain divisions, lipizones are as restricted to fine-grained regions as cell types, while they span connected divisions; lipid species within the same class often diverge spatially as much as species across classes, challenging class-level analysis; and lipizones capture heterogeneous transporter and neurotransmitter machinery gene expression rather than metabolic enzymes. Finally, we restructured the pregnancy analysis around orthogonally corroborated findings including white matter HexCer 42:2;O2 increases confirmed by LC-MS, and upstream signaling predictions (CORNETO framework(Rodriguez-Mier et al. 2025)) supported by immunofluorescence and snRNA-seq.*

*We have also refined our claims throughout: lipizones are now framed as method-robust units for studying the brain lipidome, while we avoid describing them as fundamental units, an aspect that only future studies will determine. We now describe lipizones as containing variable proportions of cellular compartments rather than "quasi-cellular", and we demonstrate quantitatively that they outperform anatomical regions in capturing the physiological adaptations occurring in pregnancy. New practical resources include lipizone-aware dissection tables for bulk lipidomics, context-dependent local lipid markers for regionally focused studies, and cell type lipidome estimates released as supplementary data.*

*Manuscript sections are numbered and referred to as "Sx" in the point-by-point response, where x is a progressive numbering, and within sections, we refer to paragraphs as "Py" where y is a progressive numbering.*

## Referee #1 (Remarks to the Author):

**This manuscript by Bassini et al present an elegant and detailed map of the lipidomics architecture of the mouse brain, far beyond any previous studies. While the brain has been mapped at great detail to reveal distinct regions, this has not been achieved on the lipidome level at the depth presented here. The study employs MALDI-MSI and advanced machine learning to map the localization of lipids across serial mouse brain sections across several model systems (e.g. age, sex and pregnancy), to demonstrate how localized lipid environments can adapt to different requirements. Not only do lipid microenvironment (called lipozones) match to cell types and previously identified genetic zones, but new zones are also identified based on lipid composition not previously identified were ground, highlighting the critical, and as yet poorly understood, role of lipid metabolism in defining brain architecture and connectivity. This includes novel information such as oligodendrocyte heterogeneity in the white matter and biochemical compartmentalization within the choroid plexus. The work is highly novel, data analysis is sound and the findings hold significant value across the biological sciences, opening up new means to understand brain metabolism (and dysregulation) and connectivity. The work reveals some very exciting new insights about brain lipid biochemistry. The manuscript has a broad readership appeal to many fields of science and is very timely in regard to interest in brain lipid biochemistry within neurosciences. Based on the findings and their broader implications, the quality of the work, and the excellent presentation of data I am supportive of publication in Nature. I do have several questions and comments that the authors should consider.**

*We thank the reviewer for their thorough evaluation of our manuscript and for the positive comments regarding the novelty, rigor, and impact of our study. We are grateful for the recognition of our efforts to generate a detailed lipidomic map of the mouse brain and for the remarks on the analytical and biological significance of our findings.*

*R1.1*

**The introduction should acknowledge that MALDI-MSI has already been employed across many studies to map lipids in brain, both in 2D and 3D. This does not detract from the novelty of this work, but should be acknowledged with relevant citations.**

*In the revised manuscript, we have rephrased the introduction to clearly acknowledge the body of work that has employed MALDI-MSI to map lipids in the brain in both 2D and 3D contexts:*

*<u>INTRODUCTION, P2 now reads:</u> "... (MALDI-MSI), a technique previously used to map lipids in the brain in 2D and 3D."*

*We now cite **10 additional relevant studies** to reflect these important contributions and to better situate our work within the existing literature.*

*R1.2*

**The MSI is performed in positive ion mode. The authors present a variety of lipids detected that are not typically detected in positive mode MALDI-MSI, such as PG, PI, and PA. While these lipids are likely present in brain, it is not clear if they are truly in the data or just happen to overlap within the ppm tolerance with an unrelated signal. The studies used to validate the assignments both used negative ion mode to detect these lipids. Do the authors have MS/MS confirmation of these. If not I suggest the authors add caveat statement round the detection of lipids that are not typically detected in positive mode MALDI-MSI so non-MS expert can have the knowledge to interpret the data and understand limitations. One way to check is whether the acyl chain distributions match those revealed by LC-MS where presumable lipids elute on a class basis.**

*We agree that lipid classes such as PG, PI, and PA require careful treatment when reported from positive ion mode MALDI-MSI. To address this, in the revised manuscript we have implemented the following:*

1. ***LC-MS/MS support of PGs and PIs*** *– All PG and PI species annotated in our MALDI-MSI dataset are supported by our tandem LC-MS/MS experiments. As pointed out by the referee, these lipids were detected in negative ion mode.*
2. *We have now* ***performed Tims-TOF MALDI2 in negative ion mode*** *to cross-check the PG and PI distributions that we reported in the original manuscript. We were able to detect 5 PGs and 9 PIs, which we have now included as Supplementary figure 2d showing side by side the patterns of these lipids in our original data and the Tims-TOF MALDI2 data for a transparent comparison.*

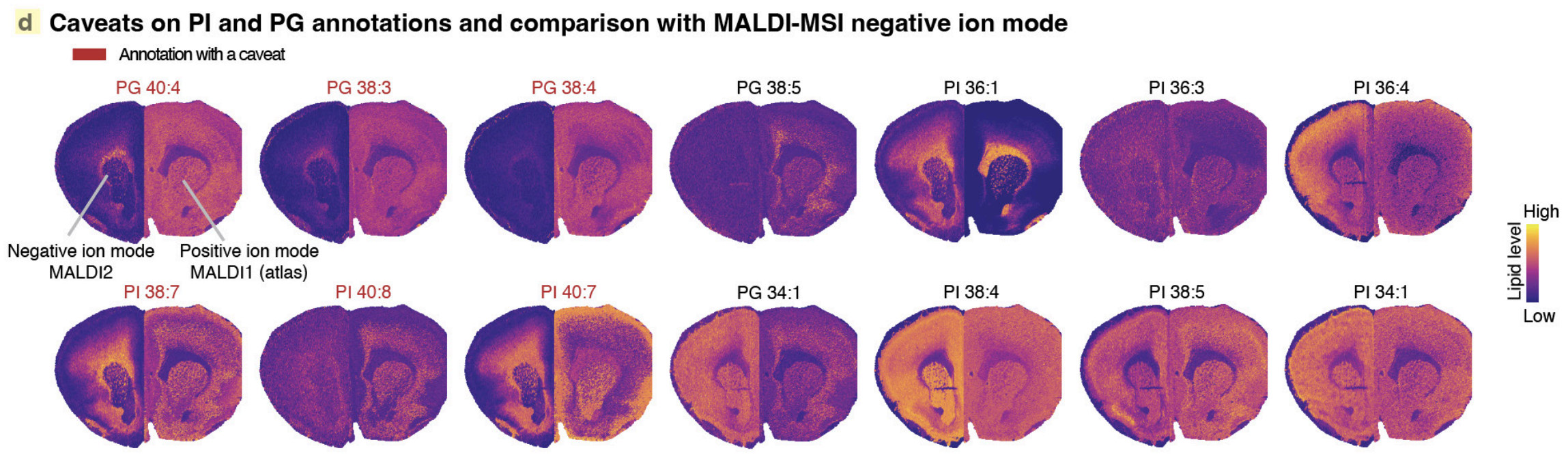


(Supplementary figure 2**d**) Split-brain spatial views for PG and PI lipids; the left half shows negative ion mode data, where PG and PI are typically detected; the right half is the best-matched section from the Lipid Brain Atlas, which is in positive ion mode.

*For PGs, patterns were often different (marked in red), whereas for PIs they were generally in good agreement. We have added a* ***caveat note*** *in the text clarifying that their observation in positive ion mode MALDI-MSI should be interpreted cautiously:*

*Result Section 1 Paragraph 2 now states:*

*"We also used negative ion mode to confirm general agreement with our detections (Supplementary Figure 2d). We note, however, that classes PG and PI, preferentially detected in negative ion mode, were less correlated with LC-MS-determined levels and their spatial patterns should be interpreted with caution."*

3. ***PAs*** *– The PA annotations are supported indirectly through agreement with METASPACE and external datasets (e.g., Fitzner et al. and the HILIC-based in-house LC-MS dataset). For this class, we now include an explicit caveat in the annotation tables and text highlighting the increased uncertainty. More details in response to comment R1.3.*
4. ***Relative abundance consistency*** *– Following the reviewer's suggestion, we have further cross-checked the relative abundance of LC-MS species within each lipid class against the MSI annotations, both across the entire brain and on a regional basis exploiting the Fitzner et al. dataset. This analysis is now reported as Supplementary figures 2a-b. This criterion has now been added to the Supplementary table 2 to flag potential misannotations. Result Section 1 Paragraph 2 now states:*

> *"Overall, the relative whole-brain abundances of lipids within classes were consistent with those previously reported by LC-MS (Fitzner et al. 2020) at the regional level (Supplementary Figure 2a, 2b)"*

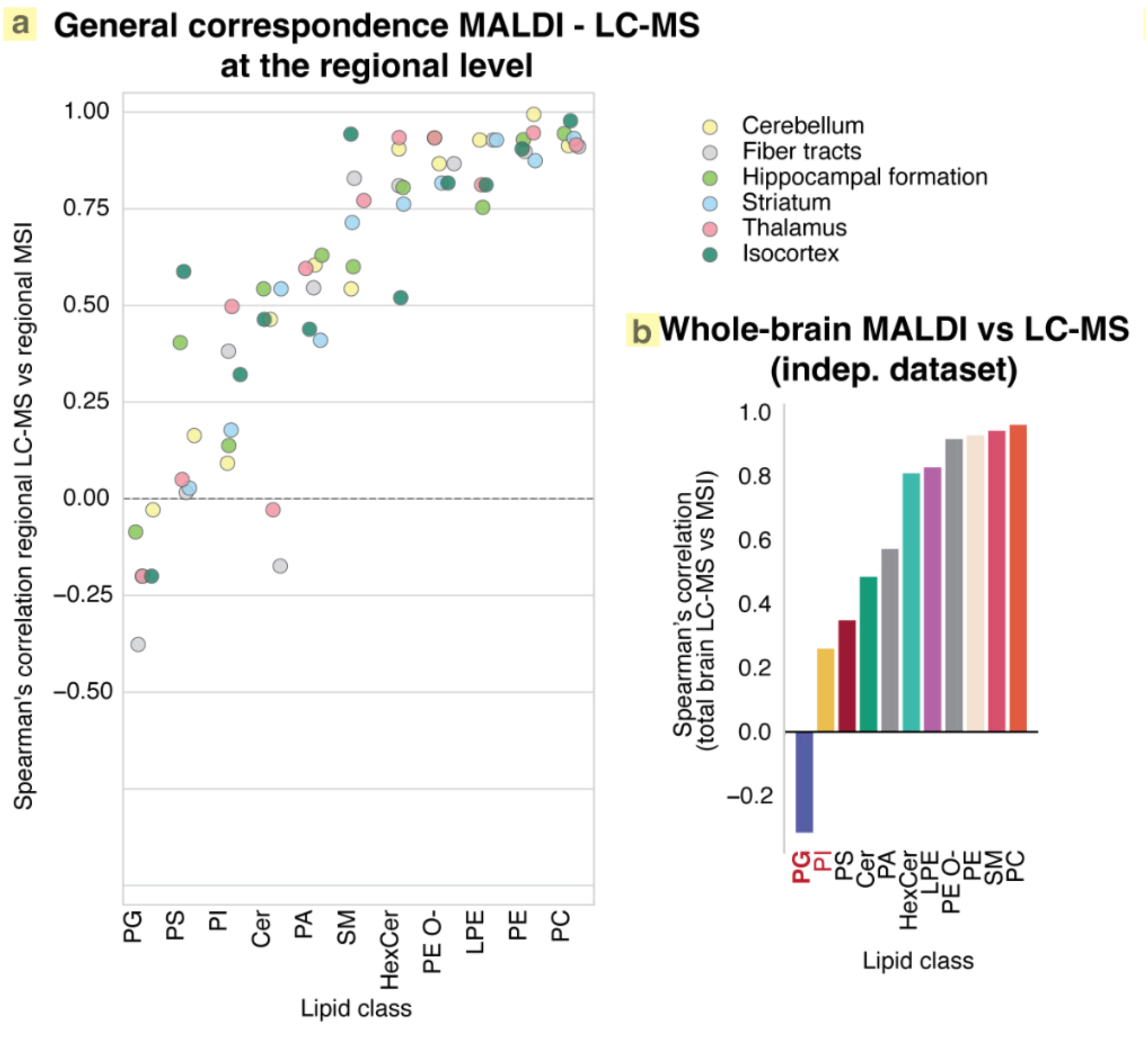


(Supplementary figure 2**a**) Strip plot showing the Spearman's correlation coefficient between the lipid abundance measured by MALDI-MSI and measured by LC-MS/MS (in an independent study), calculated across individual lipids belonging to each major class (x axis). The analysis is stratified by brain region (coloring; one dot per brain region). (**b**) Barplot of the whole-brain Spearman's correlation coefficient for the lipid abundance measured by MALDI-MSI and measured by LC-MS/MS (in the whole-brain dataset acquired for this study), stratified by lipid class. In fact, different classes are known to be uncomparable in MALDI-MSI.

5. *The **Supplementary table 2a** now includes a column that highlights lipids with MS/MS confirmation, a column that highlights lipids detected in positive ion mode, and a column that highlights whether a lipid is commonly detected in positive ion mode or not.*

6. *In the revised manuscript, we have added explicit **caveat statements** regarding PG, PI, and PA detection in positive ion mode and clarified how LC-MS/MS and external datasets were used to validate or qualify these annotations. We believe this added context will allow both MS experts and non-experts to properly interpret the data and its limitations. Specifically, in the Methods section we now state that:*

> *"Importantly, we used both lipids detected in positive and in negative ion mode for lipid annotation of our positive ion mode MALDI-MSI dataset. We next flagged in the main text and in Supplementary Table 2a, b the lipids, especially of class PA, where MS and METASPACE annotation suggest a species that is not detected in any LC-MS dataset in positive ion mode. Annotated PG and PI species are supported by tandem LC-MS/MS experiments (detected in negative ion mode)."*

*R1.3*

**Moreover for PA lipids it is more likely these arise from loss of NC3H9 from PC lipids which happens to a reasonable extent in MALDI and leads to a lipid isomeric with a PA. This can be seen when viewing the spectra on METASPACE. Eg. The PC34:1+K ion at 798.54 fragments to the 739.46 which is incorrectly assigned as a PA. Is there MS/MS confirmation that these are PAs? In Supplementary Fig. 1 lipid class compositional breakdown is shown as pie charts for LC-MSMS/MS and MALDI-MSI. There is a rather sizeable black wedge in the MALDI data set that corresponds to PA, while the LCMS does not display this, further suggesting over representation of Pas in the MALDI data. How does the PA profile by LSMS compare to that revealed by MALDI annotations? Al least limitations of PA detection should be acknowledged and discussions around PA lipids be amended,**

*We thank the reviewer for this observation and acknowledge that the overrepresentation of PAs in positive ion mode MALDI-MSI compared to LC-MS/MS may be a consequence of PC fragmentation, as suggested. To address this issue in a systematic manner, we have now performed an extensive spatial correlation analysis between each putative PA and its corresponding PC species. For each case, we **compared the PA–PC** spatial correlation with the correlations between **PCs and their naturally occurring M+1 isotopologues**. This analysis revealed an instance, specifically PA 38:5, where the correlation coefficient for PC-PA was close to PC-PC+1 (less than 0.2 difference), suggesting that the signals that we assigned to PA 38:5 might originate from PC fragmentation rather than representing a true PA. For the other PA species, this **correlation was not found**, suggesting that they do not likely represent PC fragmentation. These findings are now documented in Supplementary Figure 2c and acknowledged in the manuscript.*

*Result Section 1 Paragraph 2 now reads:*

> *"(PAs: 12 - note: not typically detected in positive ion mode, might result from PC fragmentation, see Supplementary Table 2a; Supplementary Figure 2c)"*

*In light of these results, we have revised the annotation tables to explicitly flag these cases, as described in R1.2.*

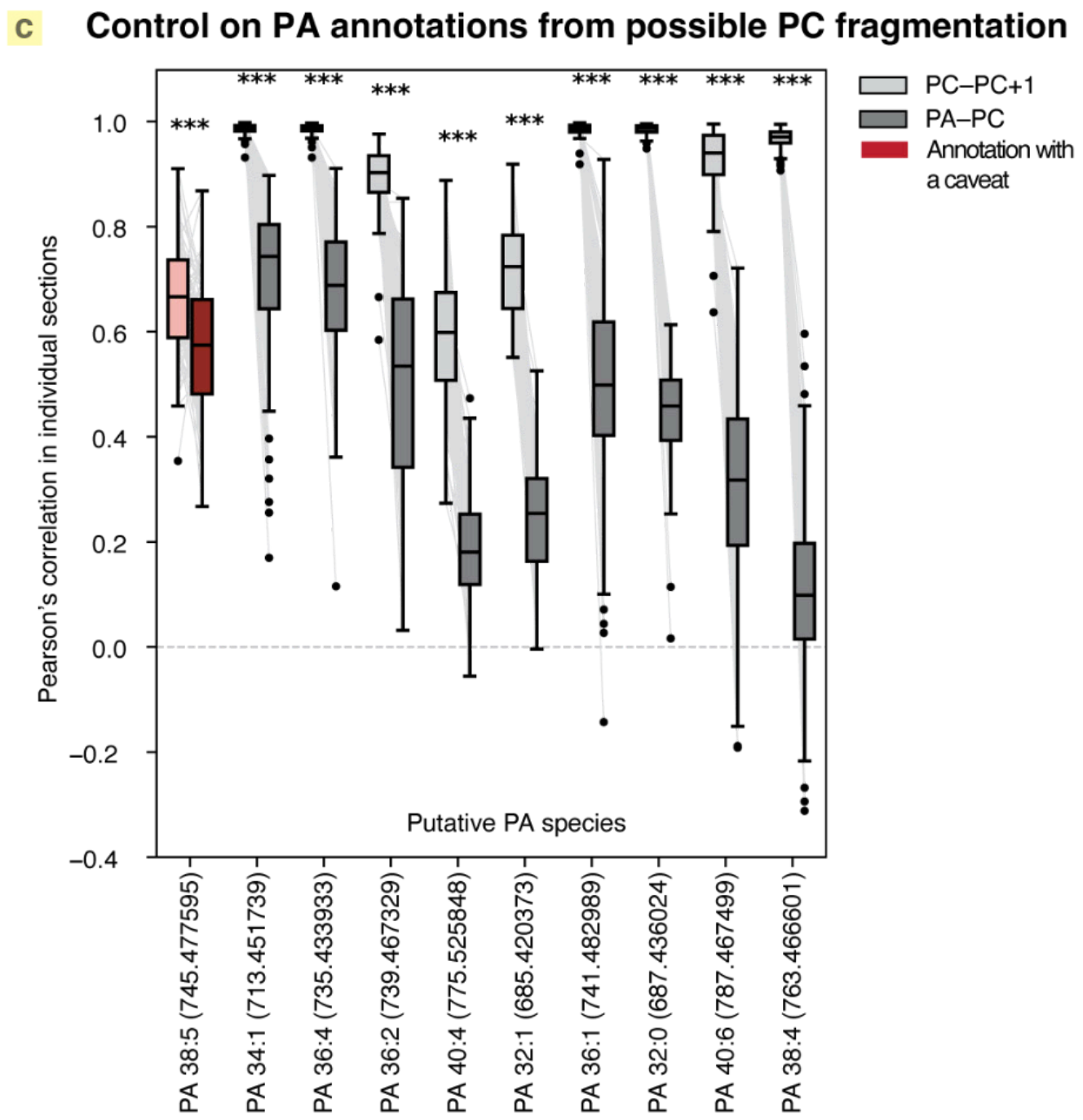


(Supplementary figure 2**c**) Boxplot pairs for the Pearson's correlation between a phosphatidylcholine (PC) and its +1 isotopologue (light boxes) and between a phosphatydic acid (PA) and the same-length, same-insaturation PC (dark boxes), calculated on the raw data on each individual section across pixels. PA annotations with less than 0.2 difference of correlation PA-PC versus PC-PC+1 are labelled in red as possibly ambiguous. Light gray line segments connect same-section correlation values between the box pairs.

*R1.4*

**Throughout the manuscript sphingolipids do not conform with standardized LIPIDMAPS Consortium nomenclature, e.g., SM 34:1 should be SM 34:1;1 etc. For MALDI the inclusion of the ";2" OH number is particularly important for ceramides as cations can arise as either/both [M+H]+ and [M-H2O+H]+, which can subsequently lead to species misidentifications. In particular, the reviewer draws note to the in-text sphingolipid annotations of Page 10, column 2 where both types of nomenclature are used for HexCer's. The reviewer suggests that the all sphingolipid shorthand nomenclature be standardized throughout and always display the OH number (e.g., either XX 42:2;2 or XX 42:2;O2).**

*We have revised the manuscript to ensure that sphingolipid annotations now follow the* ***standardized LIPIDMAPS*** *Consortium nomenclature, consistently including the OH number. This has been corrected throughout the main text, figures, supplementary tables, and the website.*

*R1.5*

**Figure 3A- can the authors comment on how this correlation is calculated. Is the MSI data based on normalized intensities**

*We thank the reviewer for raising this point. The gene expression data are derived from the* ***scRNA-seq dataset of Langlieb et al.*** *(Langlieb et al. 2023), where counts are normalized by the total counts across all genes, followed by log1p transformation. The MALDI-MSI lipid data are* ***normalized at the pixel level*** *using our recently published method uMAIA, which performs a regularized distribution matching procedure that preserves biological variability between sections while avoiding over-normalization ((Schede et al. 2025), now in Nature Methods). The normalized lipid intensities are then averaged at the cell type level. Finally, Pearson's correlation coefficients are calculated using 1,619 cell types (those with >20 usable cells across warped sections) as data points. We have now described this procedure in greater detail in the Methods section and clarified it in the legend of Figure 3.*

*In the Methods section we now state that:*

> *"For the lipid-enzyme correlation analysis, gene expression data is derived from the scRNA-seq dataset by Langlieb et al. and MALDI-MSI lipid data are normalized at the pixel level using uMAIA; genes and lipids are averaged at the cell type level. Finally, Pearson's correlation coefficients are calculated using 1,619 cell types (those with >20 usable cells across sections) as data points."*

*R1.6*

**Figure 5 and text – when discussing ventrical markers. Is it possible some this arises from residual blood? Were the mice perfused?**

*The mice were not perfused in this study. To minimize confounding effects, we performed visual inspection for residual blood, and one section that was clearly contaminated was removed from the dataset (already in the original manuscript). In the remaining sections, no obvious blood contamination was detectable. This assessment is further supported by METASPACE analysis of known blood-associated molecules such as heme (m/z 653.1451224), which appears only minimally in the meninges at the tissue periphery and at very low levels in the interior of the ventricles. Here, we show heme in red, compared with sphingomyelins in blue and green:*

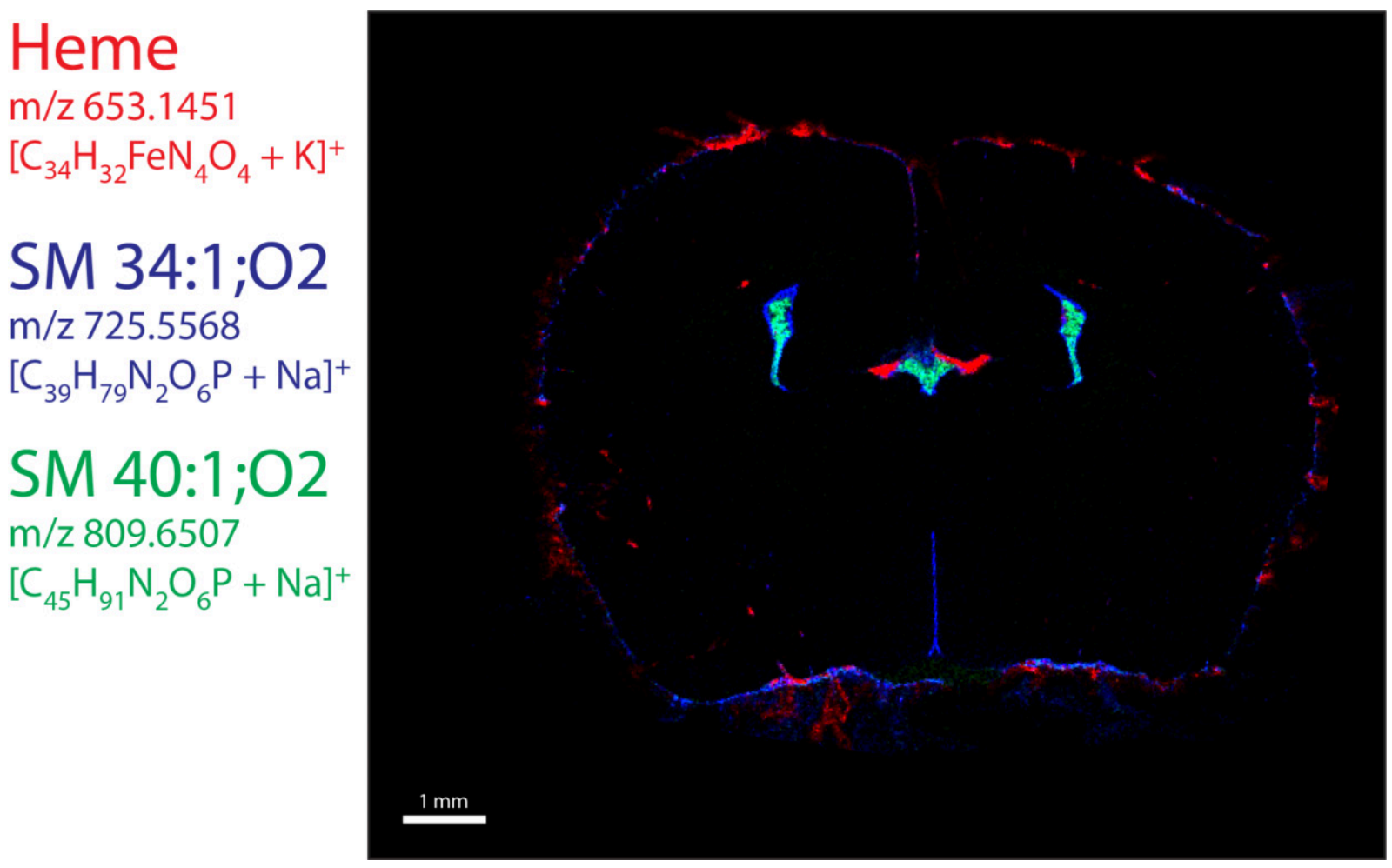


(**Rebuttal Figure 1**) Spatial plot showing heme (red), SM 34:1;O2 (blue) and 40:1;O2 (green).

*Two lipids mentioned in the manuscript, SM 40:1;O2 and SM 34:1;O2, are also found enriched in* ***blood in bulk lipidomics*** *(Surma et al. 2021); to confront the possibility that residual blood would result in artifactual ventricular heterogeneity, we have now* ***confirmed all the results*** *on the choroid plexus (ChP; subclustering, differential proportions between lateral and fourth ventricles, lipid markers, differential similarity of the two ChP lipizones with anterior and posterior lipizones, respectively) by repeating the analysis omitting 16 lipids known to be enriched in blood and present in our MALDI-MSI dataset.*

*Here we show to the Referee the results of this analysis that, for conciseness, we have not included in the updated manuscript. We have nevertheless acknowledged this point:*

> ***S6P4***: *"We acknowledge that SM 40:1;O2 and SM 34:1;O2 are also among the blood markers in bulk lipidomics (Wilcoxon test; BH-FDR correction, p<0.0001) (Surma et al. 2021); to confront the possibility that residual blood would result in artifactual ventricular heterogeneity, we confirmed all results omitting blood lipid markers."*

*Here we show the subclustering without the 16 blood lipid markers (using ventricles only for subclustering): we highlight with a confusion matrix the correspondence with the subclustering reported in original manuscript, and show the proportions of the two main ChP lipizones, which confirmed the original observations.*

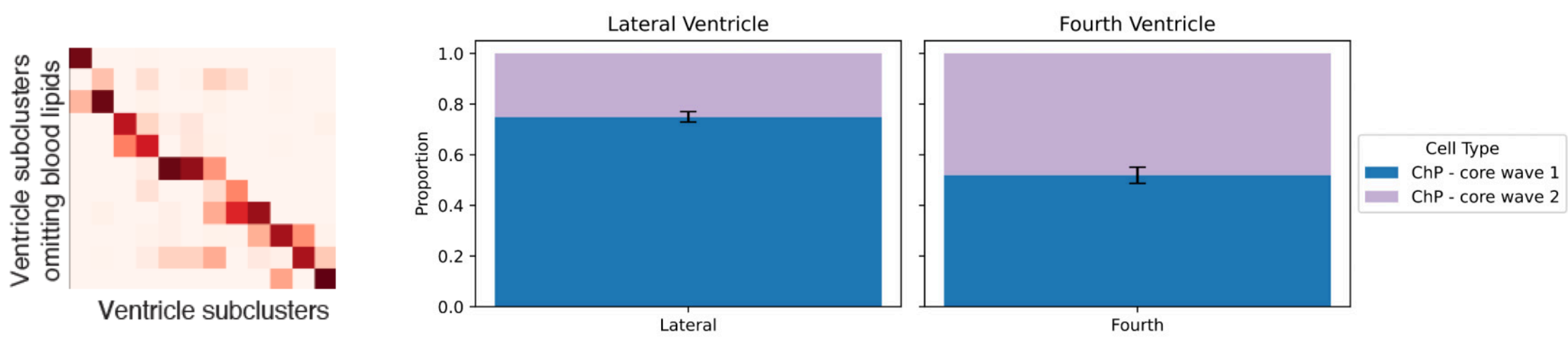


(**Rebuttal Figure 2**) Heatmap for the confusion matrix comparing the ventricular subclustering when including or excluding blood markers. Stacked barplot of the two main ChP clusters showing variation in proportion between lateral and fourth ventricles.

*Developmental and biochemical assertions were also confirmed when omitting the 16 lipids also enriched in blood.*

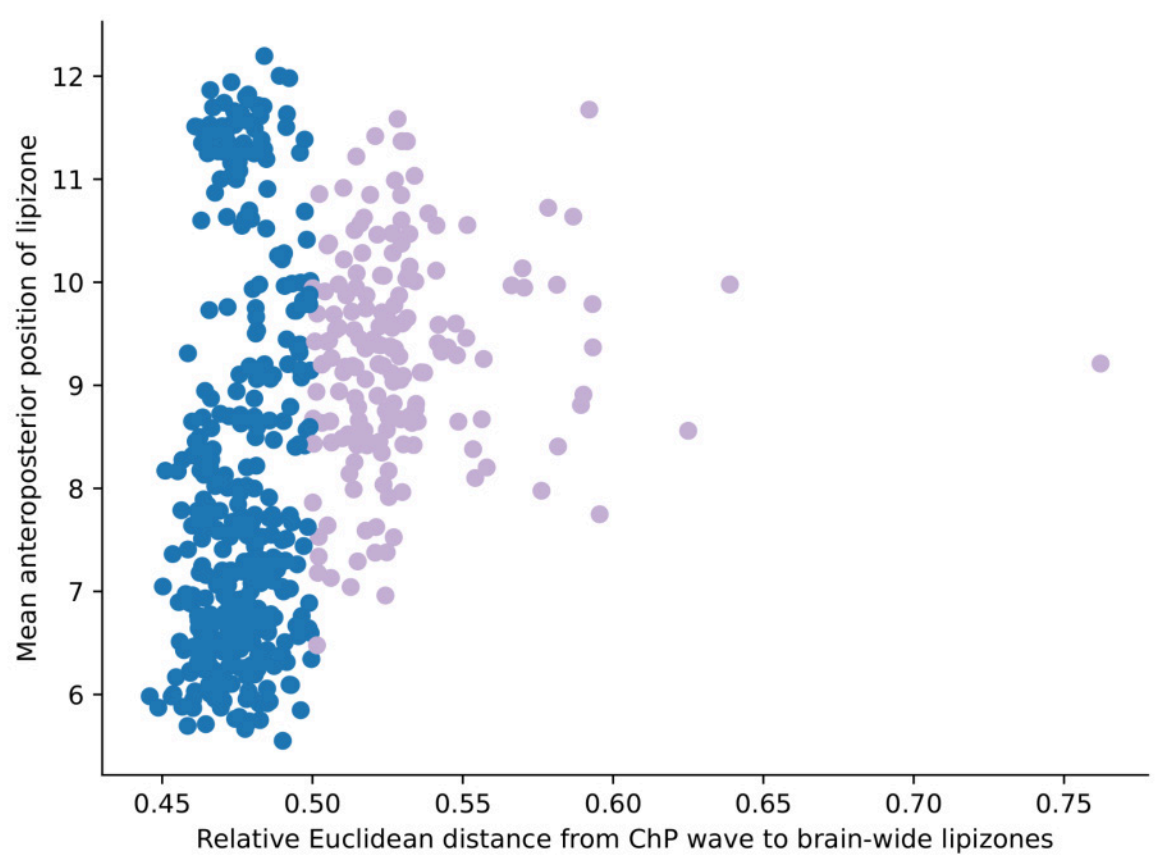


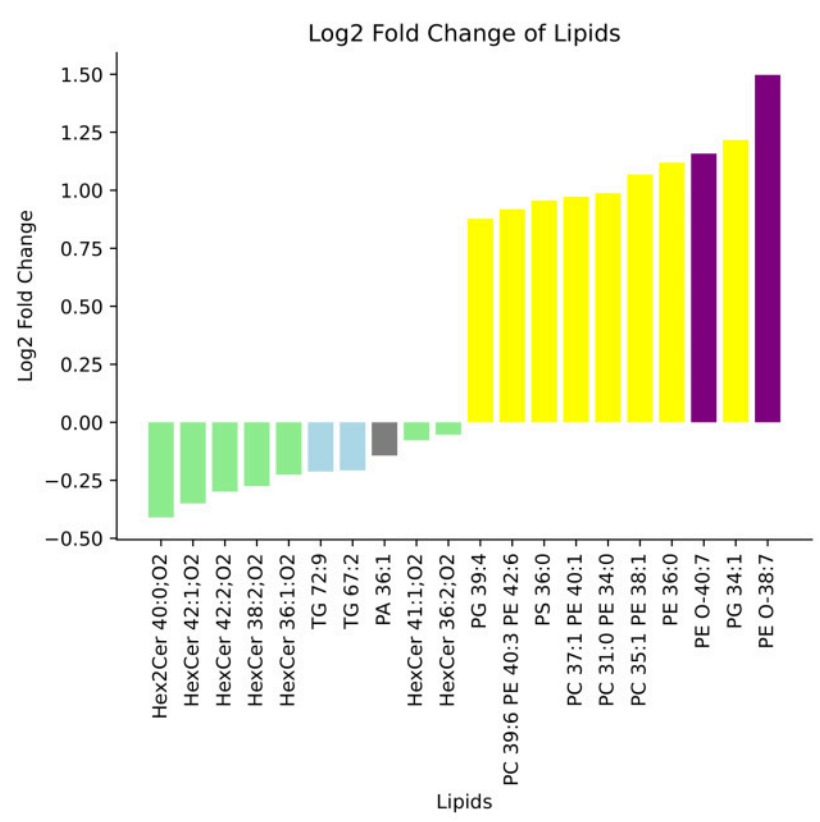

(**Rebuttal Figure 3**) Scatter plot of position along the anteroposterior axis (x axis) against the similarity with two choroid plexus *lipizones* (y axis), each point is a *lipizone*. Barplot of the log2FCs of lipids enriched in either of the two major lipizones of the choroid plexus.

*The proportion barplot and scatterplot correspond to* *<u>Figure 5e</u>* *of the manuscript:*

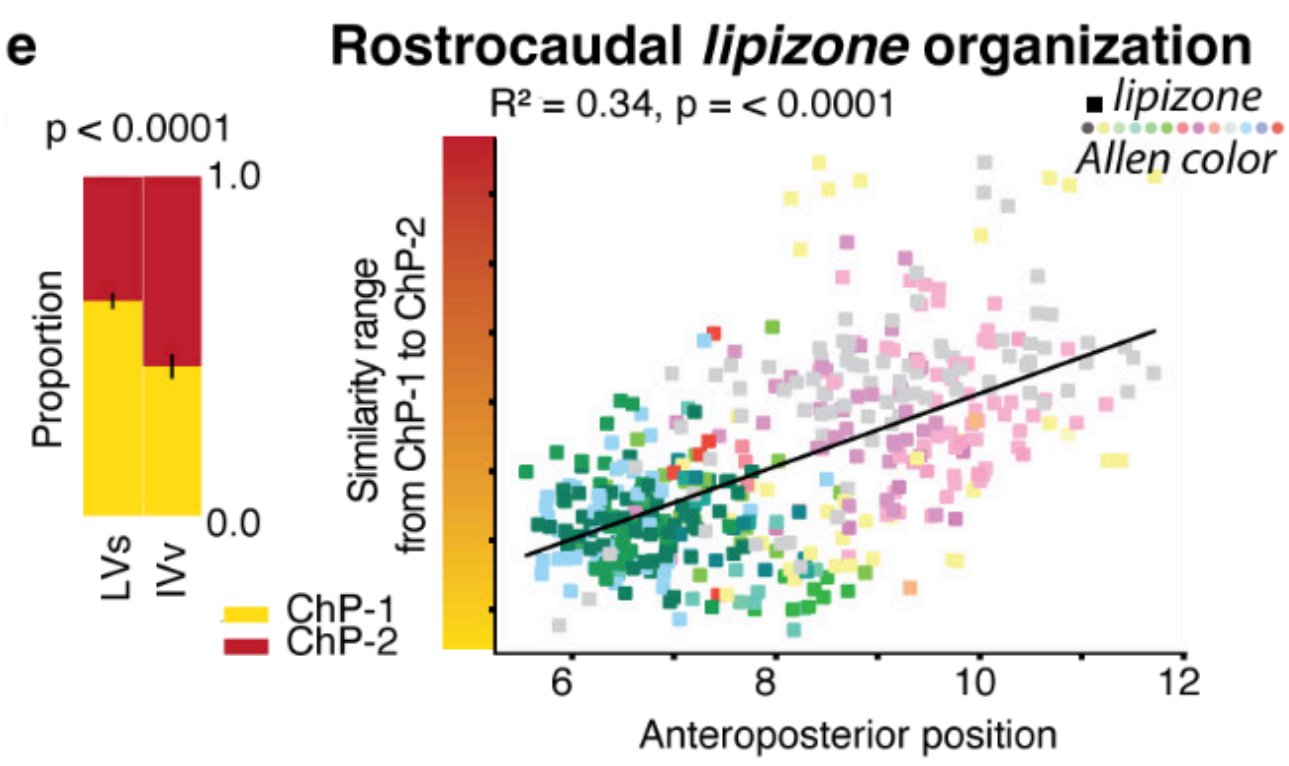


(Figure 5**e**) Left: stacked barplots of the proportions of the two core choroid plexus *lipizones*, in the lateral (LVs) and fourth (IVv) ventricles. Right, scatter plot of position along the anteroposterior axis (x axis) against the similarity with two choroid plexus *lipizones* (y axis), each point is a *lipizone* and is colored by its Allen Brain color.

*The lipid log2 fold change barplot corresponds to* *<u>Supplementary Figure 19i</u>* *of the manuscript:*

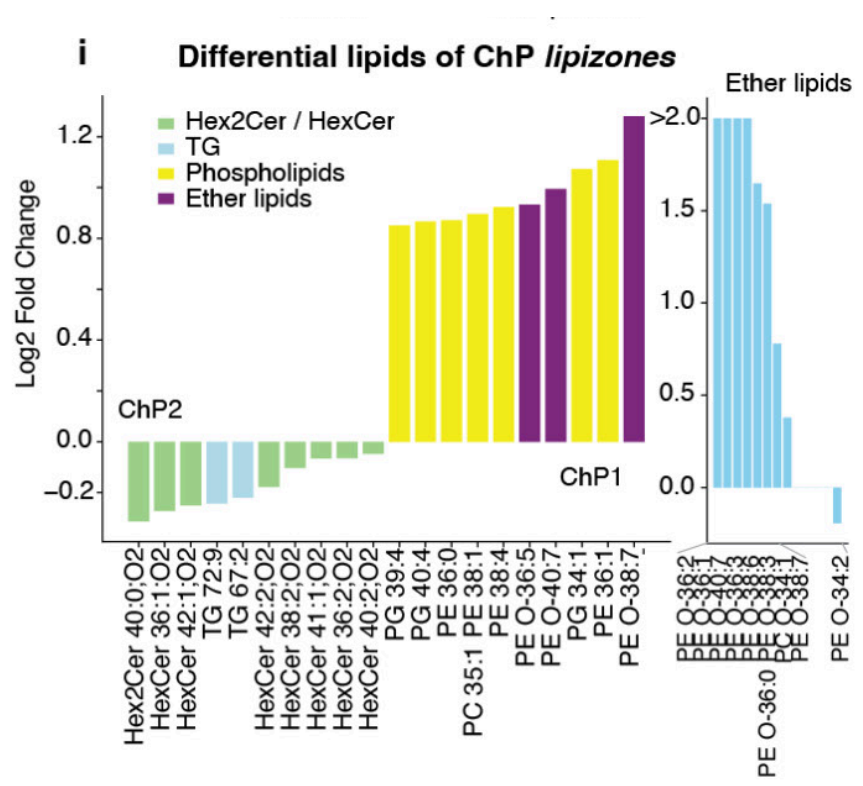


(Supplementary figure 19**i**) Left, barplot of ChP1 and ChP2 marker lipids; right, lightblue-colored barplot showing ether lipid imbalance.

*R1.7*

**For the comparison of sex differences, how many replicates were analyzed?**

*For the sex-differences comparison, we have now included* ***detailed replicate information*** *in the Methods section:*

- *3 male (M) brains: M1 = 5 sections, M2 = 4 sections, M3 = 6 sections*
- *3 non-pregnant female (F) brains: F1 = 4 sections, F2 = 5 sections, F3 = 5 sections*
- *3 pregnant female (P) brains: P1 = 6 sections, P2 = 6 sections, P3 = 6 sections*

*Furthermore, we have now added two additional pregnant female brains (P4, P5) MALDI-MSI (6 sections each) to the PCA analysis as per Reviewer 2's request.*

*R1.8*

**there is a paper that found a negative correlation between pregnancy and AD – this could be a good addition to your discussion.**
**https://doi.org/10.1177/1533317518786447**

*Thank you for the suggestion. We have now extended the final paragraph of the* ***Discussion*** *highlighting how our atlas could be used to explore physiological variation (e.g., post-pregnancy changes) and disease (e.g., Alzheimer's disease), and we have cited the suggested study reporting a* ***negative correlation between pregnancy and AD****.*

*In the DISCUSSION, P8, we now state that*

> *"Similarly, an analysis of the persistence of the lipidomic changes established during pregnancy through time could help explain physiological variation between females that underwent pregnancy and those that did not. This is relevant, for example, as pregnancy is associated with lower AD risk, and AD is intimately linked to lipids."*

*R1.9*

**It is stated that all raw data is on metaspace. However when clicking on the link there are only a handful of individual sections.**

*The full dataset (all 132 pre-filtering sections) is available on METASPACE (https://metaspace2020.org/project/mlba-2025 updated to* ***public link****, now in the Manuscript). We have now added* ***instructions*** *noting that users need to click "See all datasets" at the bottom of the page to view the entire corpus (as only a subset is shown by default in METASPACE).*

*R1.10*

**Further METASPACE is already processed data as it is centroided and converted to imzML. For such a paper, providing access to the underlying raw data would be helpful to the community. Can the authors also provide access to .raw files from the orbitrap.**

*We agree with the reviewer on the importance of making the underlying raw data accessible to the community. We have now deposited all* ***RAW/UDP files on Zenodo****, and we provide an additional supplementary table listing the corresponding links for each dataset (Supplementary table 11). These have been referenced in the Data Availability statement of the manuscript. For example, one such deposition can be found at: https://zenodo.org/records/17068820.*

*In the Data availability statement, we now clarify both R1.9 and R1.10:*

> *"All data is granted public access. Raw data are available on Zenodo (see Supplementary Table 11); iMZML and IBD datasets are available on METASPACE at https://metaspace2020.org/project/mlba-2025 (click on "See all datasets" at the bottom of the page to reveal all 132 datasets);..."*

*R1.11*

**Something to note about the web-application, is that sometimes when selecting a new rostro-caudal axis section it doesn't always change the display to that section depth – instead displaying the previously selected section depth. Moving away from and back to appears to fix it, but it may be worthwhile checking to see if this can be resolved.**

*Thank you for noticing this. We have **fixed** the bug in all pages.*

## Referee #2 (Remarks to the Author):

**This manuscript describes a large-scale spatial lipidomic profiling effort in the mouse brain using high-resolution MALDI-MSI that delivers 5 micron spatial resolution at 25 micron intervals. The dataset spans 11adult brains that includes 2 densely sampled males (32 coronal sections each, corresponding to about a third the frequency of sampling of current neuroanatomical and spatial transcriptomics atlases), and six sections each from 3 additional males, 3 females, and 3 pregnant females. The main finding of the paper is the division of the brain into hundreds of discrete lipid-defined zones ("lipizones")**

*We thank the reviewer for their careful evaluation of our manuscript. The reviewer's specific concerns about data presentation clarity, biological validation, and interpretation have prompted important revisions that we believe have significantly improved the work.*

*R2.1*

**Especially for all main findings regarding lipizones—e.g. the barrel cortex-specific lipizones, and "inside vs outside" lipizones, it would be reassuring to do some additional pressure testing of clustering reproducibility across brains. This would include transfer accuracy reporting across all brains (not just brain 2) and an assessment of cluster stability using resampling and feature downsampling.**

*We thank the reviewer for this important comment.*

*In the revised manuscript, we have validated the clustering by testing **27 alternative clustering regimes**, rigorously comparing them, and repeating all major analyses under each regime. We combine here our responses to **both Referee #2 and Referee #3 toward** validating lipizones as **data-driven** rather than method-dependent.*

*The alternative regimes were designed to probe distinct sources of potential instability. To test sensitivity to **feature composition**, we downsampled lipid features to 60%, 75%, or 90%, and separately re-ran the clustering after including imputed lipids. To test sensitivity to **spatial sampling**, we downsampled sections to 12, 18, or 24 of the 27 available, equally spaced (resampling). To test sensitivity to **algorithmic choice**, we replaced our binary splitter with Leiden, Louvain, spectral, and kMeans clustering, and also clustered on the embeddings of lipiMap. To test sensitivity to **parameter settings**, we explored plausible parameter sets around the defaults. Finally, to test **cross-subject transferability**, we clustered Brain #2 independently and transferred its labels onto Brain #1.*

*We evaluated these regimes at two levels. At the aggregate level, regimes whose clusters matched well the reported lipizones also have better lipid explanatory power (i.e., they explain more variance, Pearson R = 0.61 between normalized mutual information and $R^2$, <u>Supplementary figure 5c</u>), confirming that the lipizones capture meaningful structure, recapitulated across clustering regimes, rather than an arbitrary partition. At the level of individual discoveries, we provide <u>Supplementary file 2</u> with **one page per regime**, each reproducing the core lipizone-dependent figures, including "inside-outside" organization, cell-type colocalization, barrel-field cluster, connectivity analysis, choroid plexus composition, cerebellar layering, white matter heterogeneity, oligodendrocytes explaining white matter*

*diversity, and axonal stream identification. These analyses demonstrate that our findings are robust across methodological variations.*

*We have included these results with full details in the Methods and Supplementary figure 5a, b.*

*We have also extended the label transfer accuracy assessment to the control brains, showing the pairwise correlations for the lipizone regional distribution between individual brains (Supplementary Figure 5d).*

> ***S1P4:*** *"27 alternative clustering regimes were also tested, and compared quantitatively (Supplementary Figure 5a, c); the main discoveries in this paper were tested with these alternative clusterings and were replicable (Supplementary Figure 5b; Supplementary file 2)."*

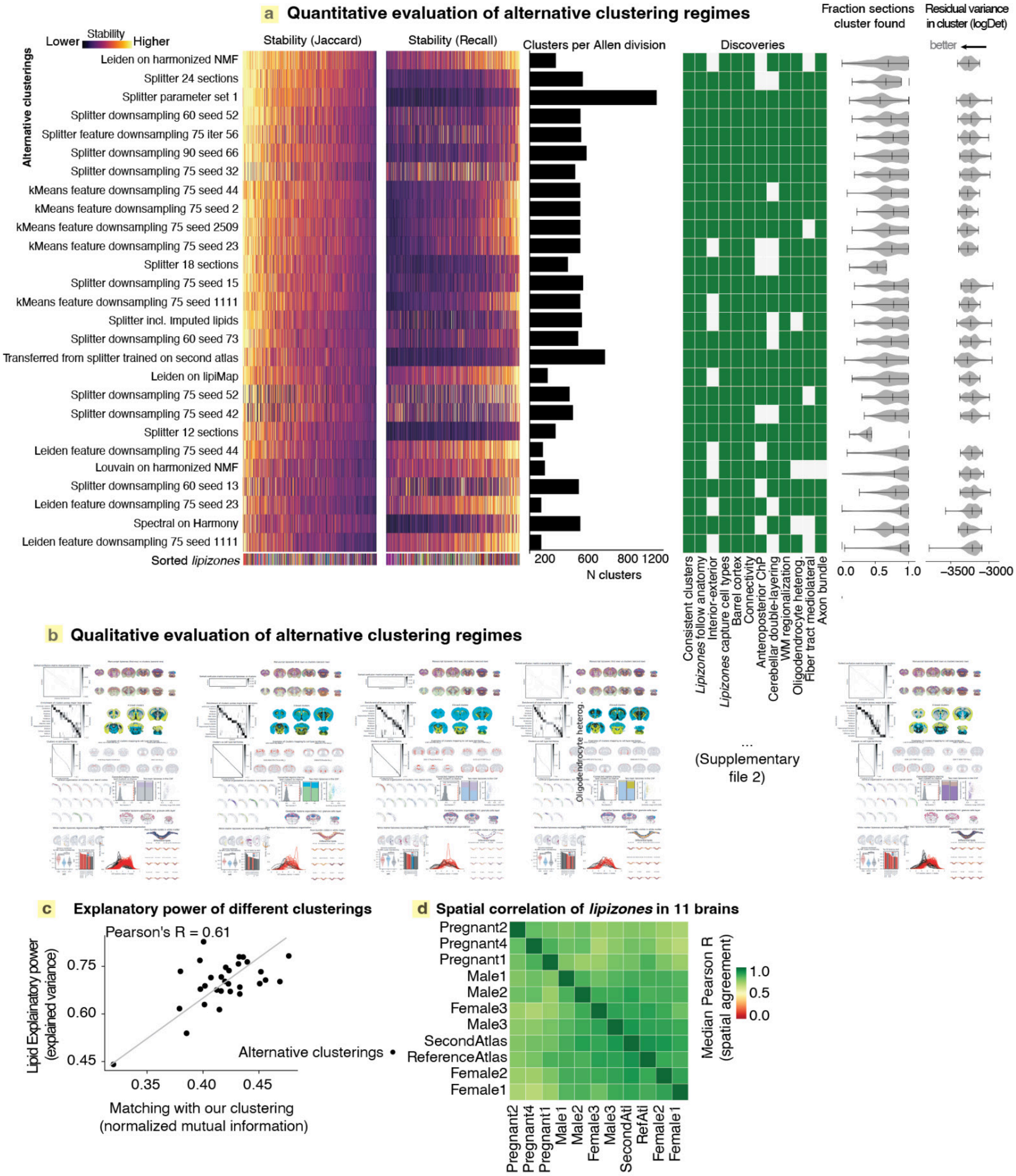


**Supplementary Figure 5. Validation of *lipizones* with alternative clustering methods and across subjects.** (**a**) Set of plots showing metrics to evaluate the consistency of the findings reported throughout the manuscript for alternative clustering regimes (rows): from the left heatmap showing for each cluster the for the Jaccard index with the matching lipizone (columns, see leaf coloring), heatmap for the recall of *lipizones* (columns, see leaf coloring), barplot of the number of clusters, binary heatmap reporting whether thediscovery (columns) is reproduced under each of the clustering regimes for the fraction of brain sections where a given cluster is found, and the residual variance by cluster (lower means  more variability is captured by the partition). (**b**) Thumbnails of the clustering reports for each clustering regime that is provided as a separated  Supplementary file 2. (**c**) Scatterplot showing for each clustering regime the fraction of explained variance as a function of  the similarity of that clustering with with our reference lipizone model . Note how the more a model is similar to ours explain better the variance in the data. (**d**) Heatmap of the pairwise spatial agreement of *lipizone* distribution across individual brains measured as Pearson's correlation of the vector of relative *lipizone* abundances of each Allen Brain Atlas regions.

*For completeness, we also show here the label transfer accuracy violin plot reported for the second atlas in the original manuscript (current Figure 4c) repeated on all the other brains (Rebuttal figure 4).*

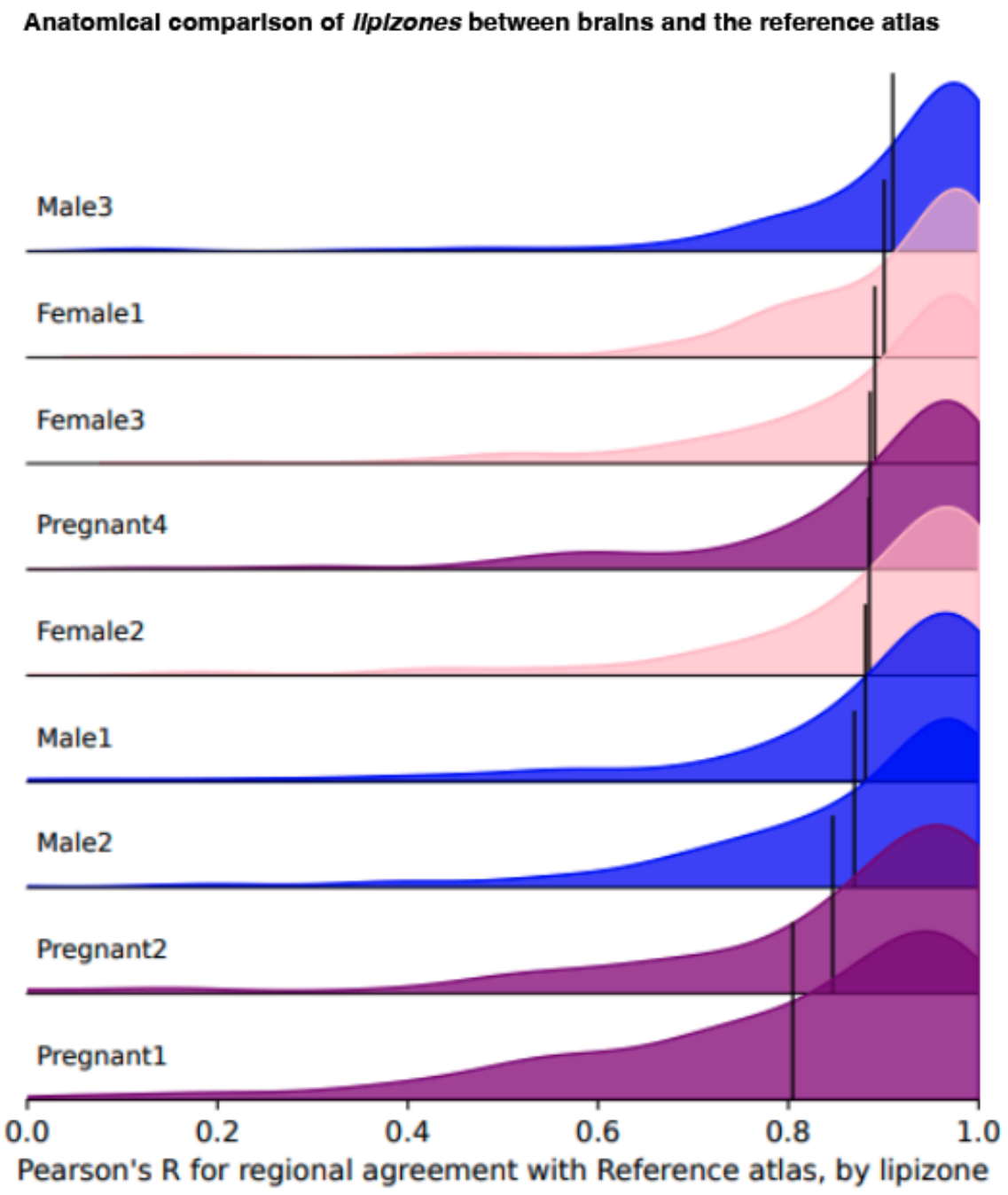


(**Rebuttal figure 4**) Frequency histograms of Pearson's R for Allen region pairs between brain #1 and the other brains in terms of *lipizone* localizations in Allen regions.

*R2.2*

**The claim that some lipizones correspond to patterns of connectivity or functionally related cells could be better supported. For every pairing of two cell types—like layer 6 paired with thalamic cells—there are plenty of other canonically connected cells (like thalamic cells to layer 4 cells, for example!) that do not seem to be paired. The main support is a permutation test controlling for inter-regional distance that shows connected regions share lipizones more often than expected (this is just a single suggest correlation). It would be helpful to also report the absolute differences in sharing probability, rather than just a p-value for this analysis. The test is also binarized between "high" and "low" connectivity; it would be helpful to motivate the selection of this binarization by examining the distribution and, if appropriate, showing robustness to alternative thresholds.**

*We thank the referee for this comment. We have now extended the connectivity–lipizone analysis by reporting **absolute effect sizes**, by **varying thresholds** systematically, and by conducting a targeted **specificity analysis** of cortical layer 4.*

*First, in the revised manuscript we report that connected regions share lipizones **2.89×** more frequently than distance-matched permuted regions. Rather than relying on a single binarization, we now present results across variable connectivity and regional enrichment thresholds, showing the relationship remains significant ($p < 0.0001$) from the **75th to the 95th percentile** of connection strength, with all effect sizes above 2× fold change (Supplementary Figure 16c). We also assessed the relationship from the opposite direction: pairs of regions that share lipizones are **2.44×** more likely to be connected (15.3% versus*

*6.3%; Fisher exact p < 0.0001), and this too is robust across thresholds (<u>Supplementary Figure 16d</u>).*

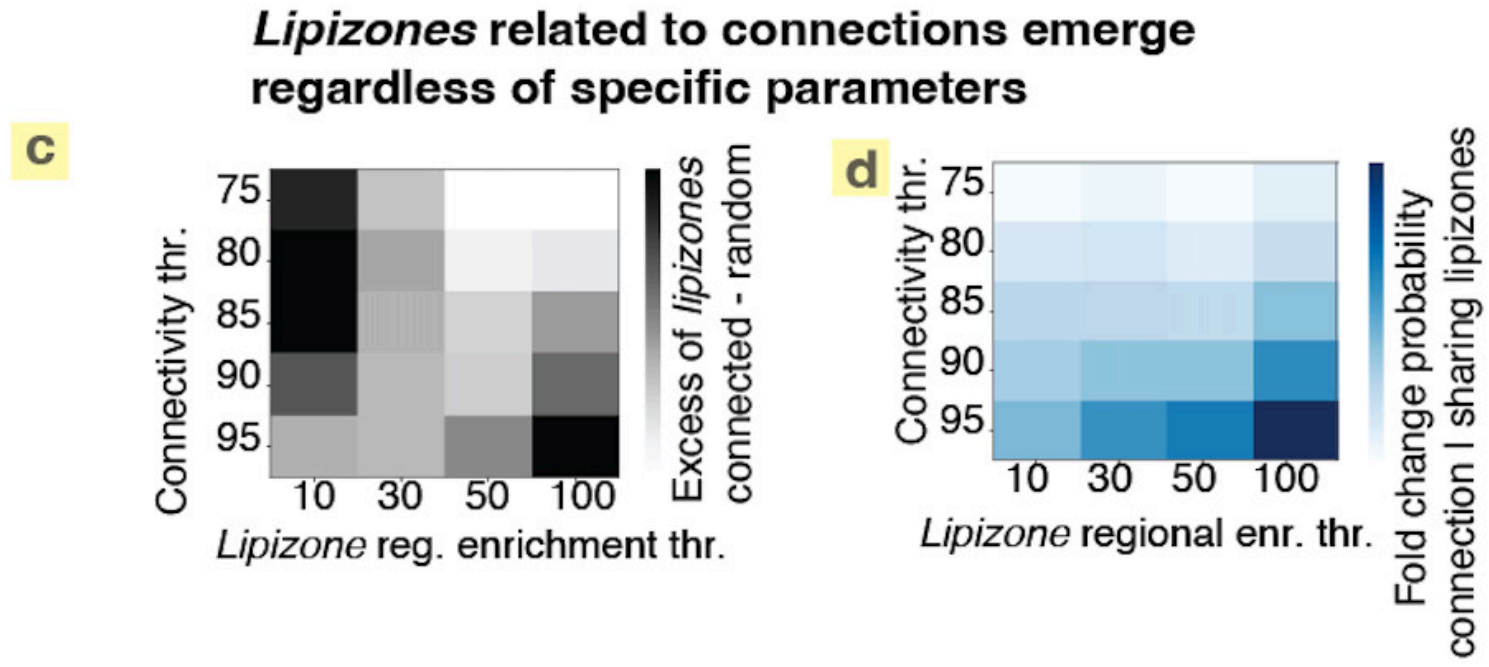


(Supplementary figure 16**c**) Heatmap showing the excess of shared *lipizones* between connected versus null-model region pairs (*see Methods*), across varying thresholds for calling a connectomic connection (y-axis) and a *lipizone* regional enrichment (x-axis). (**d**) Heatmap showing the fold change in connection frequency for region pairs that share *lipizones* versus null-model region pairs, varying the threshold for calling a connection from the Allen mesoscale connectome (y-axis) and the threshold for calling a *lipizone* regional enrichment in the Lipid Brain Atlas (x-axis).

*Second, we conducted a **targeted analysis of cortical layer 4**, where input connectivity is well characterized. We extracted all terminal lipizones and intermediate clusters enriched in layer 4 subregions, and flagged those simultaneously enriched in a region outside the cortex. We then screened these pairings against published connectivity literature to determine whether they correspond to known connections. This analysis revealed cases where, at some level of the lipizone hierarchy, layer 4 shares cluster identity with its plausibly **connected input regions**, including temporal association areas with CA1, entorhinal, and amygdalar regions, and visual cortex and barrel cortex with superior colliculus (<u>Supplementary Figure 16b</u>). With our current lipidomic parcellations, however, direct thalamus to cortical layer 4 connections have not emerged. In the revised manuscript, we added the following sentence:*

> ***<u>S4P7</u>**: "Nonetheless, some connections, such as those between the thalamus and the thalamic recipient cortical layer 4, are not exposed by our lipidomic hierarchy."*

*What predicts whether a given connection may be detectable from the lipidome remains open and, to us, an exciting question for future work. Nonetheless, the systematic analysis below provides a detailed and systematically view to connections that are or are not captured.*

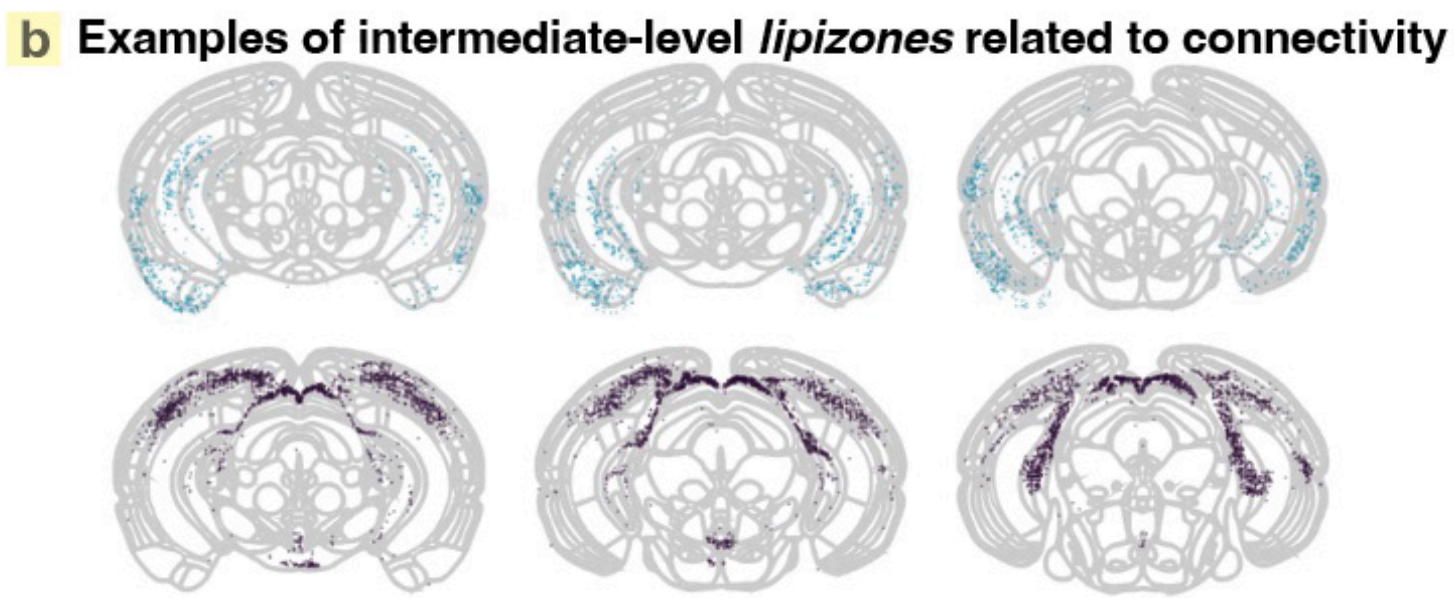

(Supplementary figure 16**b**) Spatial plots of representative intermediate-level *lipizones* whose territory spans pairs of regions that are canonically connected.

*Third, we have introduced a systematic connectome-wide analysis, fully detailed in our answer to R2.3 below.*

*R2.3*

**Given that only some connected pairs show lipizone sharing, it might be helpful to do a specificity analysis—for example, in cortex, where inputs and outputs are well established, the authors could examine whether specific pairings (e.g. corticothalamic versus corticocortical or corticostriatal) drive clustering behavior.**

*We agree that understanding which connection types are preferentially captured by lipizones is as informative as the overall association, and we have now conducted this* ***specificity analysis*** *across the full connectome.*

*As a first step, we classified every connected region pair in the Allen mesoscale ipsilateral connectome(Oh et al. 2014) as sharing or not sharing lipizones above chance (structured permutation accounting for lipizone prevalence and inter-region distance; see Methods). The result is visualized as an annotated connectivity matrix (Supplementary Figure 17a), where each connected pair is marked as non-sharing, sharing but not significantly, or significantly sharing, the latter colored by the major shared lipizone. This provides an atlas-wide view of which connections are "visible" through lipid organization.*

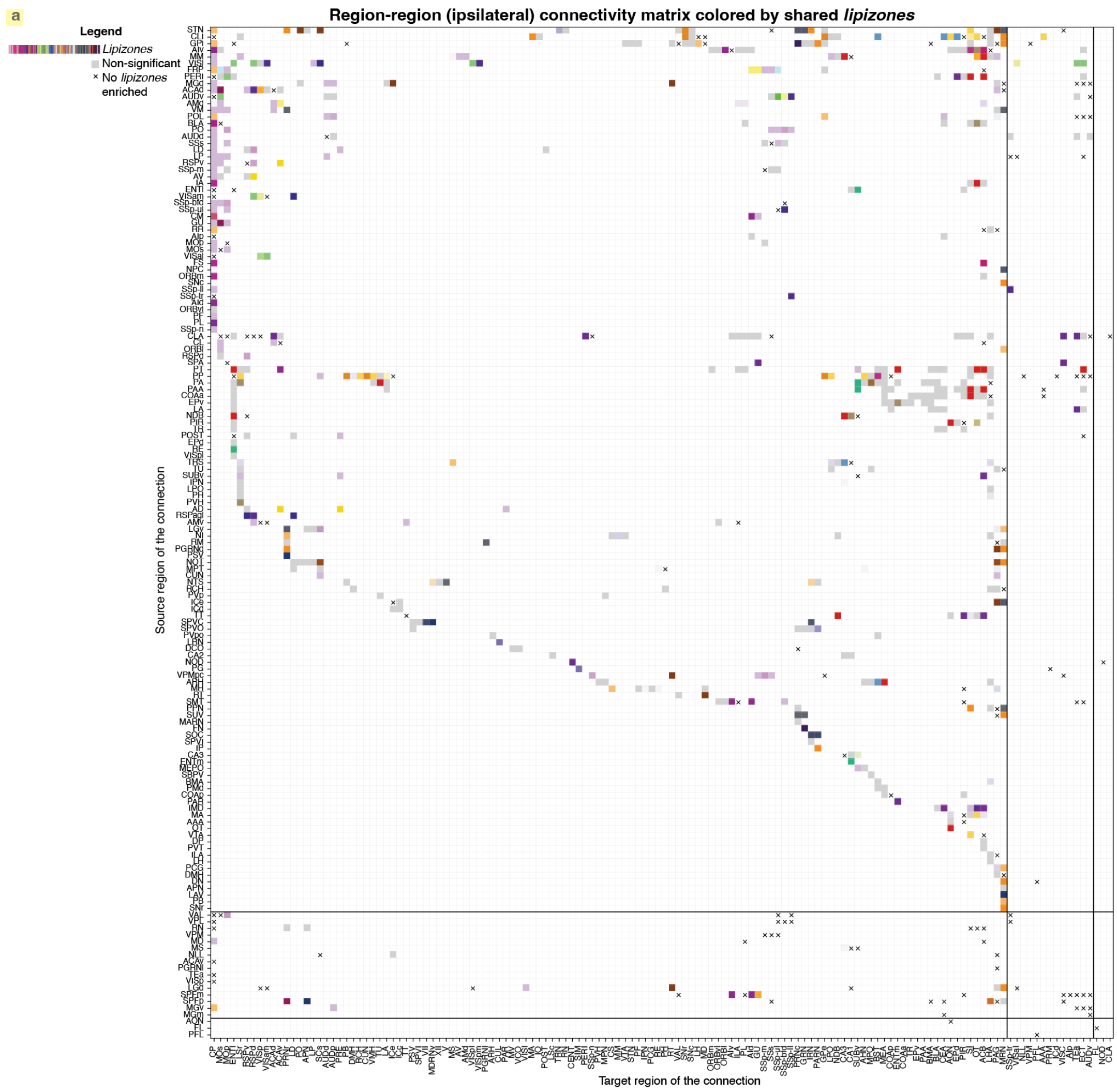


**Supplementary Figure 17. Mesoscale ipsilateral connectivity matrix annotated by *lipizone* enrichments.** (**a**) Heatmap of the thresholded ipsilateral mesoscale Allen Brain connectome. Regions are sorted by brain division. Non-white entries denote connected region pairs. "x" marks indicate connections whose start and endpoint do not share any *lipizone*. Light grey squares indicate connections whose start and endpoint share *lipizones*, but not significantly (stringently tested against a null model that accounts for other sources that could explain the sharing; *see Methods*). Coloured squares indicate connections whose start and endpoint share *lipizones* significantly, coloured by the dominant shared *lipizone*.

*We then directly addressed the reviewer's question about **type-selective** connectivity by stratifying these results by source–target brain division pair (e.g., corticocortical, corticothalamic, thalamic-hypothalamic). For each division pair, we calculated the fraction of connections whose source and target significantly share at least one lipizone (BH-FDR < 0.05). This analysis reveals that the lipid–connectivity relationship is not uniformly distributed: for example, **midbrain-to-hypothalamus** and **pallidum-to-midbrain** connections are disproportionately well captured by lipizones, whereas other division pairs show weaker lipizone correspondence (Supplementary Figure 16d). This selectivity is consistent with the idea that lipid composition reflects specific circuit-level organizational principles rather than a generic byproduct of spatial proximity.*

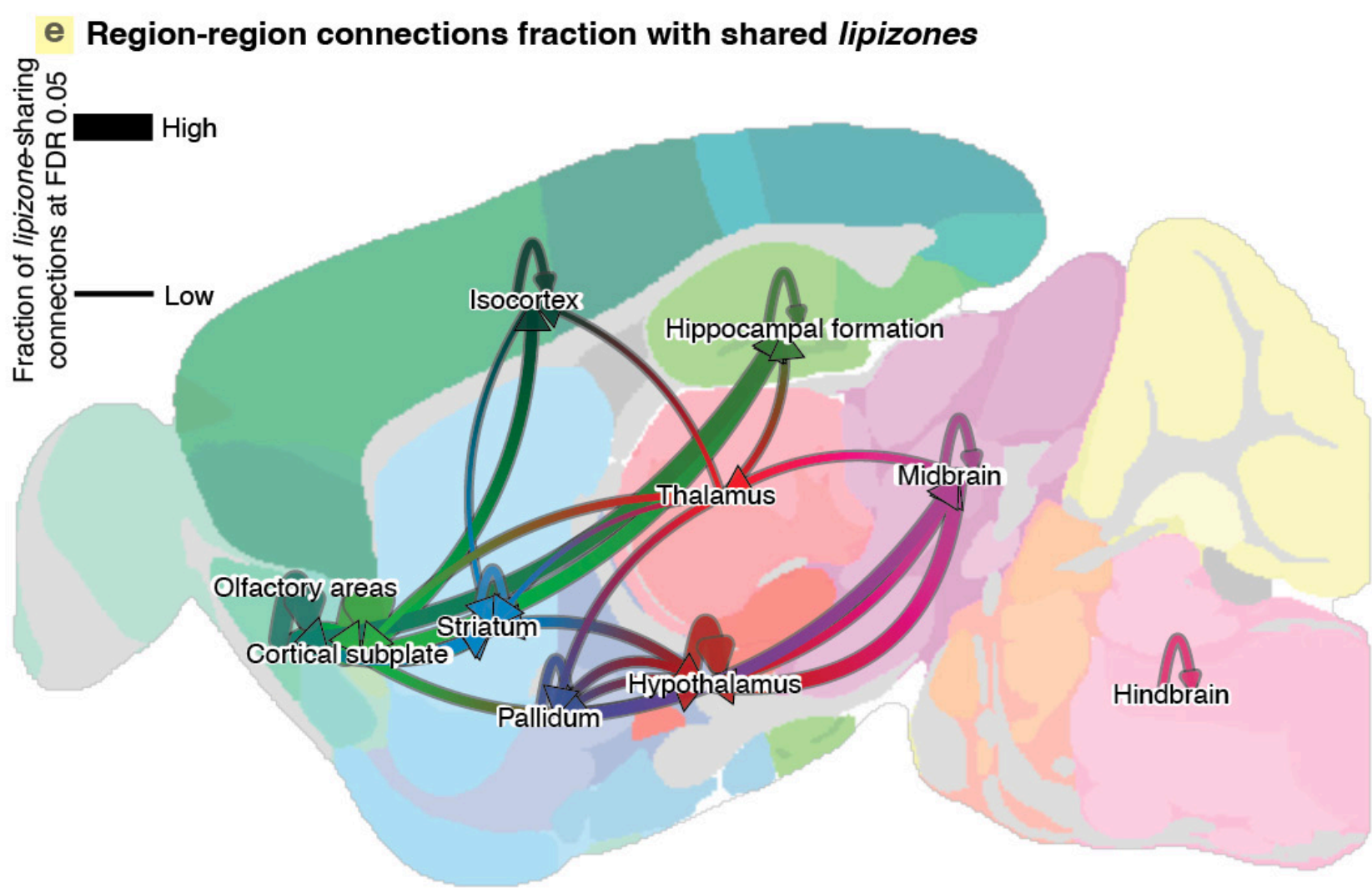


(Supplementary figure 16**e**) Spatial schematic of a sagittal mouse brain section, showing the fraction of *lipizone*-sharing connections at FDR 0.05 (curve thickness) stratified by brain division pairs. The coloring uses a gradient from the source region color to the target region color.

***Answering R2.2 and R2.3 combined**, we detail the new analysis in **S4P7**:*

> *"We found that connected brain regions shared one or more lipizones 2.89x more frequently than expected even when accounting for physical distance (26% vs 9%, structured permutation test accounting for brain region distances, p < 0.0001) (Figure 3j). This was valid for both permissive and large connectivity and lipizone overlap thresholds (Supplementary Figure 16c). Similarly, pairs of regions that share lipizones are 2.44x more likely to be connected (15.3% versus 6.3%; Fisher's exact p-value < 0.0001; valid across thresholds; Supplementary Figure 16d). We then classified all above-threshold connected region pairs of the mesoscale connectome (Supplementary Figure 17a; see Methods) as sharing or not sharing lipizones above chance (permutation test accounting for lipizone prevalence and inter-region distance), and we characterized the types of connections captured by lipizones. Midbrain to hypothalamus and pallidum to midbrain connections stand out (Supplementary Figure 16e)."*

*R2.4*

**I found the pregnancy figure to be almost completely impenetrable, beyond the assertion that "pregnancy changes the lipidome." Many panels convey little information—like panel b—or they convey information that is not supporting the key assertions that are returned to in the discussion, namely that the outer cortex and white matter are most affect. If the authors want to argue for these ideas specifically, they need clear, simple analyses that show that these regions are more perturbed than others.**

*We understand this criticism. The original figure attempted to present all pregnancy-related results in a compact format, but in doing so it obscured the central analytical question: which brain regions are most perturbed, and what is the nature of that perturbation? We have entirely restructured the pregnancy figure and supplements around a top-down narrative that addresses this question directly, proceeding from broad ranking to regional characterization to orthogonal validation using three extra experimental methods. These support experiments*

*strengthen our results, showcase the use of the atlas in conjunction with other modalities, and suggest avenues for future mechanistic investigations. We walk through the new logic below, and refer to the full Section 7 of the manuscript for details.*

*Step 1. **Ranking regions by overall perturbation.** We first quantify lipidomic change across all brain regions and lipizone supertypes, ranking them by the number of significantly altered lipids (Supplementary Figure 22a,c). Before any deeper characterization, this analysis establishes that the cortex, especially cortex layer 4 of somatosensory and visual areas, white matter tracts, and the ventricular system are the most affected regions, while deeper structures such as the hypothalamus and midbrain show comparatively modest changes.*

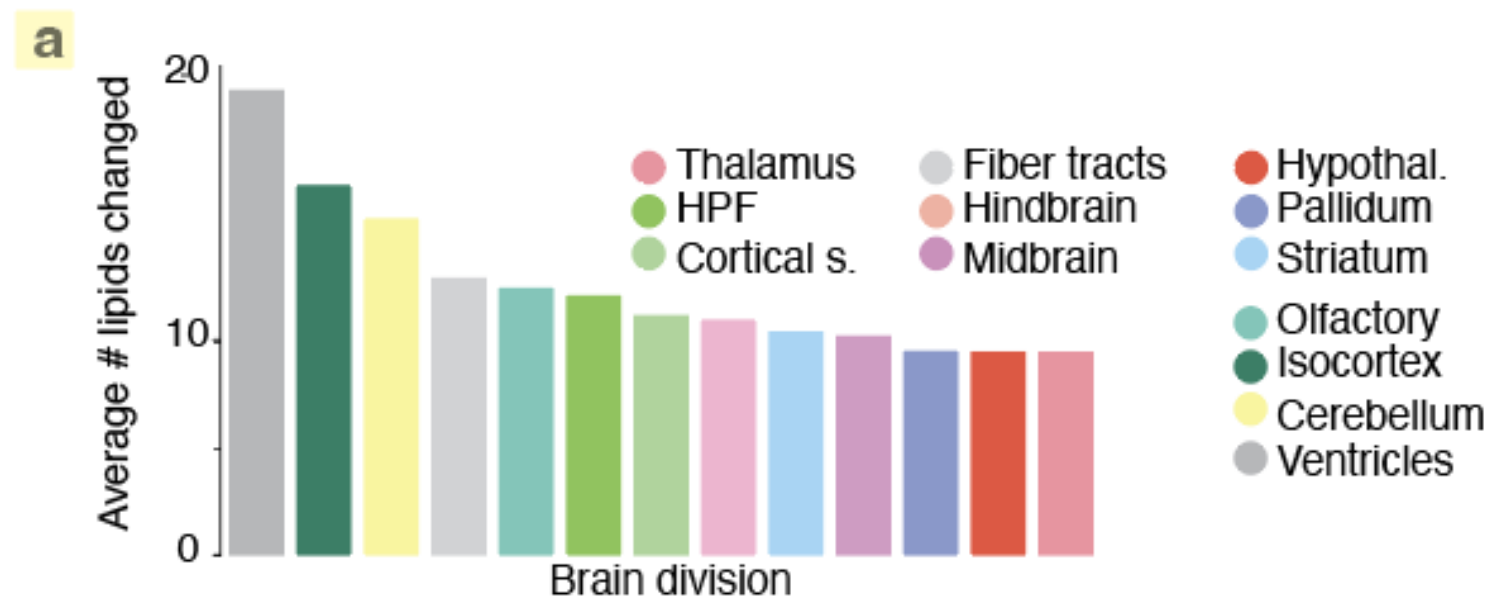


(Supplementary Figure 22**a**) Barplot of the average number of lipids changed in pregnancy, subdivided and colored by Allen Brain division.

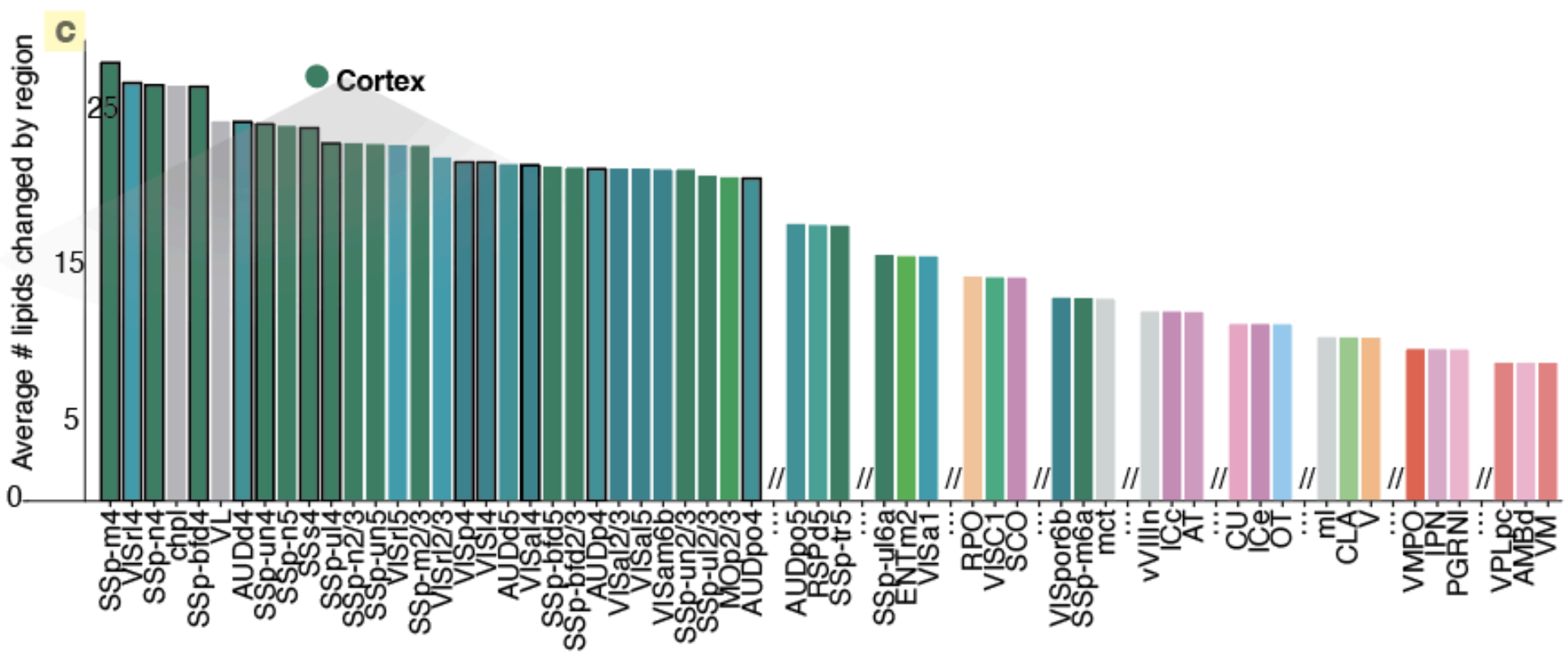


(Supplementary Figure 22**c**) Bar plot of the average number of lipids changed by pregnancy, subdivided and colored by Allen Brain fine-grained region. The x axis is broken multiple times to show representative regions from highly to minimally changed; the cortex is highlighted.

*Step 2. **Characterization of the most affected regions.** Having established which regions are most perturbed, we then ask what is changing, starting from a broad overview in Figure 6e.*

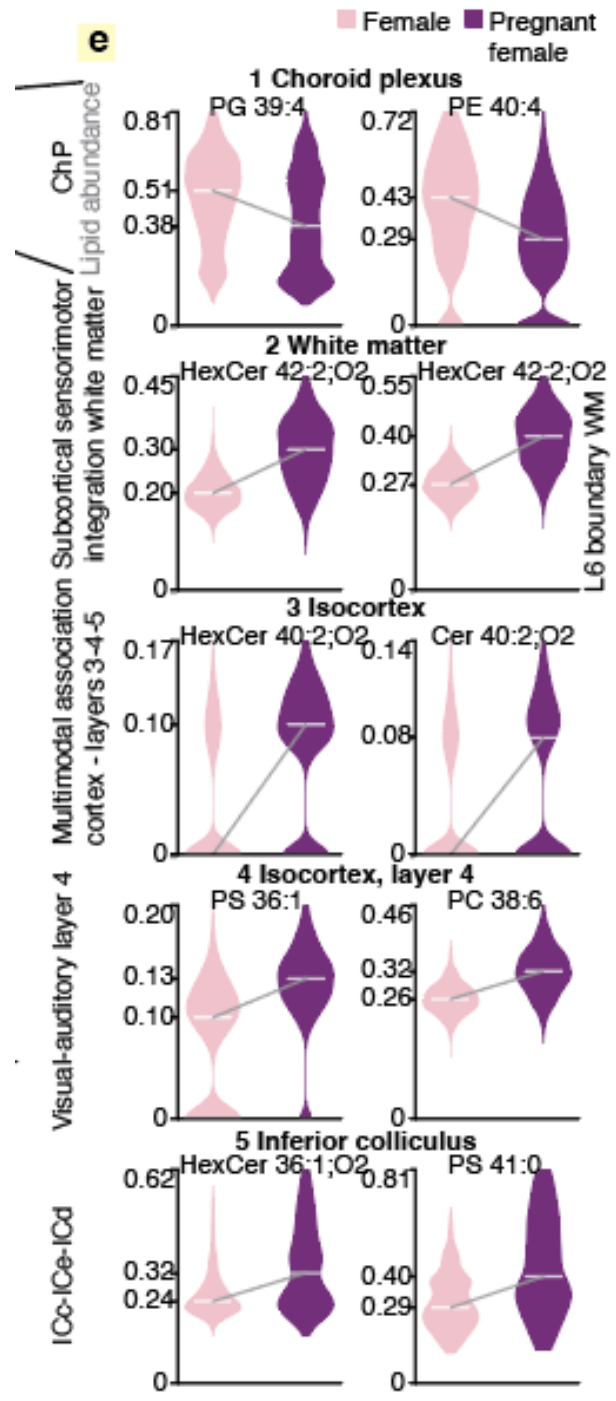


(Figure 6**e**) Violin plots of selected lipids from the co-varying blocks indicated in (d) for five selected *lipizones* (rows). White horizontal lines indicate the medians. .

*For the cortex, we show representative lipid distributions in non-pregnant versus pregnant females for major changes in the L4/5 intratelencephalic glutamatergic lipizone that is changed the most (Figure 6l, m).*

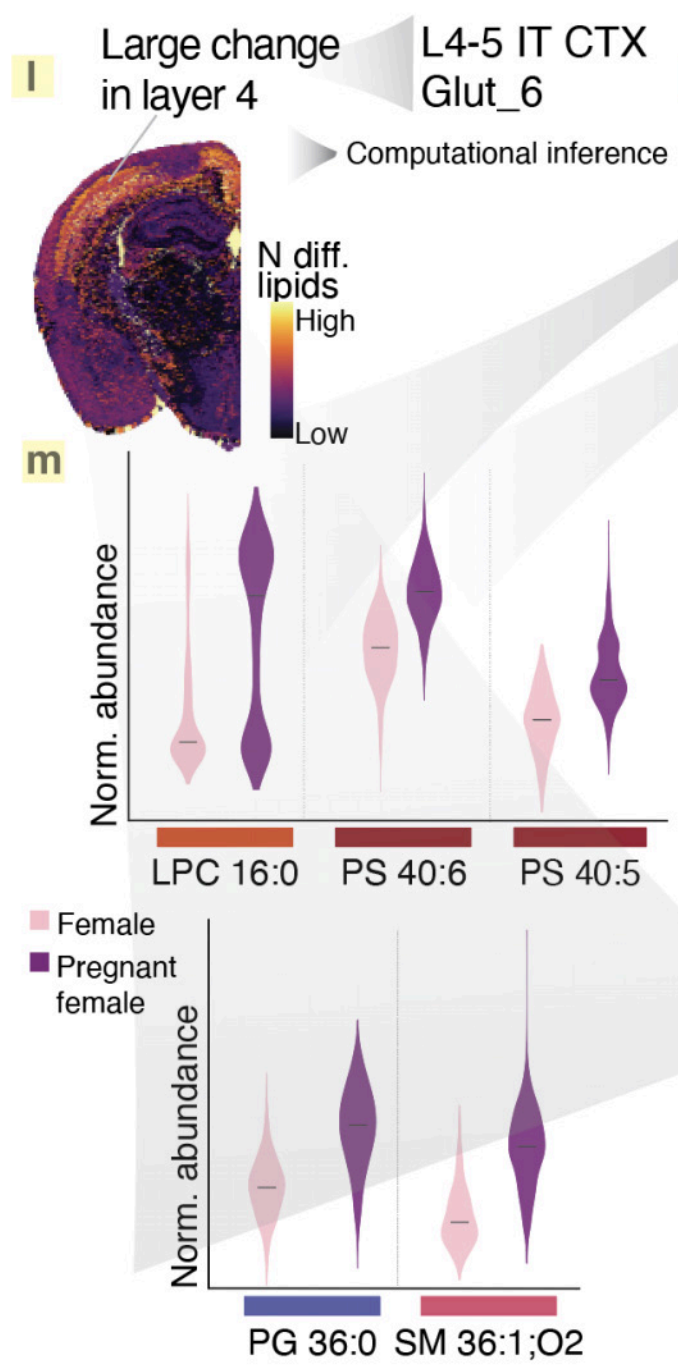


(Figure 6**l**) Representative spatial distribution plot where each pixel is colored by the number of lipids changed in pregnancy. (**m**) Violin plots showing the normalized abundance of 5 example lipids in non-pregnant (pink) and pregnant (purple) subjects.

*For the white matter, we document a pregnancy-associated increase in the myelin marker HexCer 42:2;O2 (validated by bulk lipidomics; see **R2.6**) and correlate it to an oligodendrocyte differentiation gene expression signature (Figure 6h-k).*

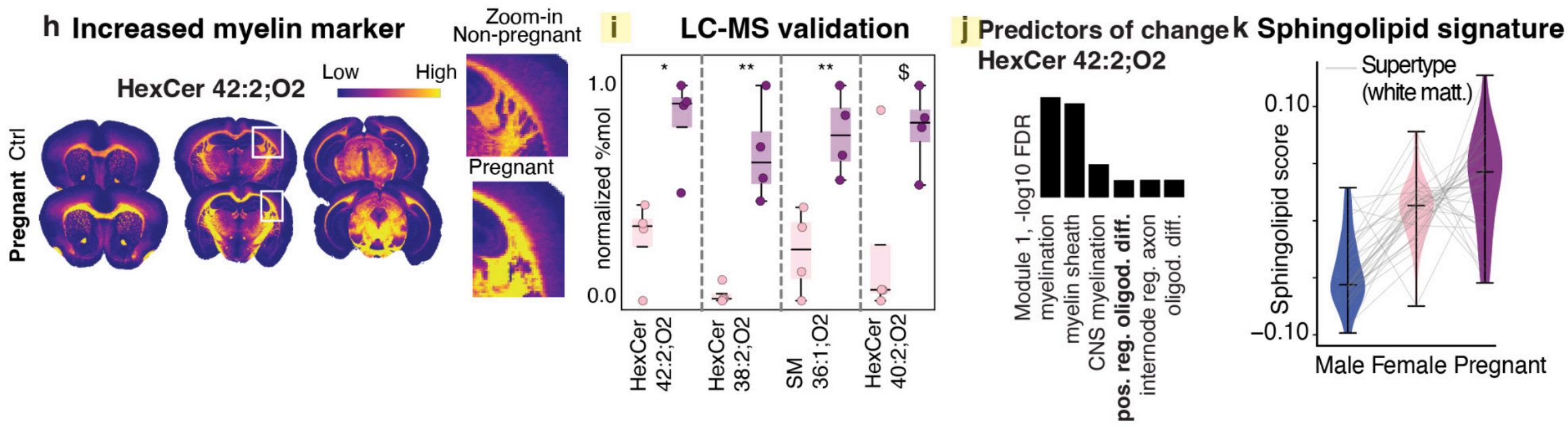


(Figure 6**h**) Spatial plot of representative sections for control and pregnant brains, colored by the myelin marker HexCer 42:2;O2. Zoom-in on the white matter in a control and a pregnant brain section, colored by HexCer 42:2;O2. (**i**) Boxplots of the (total-lipid normalized) %mol showing LC-MS abundances in control and pregnant females (dots are individual samples) for selected sphingolipids. Statistical significance was assessed by Welch's t-test (*p < 0.05, **p < 0.01, $p < 0.1). (**j**) Barplot showing the top gene ontology terms for the first module predictive of HexCer 42:2;O2 change in pregnancy. (**k**) Violin plot of the sphingolipid score in males, non-pregnant females, and pregnant females, across core white matter *lipizone* supertypes. Gray line segments connect the corresponding *lipizone* supertypes.

*Step 3.* ***Candidate upstream pathways.*** *We then ask what could explain that these regions are preferentially affected. Linking the pregnancy-associated lipid changes to gene expression, we find that they align with specific transcriptional modules (Figure 6f), that progesterone receptor is more expressed in regions undergoing lipid remodeling in pregnancy (Supplementary figure 22i, j), and that signaling through the glucocorticoid receptor pathway is predicted to be activated in the most affected cell type territories of cortical layer 4 (Figure 6n, Supplementary figure 22f).*

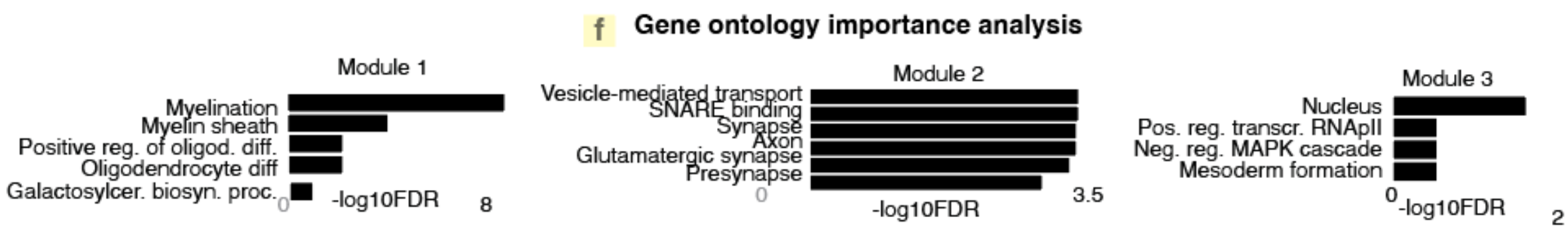


(Figure 6**f**) Barplots showing the significance level for top gene ontology terms (after FDR correction) for 3 gene expression modules predictive of pregnancy lipid changes.

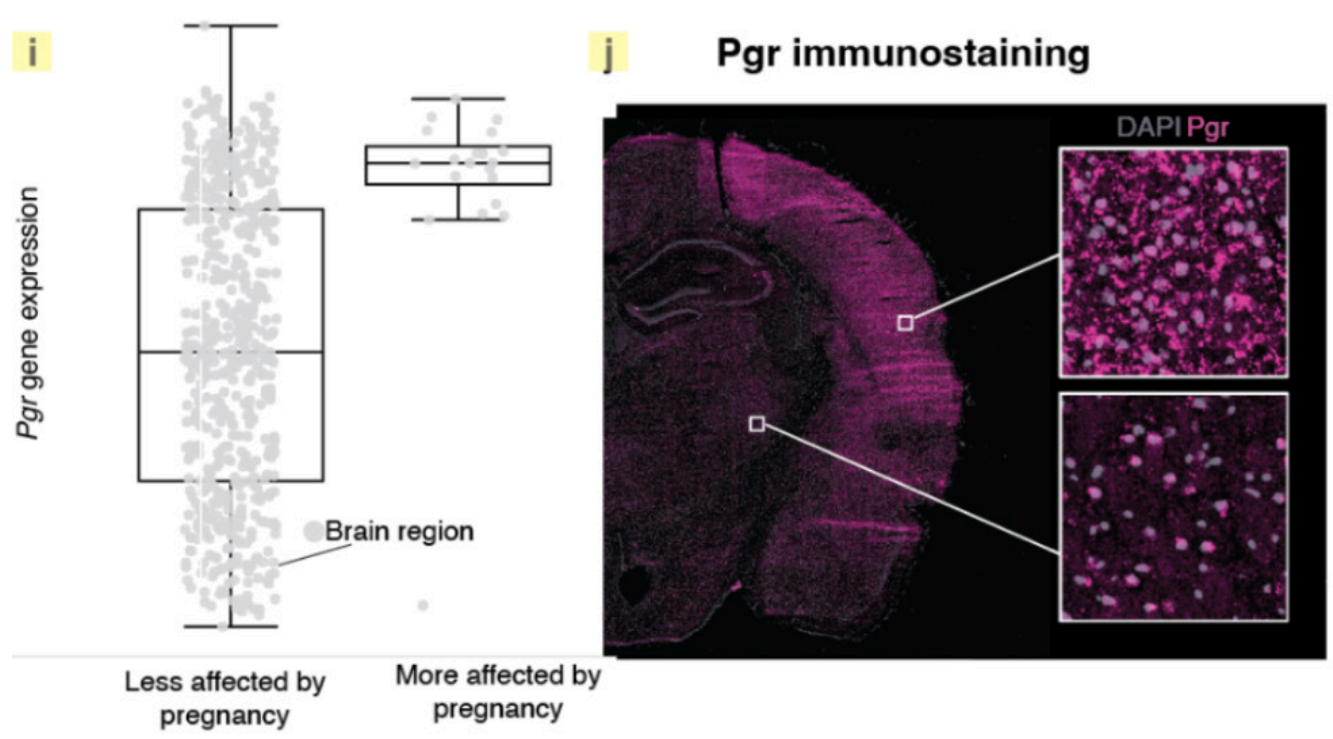


(Supplementary figure 22**i**) Box plots with overlaid strip plot of *Pgr* expression across Allen Brain Atlas regions, measured by MERFISH spatial transcriptomics, comparing regions more affected versus less affected by pregnancy as defined by

the spatial lipidomics data. (**j**) Immunostaining for Pgr protein in a representative female brain section, with insets showing cortex and thalamus.

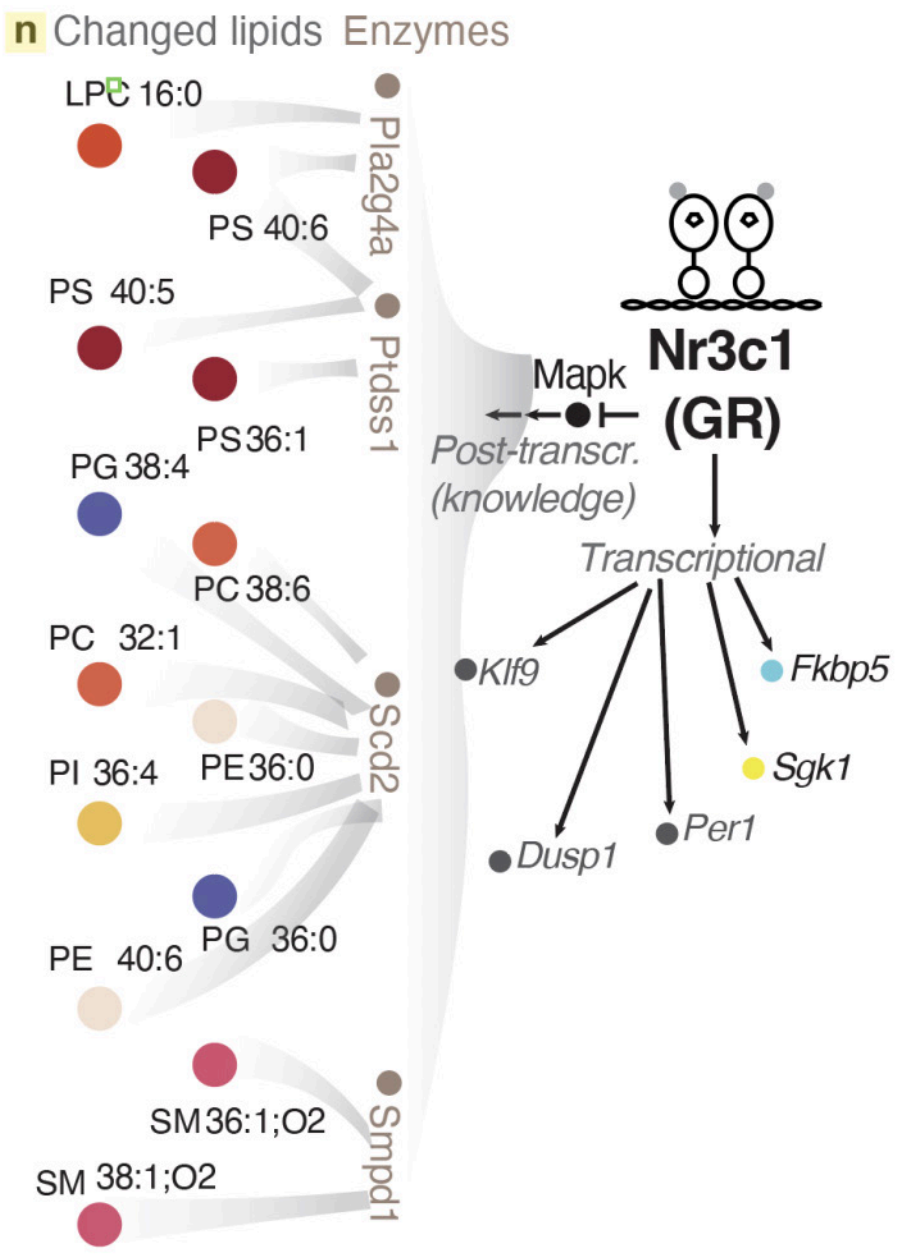


(Figure 6**n**) Schematic showing the results of the CORNETO analysis tracking the lipid changes back to a putative signaling origin, for the most affected *lipizone*, L4-5 IT CTX Glut.

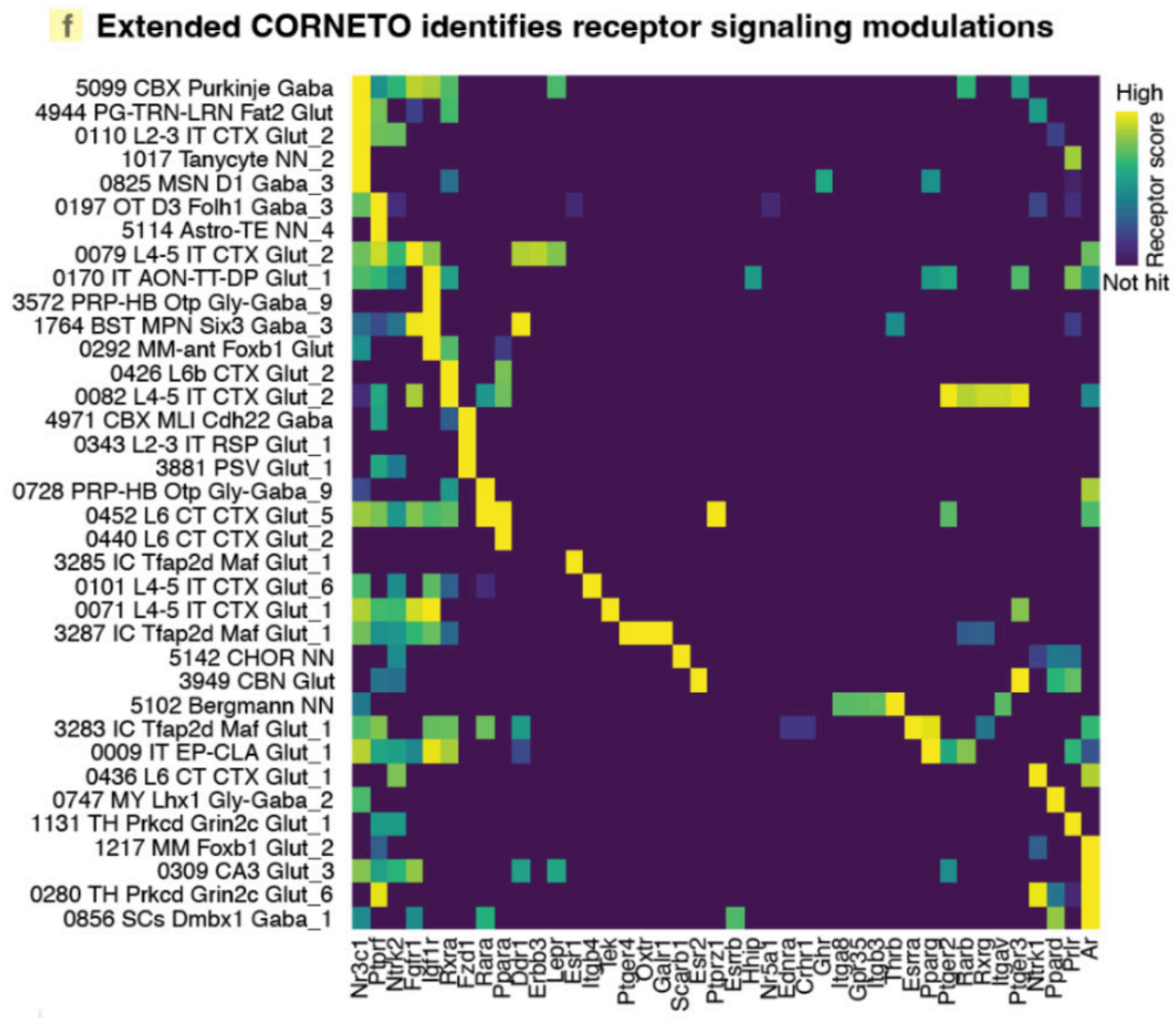


(Supplementary figure 22**f**) Heatmap showing, for each *lipizone* supertype (rows), the receptor pathways (columns) predicted by the extended CORNETO analysis to be activated in pregnancy in the corresponding cell type territory.

*Complementing this molecular perspective, regional connectivity structure independently predicts which areas are most perturbed (Supplementary figure 22d), highlighting circuits that are related to the spatial selectivity of the lipidomic changes observed.*

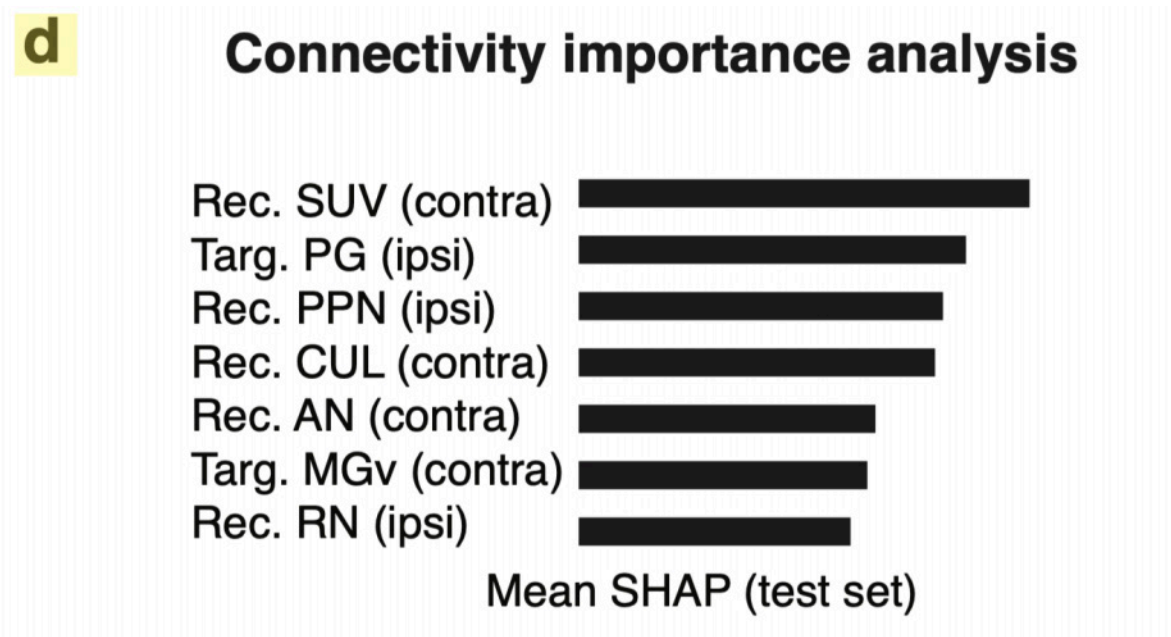


(Supplementary figure 22**d**) Bar plot of mean absolute SHAP feature importance extracted from a model predicting the number of lipids affected in pregnancy from the regional connectome.

***The pregnancy section (S7) has been largely rewritten*** *to accommodate the new corpus of in-depth analysis and support experiments (see also R2.5, R2.6). (Note that, differently from the other main-text edits we do not report section S7 here because it is an extensive part, instead, we kindly direct the reviewer to the revised manuscript) Future mechanistically-oriented studies are needed to determine the biology underlying the changes that we report.*

*R2.5*

**The sample size is also precariously low for these assertions, and incudling an individual pregnant replicate into the PCA analysis in fig 6e would really help bolster the claim that pregnancy is a huge contributor to variance, rather than batch effect**

*We note that the sample size may have appeared smaller than it is because the original figure did not make the per-individual structure visible. To detect pregnancy-related changes, each lipizone supertype in the original manuscript was represented across multiple sections per individual, for instance, the cortical L1–2/3 supertype was sampled in* ***11 sections*** *from* ***3 non-pregnant females*** *and* ***14 sections*** *from* ***3 pregnant females****, which compares favorably with current standards in spatial omics studies. An ANOVA across individuals also confirmed that pregnancy is the main source of variance ($p < 1e-4$).*

*That said, we have followed the suggestion to further strengthen the analysis. We have* ***added two additional pregnant female brains*** *(6 sections each) to increase statistical power, and updated the PCA (previously Figure 6e) to include individual animal, sex/pregnancy status, and rostrocaudal position. This analysis is reported in the main text:*

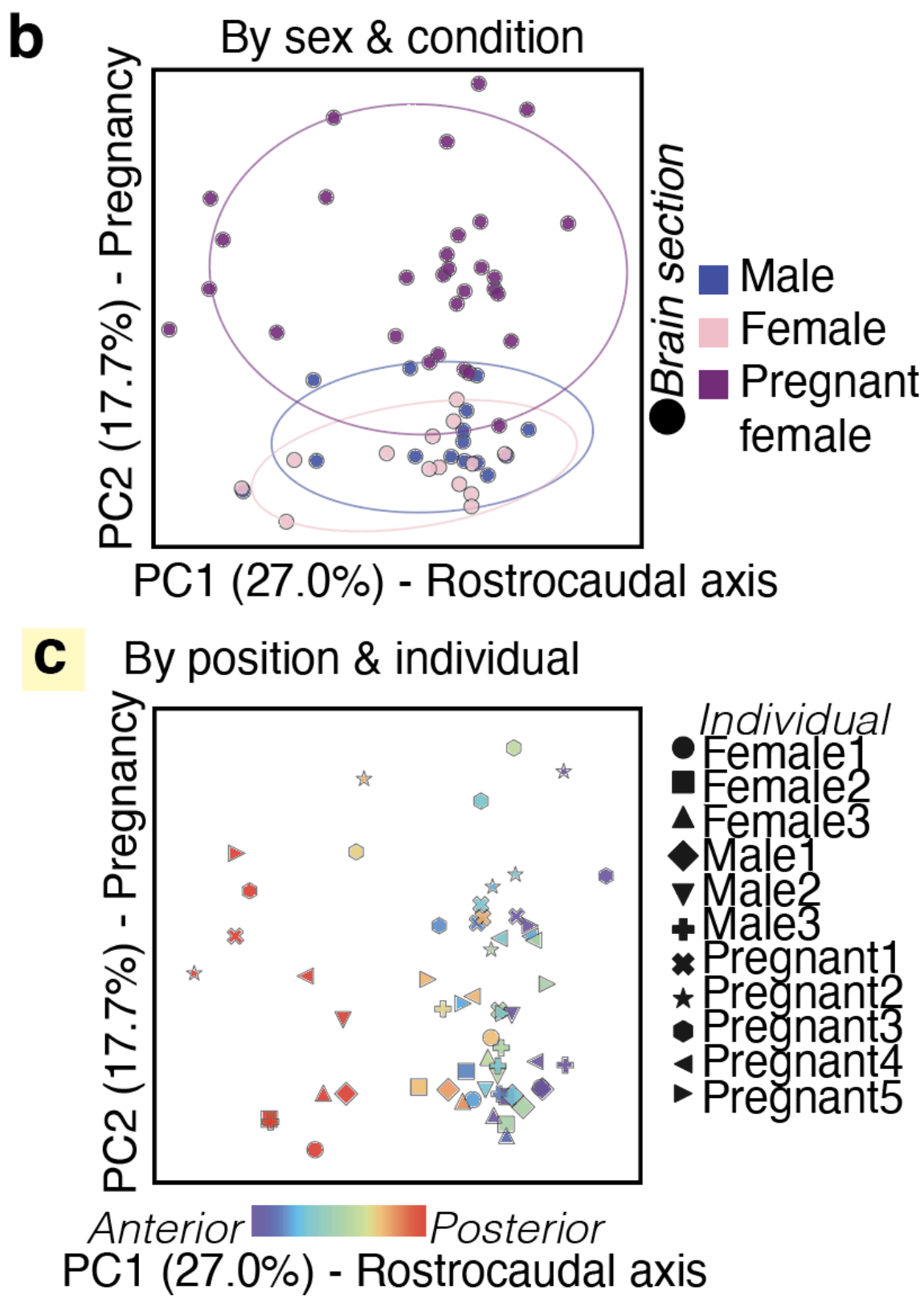


(Figure 6**b**) Scatter plot of brain sections in PCA space (one point is one section), color-coded for males (M), females (F), and pregnant females (P). Ellipses capture the data ranges, and axes (principal component coordinates) are labelled based on the covariate they correlated most with. PCA was conducted on the section-wise lipidomes; N=5 pregnant females (6 MALDI sections each) and N=3 for males and females (tot. 29 sections). (**c**) The same PCA scatterplot of (b), this time color-coded by the position along the rostrocaudal axis. The shape of the markers represent individual subjects.

***S7P3***: *"A first global analysis of the variance of this data revealed that pregnancy is associated with more extensive changes in brain membrane lipids than sex. Principal component analysis and donor-level analysis of variance (ANOVA; at the supertype level) further confirmed that the main axis of variation was strongly associated to the pregnancy covariate (Figure 6b, c)."*

*R2.6*

**It would also be helpful to independently validate a few of the strongest results orthogonally, such as showing white matter has higher HexCer 42:2 by doing microdissection and bulk lipidomics.**

*We followed this suggestion. We performed **dissection** of intact brains from an independent cohort (4 non-pregnant females and 4 pregnant females at E13.5) and analyzed the corpus callosum by bulk LC-MS: LC-MS **confirmed** the pregnancy-associated increase in **HexCer 42:2;O2** and other myelin-related sphingolipids in white matter (Figure 6i; Supplementary figure 23c).*

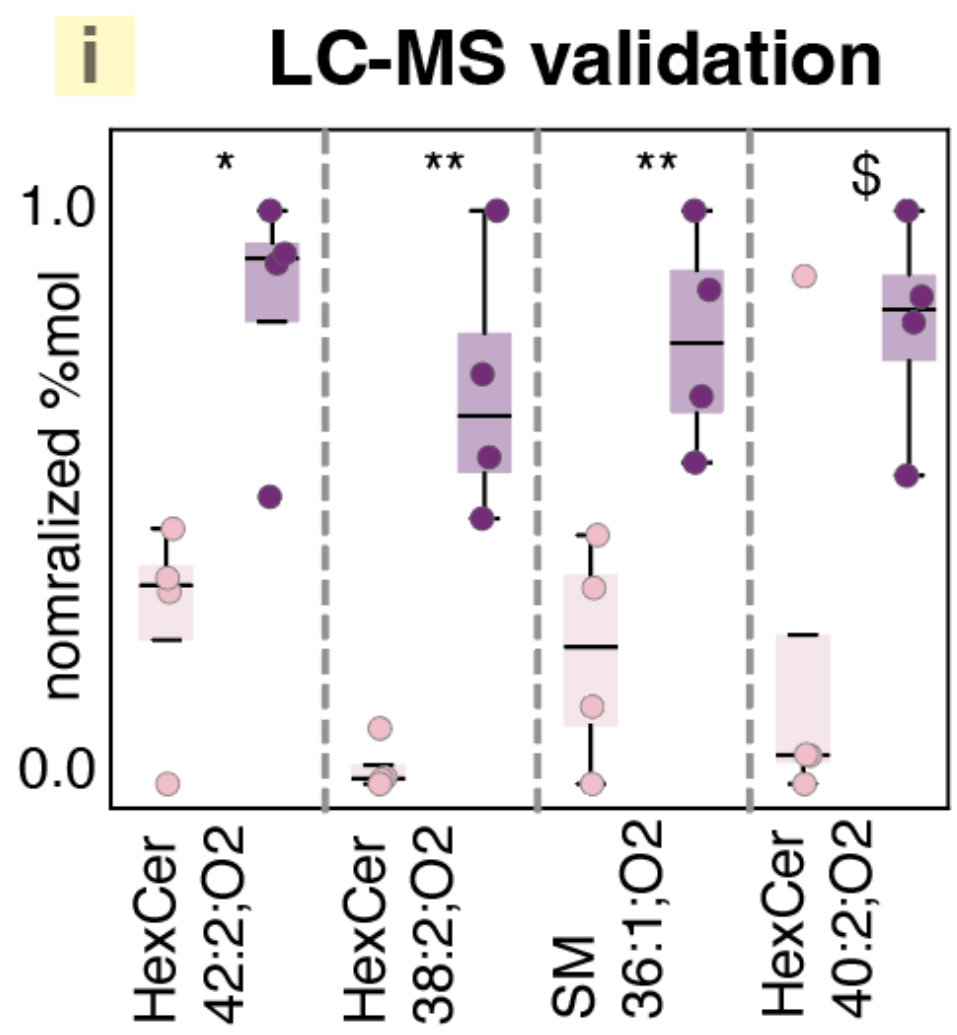


(Figure 6**i**) Boxplots of the (total-lipid normalized) %mol showing LC-MS abundances in control and pregnant females (dots are individual samples) for selected sphingolipids. Statistical significance was assessed by Welch's t-test (*p < 0.05, **p < 0.01, $p < 0.1).

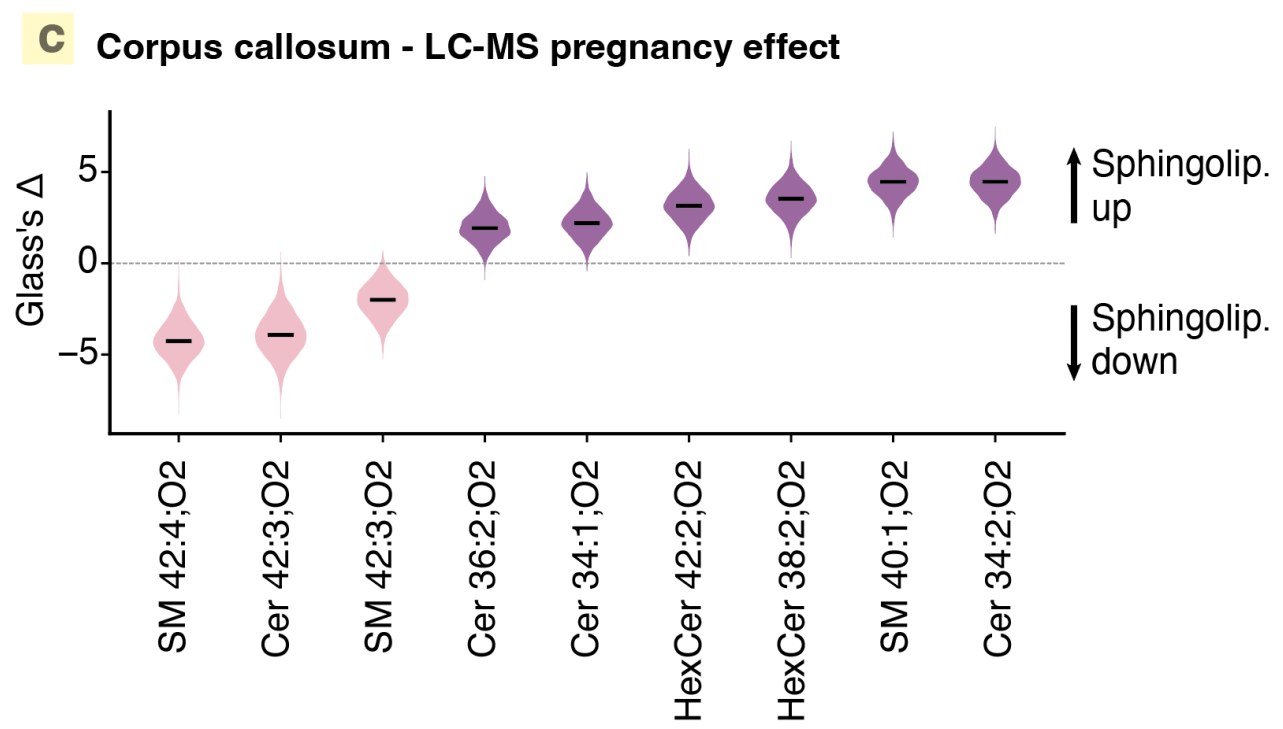


(Supplementary figure 23**c**) Violin plots of the Bayesian posterior of the pregnancy effect (in units of standard deviations from non-pregnant controls), showing LC-MS changes for selected sphingolipids.

*10 of the callosum-wide differential lipids were detected as differential also by MALDI in specific lipizones (Supplementary figure 23b); as a consistency check, the within-class correlation across lipids between our microdissection dataset, the published callosum LC-MS from Fitzner et al. and our MALDI dataset was high (Supplementary figure 23a; see R1.2 for methodological details).*

**Extended data on LC-MS validation of the discoveries with the pregnancy model**

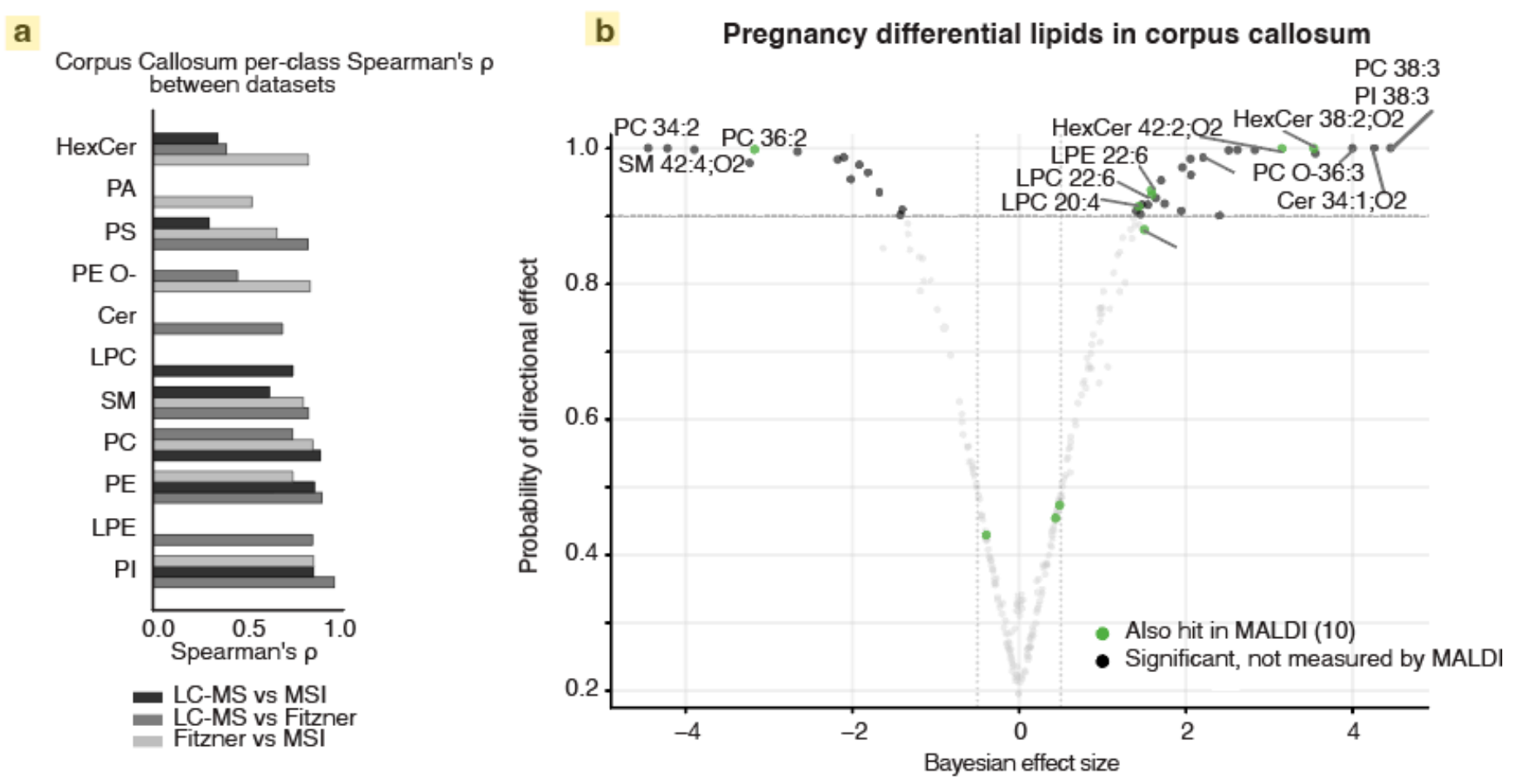


(Supplementary Figure 23**a**) Bar plots showing, for each lipid family, the Spearman correlation among individual lipids within a family across dataset pairs: microdissection-based bulk lipidomics, reference bulk lipidomics from Fitzner et al., and the spatial MSI dataset. (**b**) Volcano plot of bulk lipidomics data showing the effect size (x axis) and the significance (from the integrated Bayesian posterior); lipids with supporting evidence in callosal *lipizones* from the MALDI dataset are highlighted in green;grey dots represent lipids detected by LC-MS but not by MALDI.

*In the spirit of corroborating key results orthogonally as suggested by the referee, we furthermore validated the signaling prediction using* ***single-nucleus RNA-sequencing*** *and* ***immunofluorescence****, which we show in Figure 6 and Supplementary figure 23:*

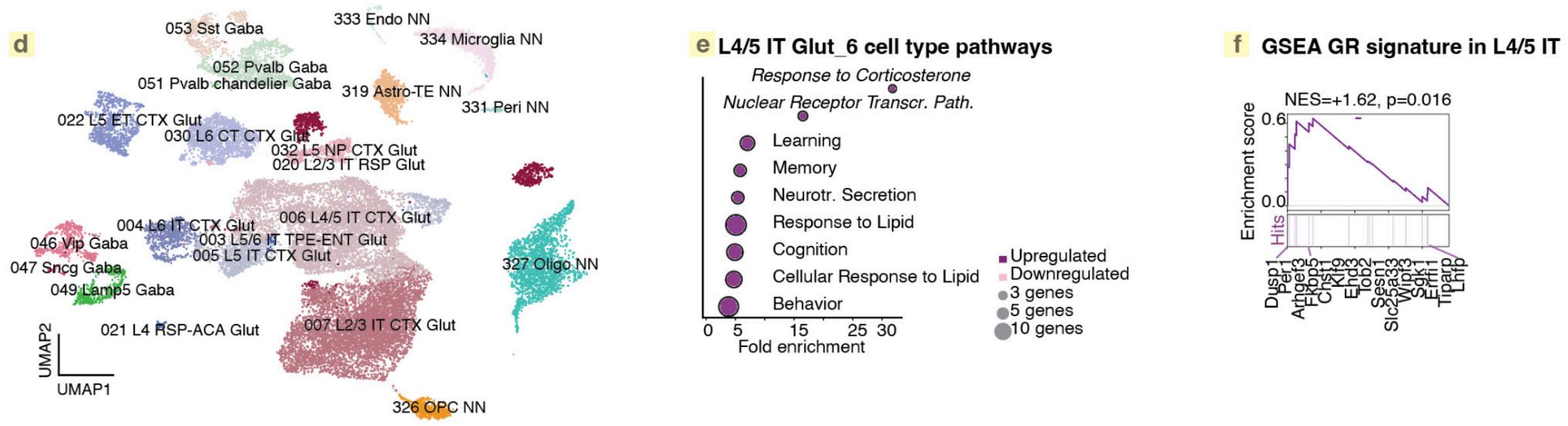


(Supplementary figure 23**d**) UMAP of the single-nucleus RNA-sequencing dataset (pregnancy and control samples), colored by Allen Brain Atlas cell types at the subclass level. (**e**) Over-representation analysis for the genes changed in the most affected cell type (L4/5 IT CTX Glut), showing fold enrichment for selected significant terms. Dot size is proportional to the number of differentially expressed genes contributing to that term, whereas color distinguishes enrichment versus depletion in pregnancy. The analysis is based on snRNA-seq. (**f**) Gene set enrichment analysis (GSEA) enrichment plot for the glucocorticoid receptor gene signature, based on snRNA-seq.

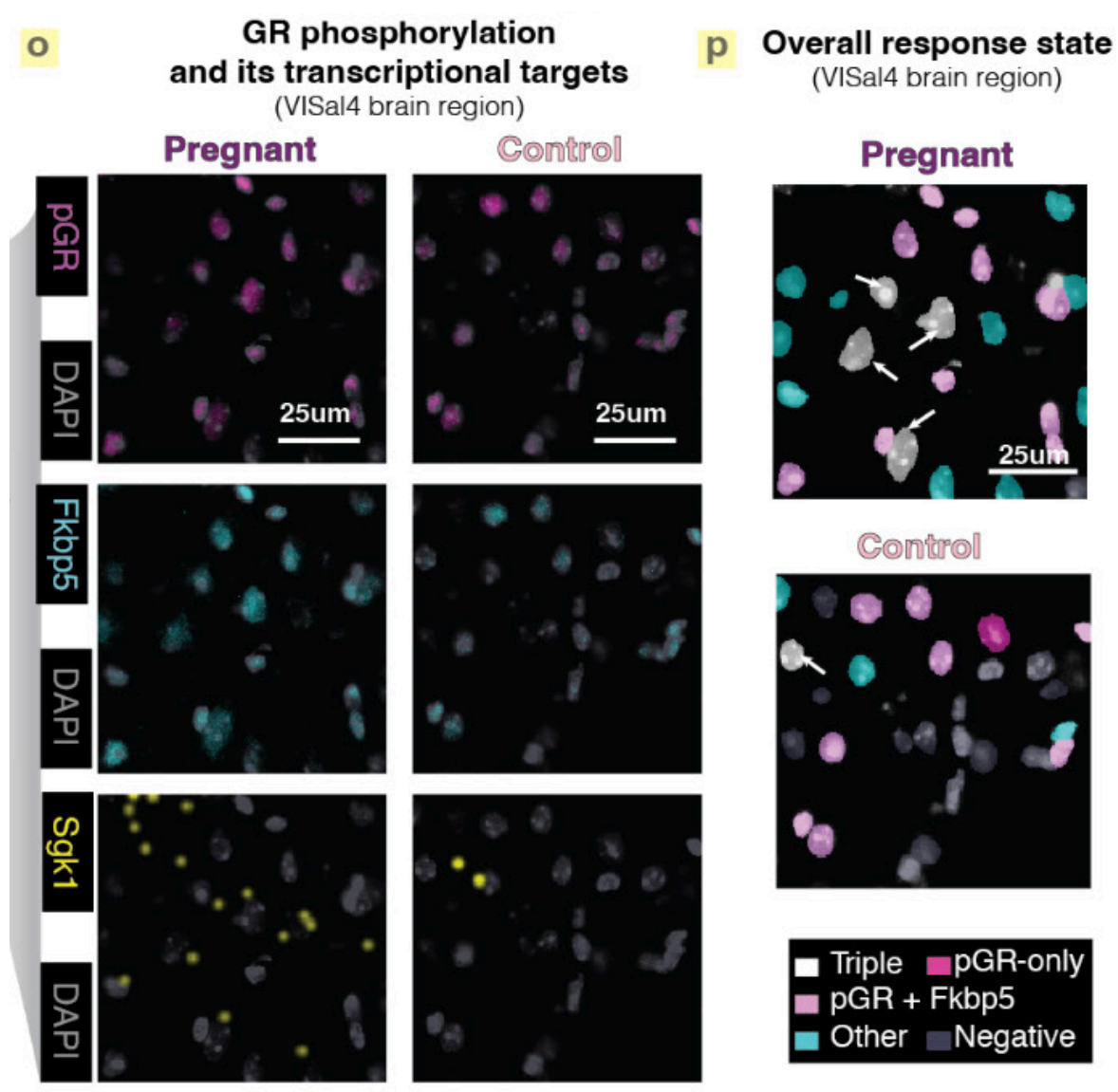


(Figure 6**o**) Immunostaining (pregnant vs. control) for glucocorticoid receptor phosphorylation and its transcriptional targets in cortical layer 4. DAPI is shown in light gray. (**p**) The same micrograph in (o) but with cells segmented and categorized based on their levels of GR phosphorylation, Fkbp5 intensity, and Sgk1 puncta density. Triple positives for GR phosphorylation and its two transcriptional targets are highlighted with arrows.

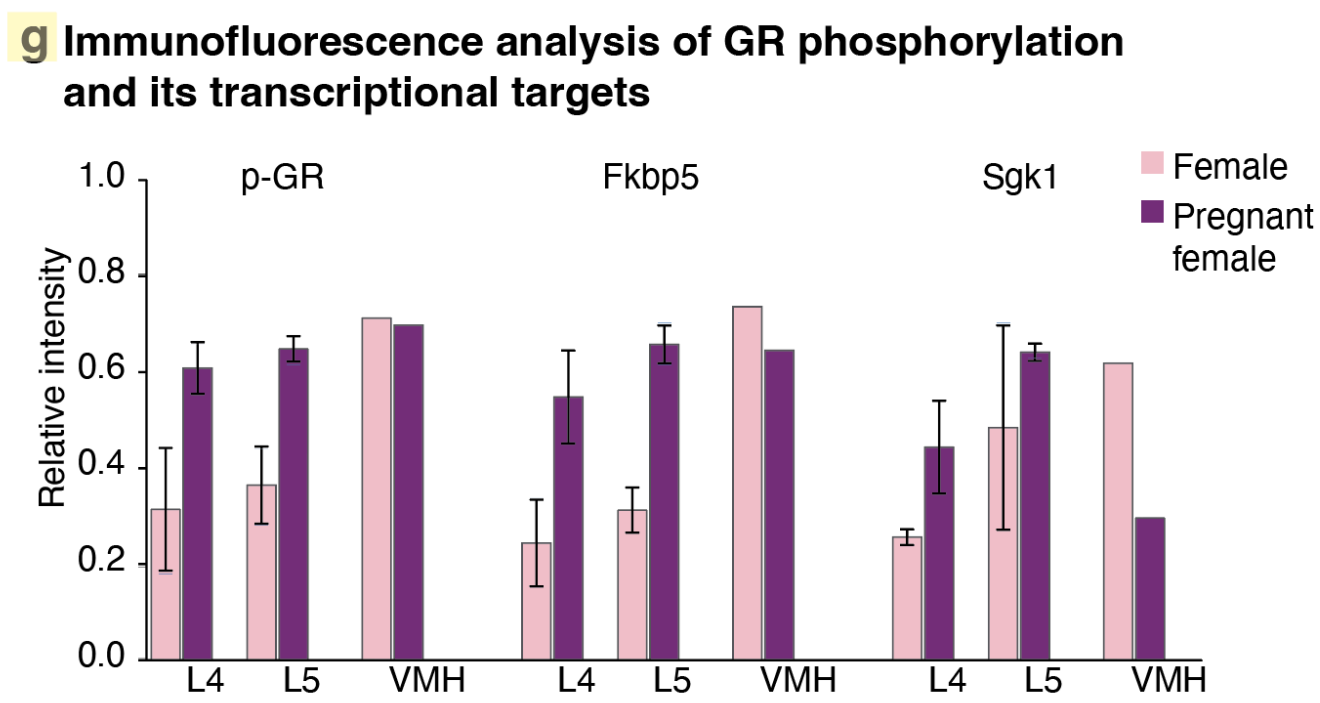


(Supplementary figure 23**g**) Bar plot showing the relative signal intensity for p-GR and Fkbp5 and relative average puncta per cell for Sgk1, in pregnant and control brain sections. Each bar represents the per-condition average; the standard error of the mean between two bregmas is shown. L4 = cortical layer 4, L5 = cortical layer 5, VMH = ventromedial nucleus of the hypothalamus.

*These results are detailed in the revised pregnancy section.*

*R2.7*

**The dataset is mentioned to be 109 sections, but Supp Fig 1b totals 118 sections.**

*We have now fixed Supplementary figure 1b to report **post-filtering numbers** as correctly suggested.*

*R2.8*

**Regarding the validation staining in figure 5, the citation 61 is to cholera toxin, which doesn't bind sphingomyelins.**

*We have now specified in the text that we also stained for **GM1 as a negative control**, which the citation 61 referred to along with citations 62-63 which were for SM toxin stainings.*

***S6P4***: *"Staining with toxins that specifically bind to SMs (and GM1 as a negative control) validated SMs as ventricular markers (Figure 5j)."*

*R2.9*

**I am not sure that the assertion in the text about Supp Fig 7g—that these finer-grained Allen anatomical regions nicely overlap lipizones—is supported by the figure itself, which is a very hard-to-read, dense confusion matrix.**

*We have now* ***substituted the heatmap*** *with the plots described in comment R2.10. The assertion is now better contextualized in the text. For clarity, our original statement was based on the implicit consideration that some Allen regions were historically defined following* ***functional considerations*** *(e.g., fine arealization of the cortex), which typically do not align with molecular differences(Hayden et al. 2026) (this is also what spatial omics studies to date consistently show). We fully agree with the referee that the suggested visualization is clearer than the original heatmap.*

*R2.10*

**For characterization of the anatomical correlation with the lipizones, it might be helpful to generate a cumulative distribution plot, showing the number of unique anatomical regions (at a given resolution level) needed to reach some reasonable percentage of the total lipizone area (say, 90% or 95%).**

*We have* ***constructed the cumulative distribution*** *curves indicated by the reviewer, one for each lipizone (e.g., if a lipizone has 30% of its pixels in region A, 50% in region B, and 20% in C, the curve will be: (1, 50%), (2, 80%), (3, 100%)). The mean trend and variance across lipizones are displayed, and the number of regions corresponding to reaching 75% of lipizone area are now reported. We found that lipizones pertaining to the forebrain, midbrain, and hindbrain are as anatomically-localized as cell types. Specifically, within each division, the mean number of unique anatomical regions covering 75% of a lipizone's total area for non-connectivity lipizones were comparable to those of cell types: in the forebrain, 3.5 vs 3.8; midbrain: 4.2 vs 3.6; hindbrain: 5.1 vs 4.7; thalamus+hypothalamus: 4.4 vs 5.6 (Mann-Whitney U, not significant in any division after BH-FDR correction at α = 0.05). We also report a more naive analysis that does not account for brain divisions and therefore captures processes spanning multiple regions, including connectivity streams; there, the numbers of unique anatomical regions to cover 75% of lipizone area are expectedly larger (8.3 vs 4.4 for cell types) consistent with our observations on connectivity-related lipizones. In light of their importance, this analysis is now shown in main Figure 3k and Supplementary figures 9g, h, i.*

> ***S4P8:*** *"Lipizones were as anatomically localised as cell types. For each lipizone and each cell type, we quantified the number of unique anatomical regions required to reach 75% coverage. The resulting distributions were not statistically significantly different between lipizones and cell types in any brain division (Mann-Whitney U; BH-FDR corrected at 0.05): forebrain (3.5 vs. 3.8), midbrain (4.2 vs. 3.6), hindbrain (5.1 vs. 4.7), and thalamus/hypothalamus (4.4 vs. 5.6) (Figure 3k; Supplementary figures 9g, h, i; see Methods). We also performed a broader analysis without separating brain divisions, which captures processes that span multiple regions. In this global view, lipizones predictably spanned more anatomical regions than cell*

*types (8.3 vs. 4.4 regions to cover 75% of area; Figure 3k). This is consistent with our finding that lipizones can track long-range connectivity across the brain."*

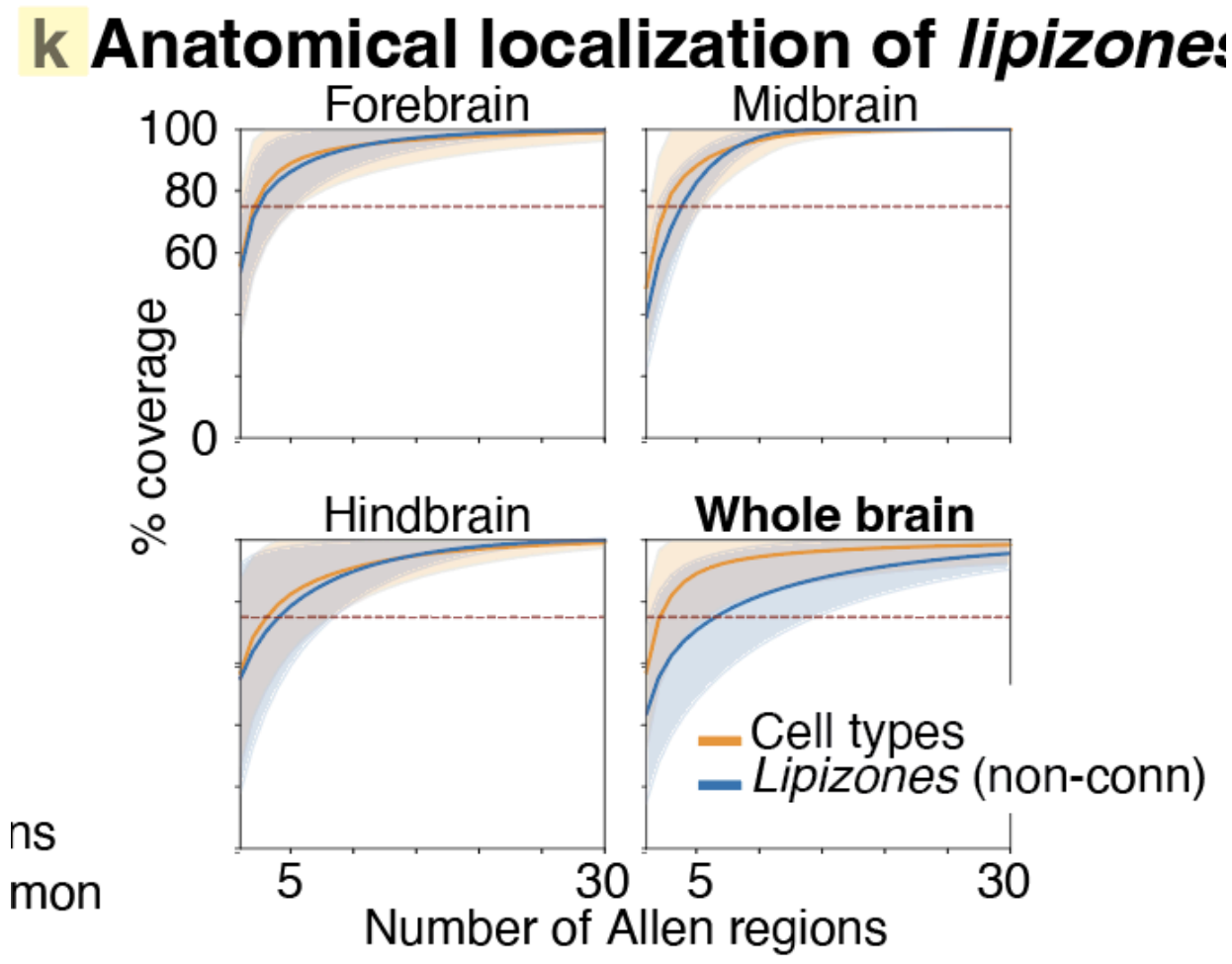


(Figure 3**k**) Cumulative distributions across *lipizones* (and cell types) representing the percentage of area of a *lipizone* (or cell type) covered when accounting for a given number of Allen regions. The same plot is repeated at the whole-brain level and for forebrain/midbrain/hindbrain separately. The *lipizones* shown are those not related to connectivity; see *Supplementary figure 9g* for the full analysis including connectivity-related *lipizones*.

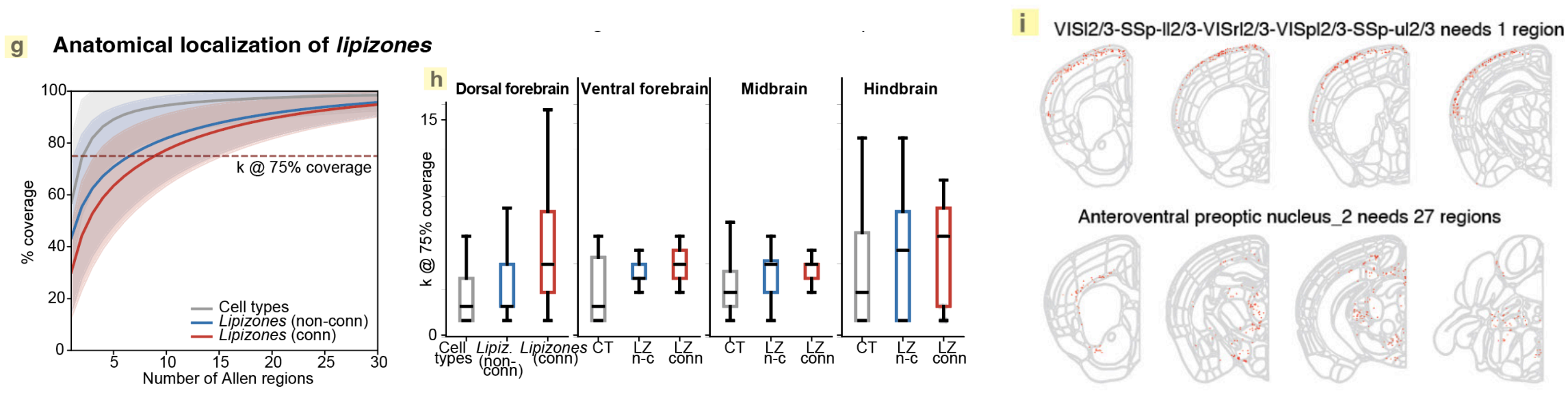


(Supplementary Figure 9**g**) Cumulative distribution of the number of anatomical regions needed to cover increasing fractions of *lipizone* or cell type area, averaged across *lipizones* (subdivided by whether they are connectivity-related or not) and Allen Brain Atlas cell supertypes. The line represents the mean; the shaded area encompasses ± 1 s.d. The dashed dark red line marks 75% area coverage. (**h**) Box plots showing, for each brain division, the number of ABA regions needed to cover 75% of *lipizone* (or cell type) area. (**i**) Examples of *lipizones* at the extremes of the cumulative distribution saturation: a lipizone contained within a single Allen region and a lipizone spanning 27 regions to cover 75% of its area.

## Referee #3 (Remarks to the Author):

**The spatial organization of lipids has been underexplored within in intact tissue. This study presents a high-resolution lipid brain atlas of the adult mouse brain, created using MALDI-MSI and computational methods, mapping lipid composition across sexes and during pregnancy. The authors identified over 500 distinct biochemical regions, termed "lipizones," which align with anatomical structures, cell types, and neural connectivity. Lipizones reveal a fine-grained biochemical architecture that correlates with functional brain anatomy, including both cell bodies and distant axon terminals, something not captured by spatial transcriptomics. Key discoveries include a new axis of oligodendrocyte heterogeneity in white matter and distinct biochemical zonation in the choroid plexus and ventricular walls. Furthermore, the lipidomic architecture demonstrates adaptability during pregnancy, the white matter becomes metabolically activated and the outer cortex undergoes structural reorganization. Overall, this atlas establishes a comprehensive spatially resolved lipid map of the mouse brain.**

*We thank the reviewer for the detailed and constructive critique.*

*R3.1*

**There is a degree of overinterpretation and overselling of the data. The authors claim to have identified "lipizones" as fundamental units of lipid composition. However, this appears to be an artificial classification largely driven by the methodology employed. A comparable categorization could likely be derived using other omics data, suggesting the approach may not uniquely reveal such structures.**

*We have revised the manuscript to* ***avoid referring*** *to lipizones as "fundamental units", and have further strengthened both the robustness and the interpretability of the lipizone framework. The discovery aspect of lipizones does not lie in them being unique descriptors of brain anatomy, but in showing that fine-grained brain architecture can be captured directly from lipid distributions, and that this parcellation constitutes a useful model for studying the brain lipidome.*

***1) Robustness to algorithmic choices****. This finding is independent of the specific computational method: to reinforce the validity of the clustering, we have now demonstrated that results are* ***consistent*** *across alternative clustering methods, feature subsets, and datasets, and documented cross-brain reproducibility through spatially coherent label transfer (detailed in* ***R2.1****).*

***2) Model comparison****. Lipizones are closer than anatomical regions to the level of granularity at which metabolic regulation occurs. We have now shown this by comparing the lipizone and anatomical models in their ability to capture the biological changes in pregnancy. We retrained our Bayesian model using Allen Brain anatomical regions rather than lipizones as atomic units, and compared how well each captures the lipidomic variance in the pregnancy dataset (which was not seen during lipizone training, avoiding double dipping). The lipizone model has higher explanatory power despite having fewer degrees of freedom (222 supertypes vs 397 anatomical categories; Supplementary Figure 21I):*

I

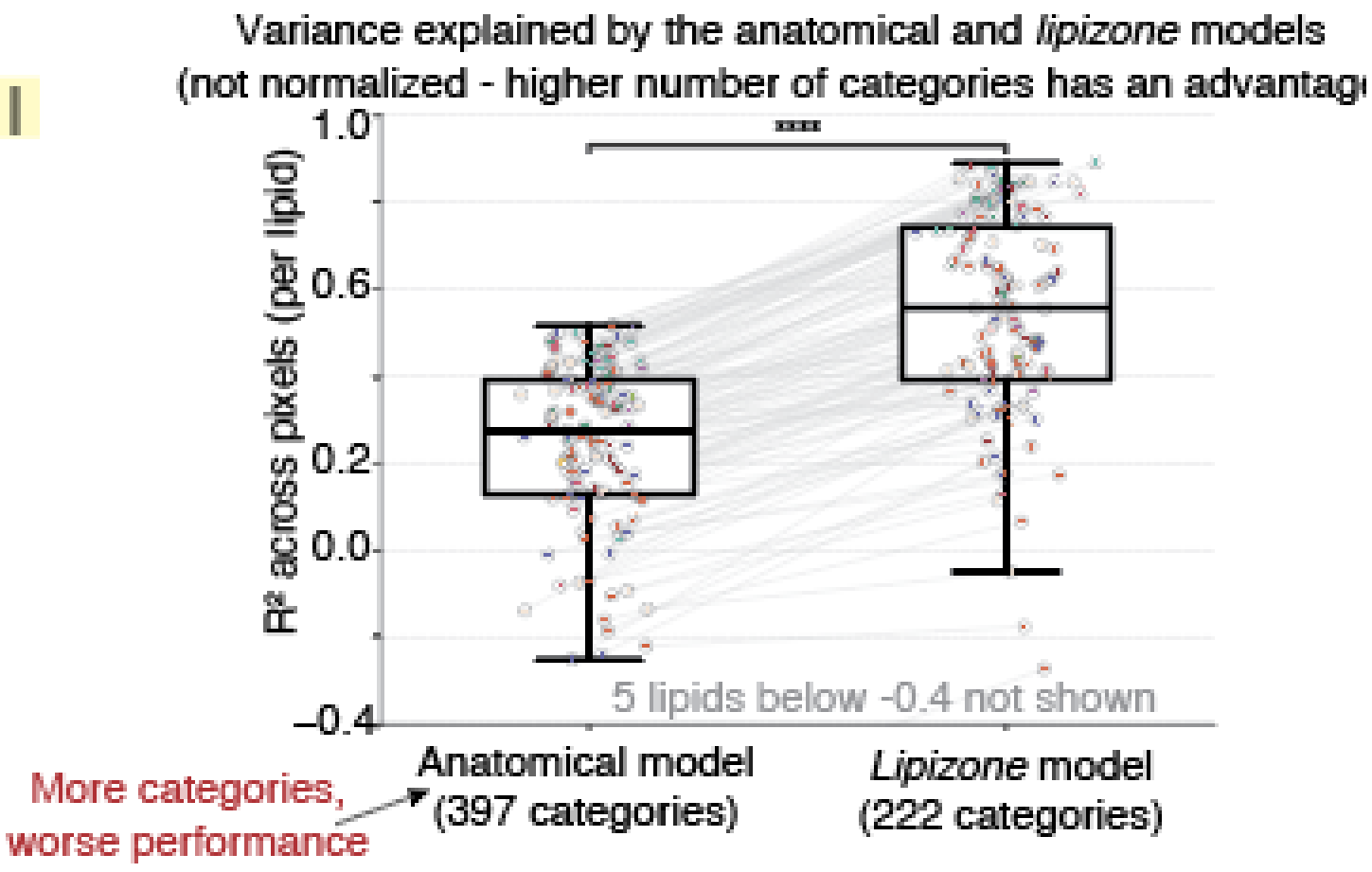


(Supplementary figure 21**I**) Box plots with overlaid strip plots showing the fit quality ($R^2$ across pixels) for individual lipids under two models: an anatomical model based on 397 Allen Brain Atlas acronyms and the *lipizone* model based on 222 supertypes. Each dot represents one lipid, coloured by lipid class; line segments connect the same lipid across the two models. Five lipids with $R^2 < -0.4$ under both models fall outside the displayed y-axis range.

*This analysis is now reported in paragraph S7P2.*

> ***S7P2****: "To assess whether lipizones offer a genuine advantage over conventional parcellations, we trained an analogous model using anatomical regions instead of lipizones. Despite nearly twice as many categories, the anatomical model yielded significantly worse fits (Wilcoxon test, p < 0.0001; Supplementary Figure 21I)."*

***3) Comparison with the transcriptomic hierarchy.*** *Despite the good correspondence between terminal lipizones and cell type territories, the hierarchical structures discovered by transcriptomics and lipidomics diverge. The transcriptomic hierarchy of cell types is known to be organized by early splits separating neurons from glia, followed by developmentally driven divisions and neurotransmitter identity; the lipidomic hierarchy does not recapitulate any of these axes. For example, the first major lipidomic splits do not recover classical transcriptomic divisions: the PAL-sAMY-TH-HY-MB-HB neuronal territory overlaps extensively with myelin-rich regions, and cerebellar and pallium glutamatergic populations fall within the same lipid-defined partition (Supplementary Figure 15a, b). At intermediate levels, the divergence is equally apparent. For example, within the grey matter branch, a major split is driven by phosphatidylserines on one side and hexosylceramides on the other. This places hypothalamic mammillary glutamatergic neurons alongside cortical and cerebellar populations, while separating them from other hypothalamic types that instead cluster with midbrain neurons and cortical layer 6; this arrangement cuts across prosomeric origin and cannot be explained by neurotransmitter identity.*
*Correspondence between the two modalities emerges only at the terminal level, where individual lipizones match cell type territories. In short, transcriptomics and lipidomics agree on the leaves but disagree on the branches.*

*Our interpretation is as follows: because cell types differ in their gene expression programs, and therefore in their regulatory and homeostatic machinery, each has the freedom to maintain a distinct lipid composition, and our data suggest that they do. Yet the way these distinct lipidomes are organized across the brain does not mirror the transcriptomic*

*hierarchy; the architecture of the lipidome follows its own principles. We have dedicated a new paragraph to these considerations in the main text (S4P3).*

> ***S4P3***: *"Previous studies described cell type transcriptome hierarchy as organized by early splits separating neurons from glia, followed by developmentally driven divisions, and finally by neurotransmitter identity; the lipidomic hierarchy does not recapitulate these axes (Supplementary Figure 15). Indeed, cell types that are transcriptomically distant are grouped together by their shared membrane composition, confirming that the two hierarchies impose fundamentally different partitions on the same tissue (Supplementary Figure 15b). For example, within the gray matter branch, a major split is driven by phosphatidylserines (PS 42:9, PS 36:2) on one side and hexosylceramides (HexCer 42:2;O2, HexCer 42:1;O2) on the other. This places hypothalamic mammillary glutamatergic neurons alongside cortical and cerebellar populations, while separating them from other hypothalamic types that instead cluster with midbrain neurons and cortical layer 6 (Supplementary Figure 15a, also visible in Figure 2b). This arrangement cuts across prosomeric origin and is not explained by neurotransmitter identity alone. We interpret both the divergence of the hierarchies and the matching at the terminal level as consistent with a single picture: the lipidome does not follow the same organizing principles as gene expression, yet it is at the level of individual cell types, the finest entity with unique gene regulation programs, that distinct lipidomic profiles are modulated."*

*This result is shown as Supplementary figure 15a, b:*

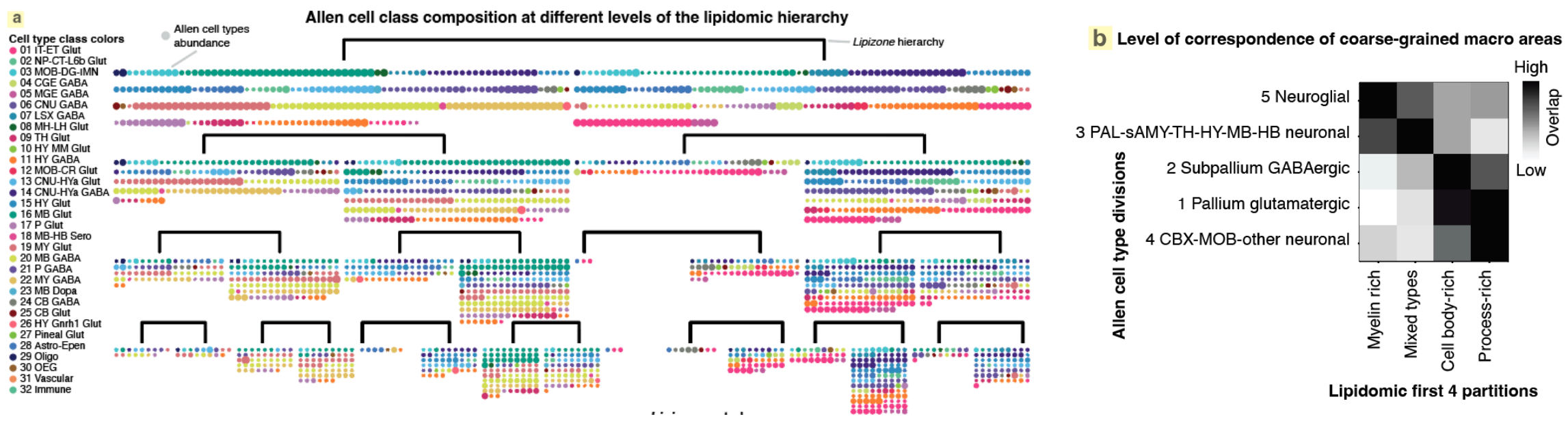


**Supplementary Figure 15. Comparison of lipidomic and transcriptomic brain hierarchies, and *lipizone*-informed dissection tables.** (**a**) Dendrogram of the *lipizone* binary hierarchy (first 4 splits) with sorted dots for Allen supertype cell types, colored by the Allen cell type class (see legend). The dot size is proportional to their abundance, and dots are assigned to the side where they colocalize the most. (**b**) Heatmap of the spatial overlap between the five Allen cell type divisions (rows) and the first four lipidomic partitions (columns), showing the degree of correspondence between coarse-grained transcriptomic and lipidomic brain parcellations.

***4) Practical implications.*** The observation of a mismatch between the two hierarchies has direct experimental consequences: the 8 major lipizone classes are so different yet not separated by classical anatomical divisions that they are likely to be mixed by traditional dissections, reducing the statistical power of future experiments and even introducing spurious differential results. To make this finding directly useful for future experimental design, we designed a pipeline that constructs a tailored parcellation of the CCFv3 by assigning each Allen anatomical region a lipizone class label by maximum overlap. This pipeline can simulate 2D slices at any orientation and bregma position to mimic practical dissections. The resulting tables indicate, for each lipizone class, which brain regions should be sampled together to minimize lipizone class intermixing. We release these tables and the

dissection guide generator as part of our EUCLID package, with full details in the Methods section.

> ***S2P5***: *"A practical consequence of the mismatch between traditional parcellations and the lipidomic architecture is that results from lipidomic analyses dividing the brain in regions spanning multiple lipizone classes risk being confounded. Therefore, to empower future experiments, we provide tables (with ABA coordinates) to guide lipizone-informed anatomical dissections (Supplementary Figure 15c, Supplementary Table 9)."*

*This result is shown as Supplementary figure 15c:*

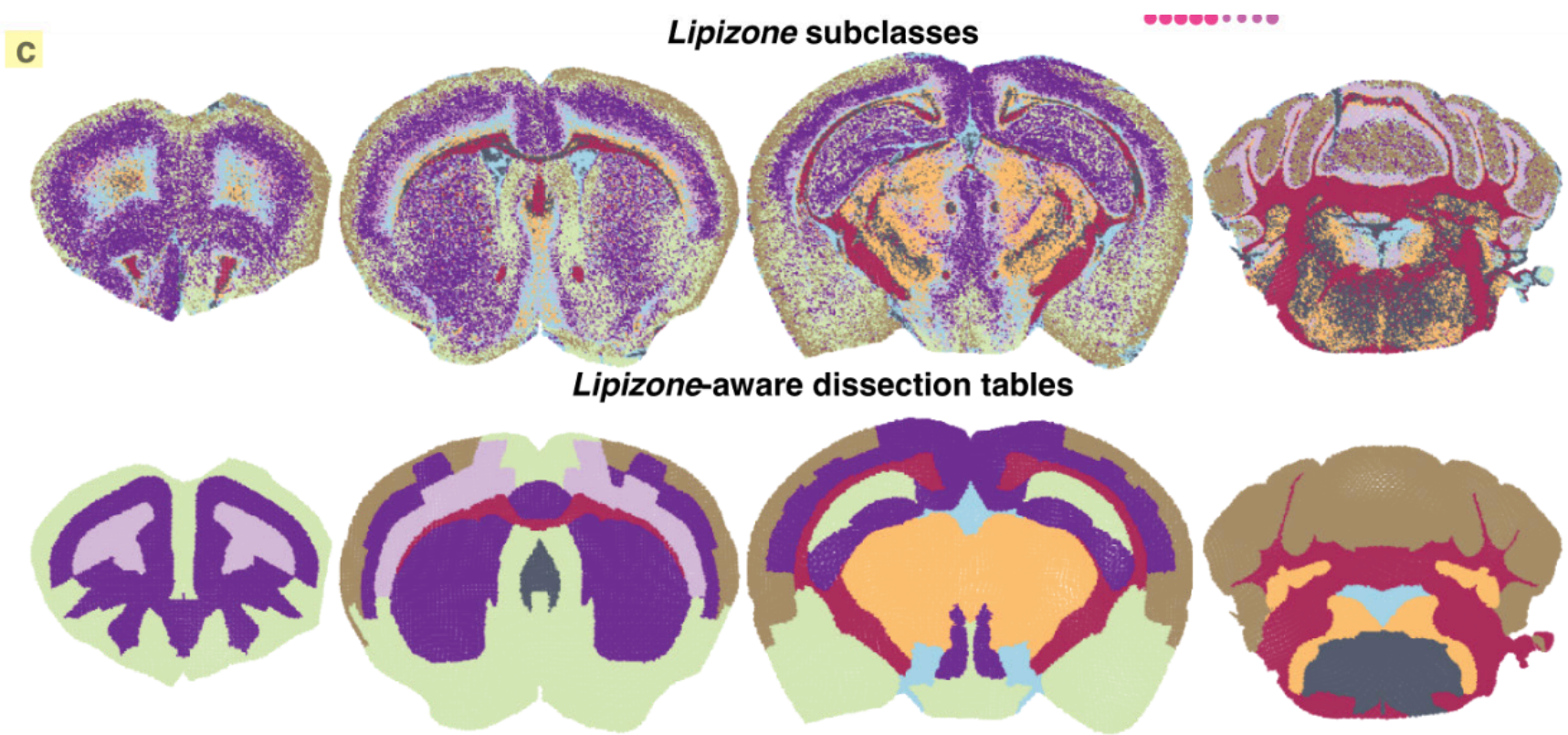


(Supplementary figure 15**c**) Representative brain sections colored by *lipizone* subclass (top) and the corresponding *lipizone*-aware dissection scheme (bottom), proposed as a reference for region selection in future targeted lipidomic studies.

*R3.2*

**Furthermore, the authors imply that they have achieved single-cell or even subcellular spatial resolution (e.g., in the Introduction), but the method is constrained by the 5 µm spot size of MALDI-MSI, which does not support this level of detail.**

*We agree that our use of these terms was wrong, and we have corrected this throughout the manuscript. We have **eliminated all uses of "quasi-cellular"** and now describe our resolution in concrete terms. At 5 µm spot size, each pixel may capture individual large cells in sparsely populated regions, but in densely populated areas it samples variable proportions of multiple cells, likely 1 to 4 per pixel. This enables the detection of cell-type-enriched territories but not the analysis of individual cells. We now state:*

> ***INTRODUCTION, P2:*** *"We mapped the distribution of 172 lipids at 5 µm laser spot size and 25 µm interpixel distance, thus sampling portions of 1-to-4 cells"*

*Where we previously used "subcellular", we now use more precise descriptions:*

> ***S4P4:*** *"Given their spatial coherence, these lipizones might represent enrichments of different subcellular regions, processes, and extracellular matrix, myelination levels, or "lipotypes""*

*The deconvolution analysis introduced in R3.3 further addresses the relationship between our pixel-level measurements and cellular composition.*

*R3.3*

**While the authors describe their spatial resolution as "quasi-cellular," they do not provide lipid composition profiles for distinct brain cell types. Such data are essential to substantiate their claims and should be included, ideally alongside a comparison to lipidomics from cultured cells.**

*We followed the referee's advice, by attempting to gain as much as possible cell type stratification, and providing a quantitative comparison between tissue lipidomes and bulk lipidomics of cultured cells. We explain here our results in 3 phases.*

***1) Cell type lipidome deconvolution.*** *True in situ single-cell lipidomics in neural tissues is currently unattainable: desorption volumes (5 µm pixel size, 10 µm section thickness) inevitably sample a mixture of cell somas and processes. Since neurons and glia have complex projections, estimating whole-cell lipidomes would require assigning distant processes to their parent soma, which no current method can do. The most informative approximation is therefore a soma-centric estimate of cell type lipidomes. We integrated cell type annotations from the Allen MERFISH spatial transcriptomics data with our atlas by mapping cells to pixels based on spatial proximity in the common coordinate framework. We modeled each pixel as contributed by the cells overlapping that location, estimating how much each cell type contributes to the observed lipid signal. This is an inverse linear problem: given the observed lipid abundances and known cell type distributions, we infer the lipid profile of each cell type. We solved it using elastic net regression with non-negativity constraints, selecting optimal parameters via cross-validation. The model generalized well to held-out regions selected for their complex cell type compositions, lending confidence to the results (Supplementary figure 12b)*

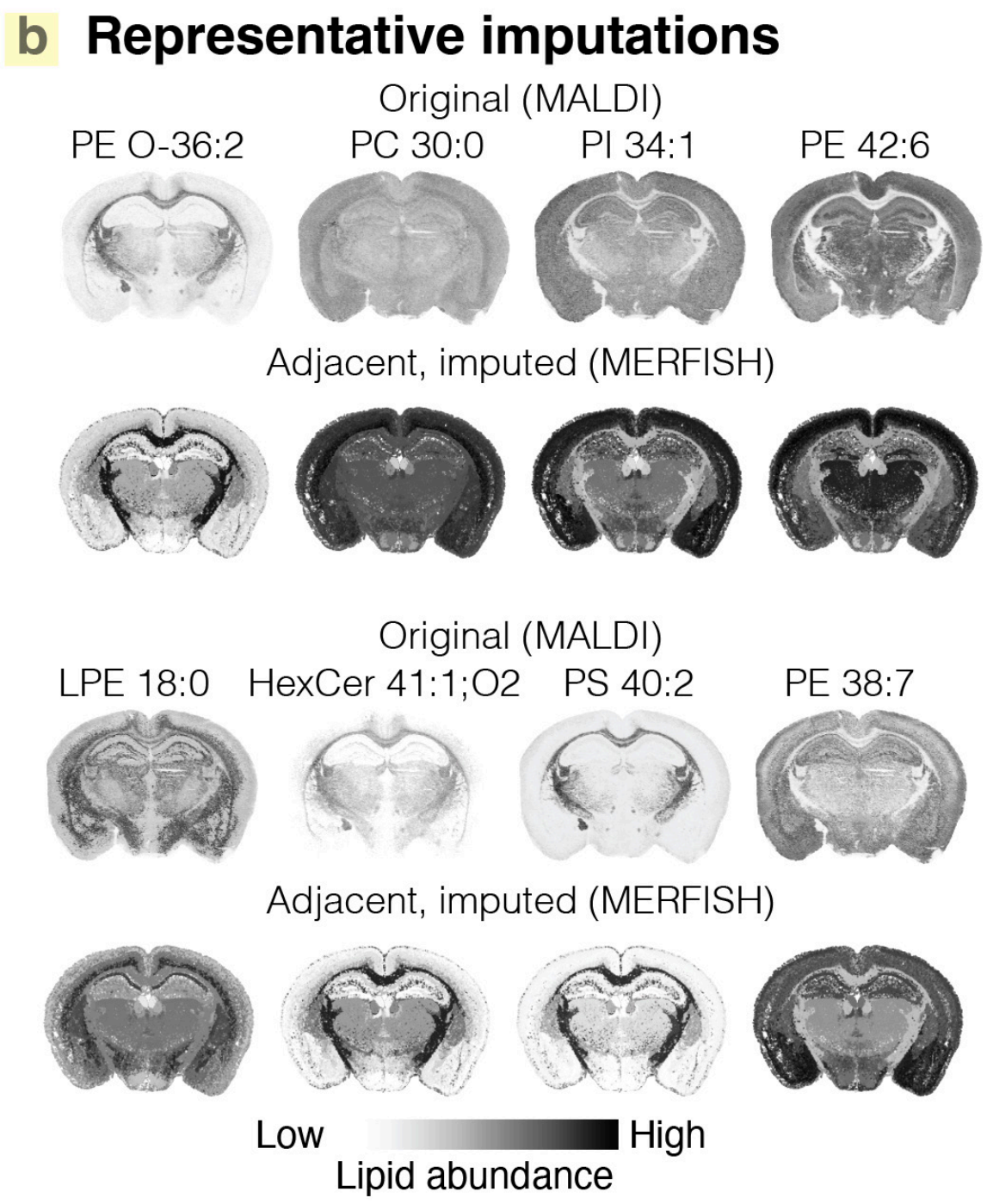


(Supplementary figure 12**b**) Spatial plots of selected lipids coloured by abundance, comparing native MALDI measurements (first row) with lipidomes reconstructed by cell type deconvolution using the closest MERFISH section (second row). Lipids were chosen to illustrate the diversity of reconstruction qualities.

*The deconvolution results are now included as Figure 3d and Supplementary figure 12; the estimated cell type lipidomes are provided as Supplementary Table 8:*

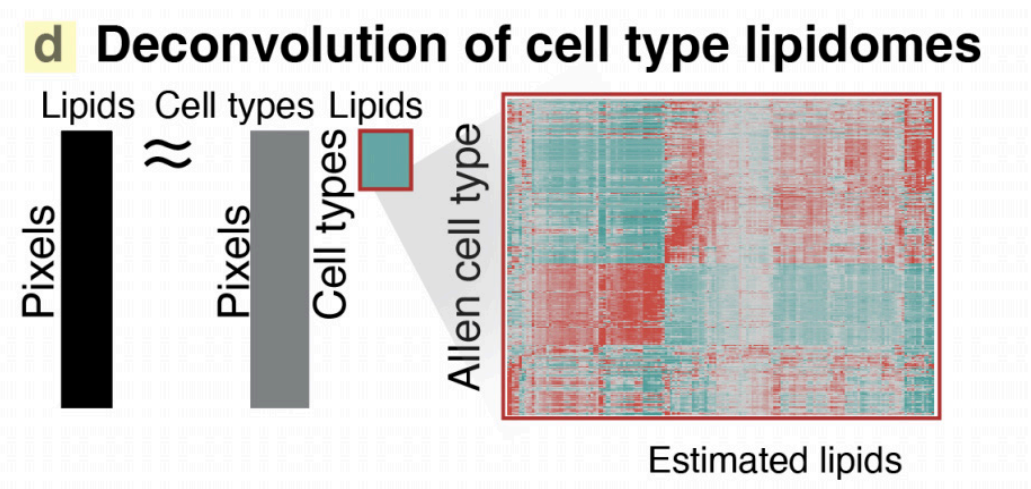


(Figure 3**d**) Schematic of the cell type lipidome deconvolution method, and miniature heatmap of the cell type x lipid matrix inferred. See *Supplementary Figure 12*.

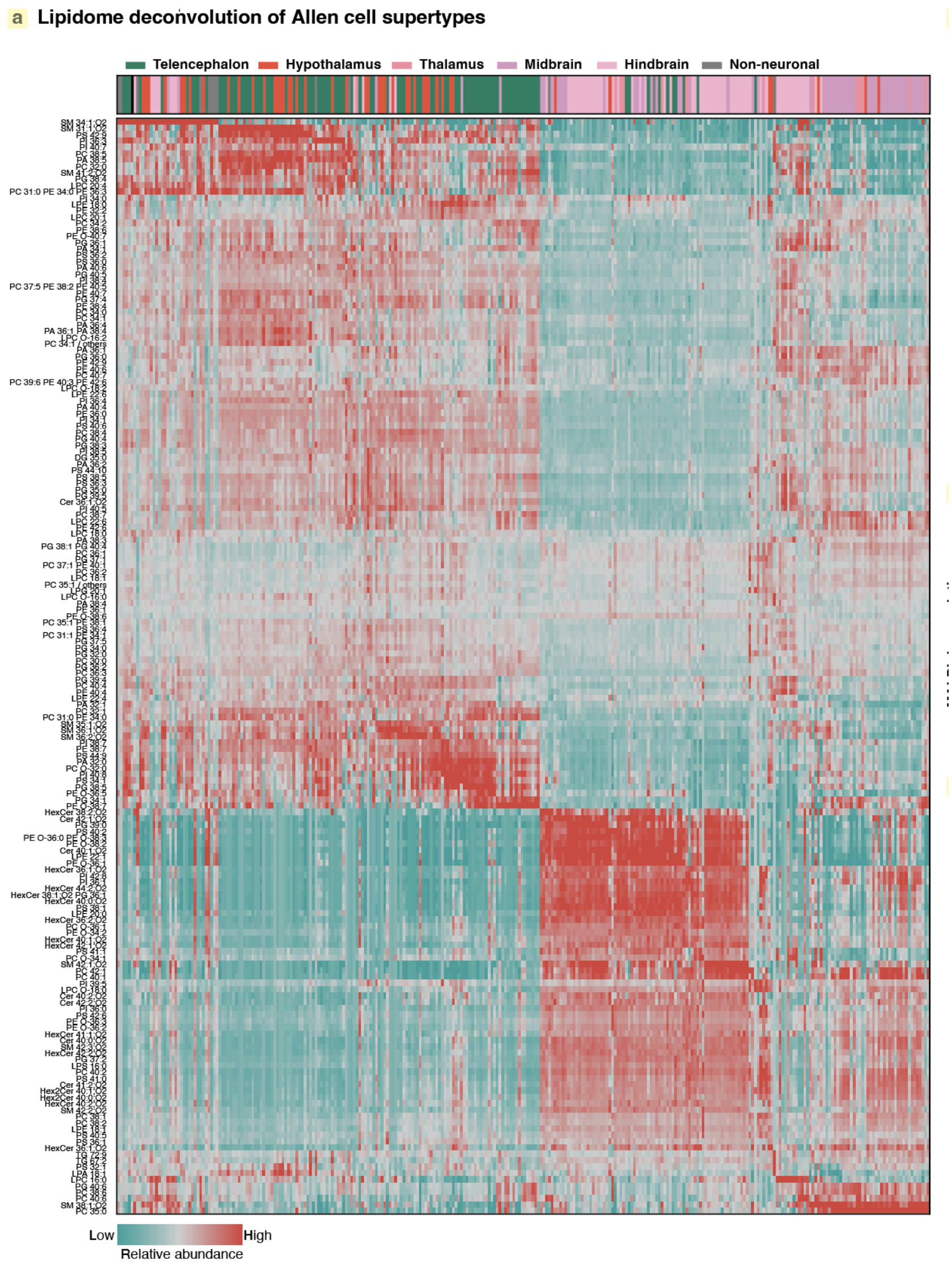


(Supplementary figure 12**a**) Heatmap of the deconvolved cell type lipidomes; colour represents relative abundance. Columns are Allen subclass-level cell types; rows are individual lipid species. The coloured bar at the top annotates each cell type by the anatomical region it is most enriched in.

*We next looked into the cell type lipidomes to understand the differential lipid enrichments in known cell types. We showed these enrichments using dotplots (Supplementary figure 13) and dividing by brain regions with a focus on populations both central in Neuroscience and heterogeneous in our dataset.*

*For instance, in the cortex, deep-layer pyramidal types (L5/L6) are enriched in ether-linked phospholipids (PE O-36:1) and hexosylceramides (HexCer 38:1;O2), whereas superficial-layer types (L2/3) favor polyunsaturated species such as PS 42:9. In the hippocampus, CA1 pyramidal neurons are enriched in LPE 18:0 and SM 34:1;O2, while DG granule cells have SM 34:1;O2 and PC 35:0 enrichment. In the hypothalamus, ventral populations such as MPO-LPO Lhx8 Gaba are distinguished by stronger SM 36:2;O2.*

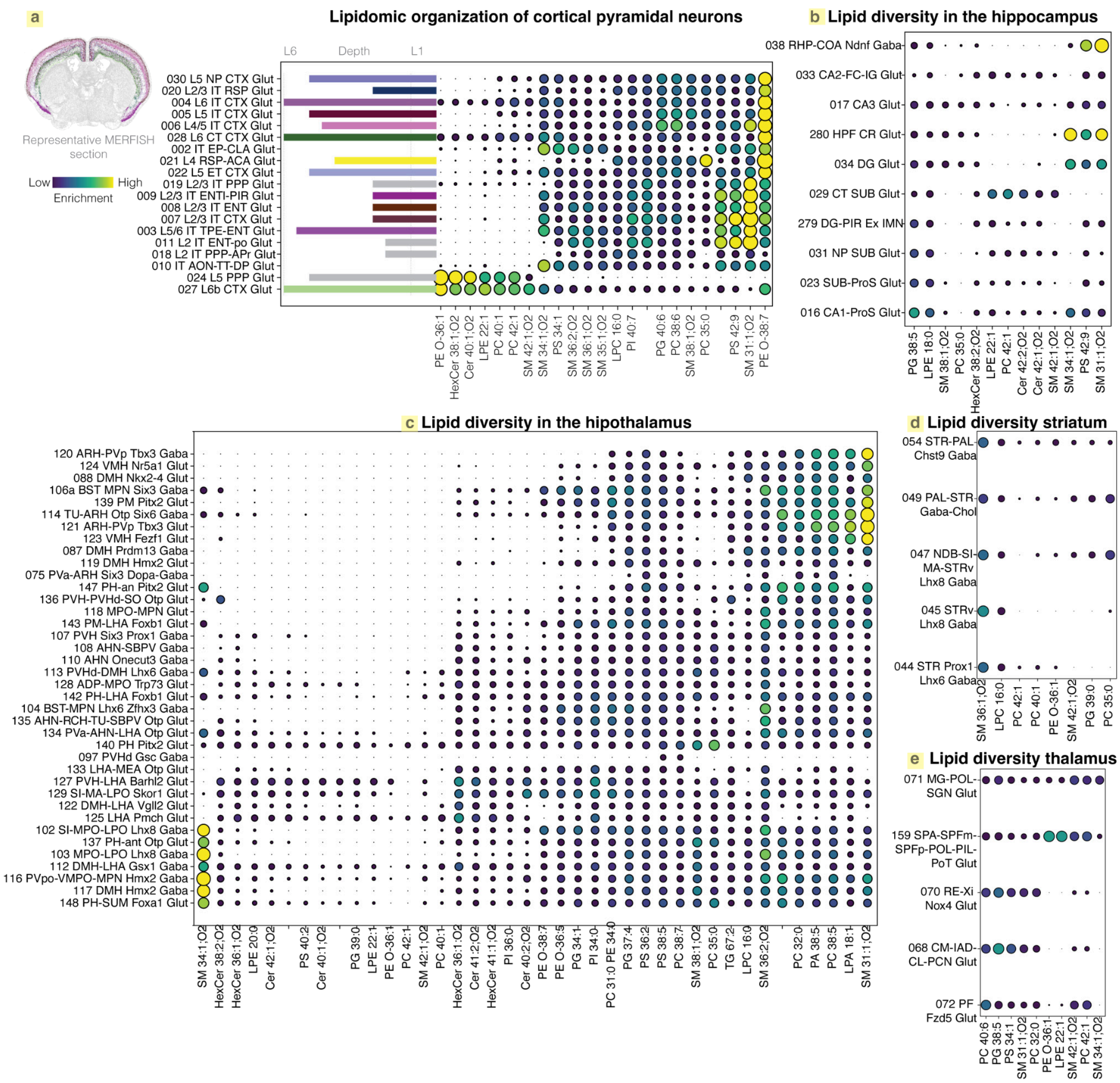


**Supplementary Figure 13. Lipid markers of cell types.** (**a**) Dot plot of lipid enrichment scores from the deconvolution analysis for cortical pyramidal neurons (rows) and selected lipids (columns). Horizontal bars indicate the cortical depth of each cell type from layer 6 to layer 1, coloured by Allen MERFISH subclass. Inset: spatial distribution of pyramidal cortical neuron subclasses in a representative MERFISH section, using the same colour code (**b–e**), Dot plots as in (a), showing lipid enrichment for hippocampal (**b**), hypothalamic (**c**), striatal inhibitory (**d**) and thalamic (**e**) neurons.

***S4P2***: *“We used CCF-registered cell type information to estimate the lipidome of individual cell types by deconvolution (Figure 3d; Supplementary figure 12a, b; Supplementary Table 8).”*

*“The deconvolution revealed specific lipid enrichments (Supplementary Figure 13a-e), such as in the cortex, deep-layer pyramidal neuron territories (L5/L6) are enriched in specific ether-linked phospholipids (PE O-36:1) and hexosylceramides (HexCer 38:1;O2), whereas superficial-layer types (L2/3) favor polyunsaturated species such as PS 42:9. In the hippocampus, CA1 pyramidal neurons are enriched in LPE 18:0 and SM 34:1;O2, while DG granule cells have SM 34:1;O2 and PC 35:0 enriched. In the hypothalamus, ventral populations such as MPO-LPO Lhx8 Gaba are distinguished by stronger SM 36:2;O2. ”*

***2) Comparison with cultured cells.*** *Comparison with cultured cells. As suggested, we compared our deconvolved estimates with bulk lipidomics data from primary neuronal, oligodendrocyte and astrocyte cultures (Fitzner et al.), using an outlier rejection procedure and quantifying agreement based on rank correlations of enrichments (as lipid classes are not directly comparable in MALDI; see Methods). We found a coherent match between corresponding cell type lipidomes obtained by deconvolution and their in vitro counterparts. This agreement was not, however, devoid of strong discrepancies for specific lipids, consistent with in vitro idiosyncrasies previously reported (Gopalan et al. 2024); neurons showed the lowest similarity, as expected given the substantial specification and maturation occurring between E16.5 (the stage used in Fitzner et al. 2020) and the adult neurons captured in our atlas.These findings are now reported in S4P2 and shown in Supplementary figure 12c, d.*

> ***S4P2***: *"Deconvolution-based lipid signatures were broadly consistent with those of cell types in vitro derived by bulk lipidomics in Fitzner et al. Oligodendrocytes were the best matched (Spearman's R 0.77; Supplementary figure 12 c, d). Yet, our analysis detected 8-15% lipids significantly discordant per cell type. This is consistent with the known partial divergence between tissue and in vitro lipid profiles."*

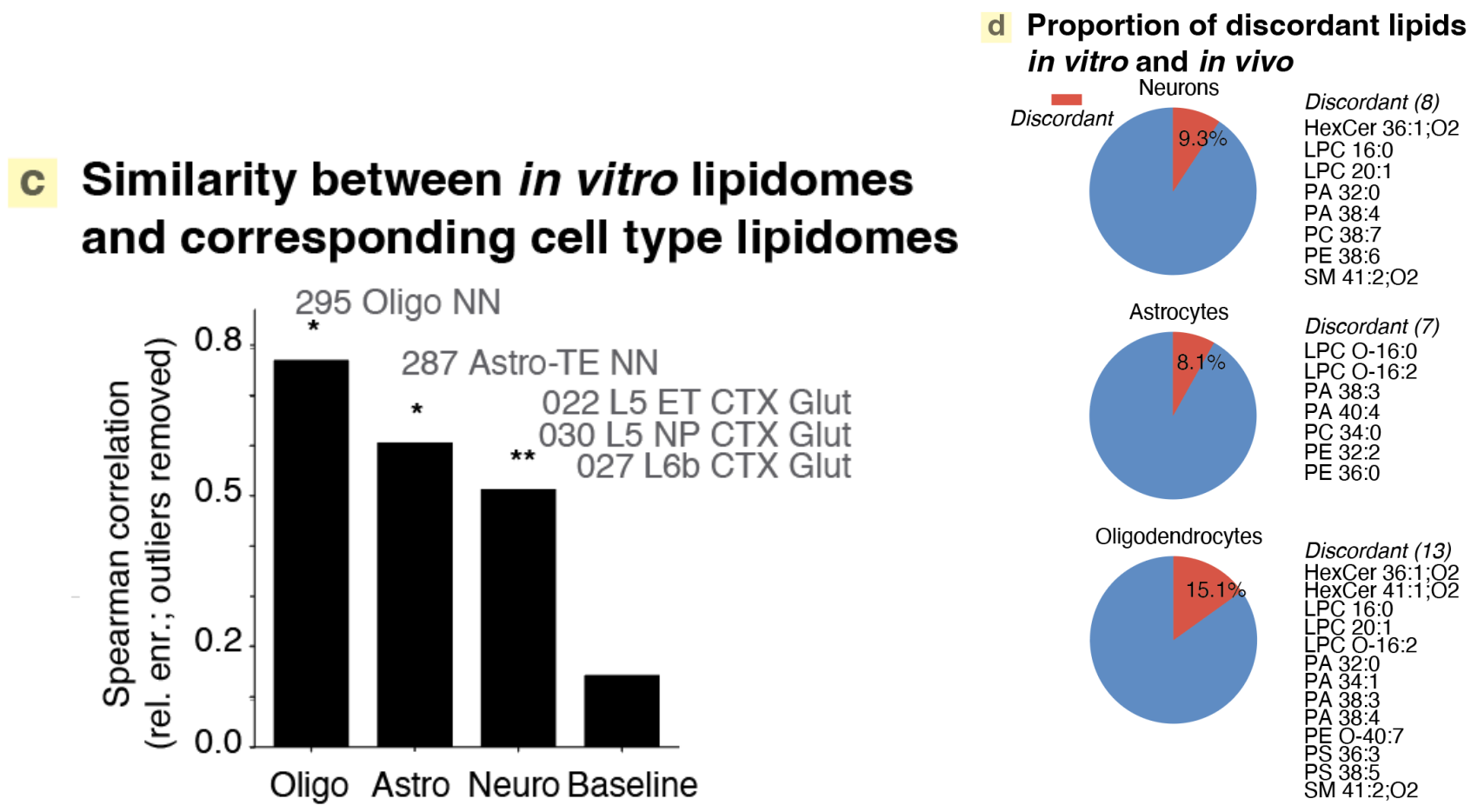


(Supplementary figure 12**c**) Bar plots of the Spearman correlation between cell type lipid enrichments estimated by deconvolution of the Lipid Brain Atlas and those measured by bulk LC-MS (Fitzner et al.), after outlier removal. (**d**) Pie charts showing the fraction of discordant lipids (red) excluded from each LC-MS versus MALDI comparison because largely and consistently diverging between tissue and *in vitro* cell types.

*This analysis and R1.2 also prompted us to revisit Figure 4d: we now introduce astrocytes along with oligodendrocytes and neurons as possible sources of white matter heterogeneity. To robustify the analysis and ask if the white matter lipizones best explain the patterns of oligodendrocytes, astrocytes, or neurons independently of the variable number of cell type-specific lipids, we calculated the variance explained by lipizones for each individual lipid. We then compared the distributions of oligodendrocyte-, astrocyte, and neuron-specific lipids to determine if the unbiased clustering was best capturing the distribution of any cell type-specific lipid profile. We have also integrated this analysis in the clustering results stability work presented in R2.1, showing that the conclusions remain valid across clustering regimes. This analysis confirmed that the white matter lipizone diversity mostly corresponds to heterogeneity of oligodendrocyte-specific lipids, and is now included as Figure 4b:*

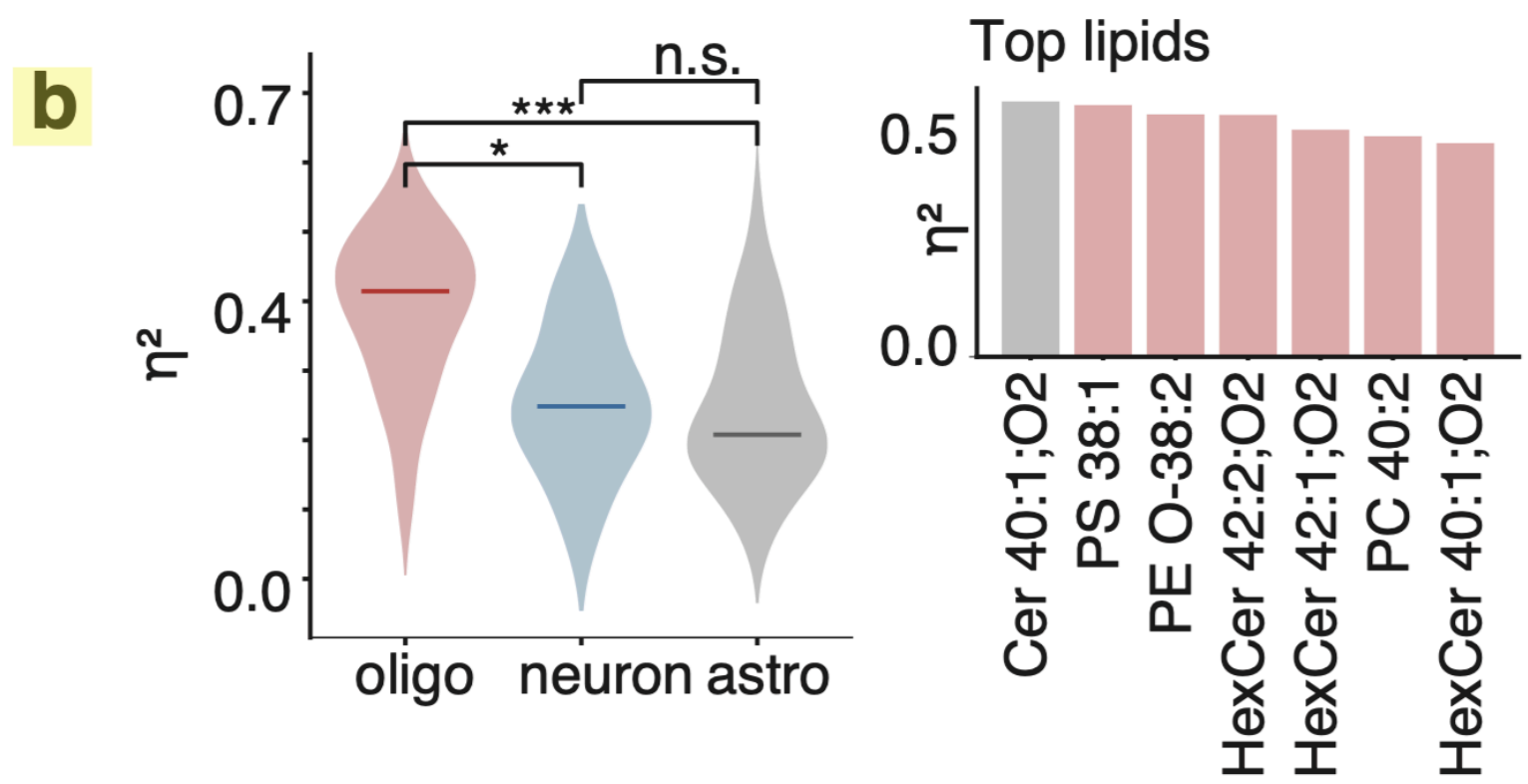


(Figure 4**b**) Violin plot for lipid-wise $\eta^2$, the ratio of the sum of squares between clusters and within+between (SS_between / SS_total), using the 65 terminal white matter *lipizones* as the grouping factor. The lipids shown are those restricted to oligodendrocytes, neurons, and astrocytes, based on Fitzner et al. Inset: barplot showing the individual lipids with highest $\eta^2$, i.e., best captured by *lipizones*, colored by category.

***3) Validation against independent single-cell lipidomes.*** *To provide more direct external validation of the cell type lipidomes, we integrated our dataset with the two only (to our best knowledge) brain single-cell lipidomics datasets published, from adult rat brain ((Xie et al. 2024)) and developing human cortex ((Bhaduri et al. 2021)). For each dataset, we subsetted to common lipids, harmonized distributions, and assigned single-cell clusters to lipizones based on cosine similarity of lipid profiles (see Methods for full details). Rat brain single-cell clusters mapped coherently onto our lipizones, recapitulating the expected spatial organization of major brain regions. For the developing human cortex, the mapping was consistent with cortical lipizones (as we report in Supplementary Fig. 14a–d). These cross-species comparisons reinforce the view that lipizones reflect conserved cell type-related lipid organization, rather than idiosyncrasies of our particular dataset or methodology.*

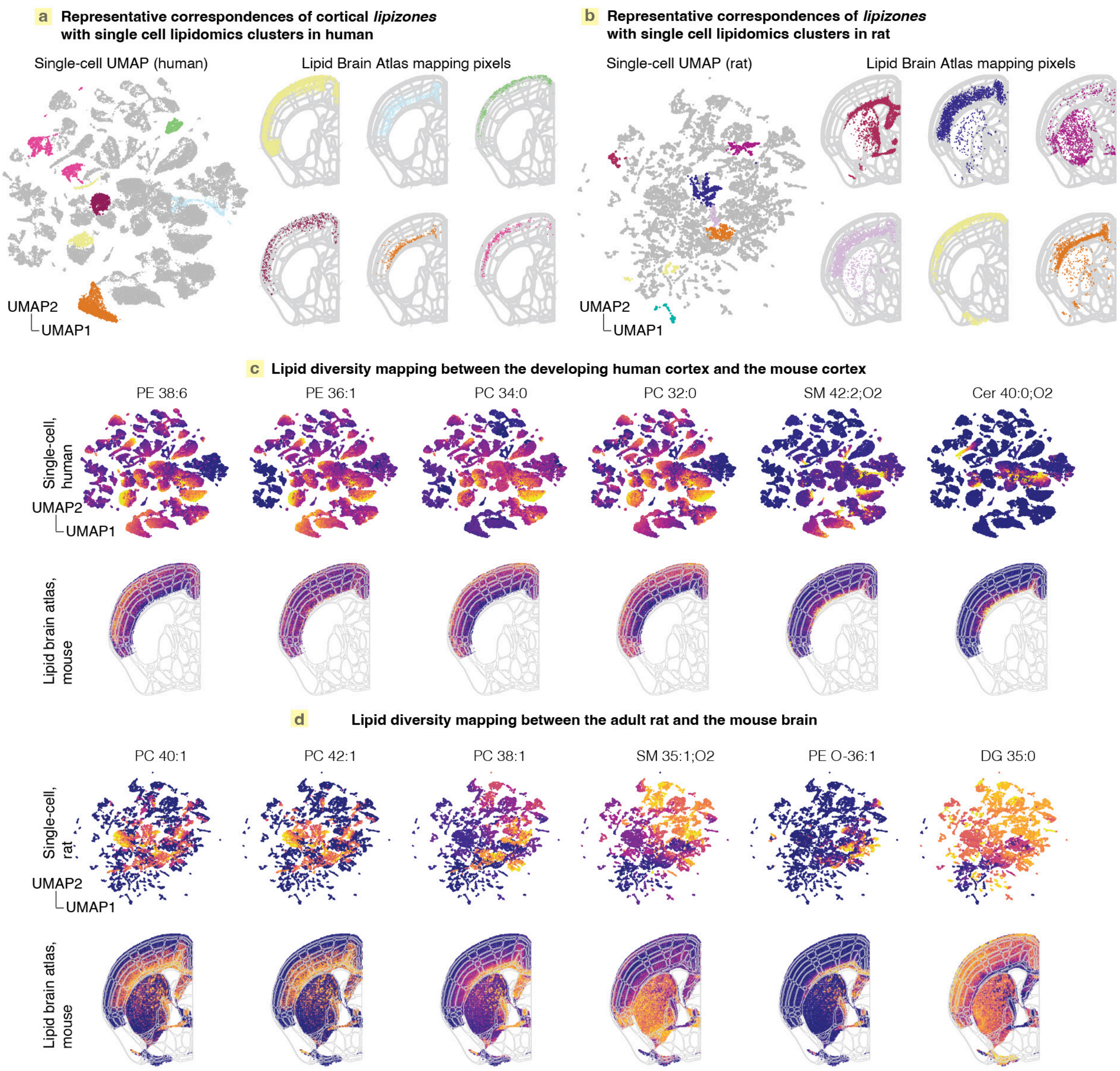


**Supplementary Figure 14. Cross-species comparison of lipidomic clusters.** (**a**) UMAP of single-cell lipid profiles from the developing human cortex (Bhaduri et al.), with selected clusters highlighted in colour. Right: spatial mapping of the selected clusters onto a half-section of the mouse Lipid Brain Atlas. (**b**) As in (a), for single-cell lipid profiles from the adult rat brain (Xie et al.). (**c**) UMAPs of single-cell lipid profiles from the developing human cortex, coloured by selected individual lipids, with corresponding spatial projections onto the mouse Lipid Brain Atlas. (**d**) As in (c), for single-cell lipid profiles from the adult rat brain.

> ***S4P2***: *"We next asked whether similar lipidomic-based entities could be recovered in single-cell data and other species. We mapped single-cell lipidomics data from adult rat brain[12] and developing human brain cortex[49] onto our atlas, finding lipid profiles aligned with lipizones (Supplementary Figure 14a-d). This correspondence reinforces the observation that lipizones capture cell type-level variability, and raises the possibility that this organization may be conserved."*

*R3.4*

**Thus, overall this study represents the first comprehensive spatially resolved lipid map of the mouse brain. However, biological insights provided are limited.**

*We respectfully disagree that the biological insights are limited, though we acknowledge that the previous narrative may not have delivered them effectively. We summarize the main discoveries, including several new analyses inspired by the referees' comments, below, noting where the revised manuscript strengthens or extends them.*

*At the atlas level, we are the first to demonstrate at scale that lipids alone can define fine-grained brain regions, highlighting how complex combinatorial patterns of this understudied class of molecules are intimately related to brain organization. The resulting parcellation is not reducible to transcriptomic hierarchies (see R3.1). We show that functionally connected brain regions share lipid signatures even when spatially distant, proposing a previously unknown correspondence between membrane composition and circuit architecture. We discover unexpected sphingomyelin enrichment in ventricles with distinct spatial markers such as SM 34:1 and SM 38:1, dorsoventral lipid gradients along neurogenic zones correlating with position-dependent neural stem cell fates, and choroid plexus heterogeneity correlating with embryonic positional identity. We identify 65 biochemically distinct white matter zones driven by oligodendrocyte-specific lipids, demonstrating that myelin composition varies systematically with anatomical location despite limited transcriptional diversity among oligodendrocytes. In the revised manuscript, we have validated that this lipid heterogeneity corresponds at least in part to microstructural variation, using immunostainings for Opalin (highlighting variable density of myelin compartments), Klk6 (an oligodendroglial serine protease involved in demyelinating diseases), and CNPase (labeling non-compact myelin portions, serving as a proxy for myelin compactedness) (Figure 4n, o).*

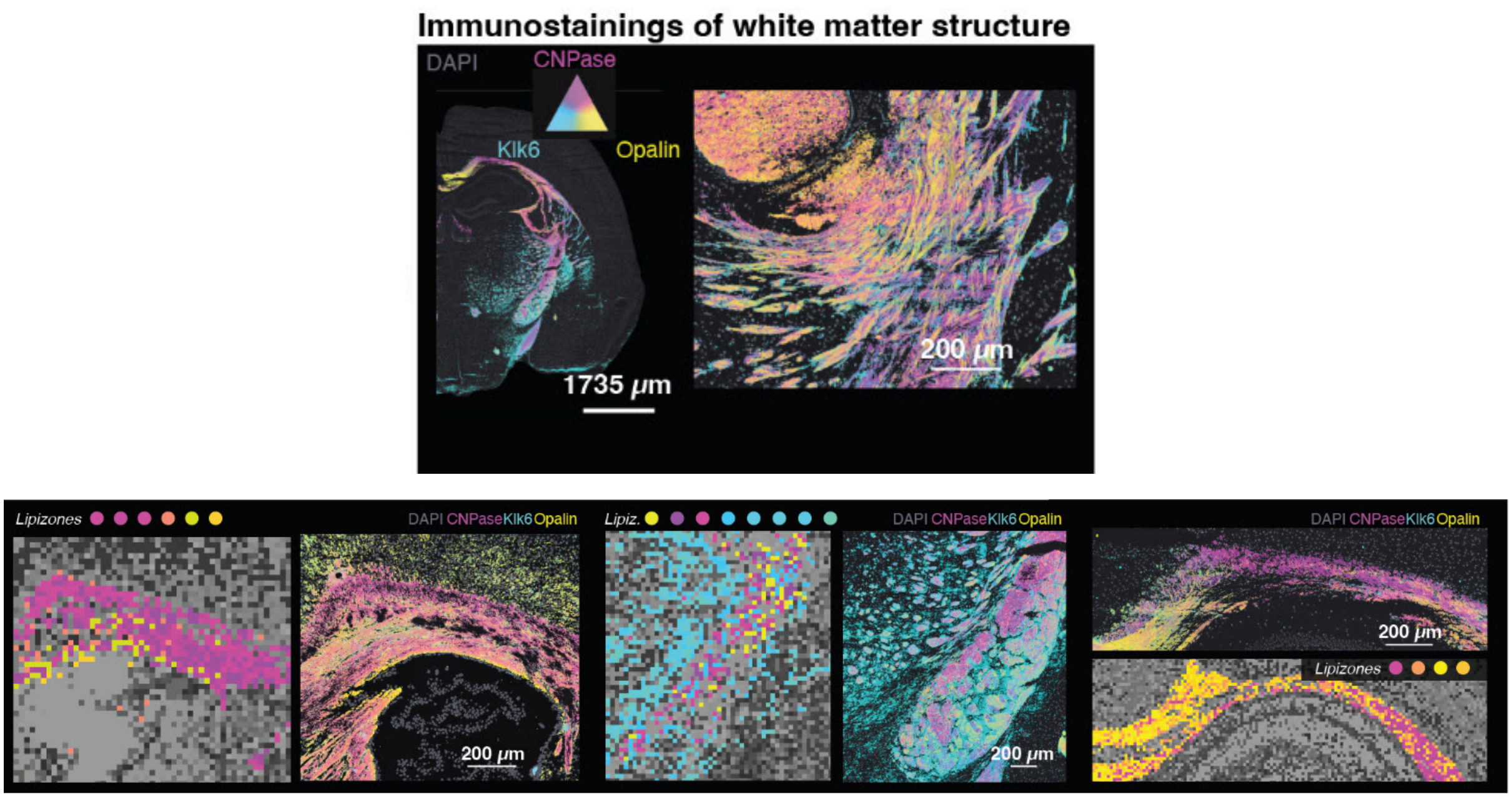


(Figure 4**n**) Immunostainings for Opalin, Klk6, and CNPase, three proteins related to myelin structure. Coloring is based on the position of a pixel in the simplex whose three vertices are the three pure colors. (**o**) Spatial plots for comparison between selected *lipizones* and the white matter microstructure measured by immunostaining. *Lipizone* colors were reassigned to ease the visual comparison with the immunostainings: one *lipizone* per C/M/Y channel was manually chosen and the colors of the others were determined using the simplex.

> ***S5P5***: *"We hypothesized that this regional heterogeneity of myelin lipidomes might correspond to local variations in myelin structure, such as compactedness, density,*

*thickness, or frequency of Ranvier nodes. We therefore conducted immunofluorescence experiments, staining for Opalin (to highlight potential variable density of myelin), Klk6 (an oligodendroglial serine protease involved in demyelination and remyelination), and CNPase (which labels the non-compact portions of myelin). White matter territories were zonated based on these three structural markers (Figure 4n), and we found instances where this zonation pattern was similar to lipizones organization (Figure 4o). This observation suggests that white matter lipizones may capture myelin biophysically-related heterogeneities."*

*In the revised manuscript, we also better highlight a key insight that remained implicit in the original submission: the substantial variability within individual lipid classes. Many lipid **species within the same class** exhibit distinct spatial distributions that **diverge** from the other members of that class, with no significant difference in within- versus between-class spatial correlation (BH-FDR-corrected Mann-Whitney one-sided test; Figure 2g; Supplementary Fig. 2e,f). This challenges the common practice of analyzing lipids at the class level and argues for maintaining species-level granularity in future investigations.*

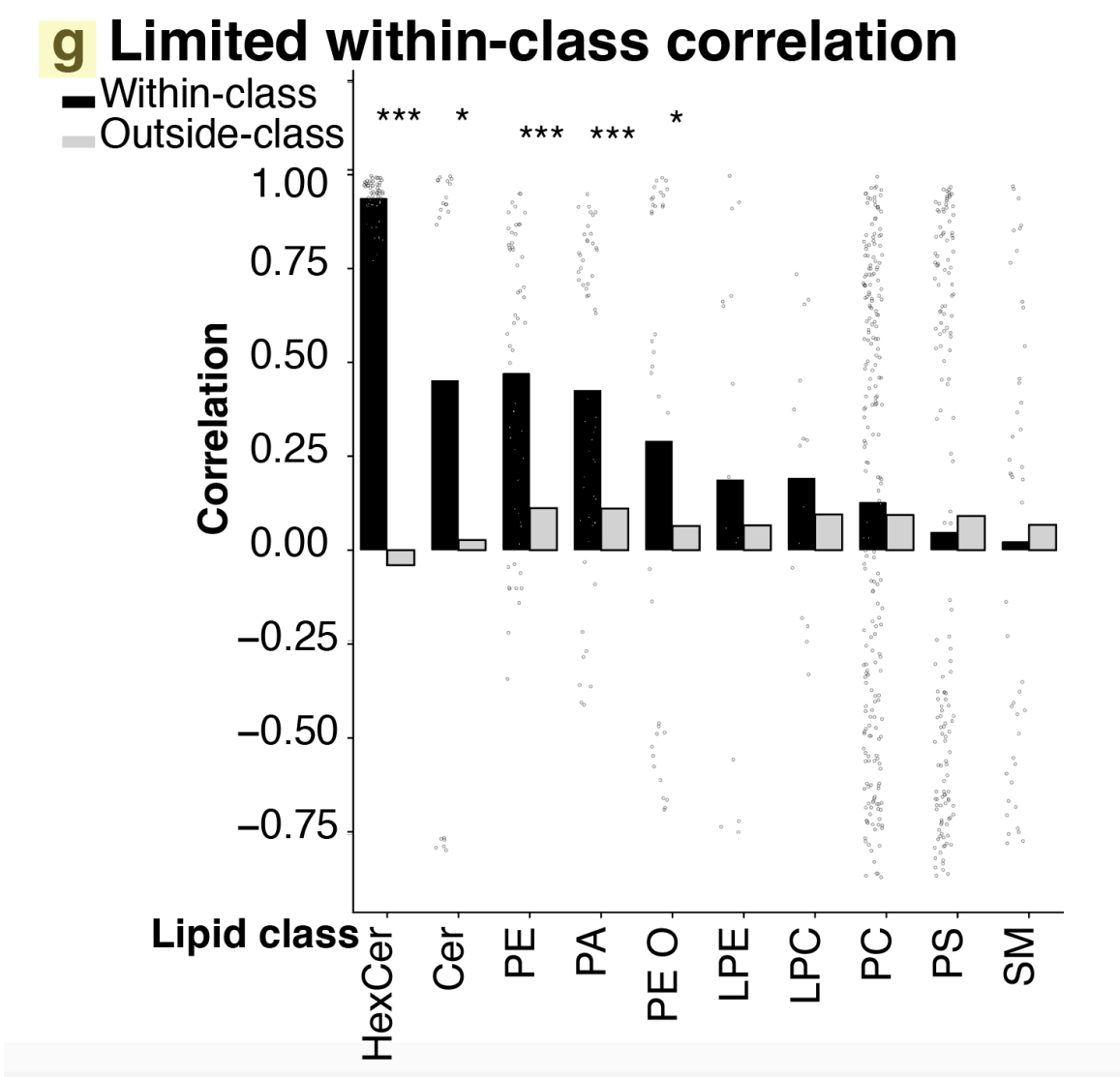


(Figure 2**g**) Barplots showing the average Pearson's correlation between lipids of the same class and of different classes. Individual lipid-lipid correlations are overlaid as a strip plot.

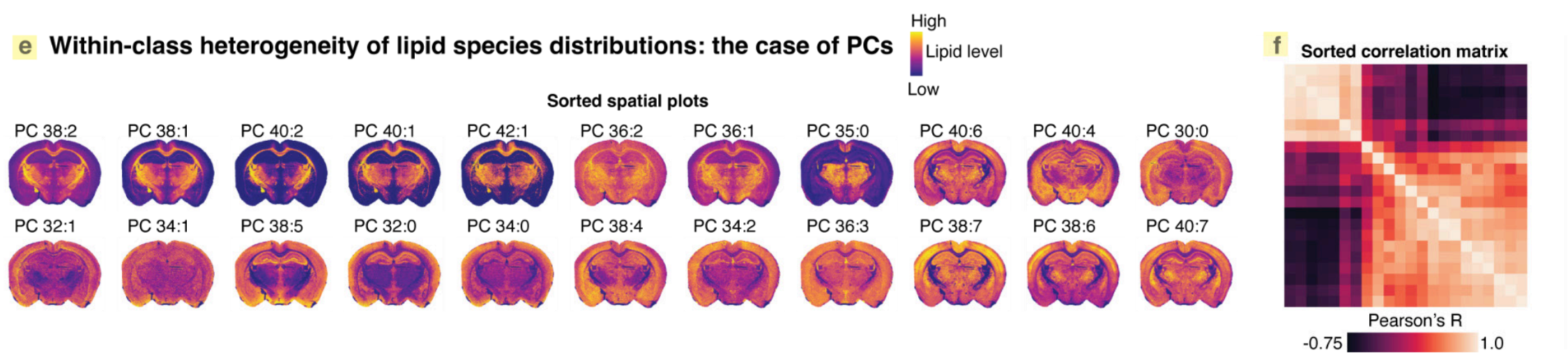


(Supplementary figure 2**e**) Spatial plots for all the PC lipids in the Lipid Brain Atlas on a representative section, sorted by the (**f**) lipid-lipid correlation matrix (heatmap).

***S2P7**: "Many lipid species within the same class, especially for classes LPC, LPE, PC, PS, and SM, had spatial distributions that diverged from the other members of the same class (no difference in within- versus between-class spatial correlation;*

*BH-FDR-corrected Mann–Whitney one-sided test, FDR=0.05 threshold; Figure 2g, Supplementary Figure 2e, f). This observation suggests that future investigations should maintain the granularity of individual lipid species whenever possible."*

*Next, in the revised manuscript, to relate the lipid heterogeneity that we reported to **known biological processes**, we performed a systematic differential expression analysis at each binary split of the lipizone hierarchy across 10 broad gene categories, including membrane organization, lipid binding, metabolic enzymes, cell adhesion, neurotransmitter and neuropeptide systems, and transcription factors (Supplementary Fig. 18). A clear progression emerged: the **early splits** of the hierarchy are dominated by **cell adhesion** and **membrane organization genes**, reflecting tissue-level structural distinctions; as the hierarchy **deepens**, **lipid transporters** and then **neurotransmission-related** genes take over, suggesting that finer lipidomic distinctions track with functional neuronal specialization. Two additional results stand out: **metabolic enzyme** genes are consistently among the **least enriched sets**, indicating that lipid configurations are not a trivial consequence of metabolic gene expression but rather support specific cytological and tissue organization processes; and **transcription factor genes** also show **low enrichment** across all splits, suggesting that lipid configurations relate to cellular processes beyond transcriptional programs.*

*We now dedicate a full paragraph to this important analysis:*

***S4P10**: "Finally, to characterize lipizones and their hierarchy in terms of known biological processes, we used the imputed (Yao et al. 2023) et al. atlas to find the differentially expressed genes (DEGs) at each binary split in the lipizone hierarchy (Welch's t-test, Supplementary Figure 18a). To assess whether specific gene sets exhibited coordinated differential expression at a given split, we then calculated the percent gene set coverage of the DEGs (see Methods). Cell adhesion and membrane organization-related genes led the second split of the hierarchy, with genes with transporter activity leading from the third split, followed by genes related to lipid binding and membrane organization. These gene sets stayed dominant, along with the neurotransmitter and neuropeptide set which emerged at deeper splits to eventually dominate the DEGs. Interestingly, the gene set related to metabolic enzymes was consistently (except for the third split) one of the two least changed sets, further corroborating that lipid configurations are likely not a trivial consequence of the expression of metabolic genes, but may rather support specific cytological and tissue organization processes. Finally, transcription factors also had low enrichment across all splits, suggesting that lipid configurations may be related to cellular processes beyond core cell-identity programs."*

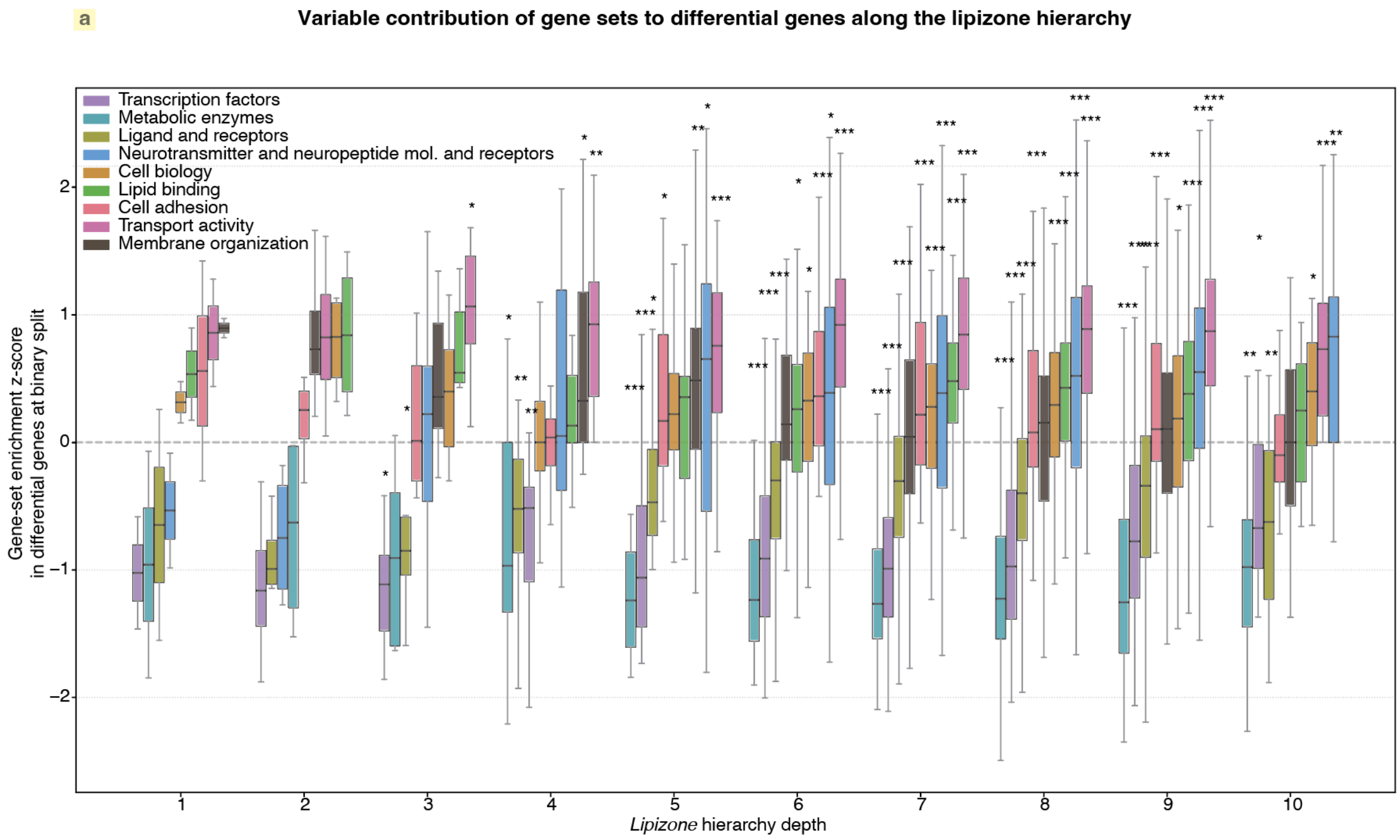


**Supplementary Figure 18. Gene set enrichment among differentially expressed genes at each level of the *lipizone* hierarchy.** (**a**) Box plots showing the enrichment z-score of nine gene sets among differentially expressed genes at each binary split of the *lipizone* hierarchy (x-axis). At each hierarchy depth, boxes are sorted by ascending median. Asterisks denote significant deviation of the median z-score from zero (two-sided Wilcoxon signed-rank test, Benjamini–Hochberg-corrected): *q < 0.05, **q < 0.01, ***q < 0.001.

*Finally, we have substantially expanded the biological insights from the pregnancy model, as **detailed in R2.4**. Beyond the original white matter and outer cortex findings, we now highlight the specific lipids changed and the most affected regions, use gene expression with feature importance and gene ontology analysis to explain lipidomic changes in terms of known biological processes, connect myelin changes to an oligodendrocyte differentiation signature, validate white matter changes using LC-MS, report a correlation between lipidomic changes in pregnancy and Pgr expression, interpret changes in the light of circuitry by feature importance analysis, extend the CORNETO framework to postulate upstream receptor pathways driving the observed lipid changes, and validate its predictions by immunostaining. These analyses and discoveries are detailed in **Section 7** and Figure 6 of the updated manuscript.*

*We acknowledge that the Lipid Brain Atlas is a starting point toward understanding the role of individual lipid species in physiology and beyond. By providing a rich, accessible reference, it offers a framework for hypothesis generation that awaits functional follow-up. At the same time, the discoveries reported here, from connectivity-lipid correspondence to myelin heterogeneity to pregnancy-associated remodeling represent substantial and in several cases unexpected biological insights.*

*R3.5*

**Moreover, it is unclear whether this analysis will serve as a valuable resource without identifying the lipid compositions of the various cell types. The concept of "lipizones" appears artificial and may prove difficult to apply in future studies.**

*We have now addressed the first concern comprehensively in **R3.3**, where we report **cell type lipidomes obtained through deconvolution**, comparison with **published bulk** and single-cell lipidomics datasets, and cross-species mapping from rat and human data onto our atlas.*

*Regarding the applicability of lipizones, we note that they emerge directly from the data across more than 25 clustering regimes (R2.1), so they are not an imposed classification. The central question is whether a researcher working on, say, thalamic circuits or cortical myelination can **concretely use lipizones** in their experimental design and interpretation. We believe the answer is yes, and in the revised manuscript we demonstrate this through several complementary lines of evidence.*

***Pragmatic tools for wet-lab experiments.** There are no universal single-lipid markers for lipizones, but most lipizones become identifiable by just a handful of lipids once the anatomical context is narrowed to a specific brain region. Since regionally focused studies are the typical experimental setting, this means that local lipid marker combinations can be used to identify and track lipizones in practice. We now provide these context-dependent markers as Supplementary Table 6 making the lipizone framework directly actionable for targeted experiments. For instance, Cer 42:1;O2 and PS 40:2 mark cortical layer 6 within the cortex; PS 44:10 and PA 38:3 mark the medial mammillary nucleus and other subregions within the hypothalamus (Supplementary Fig. 9j).*

> ***S2P10**: "In fact, although lipids functioning as markers across the entire brain are scarce, lipids acting as markers can be identified within individual brain regions. For example, Cer 42:1;O2, PS 40:2 within the cortex mark cortical layer 6; PS 44:10 and PA 38:3 within the hypothalamus mark the medial mammillary nucleus and other subregions (Supplementary Figure 9j). We provide these context-dependent, within-region lipid markers as Supplementary Tables 6 to favor their use in regionally focused neurometabolic studies."*

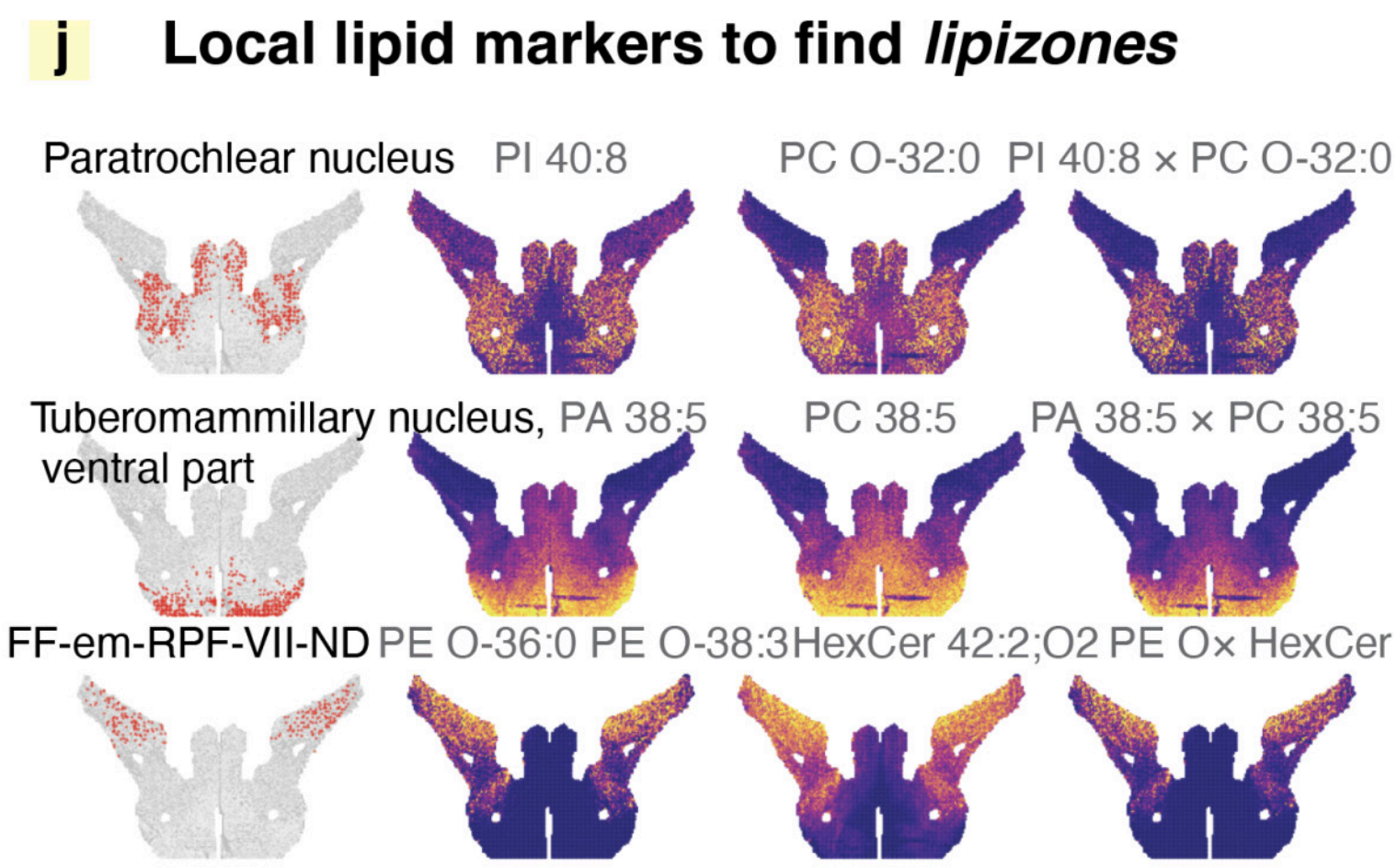


(Supplementary figure 9**j**) Spatial plots of representative hypothalamic *lipizones* (red on grey) alongside their local marker lipids, illustrating how such *lipizones* can be recognized in localized studies.

> *We now release the **regional lipidomes, cell type, and lipizone lipidomes as supplementary tables 5 and 8**.*

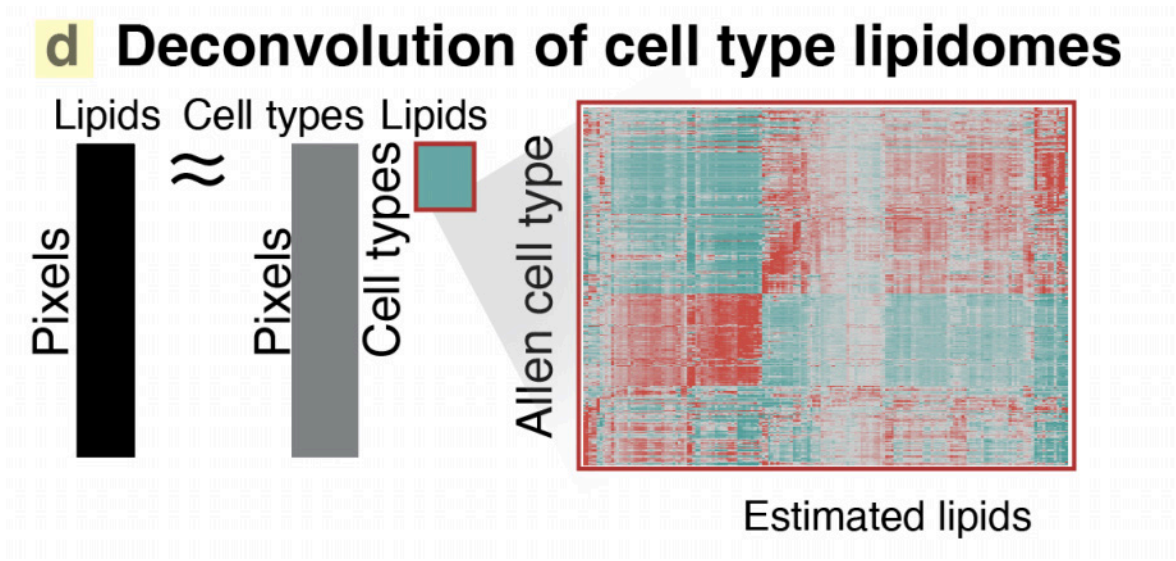


(Figure 3**d**) Schematic of the cell type lipidome deconvolution method, and miniature heatmap of the cell type x lipid matrix inferred. See *Supplementary Figure 12*.

*We have built lipizone-aware **dissection tables** for future bulk lipidomics studies, as detailed in R3.1. These tables, along with the awareness that **individual lipid species** should be characterized beyond their class-level patterns, provide a pragmatic setting for productive future experiments.*

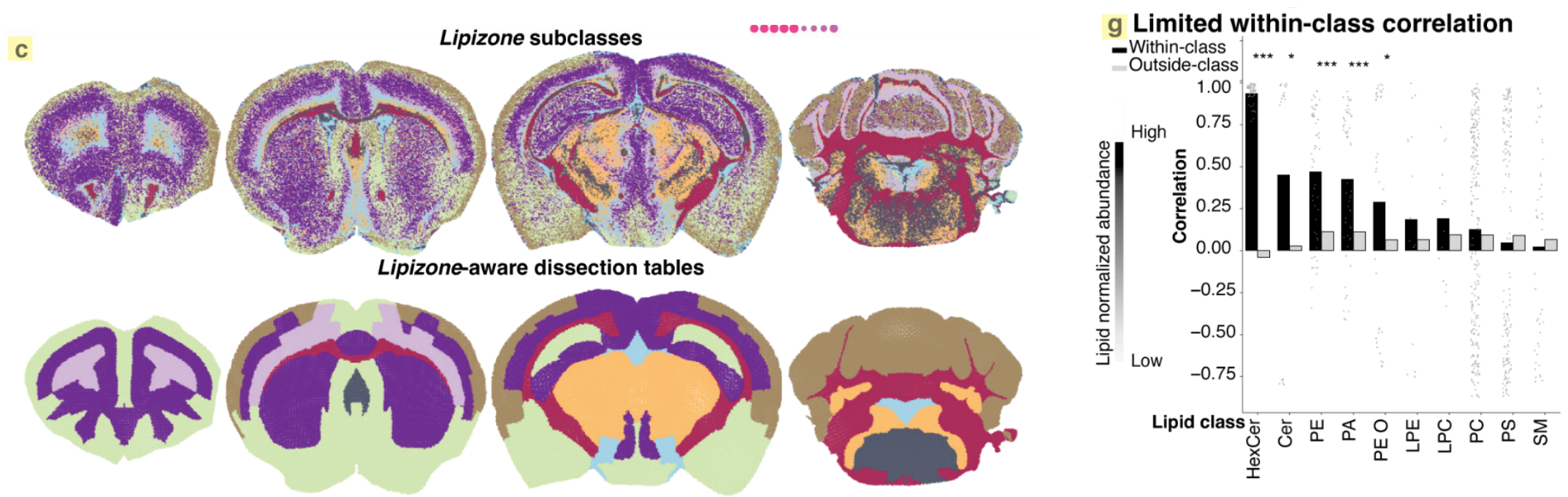


(Supplementary figure 15**c**) Representative brain sections colored by *lipizone* subclass (top) and the corresponding *lipizone*-aware dissection scheme (bottom), proposed as a reference for region selection in future targeted lipidomic studies.

***Bioinformatic tools for new data.** Our analytical pipeline, EUCLID, allows mapping of new samples onto the atlas to annotate lipizones and extract differential lipids using the atlas as reference (Supplementary Fig. 4a). This label transfer capability means that the lipizone framework is not locked to our dataset, opening to incremental additions to our resource. We now better highlight this in the manuscript:*

***S2P12**: "These results establish the presence of structured regional heterogeneity within the brain lipidome. The lipizone parcellation offers a framework for biochemical stratification, empowering discovery in disease studies and experimental perturbations (implemented in EUCLID, whose label transfer function maps lipizones onto new samples; see Methods ; see its application in Section 7)."*

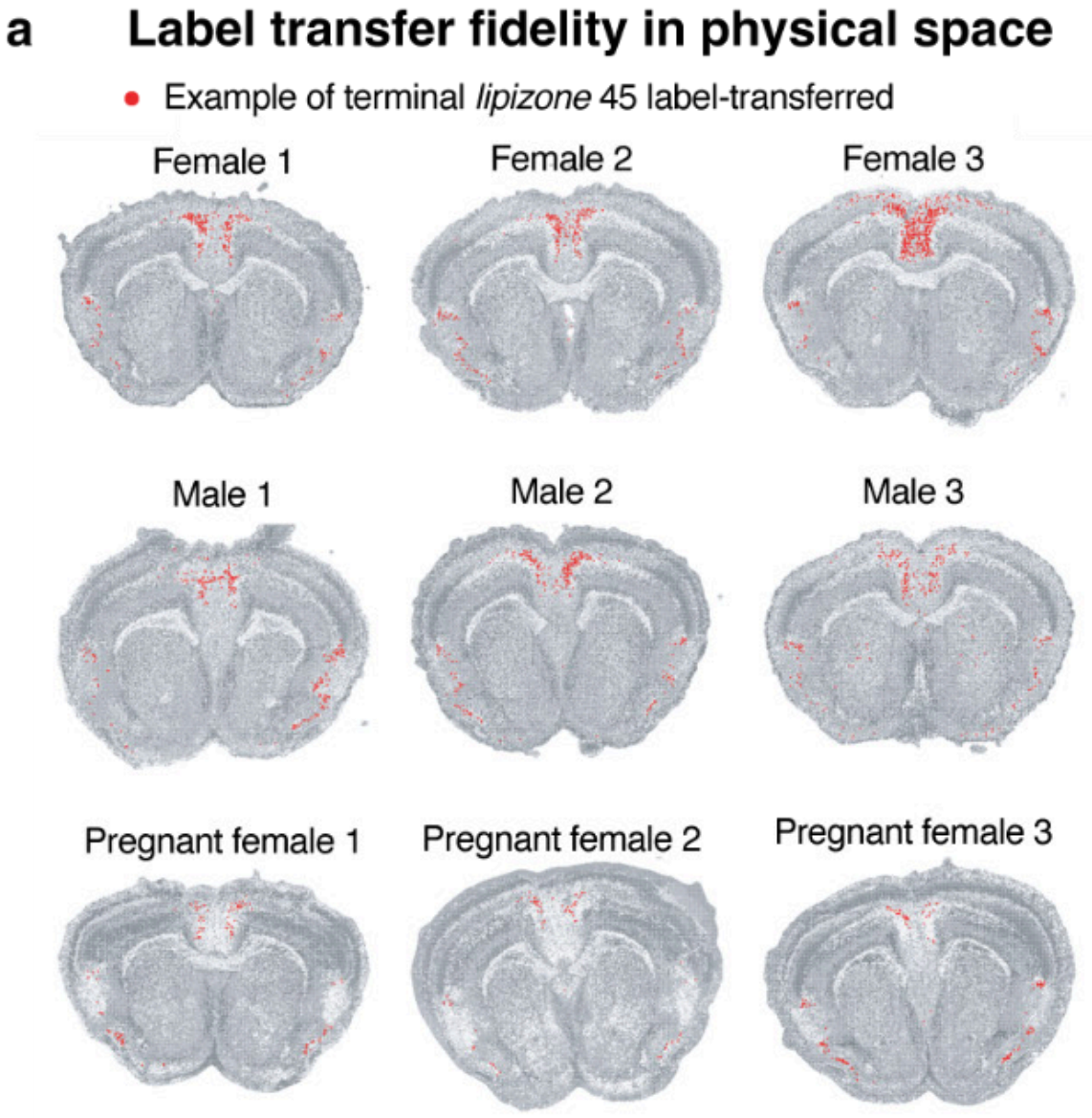


(Supplementary figure 4**a**) Spatial plot of a terminal example *lipizone* transferred across individuals showing spatial consistency.

***Demonstrated utility for perturbation analysis.*** *Lipizones are not only descriptive units but also effective analytical units for detecting biological change. We have shown quantitatively that the lipizone model outperforms anatomical regions in capturing pregnancy-induced lipidomic variance, despite having fewer degrees of freedom (222 vs 397 categories; Supplementary Fig. 21l; see also R3.1). Lipizones, transferred (without double-dipping) provide a better granularity for lipid change modelling than anatomy. We now state in the Discussion:*

*In the **DISCUSSION, P7**, we now state that*

> *"We found large-scale lipid adaptations in the brains of mid-gestation pregnant females, with changes in the cortex possibly related to signaling, and myelin production, corroborated by orthogonal experimental methods. These fine-grained biochemical inferences represent the first application using the Lipid Brain Atlas to drive biological discovery, and showcase the utility of lipizones for future studies on both normal physiology, such as aging and dietary effects, and disease conditions."*

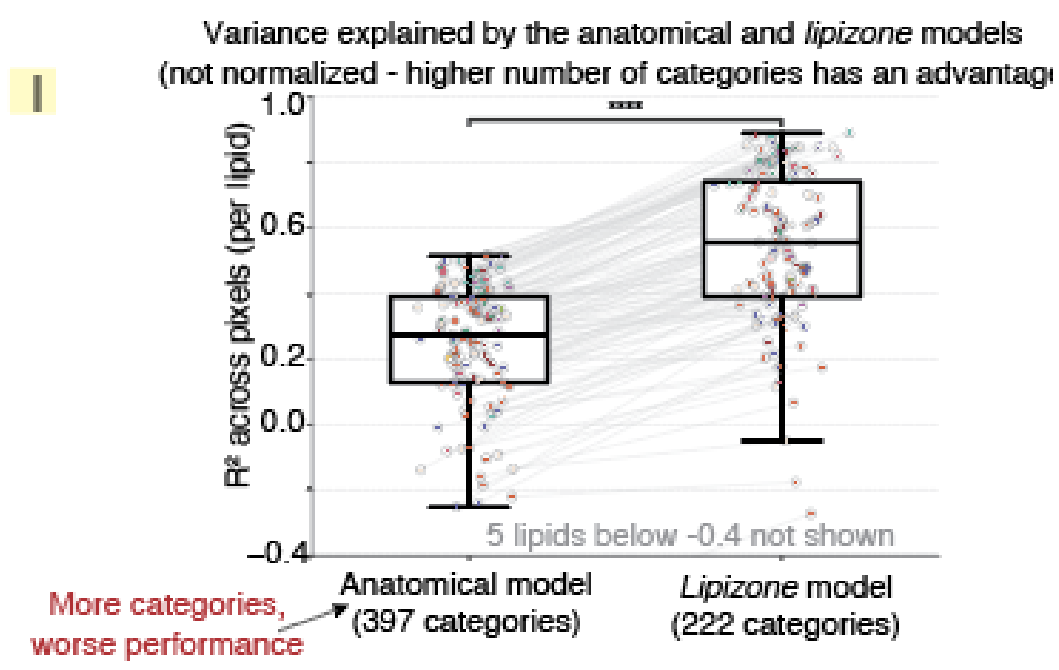


(Supplementary figure 21**l**) Box plots with overlaid strip plots showing the fit quality ($R^2$ across pixels) for individual lipids under two models: an anatomical model based on 397 Allen Brain Atlas acronyms and the *lipizone* model based on 222

supertypes. Each dot represents one lipid, coloured by lipid class; line segments connect the same lipid across the two models. Five lipids with $R^2 < -0.4$ under both models fall outside the displayed y-axis range.

*The pregnancy model itself serves as a worked example of how lipizones can be interrogated: we combine feature importance analysis, signaling modeling via the extended CORNETO framework, and experimental validations to move from lipizone-level observations to hypotheses to biological discovery (**see R2.4**). This strategy not only led to discoveries in our case but also provides a roadmap for future investigations using the atlas.*

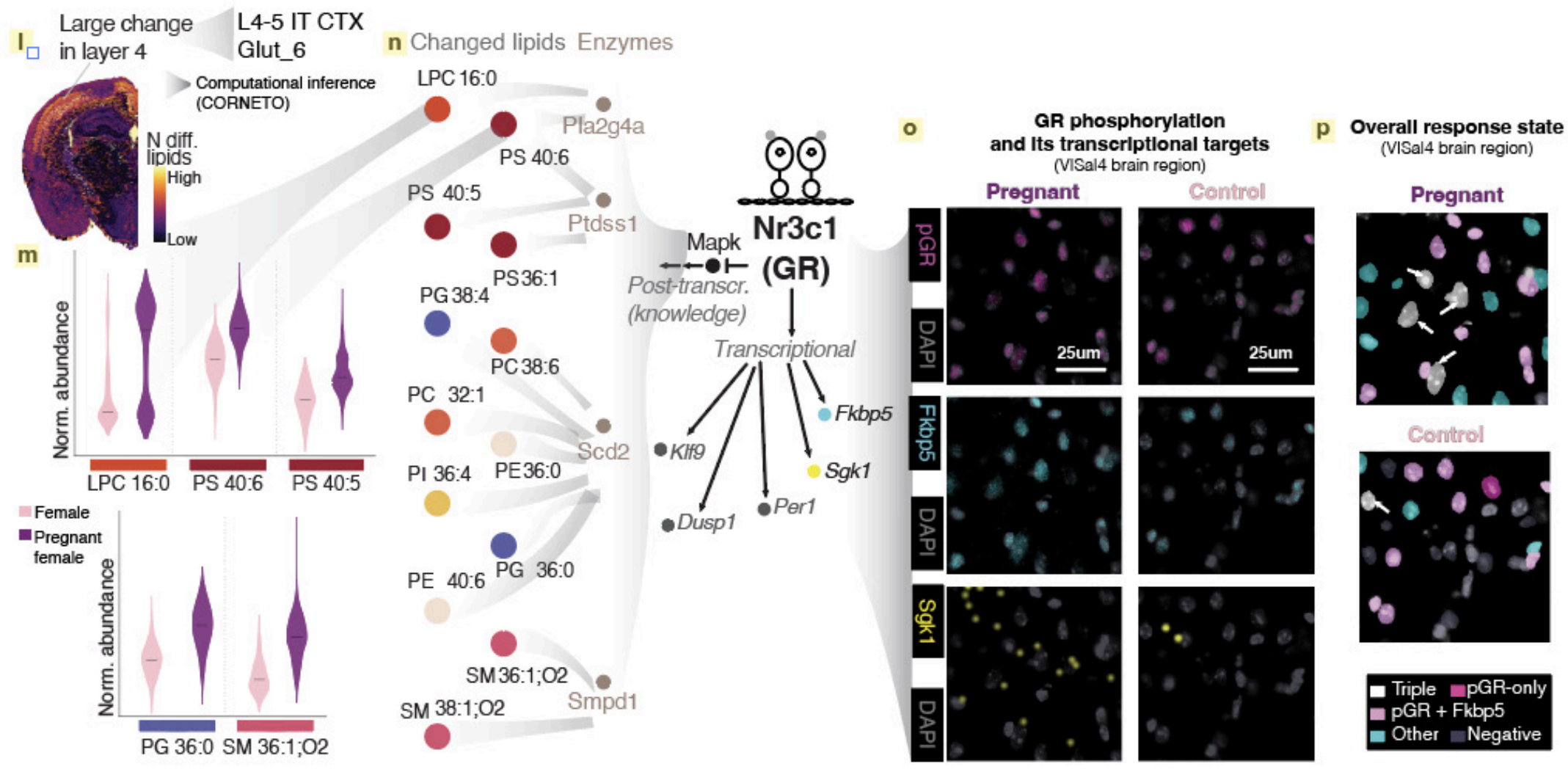


(Figure 6**l**) Representative spatial distribution plot where each pixel is colored by the number of lipids changed in pregnancy. (**m**) Violin plots showing the normalized abundance of 5 example lipids in non-pregnant (pink) and pregnant (purple) subjects. (**n**) Schematic showing the results of the CORNETO analysis tracking the lipid changes back to a putative signaling origin, for the most affected *lipizone*, L4-5 IT CTX Glut. (**o**) Immunostaining (pregnant vs. control) for glucocorticoid receptor phosphorylation and its transcriptional targets in cortical layer 4. DAPI is shown in light gray. (**p**) The same micrograph in (o) but with cells segmented and categorized based on their levels of GR phosphorylation, Fkbp5 intensity, and Sgk1 puncta density. Triple positives for GR phosphorylation and its two transcriptional targets are highlighted with arrows.

***Website as an explorative resource.*** *The Lipid Brain Atlas explorer enables any researcher to interrogate lipid distributions across the entire mouse brain, overlay cell type annotations, run differential analyses between conditions, and visualize lipid architectures in 3D. Researchers can visualize a lipid of interest to guide the selection of Allen brain regions or manually draw regions and compute differential lipids on the fly (Rebuttal Figure 5). They can also select anatomical regions and visualize lipids in 3D (Rebuttal Figure 6). The explorer is designed as an engine for hypothesis generation, letting any lab mine the brain spatial lipidome without specialized computational infrastructure.*

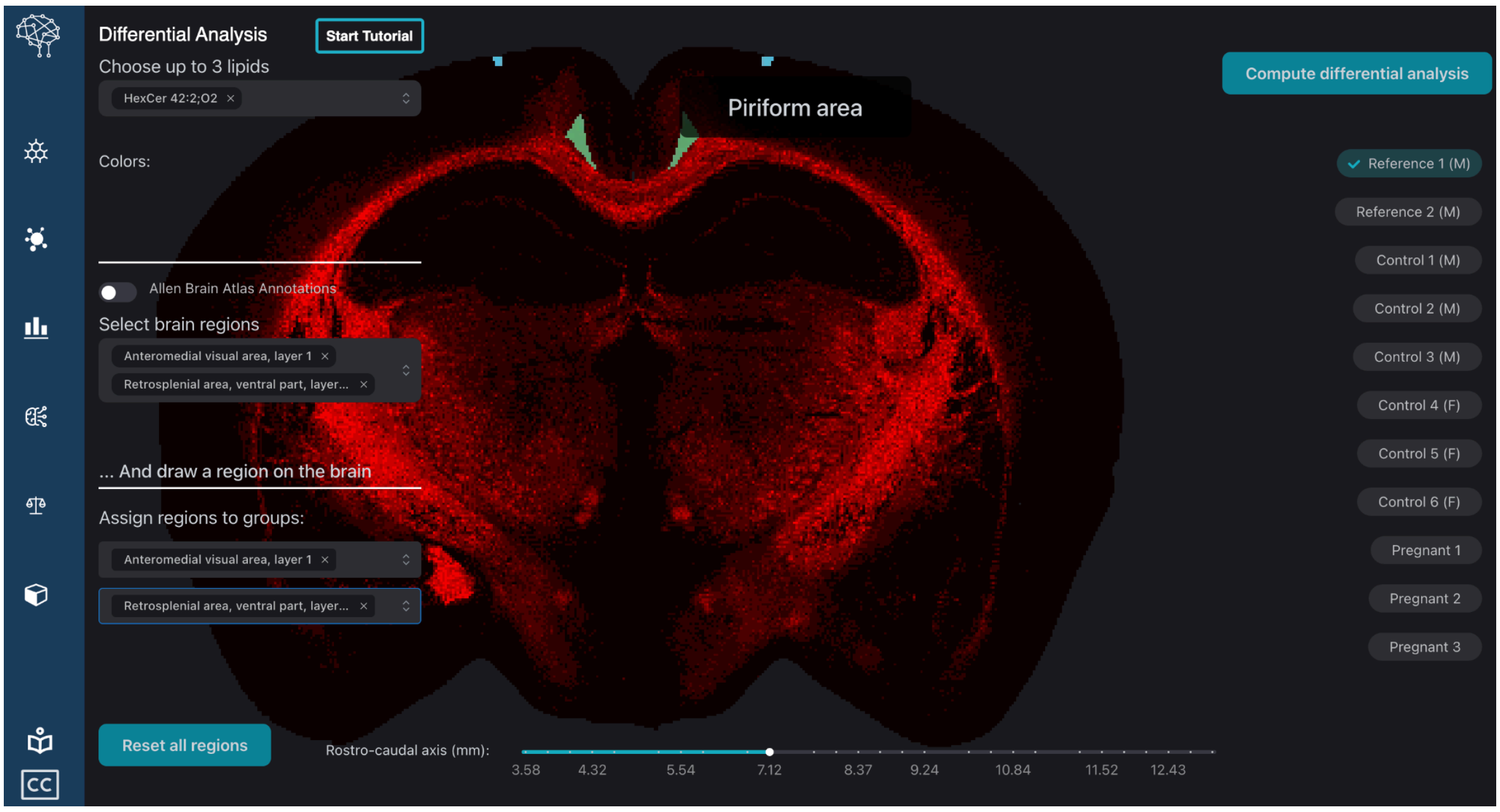


(**Rebuttal figure 5**) Screenshot of the lipid brain atlas explorer in differential analysis mode.

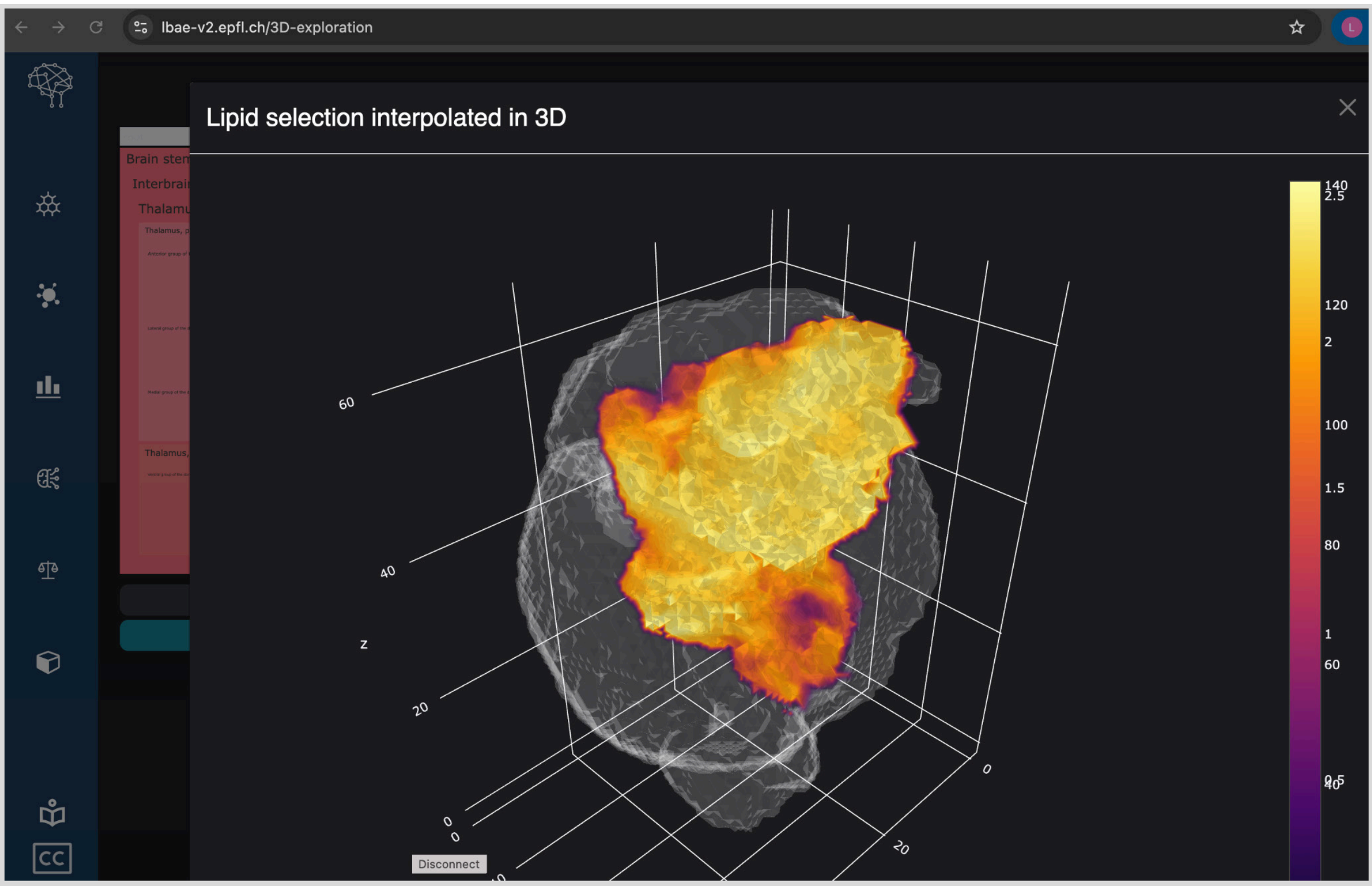


(**Rebuttal figure 6**) Screenshot of the lipid brain atlas explorer in 3D analysis mode, visualizing a myelin lipid in the brain stem.

## References

Bhaduri, Aparna, Elizabeth K. Neumann, Arnold R. Kriegstein, and Jonathan V. Sweedler. 2021. “Identification of Lipid Heterogeneity and Diversity in the Developing Human Brain.” *JACS Au* 1 (12): 2261–2270.

Fitzner, Dirk, Jakob M. Bader, Horst Penkert, et al. 2020. “Cell-Type- and Brain-Region-Resolved Mouse Brain Lipidome.” *Cell Reports* 32 (11): 108132.

Gopalan, Anusha B., Lisa van Uden, Richard R. Sprenger, et al. 2024. “Lipotype Acquisition during Neural Development Is Not Recapitulated in Stem Cell-Derived Neurons.” *Life Science Alliance* 7 (5): e202402622.

Hayden, Benjamin Yost, Sarah R. Heilbronner, and Seng Bum Michael Yoo. 2026. “Rethinking the Centrality of Brain Areas in Understanding Functional Organization.” *Nature Neuroscience* 29 (2): 267–278.

Langlieb, Jonah, Nina S. Sachdev, Karol S. Balderrama, et al. 2023. “The Molecular Cytoarchitecture of the Adult Mouse Brain.” *Nature* 624 (7991): 333–342.

Oh, Seung Wook, Julie A. Harris, Lydia Ng, et al. 2014. “A Mesoscale Connectome of the Mouse Brain.” *Nature* 508 (7495): 207–214.

Rodriguez-Mier, Pablo, Martin Garrido-Rodriguez, Attila Gabor, and Julio Saez-Rodriguez. 2025. “Unifying Multi-Sample Network Inference from Prior Knowledge and Omics Data with CORNETO.” *Nature Machine Intelligence* 7 (7): 1168–1186.

Schede, Halima Hannah, Leila Haj Abdullah Alieh, Laurel Ann Rohde, et al. 2025. “Unified Mass Imaging Maps the Lipidome of Vertebrate Development.” *Nature Methods* 22 (9): 1981–1994.

Surma, Michał A., Mathias J. Gerl, Ronny Herzog, Jussi Helppi, Kai Simons, and Christian Klose. 2021. “Mouse Lipidomics Reveals Inherent Flexibility of a Mammalian Lipidome.” *Scientific Reports* 11 (1): 19364.

Xie, Yuxuan Richard, Daniel C. Castro, Stanislav S. Rubakhin, Timothy J. Trinklein, Jonathan V. Sweedler, and Fan Lam. 2024. “Multiscale Biochemical Mapping of the Brain through Deep-Learning-Enhanced High-Throughput Mass Spectrometry.” *Nature Methods* 21 (3): 521–530.

Yao, Zizhen, Cindy T. J. van Velthoven, Michael Kunst, et al. 2023. “A High-Resolution Transcriptomic and Spatial Atlas of Cell Types in the Whole Mouse Brain.” *Nature* 624 (7991): 317–332.

## Point-by-point response to Referee #1 (round 2)

We thank Referee #1 for the careful re-reading and for the recommendation to publish. We have addressed every remaining point below. Referees #2, #3 and #4 indicated that all earlier concerns were satisfactorily addressed; we have no additional changes to report in response to them.

---

### Referee #1, point 1 — PI / PG confirmation in positive ion mode

*"The confirmation of PI and PG by comparing to negative mode data is not necessarily supporting their detection in positive ion mode, as they ionize much better in negative mode. This is evident from Figure 2d where distributions of things like PI 38:7, PI 40:7 and PG 40:4 are different across positive and negative ion mode. The authors do acknowledge these are likely not detected in positive ion mode, but I feel this should be stronger to really make clear these identifications are likely something else. The best way to confirm would be with on-tissue MS/MS in positive ion mode where class specific headgroup losses should be observed. Ideally this should be done for at least 1 or 2 representative PI and PG lipids to support their presence in the data used to define lipizones. In the absence of this a stronger caveat statement would be good."*

We have now attempted to perform on-tissue MS/MS in positive ion mode on 2 selected PI and 2 PG species, looking for class-specific headgroup losses. The signals were all weak; no lipid confounder (e.g. PC) could be confidently ascribed to the recorded spectra. Our data do not confirm the original annotations. The positive-mode peaks are therefore most likely isobaric signals of other origin as suggested by the referee. We have accordingly removed the two PI species that appeared in main-text marker lists (PI 36:4, PI 42:8); the surrounding claims hold on the remaining lipids, and PG is named in no main-text claim. We can make this inconclusive data available if requested, but we opted not to show them, but rather add strong caveat statements around PIs and PGs.

---

### Referee #1, point 2 — PA 38:5 and PA more generally

*"The new data to suggest PC fragments lead to the 'PA' identifications is good to include. The revised text on page 5 of the response suggests the PA 38:5 does not arise from PC fragmentation. Do the authors know where this comes from, as if it was a PA it should show up in the negative mode LC-MS in Table S2? It is also possible they arise from fragmentation of other classes like PS. If the authors believe some PAs are detected by MALDI this should be confirmed with MS/MS on tissue in positive mode. If a loss of 124 is observed, this will confirm they are instead PC fragments."*

The reviewer's interpretation of Supplementary Fig. 2c is inverted: PA 38:5 is explicitly flagged as ambiguous (red point) because its signal most likely derives from PC

fragmentation; we therefore agree that PA 38:5 is not a genuine PA, and SF2c shows exactly this. Regarding Table S2, PAs were not part of our LC–MS panel, so their absence there is non-informative; we have corrected Methods language to avoid misunderstandings on this point. Positive evidence that the remaining PAs are real comes from Supplementary Fig. 2a: class- and region-wise, our positive-mode MALDI matches the independent negative-mode LC–MS dataset of Fitzner et al. (2020), with negative mode being standard for PA detection, thus supporting bona fide brain PAs. Because PS could in principle yield PA-like ions, we repeated the SF2c spatial-mismatch test; the PA–PS mismatch exceeds the PA–PC mismatch, making PS an even less likely source; this plot is now part of Supplementary Fig. 2c. Finally, no main-text conclusion hinges on any single PA species.

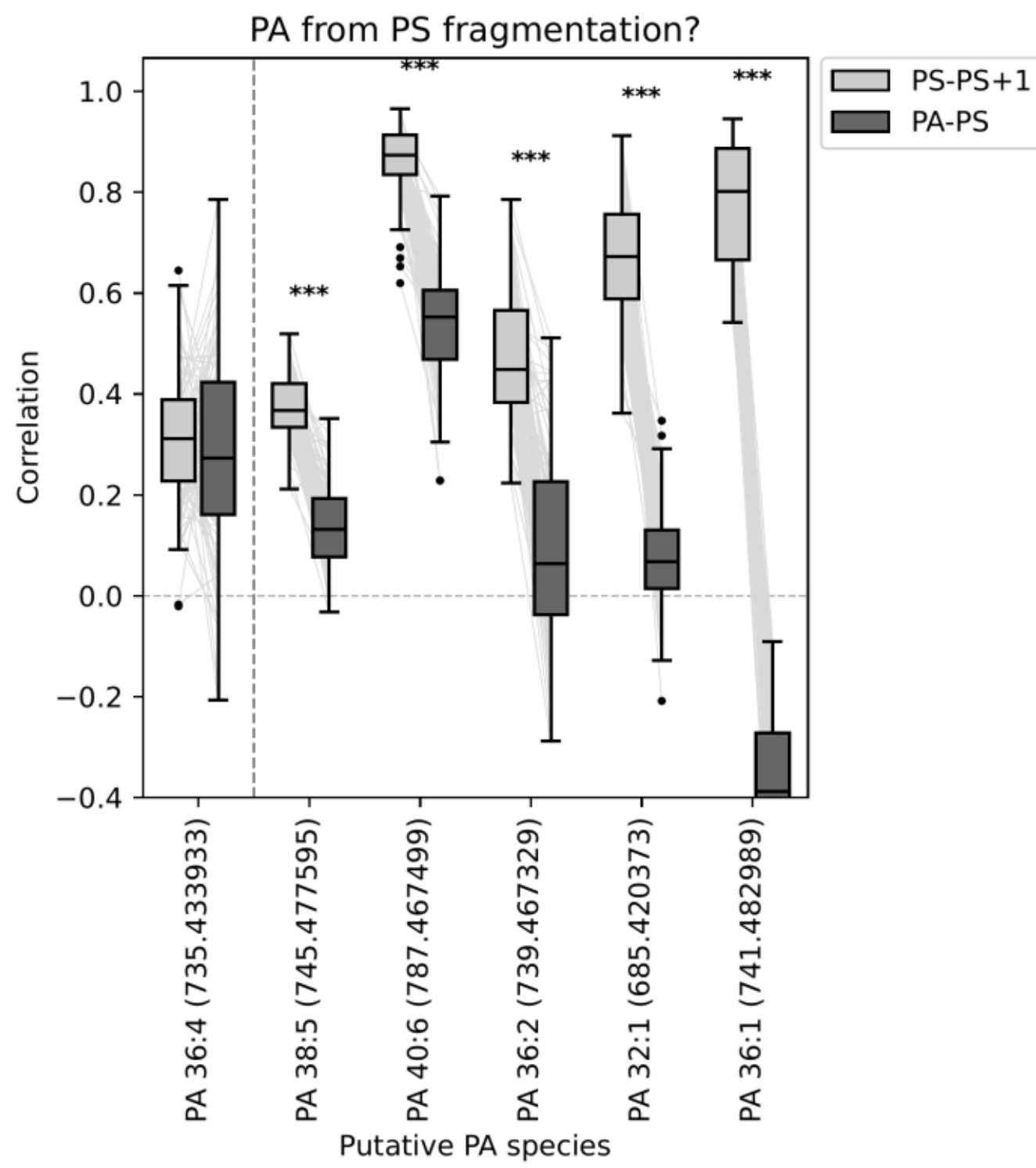


---

## Referee #1, point 3 — explain the PC → "PA" mechanism to the reader

*"As the text now includes the caveat that many PAs arise from PC fragmentation it would be good to include to the reader how this is the case. Essentially loss of NC3H9 from PC leads to an ion isomeric with PA."*

We have added the following to the Methods (Lipid annotation subsection) and the Supplementary Figure 2 legend:

> "In positive ion mode, a phosphatidylcholine that loses its entire choline moiety through in-source fragmentation yields an ion isobaric with the protonated

> phosphatidic acid of the same acyl composition; at the MS1 resolution of imaging experiments the PC in-source fragment and a genuine PA are therefore indistinguishable."

We use "entire choline moiety" rather than "loss of NC3H9" because loss of trimethylamine alone gives an ion 26 Da heavier than the corresponding PA; exact isobaricity with $[PA+H]^+$ requires loss of the whole choline unit (net C5H11N, 85 Da). We are happy to revert to the trimethylamine wording if preferred. We have also cited Vandergrift et al. (Analysis & Sensing 2023), which documents this PC → PA over-annotation in MALDI-MSI.

---

### Referee #1, point 4 — MALDI vs LC-MS correlation method

*"It is not clear how the correlation of MALDI and LC-MS was performed as this cannot be done using ion abundances as they will differ across the two methods. Given the absence of support for many PAs in the LC-MS/MS I wonder if this data is needed or rather the MSI vs LC correlations will just lead to confusion when the point that PAs are largely an artefact should be clear."*

As explained in our response to point 2, only PA 38:5 is a likely artefact; the rest are genuine. The MSI–LC-MS comparison for PA is therefore evidence the PAs are real, not a source of confusion.

The reviewer is right that absolute abundances are not comparable across MALDI-MSI and LC-MS, and we never compared them. For each class we take the Spearman correlation across brain regions of within-class relative abundances between our positive-mode MSI and a reference dataset (Fitzner et al. 2020 for SF2a; our own LC-MS/MS for SF2b). Because this compares regional rank patterns within a single class, the cross-class amplitude differences the reviewer cautions against do not apply. We have added a sentence to the Methods stating this is a within-class, rank-based correlation of regional relative abundances, not absolute intensities.

The two LC-MS datasets are distinct: Fitzner (SF2a) is negative-mode and contains PA; our own panels in Table S2 do not, since PAs were never searched for. PA's absence from Table S2 is a matter of panel scope, not evidence against the PAs.

---

### Referee #2 — *"The authors have thoroughly addressed all concerns, especially in addressing the pregnancy analyses."*

We thank Referee #2 for the positive assessment. No additional changes required.

### Referee #3 — *"The authors have satisfactorily addressed all the issues raised."*

We thank Referee #3 for the positive assessment. No additional changes required.

**Referee #4 — *"I co-reviewed this manuscript with one of the reviewers who provided the listed reports."***

We thank Referee #4 for co-reviewing the manuscript.
