# Supplementary material for: The lipidomic architecture of the mouse brain
Source: Nature. 2026 Sep 23;658(8134):205–17. doi: 10.1038/s41586-026-11050-0 (PMC13626954; doi:10.1038/s41586-026-11050-0)
Supplement: Supplementary file 4 [file 41586_2026_11050_MOESM1_ESM.docx]

# SUPPLEMENTARY METHODS

### lipiMap model and Lipid Ontology integration

lipiMap is a Variational Autoencoder (VAE) adapted from expiMap(Lotfollahi et al., 2023). The encoder employs a feedforward neural network (four hidden layers: 256, 256, 256, 128 neurons) with layer normalization, ReLU activation, and dropout. The decoder is a single linear layer with weights masked by lipid membership in specific lipid programs (LPs), in order to yield a biochemically interpretable latent space. Input data was stretched to the range [0, 1000] and approximated to the next integer. Training used brain #1 (1.7M pixels) with 10% held out for validation and male/female control brains as the test set. We implemented the network using PyTorch (version 2.2.1), and we trained the model on a single NVIDIA GeForce RTX 4090 GPU. Optimal hyperparameters were determined by random search over the hyperparameter space, and the best-performing configuration was selected by choosing the model with the lowest ELBO loss on the validation set. The Adam optimizer(Kingma & Ba, 2014) was used to optimize the ELBO loss function during training.

To constrain the latent space with biochemical knowledge, we downloaded Lipid Ontology(Molenaar et al., 2019) (LION v2020-08-07) from BioPortal, using four categories of LPs: lipid classes, cellular component, functional categories, and physicochemical properties. For example, some LPs include:

- The lipid class “PC” (phosphatidylcholines)
- The cellular component “mitochondrion”
- The functional category “lipid-mediated signaling”
- The physicochemical property “high transition temperature”

165/172 lipids appeared in more than one LP and were used for downstream analysis. To assess whether each lipid program was sufficiently represented by the lipids detected in this study, we performed bootstrap analysis with 10,000 random samples from 1,176 brain lipids found in LION, calculating a representation score for each program. Specifically, we defined the representation score as the ratio of annotated lipids to total lipids associated with each LION term. Programs with representation scores below bootstrapped median or < 3 lipid members were excluded. The matrix of lipid to program membership was used as the mask for decoder weights, enforcing interpretability by implementing hard lipid membership to LPs. This means that membership cannot be learnt during training, but it is fully pre-defined, using LION. The decoder weights matrix was initialized using PCA loadings and masked with the LP matrix. The tendency of VAEs to generate outputs that regress towards the mean of all inputs was mitigated through a weighted MSE reconstruction loss for training:

$WMSE\left( X,\hat{X} \right)=\frac{1}{N}\sum_{i=1}^{N} (X_{i,:}-\hat{X}_{i,:})^{2}\odot\left( 1+k W_{i,:} \right)$

where Wij = 1 if Xij is an extreme value, Wij = 0 otherwise, and k is a tunable parameter. Briefly, for each lipid species, extreme values were defined as those below its lower q-th quantile or above its upper (1-q)-th quantile, with quantiles precomputed across the entire training dataset. We enforced a direct relationship between the sign of latent coordinates and their contribution during linear reconstruction by the decoder, specifically, negative latent coordinates should correspond to lower associated lipid levels, while positive coordinates should lead to higher. To obtain this behavior, decoder weights were clipped within the range [0, 1], so that a deactivated LP (i.e., negative latent coordinate) cannot combine with a negative weight to paradoxically produce a positive contribution.

### Multi-omics integration

We used a whole-brain MERFISH dataset (from Yao et al.(Yao et al., 2023) - and the 8460-genes imputed dataset provided by the same paper) to assess whether some *lipizones* overlapped spatially with cell type territories and to compare lipid and gene expression distributions. This dataset was warped into the same coordinate framework as the Lipid Brain Atlas, namely, the ABA CCFv3. For each pixel of the Lipid Brain Atlas, we evaluated whether any cell was available in the MERFISH dataset within a neighborhood of radius of 50 μm in 3D, after filtering out immune and vascular cells. If at least one cell was available, a cell type territory match was putatively assigned to the pixel by majority voting across the cells of the neighborhood. For gene expression, we averaged across the cells in the neighborhood. To avoid spurious matches across the main anatomical borders due to imperfect registration, we allowed neighborhoods to be established only within the main anatomical regions. Next, to filter well matched sections, we evaluated the overlay between gene *Mog* and lipid HexCer 42:2;O2, as both mark myelin. The resulting 53 sections were uniformly distributed along the anteroposterior axis. Analogously, we considered a Slide-seq2 whole-brain dataset (from Langlieb et al (Langlieb et al., 2023)) to validate our results. To directly compare with this dataset, we used STAlign(Clifton et al., 2023) to warp the Slide-seq2 sections onto the Lipid Brain Atlas matched sections (assessed by physical proximity in CCF space), using the Slide-seq2 Nissl images. We retained ~120k Slide-seq2 beads marked as not containing a doublet of cells, and generated an imputed full transcriptome (21,877 genes) using the snRNA-seq data from the same study. The lipidome was evaluated on each Slide-seq2 bead as the profile of its closest MALDI pixel. Next, for each cell type, we averaged the lipidome of the beads belonging to that cell type. For the lipid-enzyme correlation analysis, gene expression data is derived from the scRNA-seq dataset by Langlieb et al.(Langlieb et al., 2023) and MALDI-MSI lipid data are normalized at the pixel level using uMAIA(Schede et al., 2025); genes and lipids are averaged at the cell type level. Finally, Pearson’s correlation coefficients are calculated using 1,619 cell types (those with >20 usable cells across sections) as data points.

### Prediction of lipid levels from gene expression

To predict lipids from genes, we trained linear models and XGBoost regressors using mean squared error as the loss, using spatial pixels with MERFISH data and cell type averages with Slide-seq2 data. We trained a canonical correlation analysis (CCA) model on 538 MERFISH-imputed metabolism-related genes and our 172 lipids. Redundancy was calculated as the mean squared Pearson’s R between each canonical axis derived from the CCA and each lipid. This metric represents the fraction of lipidomic variance captured by each gene-derived canonical variate, and summing across components yields the cumulative redundancy, indicating the total variance in the lipidome explained by the leading gene–lipid association modes.

We also trained XGBoost models to predict a leave-1-out lipid from all the other lipids, with the same parameters of gene to lipid models, in order to estimate the irreducible fraction of variance *F* for each lipid, where:

$F=1-\frac{R_{g2l}^{2}}{R_{l2l}^{2}}$

with Rg2l is the Pearson’s R between ground truth and reconstruction for the genes to lipid prediction and Rl2l refers to the same score for the lipids to leave-1-out lipid prediction of the same target lipid.

### Feature importance analysis on full transcriptome

Cell type-specific gene enrichment was calculated as previously described in Zeisel et al.(Zeisel et al., 2018) as:

$E\left( i,g \right) = \left( \frac{{mean}_{i,g}}{{mean}_{g}} \right)^{\alpha}\times\left( \frac{{freq}_{i,g}}{{freq}_{g}} \right)^{\beta}$

across the 8460-dimensional MERFISH-imputed transciptome dataset. Mean and freq represent expression level and detection frequency for gene *g* in cell type *i* versus all cells, defaulting to α=β=1. Genes were assigned to cell types based on maximum enrichment scores above a defined threshold, excluding vascular and immune populations.

We trained an XGBoost regressor for each of 172 lipids on the z-scored 8,460-dimensional transcriptome (predictor). To characterize the genes that contribute the most to predicting lipids, we extracted the SHAP feature importances(Lundberg & Lee, 2017) for each gene, for each lipid prediction, on every pixel. SHAP values were then averaged within 251 ABA regions (requiring minimum pixel coverage), converted to absolute values to capture feature importance magnitude, and normalized for cross-lipid comparability. To assess whether genes with the highest feature importances for lipids prediction were enriched for known cell type markers, we performed a one-sided permutation test (B=2,000) on the top 20 SHAP-ranked genes per lipid-region pair. For each pair, we compared the mean marker rank of the top 20 genes against a null distribution generated by randomly sampling 20 genes without replacement. Raw p-values were corrected for multiple testing using Benjamini-Hochberg FDR (α=0.05).

### Colocalization of *lipizones* and cell type territories

*Lipizone* overlap with cell type territories was defined as *lipizone*-cell type colocalization in terms of reciprocal enrichment (the enrichment of *lipizones* in cell types multiplying the enrichment of cell types in *lipizones*).

Specifically, let:

$I=|lipizones|, J=|cell-types|$

and for lipizone 𝑖 and cell‐type 𝑗 let:

$n_{ij}=| x:\ell\left( x \right)=i, c\left( x \right)=j|, N_{i\cdot}=\sum_{j=1}^{J} n_{ij}, N_{\cdot j}=\sum_{i=1}^{I} n_{ij}$

Then the two directional enrichments are:

$E_{ij}^{\left( \ell\mid c \right)}=\frac{\frac{n_{ij}}{N_{\cdot j}}}{\frac{1}{I}\sum_{i'=1}^{I} \frac{n_{i'j}}{N_{\cdot j}}}$

$E_{ij}^{\left( c\mid\ell\right)}=\frac{\frac{n_{ij}}{N_{i\cdot}}}{\frac{1}{J}\sum_{j'=1}^{J} \frac{n_{ij'}}{N_{i\cdot}}}$

and the reciprocal enrichment score is the element-wise multiplication:

$R_{ij}=E_{ij}^{\left( \ell\mid c \right)}\times E_{ij}^{\left( c\mid\ell\right)}$

A threshold of reciprocal enrichment (= 200) and a threshold on the number of pixels where a given cell type and a given *lipizone* were overlapping (= 100) were chosen empirically to decide that a *lipizone* matches a cell type territory.

### Deconvolution of cell type lipidomes

For each pixel, we identified the 1 to 4 nearest reference cells from the Allen MERFISH data in CCF space within a 0.05-unit radius, and used inverse-squared distance weighting to assign a cell type composition profile. The neighbor limit reflects the expected number of cell bodies within a 5 µm pixel.

To evaluate whether the deconvolution generalized beyond regions with common cell type compositions, we designed a challenging held-out test set enriched for compositionally atypical regions through a two-step procedure. We constructed region × cell type count matrices by aggregating cell type assignments across all pixels within each anatomical region. We identified "exclusive" cell types ( those with more than 100 counts in exactly one region and fewer than 100 elsewhere) and assigned their host regions to the training set. We then quantified compositional similarity between remaining regions using cosine similarity on their cell type profiles. For each region, we computed its mean similarity to its five most compositionally similar regions. Regions meeting both criteria: no exclusive cell types and mean neighbor similarity more than one standard deviation below the global average; were designated as the held-out test set..

We modeled lipid abundance as a linear combination of cell type abundances using non-negative elastic net regression. For each lipid species, we fit: B ≈ AX where A is the pixel × cell type matrix, X is the cell type × lipid coefficient matrix (constrained to be non-negative), and B is the pixel × lipid abundance matrix. For each lipid, we performed grid search over 25 hyperparameter combinations: five L1 penalties (λ₁) logarithmically spaced from 10⁻³ to 10¹, and five L2 penalties (λ₂) including zero (pure LASSO) plus four values logarithmically spaced from 10⁻³ to 10¹. Hyperparameter selection was based on 5-fold cross-validation performance on the held-out test regions. The best hyperparameters were then used to refit the model on the full training set. Model performance was evaluated by the R² score. We compared the deconvolution-derived lipidomes with the bulk lipidomics dataset of Fitzner et al., which provides lipid abundances for primary cultured neurons, astrocytes, and oligodendrocytes. Because MALDI ionization efficiency and shotgun extraction efficiency introduce class-level biases that prevent direct comparison of absolute values, we computed cell-type c specificity for each lipid l as the fraction of its total signal attributable to each cell type across the C cell types:

$spec\left( l,c \right)=\frac{abundance\left( l,c \right)}{\sum_{c\in C} abundance\left( l,c \right)}$

For each broad cell class, we matched a MALDI-derived cell type class to Fitzner cell type by selecting among the subclasses of that class whose lipid enrichments (in terms of Spearman's R) best matched the Fitzner et al. reference. For glutamatergic neurons, because the Fitzner et al. cultures derive from E16.5 cortical tissue and are likely mixtures, we restricted candidates to cortical subtypes and also considered averages of k = 1, 2, 3, or 4 subtypes. To identify lipids whose cell-type enrichment differs between *in vivo* and *in vitro* contexts, we performed rank-based outlier detection: within each lipid class, lipids were ranked independently by MALDI and Fitzner et al. specificity, and the absolute rank difference was normalized by class size. Lipids exceeding the pooled median plus 2.5 times the median absolute deviation were flagged as discordant. The Spearman correlation was then recomputed after excluding discordant lipids.

### Mapping of rat and human embryo single-cell lipidomics data

To compare *lipizones* with rat brain single-cell lipidomics data, we downloaded the dataset of Xie et al..(Xie et al., 2024) and retained only lipids in common with our study. We aligned the rat data to our atlas by quantile matching the marginal distribution of each lipid to that of the corresponding species in matched brain regions, followed by batch correction(Korsunsky et al., 2019) We then reclustered the rat single-cell data and computed a centroid lipid profile for each rat cluster. Each mouse atlas pixel was assigned to the rat cluster with the highest cosine similarity to its lipid profile. The same procedure was applied to human embryonic brain single-cell lipidomics data from Bhaduri et al.(Bhaduri et al., 2021), restricted to cortical samples.

### *Lipizone*-informed dissection tables

To obtain *lipizone*-informed anatomical dissection tables, we constructed a tailored parcellation of the CCFv3 by assigning each Allen anatomical ID a *lipizone* class label by maximum overlap on the Lipid Brain Atlas. To mimic dissections of 2D slices in any orientation (eg, at a given bregma position), we can hence simulate slices from the 3D CCF, using this tailored parcellation based on *lipizone* classes (henceforth, territories).

The dissection guides are then obtained on a slice-basis, by topology-informed cleaning:

- Small territories entirely surrounded by another territory are merged with that surrounding territory

- Remaining small territories are merged into the adjacent territory with which they share the largest boundary

- Iteratively, connected components are extracted for each territory, merging the remaining small/fragmented components to yield the final territories

We released this dissection guide generator as a python script in our EUCLID package. These tables indicate, for each *lipizone* class, which brain regions should be sampled together to form a territory with minimal *lipizone* class intermixing.

### Connectomic data and statistical analysis

To test whether highly connected region pairs share on average at least one *lipizone* above chance levels, we integrated three matrices: the ipsilateral connectomic strength matrix (C), a physical distance matrix (D), calculated from the ABA regional centroids, and a region by region *lipizone* similarity matrix (S). C was downloaded from Oh et al.(Oh et al., 2014); S was obtained as

$X^{T}X$

, where X is the binarized colocalization matrix between *lipizones* and Allen anatomical acronyms using reciprocal enrichment as the score. Highly connected pairs were defined as those exceeding the 95th percentile of C, and the observed test statistic (T_obs) was computed as the difference in the proportion of pairs sharing at least one *lipizone* between highly connected vs other pairs. To account for the confounding effect of spatial proximity, we estimated the conditional probability of high connectivity as a function of physical distance by binning D and computing the fraction of pairs meeting the connectivity threshold within each bin. Connectivity labels were then resampled for each pair from a Bernoulli distribution parameterized by this estimated probability, to preserve the marginal distribution of inter-regional distances. Repeating this procedure for 1,000 iterations generated an empirical null distribution for T, against which T_obs was compared using a one-tailed test; the p-value was calculated as the fraction of simulated T values that equaled or exceeded T_obs. The same qualitative results held when using the contralateral, instead of the ipsilateral, connectivity matrix, and when varying binning and connectivity thresholds. To evaluate for each pair of connected regions whether one or more *lipizones* could explain the connection, we scored each connected region pair by the minimum of the *lipizone* fraction between the two regions (requiring this fraction to be > 1%); we then assessed significance by a one-sided structured permutation test computing a null distribution for 8 inter-region proximity bins and 5 whole-brain *lipizone* prevalence bins to account for these two confounders. After sampling a region pair, candidate *lipizones* were conditionally sampled from the pool of *lipizones* present in both regions of the pair, and restricted to those belonging to the given prevalence bin. Empirical p-values were BH-FDR-corrected.

### Integration of cell type and connectomic data to analyze cell terminals

We devised a strategy to assess whether a *lipizone* might contain both the cell bodies and the terminals of a neuronal population. We focused on the *lipizones* that were detected as substantially overlapping with one or more cell type territories based on the MERFISH dataset. We extracted the pixels belonging to a given *lipizone*, and clustered them in the space of their 3D spatial coordinates using kMeans clustering with k = 2. For each *lipizone*, we then asked whether one of these two *lipizone* subclusters was largely responsible for the overlap with the corresponding cell type territory in the MERFISH data. Since MERFISH is a nucleus-based technique, a *lipizone* spatial subcluster overlapping > 80% with the corresponding MERFISH territory suggests a cell body subcluster. To further support the observation that a *lipizone* might contain two spatial subclusters representing the cell bodies and the terminals of a cell type, we looked for axonal streams connecting these two subclusters. To do so, we used the whole-brain mesoscale Allen connectome(Oh et al., 2014). This connectome, downloaded via API using the MouseConnectivityCache, traces axons from 2000+ viral tracer injection sites through the entire volume of the mouse brain at submicrometric resolution. Every tracer injection site is available as a different channel, and it represents a connectivity stream. We binarized the connectome intensities (signal above 95-percentile, independently for each channel) to capture whether a stream passes through a pixel or not, and we evaluated the connectome on the positions where we measured a lipidomic pixel. *Lipizone* spatial subclusters were putatively assigned as containing cell body and terminal territories when 1+ connectomic streams passed through both spatial subclusters, with the two subclusters at opposite extremes of the stream, as assessed using the first eigenvector of the spatial coordinates of all points spanned by the stream.

### Cumulative curves for regional specificity of *lipizones*

To chart the correlation between *lipizones* and fine-grained anatomy, we used a cumulative distribution plot, showing the number of unique anatomical regions needed to reach a percentage of the total *lipizone* area (e.g., if a *lipizone* has 30% of its pixels in region A, 50% in region B, and 20% in C, the curve will be: (1, 50%), (2, 80%), (3, 100%)). Allen Brain Atlas acronyms were preprocessed: cortical region acronyms were collapsed to layer labels (L1 through L6); cerebellar lobule acronyms were merged into a single label; and regions with fewer than 200 pixels across all sections were excluded. Pixels within a radius of 2 units of an anatomical boundary, as well as pixels whose 2-unit neighborhood contained four or more distinct region labels, were removed. For each *lipizone* (or cell type supertype), the number of pixels per anatomical region was tabulated, sorted in descending order, and converted to cumulative percentages. From each curve, we extracted k@75, defined as the smallest number of regions needed to cover 75% of the entity's total area. The same procedure was applied to cell type supertypes from the Yao et al. atlas using the same cleaned region labels. The brain was partitioned into four divisions: dorsal forebrain (isocortex, olfactory areas, cortical subplate, hippocampal formation, striatum, pallidum), ventral forebrain (thalamus/hypothalamus), midbrain, and hindbrain (including cerebellum). Within each division, only entities (*lipizones* or cell types) with at least 400 pixels were retained. *Lipizones* were further split into connectivity-related and non-connectivity-related subsets; ‘Paratrochlear nucleus’ and ‘_1’ were omitted due to their large size. Statistical comparison of k@75 distributions was performed by two-sided Mann–Whitney U test with Benjamini–Hochberg FDR correction (α = 0.05).

### Differential genes along the *lipizone* hierarchy

We performed a systematic differential expression analysis at each binary split in the *lipizone* hierarchy. At each split, we compared gene expression between the two branches using two-sided Welch's t-test, with Benjamini-Hochberg false discovery rate (FDR) correction, with an adjusted p-value threshold of 0.05. A gene was classified as differentially expressed if it had an FDR-adjusted p-value < 0.05 and absolute log₂ fold change > 0.25. To assess whether specific gene sets exhibited coordinated differential regulation at a given split, we then calculated the percent gene set coverage of the DEGs. We focused on 9 broad gene categories: Neurotransmitter and neuropeptide molecules and receptors (306 genes), aggregating genenames.org neuropeptides, their receptors from the LIANA database(Dimitrov et al., 2024), the neurotransmitter-related gene ontology term; Membrane organization (379 genes), from MGI (Mouse Genome Informatics(Baldarelli et al., 2024)); Lipid binding (432 genes), from MGI Metabolic enzymes (580 genes), from gene ontology; Transporter activity (611 genes), from gene ontology; Transcription factors (662 genes), from the SCENIC+ database(Bravo González-Blas et al., 2023); Cell biology (859 genes), obtained by merging classic representatives of a variety of functions: cytoskeleton, ECM/junctions, cell cycle, vesicles, organelles, polarity, migration, cell death, autophagy, flippases and floppases, SNARE complex, synaptic vesicles, and clathrin-mediated endocytosis (mostly from MGI); Ligands and receptors: cell communication (866 genes), from the LIANA database; Cell adhesion: tissue structure (869 genes), from MGI. To enable comparison across nodes with varying numbers of DEGs, the percentage of hits per gene set was z-score normalized within each node. A two-sided Wilcoxon signed-rank test was then applied per depth level × gene set to assess whether the median z-score significantly deviated from zero (i.e., whether a gene set was consistently over- or under-represented relative to the node average at a given depth). Wilcoxon p-values were BH-corrected across all depth × gene-set tests.

### Analysis of white matter clusters in neuronal, astrocyte, and oligodendrocyte feature spaces

We ran four computational experiments to determine whether oligodendrocyte or neuronal or astrocytic lipids (from Fitzner et al.(Fitzner et al., 2020); excluding PG and PI classes, and intersecting oligodendrocyte markers with lipids enriched in the white matter branch and neuron markers with those enriched in the gray matter branch of the *lipizones* tree) drive white matter heterogeneity.

First, for each lipid, we computed η² as the ratio of the sum of squares between clusters and within+between (SS_between / SS_total) using the 65 terminal white matter *lipizones* as the grouping factor, and repeated this at four additional dendrogram depths (2, 4, 8, and 16 groups). Significance was assessed by permuting group labels across lipids (10,000 iterations, preserving set sizes) and computing two-sided p-values for the observed difference in median η² for the three pairwise comparisons.

Second, we retrained a 65-class XGBoost classifier using as the only sets of features the oligodendrocyte-restricted and neuron-restricted lipids. We then used SHAP to extract class-wise feature importances for each lipid, and assessing the relative enrichment of the neuronal-restricted and oligodendrocyte-restricted lipids in the top predictive features set by gene set enrichment analysis (GSEA)(Subramanian et al., 2005).

Third, we subset the pixels belonging to the 65 white matter *lipizones*, and we reclustered, independently on the neuron-specific and oligodendrocyte-specific lipid sets, these pixels using N=10 principal components to avoid feature number imbalance, and proceeding with Leiden clustering. We next compared the 65 *lipizones* with the clusters deriving from these restricted feature spaces computing the confusion matrix, NMI and ARI scores.

Finally, we retrained a 65-class XGBoost classifier as above by using as features the “lipid programs” inferred from our lipiMap model, i.e., its latent space, and extracting the feature importances using SHAP.

### Case-control analysis method development

We formulated a hierarchical Bayesian model to analyze changes during pregnancy or dictated by sex, while accounting for *lipizone* supertypes and potential batch effects. The model incorporates four nested biological variation levels with the following likelihood:

For lipid abundance *xgst* at pixel *g* of supertype *t* in section *s*:

$x_{gst}\sim N\left( \mu_{st},0.1 \right)$

$\mu_{st}=\alpha_{s}+\alpha_{t}+I\left( c_{s}=1 \right)\cdot\beta_{t}$

where *cs* indicates the condition for section *s*. The parameters *αs* and *αt* represent section and supertype (“baseline”) effects, respectively, while *βt* captures the condition effect for supertype *t* (“shift”). The indicator function I(*cs* = 1) is 1 if section *s* is in the pregnant condition, and 0 if it is in the control, non-pregnant condition. The model structure is as follows:

$\alpha_{t}\sim LogitNormal\left( 0,1.0 \right)$

$\beta_{t}\sim N\left( 0,1.0 \right)$

$\mu_{j}\sim N\left( 0,1.0 \right)$

$log\sigma_{j}\sim N\left( 0,5.0 \right)$

$z_{s}\sim N\left( 0,1 \right)$

$a_{s}=\mu_{j\left[ s \right]}+z_{s}\sigma_{j\left[ s \right]}$

$\alpha_{s}=a_{s}-\frac{1}{\left| C_{c_{s}} \right|}\sum_{s'\in C_{c_{s}}} a_{s'}$

where j[*s*] maps section *s* to its parent sample j. We impose a sum-to-zero constraint: where C*cs* denotes sections in condition *cs*. Any global, systematic offset of all pregnant sections versus all non-pregnant ones cannot be distinguished from a batch artifact a priori. By enforcing that the mean section effect is zero in both conditions, we guarantee that the model attributes any consistent difference to the biological “shift” term. Meanwhile, allowing each sample to have its own section‐level standard deviation captures the fact that batch effects manifest at the level of individual slides rather than entire samples.

For inference, we employ a mean-field variational family:

$Q\left( \alpha_{t},\beta_{t},\mu_{j},log\sigma_{j},z_{s} \right)$

$=\prod_{t=1}^{T} Q\left( \alpha_{t} \right) Q\left( \beta_{t} \right)\prod_{j=1}^{J} Q\left( \mu_{j} \right) Q\left( log\sigma_{j} \right) \prod_{s=1}^{S} Q\left( z_{s} \right)$

The variational distribution is parametrized as follows:

$Q\left( \alpha_{t} \right)=LogitNormal\left( \hat{\alpha\mu}_{t},\hat{\alpha\sigma}_{t} \right)$

$Q\left( \beta_{t} \right)=N\left( \hat{\beta\mu}_{t},\hat{\beta\sigma}_{t} \right)$

$Q\left( \mu_{j} \right)=Delta\left( \hat{\mu}_{j} \right)$

$Q\left( log\sigma_{j} \right)=Delta\left( \hat{log\sigma}_{j} \right)$

$Q\left( z_{s} \right)=Delta\left( \hat{z}_{s} \right)$

To prevent spatial autocorrelation to overinflate posterior confidence, data was prepared by ensuring that only one pixel within each 3x3 squared neighborhood would be in the training (and in the test) set, selecting pixels that respected the criterion randomly until pixel exhaustion. The model was implemented in NumPyro(Phan et al., 2019) and fit using stochastic variational inference, estimated through 2,500 Adam optimizer iterations (learning rate=0.01) minimizing the ELBO loss.

Performance evaluation included loss convergence; plateau of parameter traces; Pearson’s correlation between measured and reconstructed supertype centroids by section on training and test pixels; QQ-plots of observed versus posterior predictive distributions; and parameter‐stability tests comparing estimated shifts to simple centroid differences. For inter-individual variation detection, we compared the inferred sample means with the empirical sample averages. We evaluated 15 hyperparameter configurations (varying downsampling ratio, random seed, learning rate, number of epochs, prior scales and guide initialization) on ten lipids spanning a range of batch‐effect severities, exporting visualizations and inspecting each posterior distribution one parameter at a time. Learning curves over 2,500 epochs were assessed to confirm that posterior means and uncertainties reached stable plateaus, with only modest uncertainty increases under downsampling. Pixel‐level XGBoost models Pearson’s Rs between ground truth and fitted, trained on female samples (pregnant and non-pregnant), correlated strongly (Pearson’s R = 0.73) with our hierarchical model’s performance in terms of Pearson’s R, validating its prediction abilities. log2FC was defined as the log2 of the ratio of a supertype baseline plus its shift and the baseline. Significantly shifted lipid-supertype pairs were detected as having >= 98% posterior samples with log2FC > 0.2 or >= 98% posterior samples with log2FC < -0.2.

### Reaction matrix

We constructed a reagent-product-enzyme table using LINEX2(Rose et al., 2023), followed by manual literature-based curation (***Supplementary Table 10***). We defined sixteen biochemical criteria as logical conditions to capture biologically plausible lipid transformations, including: LPX↔PX conversions (maintaining the "X" moiety), PC→PA transitions, PE ↔PC, PE↔PS, LPC→longer-chain LPC, and sphingolipid interconversions (SM↔Cer↔HexCer). These conditions were implemented via string operations and boolean masking, filtering only lipid-lipid pairs meeting at least one criterion. This computational approach yielded putative reactions that matched 100% with manually curated LINEX2 extractions. Each reaction pair was assigned its putative enzyme using a curated lookup dictionary mapping reagent-product combinations to enzymes (e.g., Lpcat1/2 for LPC→PC, Pla2g2a for PC→LPC, Smpd1-4 for SM→Cer).

### Lipid-lipid comodulation analysis in pregnancy

For each of the 172 annotated lipid species, we extracted the Bayesian model parameters for the shift and supertype baseline, and generated 1000 posterior samples using normal distributions with these learned location and scale parameters. Modulation was defined as shifts exceeding 20% of baseline expression. Lipids were classified as increased, decreased, or none when > 98% of samples had shift > 0.2×baseline, or shift < -0.2×baseline, respectively. Expression was evaluated as baseline > 0.05 for > 98% of posterior samples. For each supertype, we calculated the proportion of expressed lipids showing significant regulation (increase or decrease) and computed 95% confidence intervals using bootstrap resampling (10,000 iterations) to assess the reliability of regulation estimates across supertypes. Lipid shits were transformed into log2 fold changes compared to non-pregnant baseline, and hierarchically clustered using cosine distance with a custom metric that handles sparse data by excluding zero-zero pairs and returning maximum distance for zero-norm vectors. Clustering used weighted linkage with optimal leaf ordering, and supertypes were partitioned into 16 clusters using the fcluster algorithm (“comodulation clusters”). To quantify coordinated lipid regulation within metabolic pathways, we computed edge modulation scores by first standardizing fold-change values to Z-scores across supertypes for each lipid, then calculating pairwise modulation strength as the product of absolute Z-scores between metabolically connected lipids. The analysis was restricted to connected components of the metabolic network containing ≥2 lipids, with zero-variance lipids excluded. Cluster-level modulation scores were obtained by averaging edge scores across all supertypes within each comodulation cluster.

### Sphingolipid signature

The sphingolipid signature (ceramides, hexosylceramides, and sphingomyelins) across 3x male, 3x non-pregnant female, and 3x pregnant female brains were computed by first z-score normalizing individual lipid species, then averaging by supertype and condition (male, non-pregnant female, female). This procedure is analogous to the one commonly used for gene expression analysis and implemented in Seurat(Satija et al., 2015). After filtering for supertypes belonging to the core white matter class, the data were row-centered to remove supertype-specific baseline differences, allowing direct comparison of relative lipid abundances across biological conditions. The resulting per-supertype means for each condition were visualized using violin plots, connecting individual supertypes with line segments from male to non pregnant female, and from non pregnant female to pregnant female.

### Feature importance analysis for pregnancy

To identify gene expression and connectivity predictors of pregnancy-associated lipid changes, we trained XGBoost regression models, using SHAP (SHapley Additive exPlanations) for feature importance analysis. As predictors, we used the NMF factors derived from Allen regional pseudobulks of the MERFISH transcriptome-imputed dataset; as targets, we used the lipid log₂ fold changes estimated by our Bayesian model. For each NMF factor ranked as important by SHAP, we extracted its leading genes by sorting them by their NMF loading weight and applying a knee-point detection on the cumulative weight curve to threshold informative from non-informative contributors. Overrepresentation analysis against Gene Ontology was then performed on the resulting gene sets. We repeated this analysis using only genes encoding receptors, applied directly to individual genes rather than NMF factors. To verify that the prioritized genes and enriched ontologies were not simply recapitulating markers of the most affected cell types, we identified differentially expressed genes across cell types using a two-sided Wilcoxon rank-sum test with Benjamini–Hochberg FDR correction and performed Gene Ontology enrichment on those markers separately for comparison. The same XGBoost–SHAP framework was applied to connectivity features, constructed by concatenating the ipsilateral incoming, ipsilateral outgoing, contralateral incoming, and contralateral outgoing mesoscale connectome matrices from Oh et al.(Oh et al., 2014).

### *CORNETO* model to interpret differential lipids

To prioritize receptor-to-enzyme signaling pathways that could explain the differential lipid profiles observed in pregnancy, given cell type-specific expression of receptors and metabolic enzymes, we extended the CORNETO method. The method identifies putative receptors and intermediate signaling proteins whose cell type-specific expression would parsimoniously drive the locally-observed lipid changes, jointly across lipids. The aim is to look for receptor-signaling-enzyme-lipid pathways that can simultaneously explain why specific lipids would increase and others decrease in a *lipizone*. To do so, we first subselected cell surface and intracellular receptors that have predictive power on the multivariate lipid changes brought about by pregnancy or are known to be related to pregnancy and metabolism (see paragraph on Feature importance analysis for pregnancy**)** and have soluble ligands such as well-known endocrine hormones. Then, Omnipath was used to extract PTM-based signaling protein-protein interactions, along with SIGNOR to also account for non-protein signaling molecules.(Lo Surdo et al., 2023; Türei et al., 2025) An exhaustive reaction network encompassing the measured lipid species was manually curated starting from a draft obtained from the Rhea database and the acyl chain composition information from the paired LC-MS dataset. The final network includes the signaling interactions, transcription factor connections to lipid metabolic enzymes parsed from Omnipath and Dorothea, and the manually-curated enzyme-lipid relationships. Node weights were assigned to this prior knowledge graph as the percentage of cells expressing a gene in the *lipizone* of interest. The output score passed to CORNETO was the lipid log2FC upon pregnancy; the input score was the product of the global receptor activity proxy score and the 0-1-normalized receptor expression in the *lipizone* of interest. The global receptor activity proxy score was computed, for each receptor, as the mean of its top-k Pearson correlations (ranked by absolute magnitude) with lipid log₂ fold changes across all *lipizones*. This yields one score per receptor that reflects its strongest cross-target relationships. The knowledge graph was pruned to keep only nodes and edges relevant for the lipids changed in the *lipizone* of interest, and the regularization parameter lambda was decided by sweeping a range of plausible parameters, selecting, among the lambda values that achieved the minimum error, the one producing the fewest active edges, and then taking the smallest such lambda. The indirect_rule_penalty parameter was set to zero after a sweep revealed that it had no effects. The lambda parameter for further analysis was pinned at 0.0001. To mitigate the partial instance-dependence of the solutions, the CORNETO solver was called 40 times to enumerate near-optimal alternatives by randomly removing a small fraction of edges (graph dropout), solving the same objective, and accepting solutions within ε of the baseline objective. The frequency of each edge detected as active across the 40 alternative solutions was chosen as the score for edge activation. The method was run on 46 *lipizones* that have a prevalent supertype and match the territory of an individual cell type. To assess the significance of the call set, a null distribution was generated by permutation. Specifically, the same workflow was run 1000 times using as the output score a vector drawn by randomly permuting entries of the entire log₂FC matrix across both lipids and *lipizones*. Input scores were generated by block-permutation of receptor and node-weight features: hierarchical clustering was applied to the correlation matrix of these features to identify co-varying blocks, and features within each block were permuted jointly to preserve their internal correlation structure. Finally, each edge in each *lipizone* was scored as the frequency of that edge across the 40 near-optimal solutions, and a p-value for that edge was calculated as the proportion of permutations yielding the same or higher frequency on that specific edge. Such p-values were then used as the main statistics, since they account for both the frequency of edge activation across near-optimal solutions and the likelihood of observing such frequency by chance; an effect size score was then calculated as the z-score of the p-values for a given edge across *lipizones*, and zeroed-out when p-value > 0.05. For every *lipizone*, next each receptor-to-lipid shortest path on the subgraph obtained from the significant edges was scored as the mean of its traversed edge scores; a receptor score was obtained by summing these receptor-to-lipid scores across all lipids that are explained by that receptor, so that receptors explaining more lipid targets receive higher aggregate scores.

### Regional bulk LC-MS Bayesian analysis

To quantify pregnancy-induced lipid changes in microdissected corpus callosum (4 non-pregnant and 4 pregnant female mice), we fit a hierarchical Bayesian model with per-lipid pregnancy effects drawn from a shared zero-centered normal prior and observations modeled as Student's t-distributed. Raw nmol lipid abundances were first rescaled using internal lipid class standards, then aligned to a robust reference profile (mean of total-normalized non-pregnant samples) by RANSAC(Fischler & Bolles, 1981) regression in log-log space with slope fixed to 1, yielding a per-sample multiplicative scale factor from the intercept. After RANSAC normalization, intensities were z-scored per lipid using the mean and standard deviation of the non-pregnant group, setting the non-pregnant baseline to zero and placing all lipids on a common scale (units of control-group standard deviations). This allows a single hierarchical prior to be shared across lipids without per-lipid intercepts. The likelihood mean is zero for non-pregnant observations and equal to a per-lipid pregnancy effect βₗ for pregnant observations, with βₗ ~ Normal(0, σ_β) as the shared hierarchical prior. Posterior distributions were obtained via MCMC using the No-U-Turn Sampler (NUTS), with 4 independent chains. The posterior pregnancy effects correspond to Glass's Δ.

### Inter-individual variation analysis

We computed intraclass correlation coefficients (ICC) to assess inter-individual variation using a one-way random effects model, where ICC represents the proportion of total variance attributable to between-subject differences relative to within-subject variation. For each *lipizone* subclass and lipid, we calculated ICC values by comparing measurements across three male samples, estimating between-subject (σ²b) and within-subject (σ²w) variance components through analysis of variance decomposition. The ICC was computed as:

$\frac{\sigma_{b}^{2}}{\sigma_{b}^{2}+\sigma_{w}^{2}}$

,

and averaged across subclasses, providing a metric of inter-individual variation for every lipid. We repeated the same procedure across three female samples, and averaged.

### Lipidome2Location model

The Lipidome2Location model is an out-of-the-box MLP (no hyperparameter tuning) to assess spatial predictability from lipidome profiles. The network uses 6 layers with ReLU activation, dropout (0.5, 0.3, 0.15, 0.1, 0.05), and batch normalization, trained for 50 epochs using MSE loss and Adam optimization with learning rate scheduling (ReduceLROnPlateau). The model was trained on 80% of right hemisphere sections of brain #1 with validation pixels, and tested on the remaining 20% and on the fully-held-out brain #2.

### Immunofluorescence analysis

Nuclear segmentation was performed with Cellpose(Stringer & Pachitariu, 2025) with auto-detected cell diameters; for intensity-based nuclear markers (pGR, Fkbp5), mean fluorescence intensity was quantified within nuclear masks. Samples belonging to the same bregma were jointly processed (as they were part of the same sample preparation, staining, and microscopy experiment). For puncta-based markers (Sgk1, Pgr), individual spots were detected with Spotiflow(Dominguez Mantes et al., 2025) using the pretrained model and assigned to cells by mask lookup; per-cell puncta density was computed as spot count normalized by cell area. Cells with nuclear area below 150 px² were excluded as debris. Sections were registered to the CCF via ABBA(Chiaruttini et al., 2025).
For the white matter heterogeneity analysis, per-pixel CNPase, Klk6, and Opalin intensities were downsampled (1:16) into macro pixels. The three channels were min-max normalized and mapped to a ternary color space (magenta = CNPase, cyan = Klk6, yellow = Opalin), with hue encoding marker composition and brightness encoding total expression level. The Alexa Fluor 568 background was masked in regions where the tissue was torn , and DAPI was blended as an anatomical underlay.
For the glucocorticoid receptor signaling analysis, pGR nuclear intensity, Fkbp5 nuclear intensity, and Sgk1 puncta density were visualized as representative 256 × 256 px crops (88 × 88 µm) overlaid on DAPI. Positivity thresholds were determined by Otsu thresholding on the log-intensity histograms. Per-region mean intensities were min-max normalized within each bregma; only regions represented in at least 2 animals per condition were included.

### snRNA-seq analysis

Raw sequencing data were demultiplexed and processed using the Cell Ranger(Zheng et al., 2017) pipeline (version 10.0.0, 10x Genomics). The FASTQ files were aligned to the mouse reference genome assembly (GRCm39-2024-A). Because the input material consisted of isolated nuclei, reads mapping to intronic regions were explicitly included in the Cell Ranger counting parameters. The pipeline yielded between 6,357 and 6,932 recovered nuclei per sample, with a median detection rate of ~3,900 to 4,500 genes per nucleus. Doublets were identified and removed using Scrublet(Wolock et al., 2019) (expected doublet rate 0.06, score threshold determined per sample). Nuclei passing quality filters (≥200 genes, ≥500 UMIs, < 2% mitochondrial reads) were retained, yielding 22,953 nuclei across 24,156 genes. Read counts were normalized to 10,000 counts per cell and log-transformed (log1p). The 2,000 most variable genes were selected using the Seurat(Satija et al., 2015) v3 method applied to the raw count layer. Principal component analysis was computed on scaled expression data (max_value=10) using 50 components. No batch correction was applied, as samples showed well-mixed integration in UMAP space and as evaluated by scIB(Luecken et al., 2022) metrics. Cell type annotation (and evaluation of its confidence) was performed using MapMyCells(Yao et al., 2023), assigning each nucleus a class, subclass, supertype, and cluster label. Small clusters and clusters with poor quality or poor annotation confidence were removed. Ambient RNA was removed with CellBender(Fleming et al., 2023) v0.3.0 (remove-background, 150 epochs, expected false-positive rate 0.01, learning rate 5×10⁻⁵, expected cells 7,000 per sample, total droplets included 25,000). All analyses were repeated on both datasets with and without ambient RNA removal. Differentially expressed genes (DEGs) between pregnant and control nuclei were assessed using the two-sided Mann–Whitney U test (Wilcoxon rank-sum). For each cell population of interest, raw counts were normalized to CP10K. P-values were corrected for multiple testing using Benjamini–Hochberg FDR. To control for ambient RNA artifacts, DE was performed independently on both CellBender-corrected and uncorrected (original) count matrices. A gene was called differentially expressed only if it reached significance (adjusted p < 0.05) in both analyses with consistent direction of effect. Log₂ fold-changes were computed from the CellBender-corrected analysis. Pre-ranked GSEA was run using gseapy(Fang et al., 2023) against three MSigDB v2025.1 mouse symbol collections: Gene Ontology Biological Process (GO:BP), Canonical Pathways, and Reactome; genes were ranked by log₂FC. Over-representation analysis (Fisher's exact test) was performed on intersection DEGs using gseapy.enrich against the same three MSigDB collections. The background gene set comprised all genes expressed in the tested population. A glucocorticoid-response gene signature was constructed from the Juszczak et al.(Juszczak & Stankiewicz, 2018) meta-analysis of 17 transcriptomic studies of GC-treated brain and CNS cells. Genes reported as up-regulated in ≥4 independent studies with zero down-regulation reports were selected, yielding 51 candidates. These candidates were cross-referenced against other independent studies (and manual inspection of the literature): Zannas et al.(Zannas et al., 2019) (CORT-induced and DC4 classical GC component, blood monocytes), Heard et al.(Heard et al., 2021) (chronic cortisol, iPSC-derived astrocytes). Genes reported as down-regulated in any study were excluded; among the remaining, only those independently confirmed in ≥1 external study were retained. Three canonical GR targets were found missing and added: *Per1*, *Sgk1*, and *Errfi1*. Two astrocyte-specific genes were excluded: *Pdk4* and *Aldoc*. Finally, genes were filtered to those expressed, i.e., with ≥10% detection in the cluster of interest in both CellBender and original count matrices, yielding a 15-gene signature. Gene set enrichment analysis was performed using GSEApy prerank with the log₂FC for ranking and 10,000 permutations. Bayesian generalized linear mixed models were fitted per gene using NumPyro to estimate the posterior distributions for the parameters of interest. For DEG count data, a negative binomial model was specified:

$y_{gc}\sim GammaPoisson\left( r, \frac{r}{\mu_{gc}} \right)$

where

$log\left( \mu_{gc} \right)=log\left( L_{c} \right)+\alpha+\beta\cdot x_{c}+u_{s\left[ c \right]}$

with L_c the library size of cell c, x_c a binary condition indicator (1 = pregnant), and

$u_{s}\sim Normal\left( 0, \sigma_{sample} \right)$

a random intercept for biological sample. Priors:

$\alpha\sim Normal\left( 0, 5 \right)$

$\beta\sim Normal\left( 0, 3 \right)$

$\sigma_{sample}\sim HalfNormal\left( 2 \right)$

$r\sim Gamma\left( 2, 0.5 \right)$

NUTS was run with max_tree_depth=8, 500 warmup and 2,000 posterior samples per gene.

### Negative-ion-mode MALDI2 acquisition

For the single coronal section acquired in negative ion mode with MALDI2, 1,5 DAN matrix was used. The matrix was sublimated using an HTX SubliMATE™ (HTX Technologies, Carrboro, NC). 20mg of 1,5 DAN was weighted and resuspended into 1.5 ml of acetone for uniform matrix distribution. Acetone was then allowed to fully evaporate. The lid was closed and the vacuum line opened. Sublimation was performed at 60 mTorr, after prior cooling for 10 min using a water bath with ice; 1,5 DAN was sublimated for 5 min at 180 degrees. MALDI-MS imaging experiments were performed on a TimsTOF flex mass spectrometer (Bruker Scientific, LLC, Bremen, Germany) coupled with a SmartBeam 3D 10 kHz frequency tripled Nd:YAG laser (355 nm). The laser settings used were 5 μm diameter circular spot size, with 500 shots per pixel and a raster step size of 25 μm. The laser power was set to 50%. The MS imaging data were collected over a mass range of 350-1800 Da in negative-ion mode. Ion mobility separation was performed using trapped ion mobility spectrometry (TIMS) in default mode. The TIMS funnel was operated with an ion mobility range of 0.74-1.85 V·s/cm² (1/K₀), with a ramp time of 144.1 ms and an accumulation time of 50.1 ms. The duty cycle was 34.76% with a ramp rate of 6.66 Hz. Data acquisition was performed without post-ionization. The calibration of the mass spec was performed using Red Phosphorus and the TIMS calibration was performed using the ESI-L Low Concentration Tuning Mix (Agilent Technologies).

### *Lipizone* validation by alternative clusterings

We validated the clustering across 25+ alternative regimes and repeated the analyses supporting the major claims of the paper under each regime. To test sensitivity to feature composition, we downsampled lipid features to 60%, 75%, or 90%, and separately re-ran the clustering after including imputed lipids. To test sensitivity to spatial sampling, we downsampled sections to 12, 18, or 24 of the 27 available, equally spaced (resampling). To test sensitivity to algorithmic choice, we replaced the binary splitter with Leiden, Louvain, spectral, and kMeans clustering, and clustered on the embeddings of lipiMap. To test sensitivity to parameter settings, we explored plausible parameter sets around the defaults. To test cross-subject transferability, we clustered Brain #2 independently and transferred its labels onto Brain #1.

Alternative regimes were compared quantitatively by: correlating normalized mutual information (NMI) between each regime and the reported lipizones against variance explained (R²); computing Jaccard similarity and recall of the reported lipizones against clusters found in each regime; quantifying cluster abundance per region; and measuring within-cluster residual heterogeneity (log determinant of the covariance matrix). Replicability of the main findings across regimes was assessed by manual confirmation (***Supplementary data file 2***).

# SUPPLEMENTARY REFERENCES

Baldarelli, R. M. et al. Mouse Genome Informatics: an integrated knowledgebase system for the laboratory mouse. *Genetics* **227**, iyae031 (2024).

Bravo González-Blas, C. et al. SCENIC+: single-cell multiomic inference of enhancers and gene regulatory networks. *Nat. Methods* **20**, 1355–1367 (2023).

Dimitrov, D. et al. LIANA+ provides an all-in-one framework for cell–cell communication inference. *Nat. Cell Biol.* **26**, 1613–1622 (2024).

Dominguez Mantes, A. et al. Spotiflow: accurate and efficient spot detection for fluorescence microscopy with deep stereographic flow regression. *Nat. Methods* **22**, 1495–1504 (2025).

Fang, Z., Liu, X. & Peltz, G. GSEApy: a comprehensive package for performing gene set enrichment analysis in Python. *Bioinformatics* **39**, btac757 (2023).

Fischler, M. A. & Bolles, R. C. Random sample consensus: a paradigm for model fitting with applications to image analysis and automated cartography. *Commun. ACM* **24**, 381–395 (1981).

Fleming, S. J. et al. Unsupervised removal of systematic background noise from droplet-based single-cell experiments using CellBender. *Nat. Methods* **20**, 1323–1335 (2023).

Heard, K. J. et al. Chronic cortisol differentially impacts stem cell-derived astrocytes from major depressive disorder patients. *Transl. Psychiatry* **11**, 608 (2021).

Kingma, D. P. & Ba, J. Adam: a method for stochastic optimization. Preprint at *arXiv* https://doi.org/10.48550/arXiv.1412.6980 (2014).

Lo Surdo, P. et al. SIGNOR 3.0, the SIGnaling network open resource 3.0: 2022 update. *Nucleic Acids Res.* **51**, D631–D637 (2023).

Luecken, M. D. et al. Benchmarking atlas-level data integration in single-cell genomics. *Nat. Methods* **19**, 41–50 (2022).

Molenaar, M. R. et al. LION/web: a web-based ontology enrichment tool for lipidomic data analysis. *Gigascience* **8**, giz061 (2019).

Phan, D., Pradhan, N. & Jankowiak, M. Composable effects for flexible and accelerated probabilistic programming in NumPyro. Preprint at *arXiv* https://doi.org/10.48550/arXiv.1912.11554 (2019).

Rose, T. D., Köhler, N., Falk, L., Klischat, L., Lazareva, O. E. & Pauling, J. K. Lipid network and moiety analysis for revealing enzymatic dysregulation and mechanistic alterations from lipidomics data. *Brief. Bioinform.* **24**, bbac572 (2023).

Satija, R., Farrell, J. A., Gennert, D., Schier, A. F. & Regev, A. Spatial reconstruction of single-cell gene expression data. *Nat. Biotechnol.* **33**, 495–502 (2015).

Stringer, C. & Pachitariu, M. Cellpose3: one-click image restoration for improved cellular segmentation. *Nat. Methods* **22**, 592–599 (2025).

Subramanian, A. et al. Gene set enrichment analysis: a knowledge-based approach for interpreting genome-wide expression profiles. *Proc. Natl Acad. Sci. USA* **102**, 15545–15550 (2005).

Türei, D. et al. OmniPath: integrated knowledgebase for multi-omics analysis. *Nucleic Acids Res.* **54**, D652–D660 (2025).

Wolock, S. L., Lopez, R. & Klein, A. M. Scrublet: computational identification of cell doublets in single-cell transcriptomic data. *Cell Syst.* **8**, 281–291.e9 (2019).

Zannas, A. S. et al. Epigenetic upregulation of FKBP5 by aging and stress contributes to NF-κB–driven inflammation and cardiovascular risk. *Proc. Natl Acad. Sci. USA* **116**, 11370–11379 (2019).

Zheng, G. X. Y. et al. Massively parallel digital transcriptional profiling of single cells. *Nat. Commun.* **8**, 14049 (2017).

# SUPPLEMENTARY FIGURES

**Supplementary Figure 1. Study design, biochemical characterization of the lipids in this study, and feature restoration.** (**a**) Spatial zoom-in representation of MALDI-MSI pixel sampling scheme, highlighting the pixel size and inter-pixel distance, colored by the abundance of the lipid HexCer 42:2;O2. (**b**) Study design. (**c**) Computational processing overview. (**d**) Spatial plot for dataset overview, showing all brain sections colored by cluster (*lipizone*). (**e**) Pie chart for the proportions of lipid classes in terms of number of nonredundant lipids in this study (MALDI-MSI). (**f**) Pie chart for the proportions of lipid classes in this study in terms of number of nonredundant lipids for the paired whole-brain LC-MS. (**g**) Pie chart of nanomolar fractions for each lipid class in LC-MS. (**h**) Histogram for the absolute parts per million (ppm) distance between MALDI-MSI and LC-MS m/z peaks for the 172 nonredundant lipids used for the analysis in this study. (**i**) Nested histograms for carbon chain length and insaturation, for the lipids detected by MALDI-MSI in this study. (**j**) Nested histograms for carbon chain length and insaturation, in for the lipids detected by LC-MS in this study. (**k**) Strip plot of the Moran's I scores for individual lipids in our MALDI-MSI dataset (average across sections), grouped by lipid class. (**l**) Schematic illustrating the XGBoost-based feature restoration strategy that we devised to recover lipid distribution estimates for sections with corrupted measurements. (**m**) Examples with spatial plots and reconstruction scatter plots measured vs restored of a lipid undergoing restoration (PE O-36:3). (**n**) Histogram of XGBoost performance on held-out data across lipids, evaluated by Pearson’s R. (**o**) Barplot for the average fraction of acquisitions with Moran’s I > 0.4, one bar per lipid class.

**Supplementary Figure 2. Comparison MALDI - LC-MS, caveats on annotations, and within-class heterogeneity.** (**a**) Strip plot showing the Spearman’s correlation coefficient between the lipid abundance measured by MALDI-MSI and measured by LC-MS/MS (in an independent study), calculated across individual lipids belonging to each major class (x axis). The analysis is stratified by brain region (coloring; one dot per brain region). (**b**) Barplot of the whole-brain Spearman’s correlation coefficient for the lipid abundance measured by MALDI-MSI and measured by LC-MS/MS (in the whole-brain dataset acquired for this study), stratified by lipid class. In fact, different classes are known to be uncomparable in MALDI-MSI. (**c**) Boxplot pairs for the Pearson’s correlation between (left) a phosphatidylcholine (PC) and its +1 isotopologue (light boxes) and between a phosphatidic acid (PA) and the same-length, same-insaturation PC (dark boxes), calculated on the raw data on each individual section across pixels. n = 10 PA–PC pairs, each evaluated across 100 brain sections; centre line, median; box, 25–75 percentiles; whiskers, 1.5 × IQR. PA annotations with less than 0.2 difference of correlation PA-PC versus PC-PC+1 are labelled in red as possibly ambiguous. Light gray line segments connect same-section correlation values between the box pairs. (Right) Same boxplots, comparing PA-PS correlations against PS-(PS+1) correlations. (**d**) Split-brain spatial views for PG and PI lipids; the left half shows negative ion mode data, where PG and PI are typically detected; the right half is the best-matched section from the Lipid Brain Atlas, which is in positive ion mode. (**e**) Spatial plots for all the PC lipids in the Lipid Brain Atlas on a representative section, sorted by the (**f**) lipid-lipid correlation matrix (heatmap).

**Supplementary Figure 3. Label transfer, *lipizone* nomenclature, and inter-sample controls.** (**a**) Spatial plot of a terminal example *lipizone* transferred across individuals showing spatial consistency. (**b**) t-SNEs computed from the lipidome, highlighting the same example *lipizone* as in (a), across individuals. (**c**) Frequency histograms of Pearson's R for Allen region pairs between brain #1 and brain #2 in terms of *lipizone* enrichments and *lipizone* pairs in terms of Allen regions to explore fine-grained spatial consistency between the two brains after label transfer. (**d**) Heatmap of sample-sample correlation in terms of *lipizone* lipidomes and heatmap of sample-sample correlation in terms of *lipizone* proportions. (**e**) Heatmap of the normalized confusion matrix comparing reclustering and label transfer on brain #2. (**f**) Spatial plots for the *lipizone* hierarchy and naming convention at three hierarchical levels. (**g**) Histogram of the frequency distribution of the number of pixels per *lipizone*, pooling all the 11 brains.

**Supplementary Figure 4. Validation of *lipizones* with alternative clustering methods and across subjects.** (**a**) Set of plots showing metrics to evaluate the consistency of the findings reported throughout the manuscript for alternative clustering regimes (rows): from the left heatmap showing for each cluster the for the Jaccard index with the matching lipizone (columns, see leaf coloring), heatmap for the recall of *lipizones* (columns, see leaf coloring), barplot of the number of clusters, binary heatmap reporting whether thediscovery (columns) is reproduced under each of the clustering regimes for the fraction of brain sections where a given cluster is found, and the residual variance by cluster (lower means more variability is captured by the partition). (**b**) Thumbnails of the clustering reports for each clustering regime that is provided as a separated ***Supplementary data file 2***. (**c**) Scatterplot showing for each clustering regime the fraction of explained variance as a function of the similarity of that clustering with with our reference lipizone model . Note how the more a model is similar to ours explain better the variance in the data. (**d**) Heatmap of the pairwise spatial agreement of *lipizone* distribution across individual brains measured as Pearson’s correlation of the vector of relative *lipizone* abundances of each Allen Brain Atlas regions.

**Supplementary Figure 5. Mosaic plots for different brain regions.** (**a**) Half-brain spatial plots spaced along the rostrocaudal axis, colored by *lipizone*, highlighting their spatial organization.

**Supplementary Figure 6. *Lipizones* spatial overviews by anatomical division and by lipidomic subclass.** (**a**) Spatial plots of *lipizones* by brain division. (**b**) Spatial plot of the 31 lipid-derived subclasses of the brain for two representative sections.

**Supplementary Figure 7. Visual comparison of the Allen Brain cell type atlas and the lipid brain atlas.** (**a**) Half-brain spatial transcriptomics parcellation and half-brain spatial lipidomics parcellation, colored by subclass-level cell types, and *lipizones*, respectively.

**Supplementary Figure 8. Multimodal analysis and organizational principles of *lipizone* distribution.** (**a**) Redundancy‐scree plot showing per‐component and cumulative lipid variance explained by metabolic gene‐derived canonical variates (canonical component analysis using the Allen 8460-genes imputed transcriptome and the lipid brain atlas, matching voxels in physical space). (**b**) Frequency histogram of BH-corrected FDRs for cell type marker gene overrepresentation in the top 20 important features for the XGBoost gene to lipid regression, across all lipids and anatomical regions, showing that, for the large majority of lipid-region pairs, cell type markers are largely enriched in the top important features set. (**c**) Spatial plot examples of SHAP feature importance maps for the gene to lipid XGBoost regression, averaged across all lipids. Shown are selected well-known cell type regional marker genes. (**d**) Example spatial plots of *lipizones* and the cell type territories they overlap with. (**e**) Zoom-in spatial plots for the cerebellar granule cells layer, detected as two major layered *lipizones*, colored by two lipids that were differential between the inner and outer layer *lipizones*.

**Supplementary Figure 9. Approximation of cell type lipidomes by deconvolution.** (**a**) Heatmap of the deconvolved cell type lipidomes; colour represents relative abundance. Columns are Allen subclass-level cell types; rows are individual lipid species. The coloured bar at the top annotates each cell type by the anatomical region it is most enriched in. (**b**) Spatial plots of selected lipids coloured by abundance, comparing native MALDI measurements (first row) with lipidomes reconstructed by cell type deconvolution using the closest MERFISH section (second row). Lipids were chosen to illustrate the diversity of reconstruction qualities. (**c**) Bar plots of the Spearman correlation between cell type lipid enrichments estimated by deconvolution of the Lipid Brain Atlas and those measured by bulk LC-MS (Fitzner et al.), after outlier removal. (**d**) Pie charts showing the fraction of discordant lipids (red) excluded from each LC-MS versus MALDI comparison because largely and consistently diverging between tissue and *in vitro* cell types.

**Supplementary Figure 10. Cross-species comparison of lipidomic clusters.** (**a**) UMAP of single-cell lipid profiles from the developing human cortex (Bhaduri et al.), with selected clusters highlighted in colour. Right: spatial mapping of the selected clusters onto a half-section of the mouse Lipid Brain Atlas. (**b**) As in (a), for single-cell lipid profiles from the adult rat brain (Xie et al.). (**c**) UMAPs of single-cell lipid profiles from the developing human cortex, coloured by selected individual lipids, with corresponding spatial projections onto the mouse Lipid Brain Atlas. (**d**) As in (c), for single-cell lipid profiles from the adult rat brain.

**Supplementary Figure 11. Mesoscale ipsilateral connectivity matrix annotated by *lipizone* enrichments.** (**a**) Heatmap of the thresholded ipsilateral mesoscale Allen Brain connectome. Regions are sorted by brain division. Non-white entries denote connected region pairs. "x" marks indicate connections whose start and endpoint do not share any *lipizone*. Light grey squares indicate connections whose start and endpoint share *lipizones*, but not significantly (stringently tested against a null model that accounts for other sources that could explain the sharing; *see Methods*). Coloured squares indicate connections whose start and endpoint share *lipizones* significantly, coloured by the dominant shared *lipizone*.

**Supplementary Figure 12. Lipidomic features of the white matter and the ventricular systems.** (**a**) Barplot of myelination and oligodendrocyte scores for each Allen division, sorted and colored by Allen division color. (**b**) Annotated *lipizones* tree for the 65 core white matter-related *lipizones*. (**c**) Spatial plots of example regionalized white matter-related *lipizones*, in addition to those in Figure 4. (**d**) Line plot showing, for each level of the *lipizone* hierarchy, the difference in variance explained by the *lipizone* model for lipids restricted to individual cell types (oligodendrocytes, neurons and astrocytes) relative to the other two cell types in the white matter. Permutation distributions are also shown as bands. (**e**) Gene set enrichment analysis (GSEA) plot showing the ranks of oligodendrocyte and neuronal lipids in the important features set for the 65-class *lipizone* classification task. (**f**) Zoom-in spatial plots for replication of the axonal bundle marker lipids on brain #2. (**g**) Kernel density estimation for replication on brain #2 of the mediolateral pattern of the 65 core white matter-related *lipizones* in the anterior callosum. (**h**) Spatial plot replication of the ChP1, ChP2, and lining *lipizones*, on brain #2. (**i**) Left, barplot of ChP1 and ChP2 marker lipids; right, lightblue-colored barplot showing ether lipid imbalance. (**j**) Scatterplot of the similarity range vs anteroposterior position for whole-brain *lipizones* against ChP1-2, showing the cerebellar *lipizones* omitted from the analysis of Extended Data Figure 9e. (**k**) Spatial plot replication of SM 34:1;O2 as a ventricular marker, on brain #2. (**l**) Zoom-in spatial plot replication of the dorsoventral lipid gradients along the ventricular walls, on brain #2.

**Supplementary Figure 13. Localized variation between sexes and in pregnancy discovered with Bayesian modeling.** (**a**) Spatial plot for the overall change in lipidome between sexes as obtained by summing the net female-male differences across lipids, shown for an anterior section. (**b**) Heatmap of log2FCs for all lipids in all supertypes between males and females. (**c**) Spatial plots of localized lipid differences between males and females, two example lipids in a ventricular *lipizone*. (**d**) Barplot showing the whole-brain average lipid change between males and females for all the lipids in this study, colored by lipid class. (**e**) Schematic of the hierarchical Bayesian model used to detect supertype-specific changes in pregnancy and between sexes, while accounting for interindividual variation and batch effects. (**f**) Histogram of Pearson’s R on *lipizone* centroids for the Bayesian model SVI reconstruction performance across lipids in pregnancy, evaluated as Pearson’s R between the ground truth lipid abundance and its posterior average. (**g**) Scatterplot of the Bayesian model shifts and the naïve empirical differences of average in pregnant mouse brains and average in non pregnant mouse brains, across all lipids and supertypes. (**h**) Scatterplot of lipid-lipid correlation versus the correlations of the same lipid pairs calculated from their changes in response to pregnancy, showing that covariation in response to the pregnancy perturbation does not trivially reflect the baseline lipid covariation patterns in the brain. (**i-k**) Frequency histograms for the average, minimum, and maximum fold-change per lipid across supertypes. (**l**) Box plots with overlaid strip plots showing the fit quality (R² across pixels) for individual lipids under two models: an anatomical model based on 397 Allen Brain Atlas acronyms and the *lipizone* model based on 222 supertypes. n = 172 lipids; centre line, median; box, 25–75 percentiles; whiskers, 1.5 × IQR. Each dot represents one lipid, coloured by lipid class; line segments connect the same lipid across the two models. Five lipids with R² < −0.4 under both models fall outside the displayed y-axis range.

# SUPPLEMENTARY FILES LEGENDS

**Supplementary data file 1. 3D rendering of SM 34:1;O2.** Video showing an example 3D reconstruction of a lipid. All reconstructions are explorable on the companion website.

**Supplementary data file 2. Validation of *lipizones* by alternative clustering methods.** Each page of this file reports the major figures and findings of the paper using an alternative clustering method, as described in the Main text and Methods. The subplots include: a heatmap of the normalized and sorted confusion matrix comparing the *lipizones* reported in the paper and the clusters obtained by the alternative method; a spatial plot with matched color coding for representative sections along the rostrocaudal axis; a heatmap of the normalized and sorted confusion matrix comparing the *lipizones* and the anatomical divisions; a spatial plot of the 4 major brain divisions found by lipidomic clustering; a heatmap of the normalized and sorted confusion matrix comparing the *lipizones* and cell types; spatial plots for four *lipizones* matching cell type territories; zoom-in spatial plots showing cortical lipizones; the histogram with red bar replicating ***Figure 3j***; a stacked barplot and a scatterplot replicating ***Extended Data Figure 9e***; spatial views on the cerebellar *lipizones*; half-brain spatial plots showing example white matter *lipizones*; a violin plot and an accompanying barplot replicating ***Figure 4b***; density plots for the mediolateral distribution of white matter *lipizones*, replicating ***Figure 4l***; spatial zoom-ins of the axon bundle *lipizones* and their differential lipids, replicating ***Figure 4k***.
