# Supplementary material for: The lipidomic architecture of the mouse brain
Source: Nature. 2026 Sep 23;658(8134):205–17. doi: 10.1038/s41586-026-11050-0 (PMC13626954; doi:10.1038/s41586-026-11050-0)
Supplement: Supplementary file 5 [file 41586_2026_11050_MOESM3_ESM.zip › 2025-06-14969CD-s3/LaManno_SuppTable6.pdf]

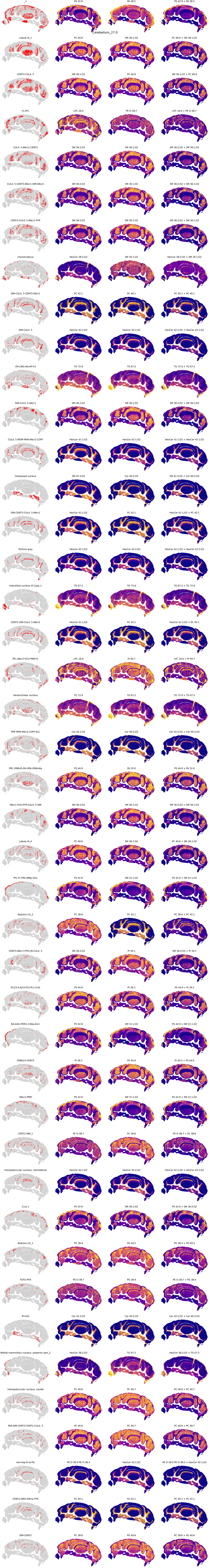

Cerebellum_27.0
_1
PS 42:9
PA 38:5
PS 42:9 × PA 38:5
Lobule III_1
PC 40:6
SM 36:1;O2
PC 40:6 × SM 36:1;O2
CENT3-CUL4, 5
SM 36:1;O2
PC 40:6
SM 36:1;O2 × PC 40:6
FL-PFL
LPC 16:0
PE O-38:7
LPC 16:0 × PE O-38:7
CUL4, 5-ANcr1-CENT3
SM 36:2;O2
SM 36:1;O2
SM 36:2;O2 × SM 36:1;O2
CUL4, 5-CENT3-ANcr1-SIM-ANcr2
SM 36:2;O2
SM 36:1;O2
SM 36:2;O2 × SM 36:1;O2
CENT3-CUL4, 5-ANcr1-PYR
SM 36:2;O2
SM 36:1;O2
SM 36:2;O2 × SM 36:1;O2
choroid plexus
HexCer 38:2;O2
SM 34:1;O2
HexCer 38:2;O2 × SM 34:1;O2
SIM-CUL4, 5-CENT2-ANcr1
PC 42:1
PC 40:1
PC 42:1 × PC 40:1
SIM-CUL4, 5
HexCer 42:2;O2
HexCer 42:1;O2
HexCer 42:2;O2 × HexCer 42:1;O2
OV-LING-AQ-AP-V3
TG 72:9
TG 67:2
TG 72:9 × TG 67:2
SIM-CUL4, 5-ANcr1
SM 36:2;O2
SM 36:1;O2
SM 36:2;O2 × SM 36:1;O2
CUL4, 5-IPDM-PRM-ANcr2-COPY
HexCer 41:1;O2
HexCer 42:1;O2
HexCer 41:1;O2 × HexCer 42:1;O2
Interposed nucleus
SM 42:3;O2
Cer 40:2;O2
SM 42:3;O2 × Cer 40:2;O2
SIM-CENT2-CUL4, 5-ANcr1
HexCer 41:1;O2
PC 42:1
HexCer 41:1;O2 × PC 42:1
Pontine gray
HexCer 42:1;O2
HexCer 42:2;O2
HexCer 42:1;O2 × HexCer 42:2;O2
Interstitial nucleus of Cajal_1
TG 67:2
TG 72:9
TG 67:2 × TG 72:9
CENT2-SIM-CUL4, 5-ANcr2
HexCer 41:1;O2
PC 42:1
HexCer 41:1;O2 × PC 42:1
PFL-ANcr2-VCO-PRM-FL
LPC 16:0
PI 40:7
LPC 16:0 × PI 40:7
Paratrochlear nucleus
TG 72:9
TG 67:2
TG 72:9 × TG 67:2
PRP-PRM-ANcr2-COPY-SLC
Cer 42:2;O2
Cer 40:2;O2
Cer 42:2;O2 × Cer 40:2;O2
PRC-ORBvl5-RH-SPA-ORBm6a
PS 44:9
PA 32:0
PS 44:9 × PA 32:0
ANcr1-UVU-PYR-CUL4, 5-SIM
SM 36:2;O2
SM 36:1;O2
SM 36:2;O2 × SM 36:1;O2
Lobule III_4
PC 40:6
SM 36:2;O2
PC 40:6 × SM 36:2;O2
PFL-FL-TMv-MMp-GU1
PS 42:9
SM 31:1;O2
PS 42:9 × SM 31:1;O2
Nodulus (X)_2
PC 38:6
PC 42:1
PC 38:6 × PC 42:1
CENT3-ANcr1-PYR-CB-CUL4, 5
SM 36:2;O2
PI 34:1
SM 36:2;O2 × PI 34:1
PL2/3-ILA2/3-PL5-PL1-ILA5
PS 44:9
PI 36:3
PS 44:9 × PI 36:3
BA-AId1-PERI1-COAa-AIv1
PS 42:9
SM 31:1;O2
PS 42:9 × SM 31:1;O2
ORBl2/3-CENT3
PI 36:3
PS 44:9
PI 36:3 × PS 44:9
ANcr2-PRM
PS 42:9
SM 31:1;O2
PS 42:9 × SM 31:1;O2
CENT2-SIM_1
PE O-38:7
PC 38:6
PE O-38:7 × PC 38:6
Interpeduncular nucleus, rostrolateral
HexCer 42:1;O2
HexCer 42:2;O2
HexCer 42:1;O2 × HexCer 42:2;O2
Crus 2
PS 42:9
SM 36:2;O2
PS 42:9 × SM 36:2;O2
Nodulus (X)_1
PG 38:4
PG 40:5
PG 38:4 × PG 40:5
FOTU-PYR
PE O-38:7
PG 38:4
PE O-38:7 × PG 38:4
IP-IntG
Cer 42:2;O2
Cer 40:2;O2
Cer 42:2;O2 × Cer 40:2;O2
Medial mammillary nucleus, posterior part_2
HexCer 38:2;O2
TG 67:2
HexCer 38:2;O2 × TG 67:2
Interpeduncular nucleus, caudal
PC 40:6
PC 40:7
PC 40:6 × PC 40:7
PAR-SIM-CENT3-CENT2-CUL4, 5
PC 40:6
PC 40:7
PC 40:6 × PC 40:7
vtd-mtg-VI-sV-RL
PE O-36:0 PE O-38:3
HexCer 42:1;O2
PE O-36:0 PE O-38:3 × HexCer 42:1;O2
CENT2-DMX-SIM-ts-PYR
PC 40:1
PC 42:1
PC 40:1 × PC 42:1
SIM-CENT2
PC 38:6
PG 40:6
PC 38:6 × PG 40:6

Cortical subplate_15.0

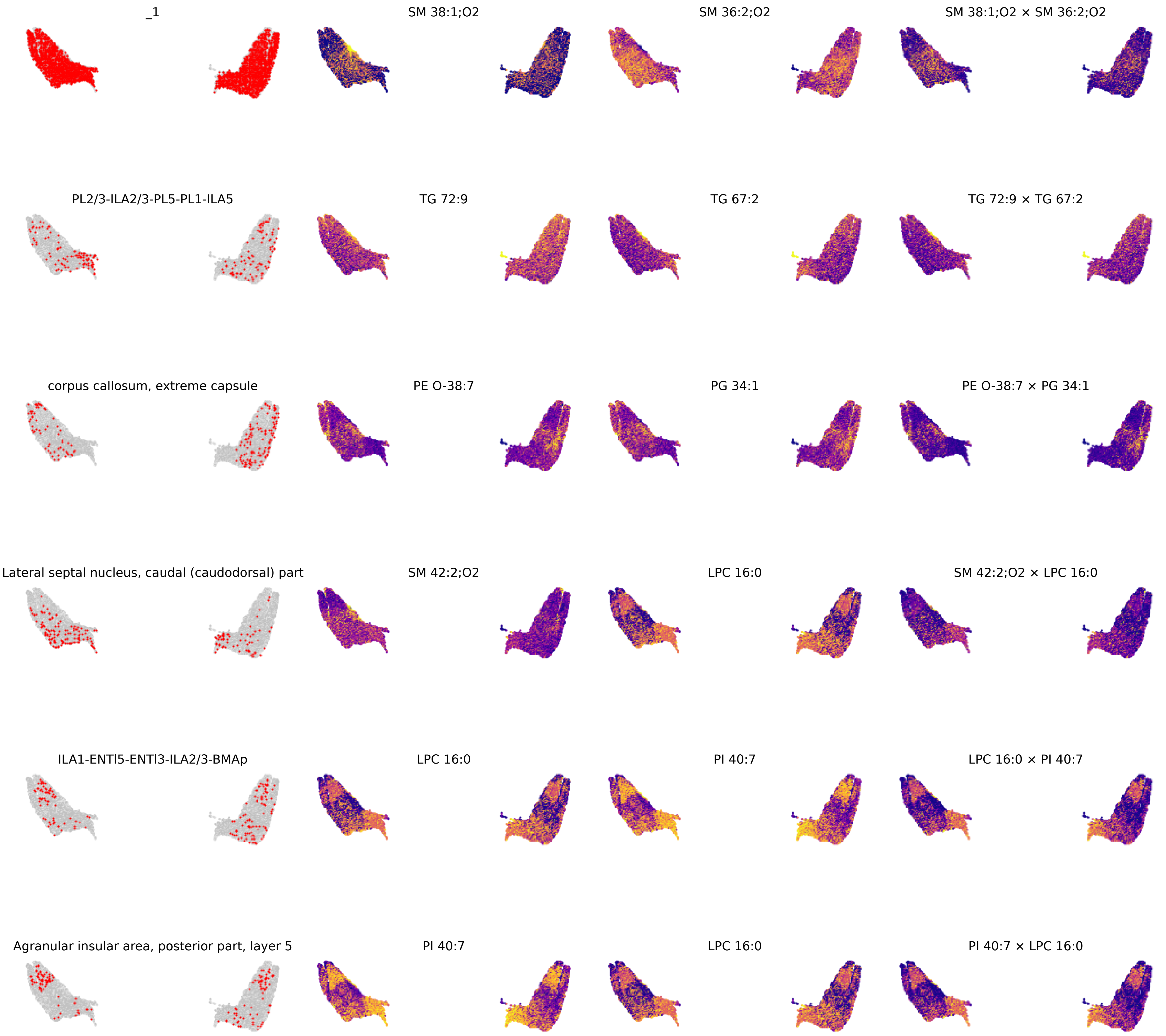

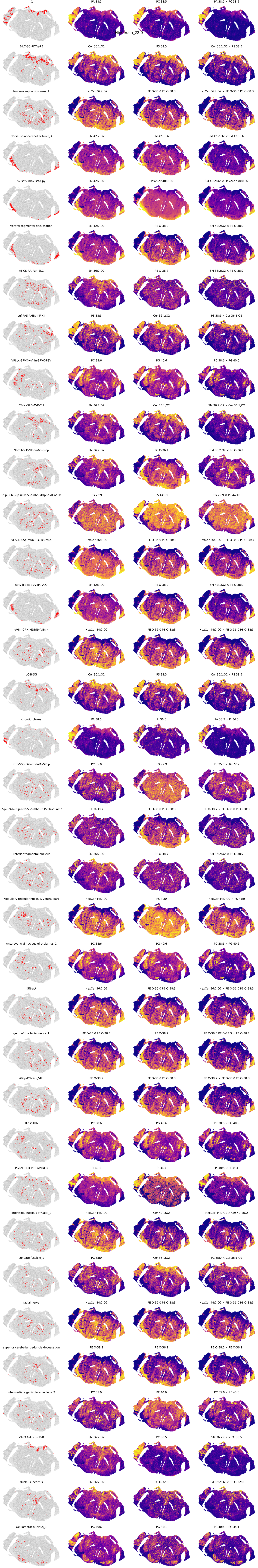
Hindbrain_22.0
_1
PA 38:5
PC 38:5
PA 38:5 × PC 38:5
B-LC-SG-PDTg-PB
Cer 36:1;O2
PS 38:5
Cer 36:1;O2 × PS 38:5
Nucleus raphe obscurus_1
HexCer 36:2;O2
PE O-36:0 PE O-38:3
HexCer 36:2;O2 × PE O-36:0 PE O-38:3
dorsal spinocerebellar tract_3
SM 42:2;O2
SM 42:1;O2
SM 42:2;O2 × SM 42:1;O2
sV-sptV-moV-sctd-py
SM 42:2;O2
Hex2Cer 40:0;O2
SM 42:2;O2 × Hex2Cer 40:0;O2
ventral tegmental decussation
SM 42:2;O2
PE O-38:2
SM 42:2;O2 × PE O-38:2
AT-CS-RR-Pa4-SLC
SM 36:2;O2
PE O-38:7
SM 36:2;O2 × PE O-38:7
cul-PAS-AMBv-KF-XII
PS 38:5
Cer 36:1;O2
PS 38:5 × Cer 36:1;O2
VPLpc-SPVO-vVIIn-SPVC-PSV
PC 38:6
PG 40:6
PC 38:6 × PG 40:6
CS-NI-SLD-AVP-CLI
SM 36:2;O2
Cer 36:1;O2
SM 36:2;O2 × Cer 36:1;O2
NI-CLI-SLD-VISpm6b-dscp
SM 36:2;O2
PC O-36:1
SM 36:2;O2 × PC O-36:1
SSp-II6b-SSp-un6b-SSp-n6b-MOp6b-ACAd6b
TG 72:9
PS 44:10
TG 72:9 × PS 44:10
VI-SLD-SSp-m6b-SLC-RSPv6b
HexCer 36:1;O2
PE O-36:0 PE O-38:3
HexCer 36:1;O2 × PE O-36:0 PE O-38:3
sptV-icp-cbc-vVIIn-VCO
SM 42:1;O2
PE O-38:2
SM 42:1;O2 × PE O-38:2
gVIIn-GRN-MDRNv-VIIn-x
HexCer 44:2;O2
PE O-36:0 PE O-38:3
HexCer 44:2;O2 × PE O-36:0 PE O-38:3
LC-B-SG
Cer 36:1;O2
PS 38:5
Cer 36:1;O2 × PS 38:5
choroid plexus
PA 38:5
PI 36:3
PA 38:5 × PI 36:3
mfb-SSp-n6b-RR-IntG-SPFp
PC 35:0
TG 72:9
PC 35:0 × TG 72:9
SSp-un6b-SSp-n6b-SSp-m6b-RSPv6b-VISal6b
PE O-38:7
PE O-36:0 PE O-38:3
PE O-38:7 × PE O-36:0 PE O-38:3
Anterior tegmental nucleus
SM 36:2;O2
PE O-38:7
SM 36:2;O2 × PE O-38:7
Medullary reticular nucleus, ventral part
HexCer 44:2;O2
PS 41:0
HexCer 44:2;O2 × PS 41:0
Anteroventral nucleus of thalamus_1
PC 38:6
PG 40:6
PC 38:6 × PG 40:6
ISN-act
HexCer 36:2;O2
PE O-36:0 PE O-38:3
HexCer 36:2;O2 × PE O-36:0 PE O-38:3
genu of the facial nerve_1
PE O-36:0 PE O-38:3
PE O-38:2
PE O-36:0 PE O-38:3 × PE O-38:2
AT-fp-PN-cic-gVIIn
PE O-38:2
PE O-36:0 PE O-38:3
PE O-38:2 × PE O-36:0 PE O-38:3
III-cst-TRN
PC 38:6
PG 40:6
PC 38:6 × PG 40:6
PGRNl-SLD-PRP-AMBd-B
PI 40:5
PI 36:4
PI 40:5 × PI 36:4
Interstitial nucleus of Cajal_2
HexCer 44:2;O2
Cer 42:1;O2
HexCer 44:2;O2 × Cer 42:1;O2
cuneate fascicle_1
PC 35:0
Cer 36:1;O2
PC 35:0 × Cer 36:1;O2
facial nerve
HexCer 44:2;O2
PE O-36:0 PE O-38:3
HexCer 44:2;O2 × PE O-36:0 PE O-38:3
superior cerebellar peduncle decussation
PE O-38:2
PE O-36:1
PE O-38:2 × PE O-36:1
Intermediate geniculate nucleus_2
PC 35:0
PE 40:6
PC 35:0 × PE 40:6
V4-PCG-LING-PB-B
SM 36:2;O2
PC 38:5
SM 36:2;O2 × PC 38:5
Nucleus incertus
SM 36:2;O2
PC O-32:0
SM 36:2;O2 × PC O-32:0
Oculomotor nucleus_1
PC 40:6
PG 34:1
PC 40:6 × PG 34:1

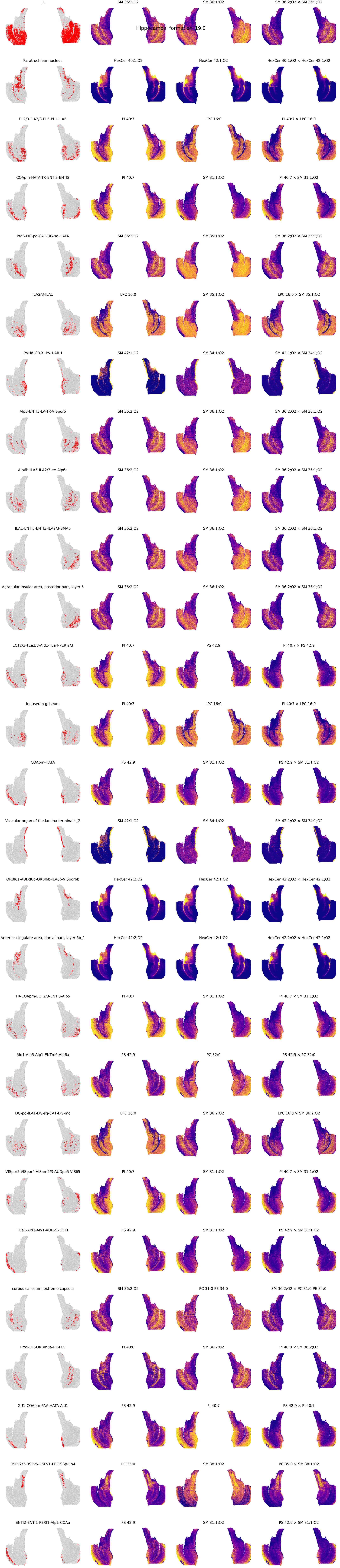
Hippocampal formation 19.0
_1
SM 36:2;O2
SM 36:1;O2
SM 36:2;O2 × SM 36:1;O2
Paratrochlear nucleus
HexCer 40:1;O2
HexCer 42:1;O2
HexCer 40:1;O2 × HexCer 42:1;O2
PL2/3-ILA2/3-PL5-PL1-ILA5
PI 40:7
LPC 16:0
PI 40:7 × LPC 16:0
COApm-HATA-TR-ENTl3-ENTl2
PI 40:7
SM 31:1;O2
PI 40:7 × SM 31:1;O2
ProS-DG-po-CA1-DG-sg-HATA
SM 36:2;O2
SM 35:1;O2
SM 36:2;O2 × SM 35:1;O2
ILA2/3-ILA1
LPC 16:0
SM 35:1;O2
LPC 16:0 × SM 35:1;O2
PVHd-GR-Xi-PVH-ARH
SM 42:1;O2
SM 34:1;O2
SM 42:1;O2 × SM 34:1;O2
AIp5-ENTl5-LA-TR-VISpor5
SM 36:2;O2
SM 36:1;O2
SM 36:2;O2 × SM 36:1;O2
AIp6b-ILA5-ILA2/3-ee-AIp6a
SM 36:2;O2
SM 36:1;O2
SM 36:2;O2 × SM 36:1;O2
ILA1-ENTl5-ENTl3-ILA2/3-BMAp
SM 36:2;O2
SM 36:1;O2
SM 36:2;O2 × SM 36:1;O2
Agranular insular area, posterior part, layer 5
SM 36:2;O2
SM 36:1;O2
SM 36:2;O2 × SM 36:1;O2
ECT2/3-TEa2/3-AId1-TEa4-PERI2/3
PI 40:7
PS 42:9
PI 40:7 × PS 42:9
Induseum griseum
PI 40:7
LPC 16:0
PI 40:7 × LPC 16:0
COApm-HATA
PS 42:9
SM 31:1;O2
PS 42:9 × SM 31:1;O2
Vascular organ of the lamina terminalis_2
SM 42:1;O2
SM 34:1;O2
SM 42:1;O2 × SM 34:1;O2
ORBl6a-AUDd6b-ORBl6b-ILA6b-VISpor6b
HexCer 42:2;O2
HexCer 42:1;O2
HexCer 42:2;O2 × HexCer 42:1;O2
Anterior cingulate area, dorsal part, layer 6b_1
HexCer 42:2;O2
HexCer 42:1;O2
HexCer 42:2;O2 × HexCer 42:1;O2
TR-COApm-ECT2/3-ENTl3-AIp5
PI 40:7
SM 31:1;O2
PI 40:7 × SM 31:1;O2
AId1-AIp5-AIp1-ENTm6-AIp6a
PS 42:9
PC 32:0
PS 42:9 × PC 32:0
DG-po-ILA1-DG-sg-CA1-DG-mo
LPC 16:0
SM 36:2;O2
LPC 16:0 × SM 36:2;O2
VISpor5-VISpor4-VISam2/3-AUDpo5-VISl5
PI 40:7
SM 31:1;O2
PI 40:7 × SM 31:1;O2
TEa1-AId1-AIv1-AUDv1-ECT1
PS 42:9
SM 31:1;O2
PS 42:9 × SM 31:1;O2
corpus callosum, extreme capsule
SM 36:2;O2
PC 31:0 PE 34:0
SM 36:2;O2 × PC 31:0 PE 34:0
ProS-DR-ORBm6a-PR-PL5
PI 40:8
SM 36:2;O2
PI 40:8 × SM 36:2;O2
GU1-COApm-PAA-HATA-AId1
PS 42:9
PI 40:7
PS 42:9 × PI 40:7
RSPv2/3-RSPv5-RSPv1-PRE-SSp-un4
PC 35:0
SM 38:1;O2
PC 35:0 × SM 38:1;O2
ENTl2-ENTl1-PERI1-AIp1-COAa
PS 42:9
SM 31:1;O2
PS 42:9 × SM 31:1;O2

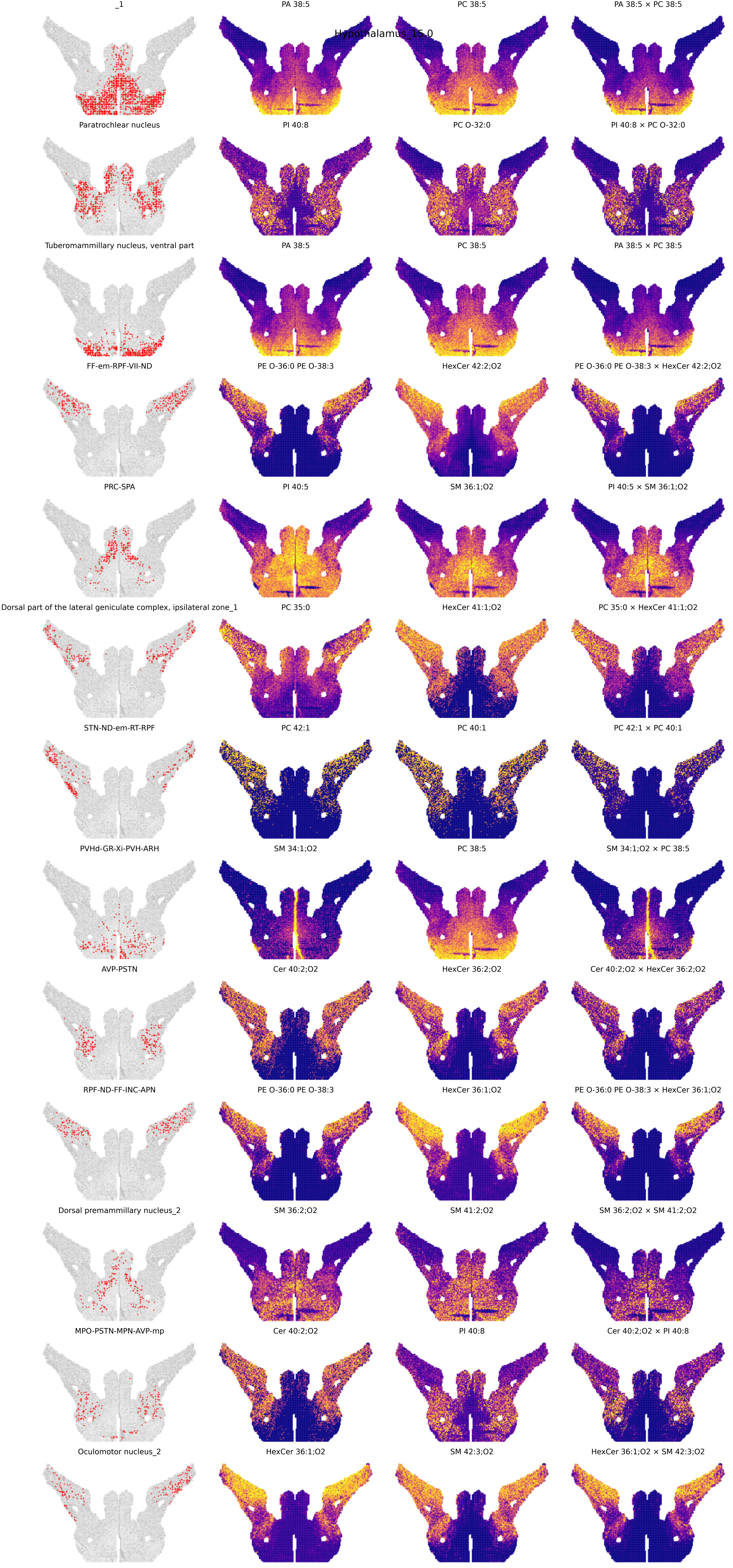
Hypothalamus_15.0
_1
PA 38:5
PC 38:5
PA 38:5 × PC 38:5
Paratrochlear nucleus
PI 40:8
PC O-32:0
PI 40:8 × PC O-32:0
Tuberomammillary nucleus, ventral part
PA 38:5
PC 38:5
PA 38:5 × PC 38:5
FF-em-RPF-VII-ND
PE O-36:0 PE O-38:3
HexCer 42:2;O2
PE O-36:0 PE O-38:3 × HexCer 42:2;O2
PRC-SPA
PI 40:5
SM 36:1;O2
PI 40:5 × SM 36:1;O2
Dorsal part of the lateral geniculate complex, ipsilateral zone_1
PC 35:0
HexCer 41:1;O2
PC 35:0 × HexCer 41:1;O2
STN-ND-em-RT-RPF
PC 42:1
PC 40:1
PC 42:1 × PC 40:1
PVHd-GR-Xi-PVH-ARH
SM 34:1;O2
PC 38:5
SM 34:1;O2 × PC 38:5
AVP-PSTN
Cer 40:2;O2
HexCer 36:2;O2
Cer 40:2;O2 × HexCer 36:2;O2
RPF-ND-FF-INC-APN
PE O-36:0 PE O-38:3
HexCer 36:1;O2
PE O-36:0 PE O-38:3 × HexCer 36:1;O2
Dorsal premammillary nucleus_2
SM 36:2;O2
SM 41:2;O2
SM 36:2;O2 × SM 41:2;O2
MPO-PSTN-MPN-AVP-mp
Cer 40:2;O2
PI 40:8
Cer 40:2;O2 × PI 40:8
Oculomotor nucleus_2
HexCer 36:1;O2
SM 42:3;O2
HexCer 36:1;O2 × SM 42:3;O2

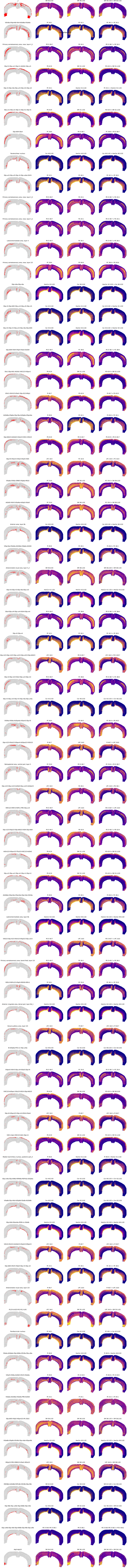

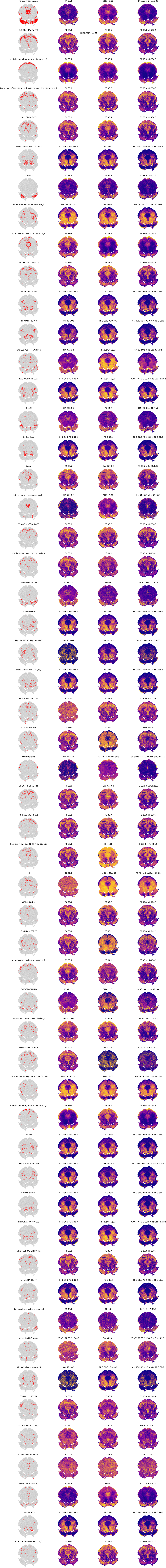
Midbrain_17.0

Olfactory areas_1.0

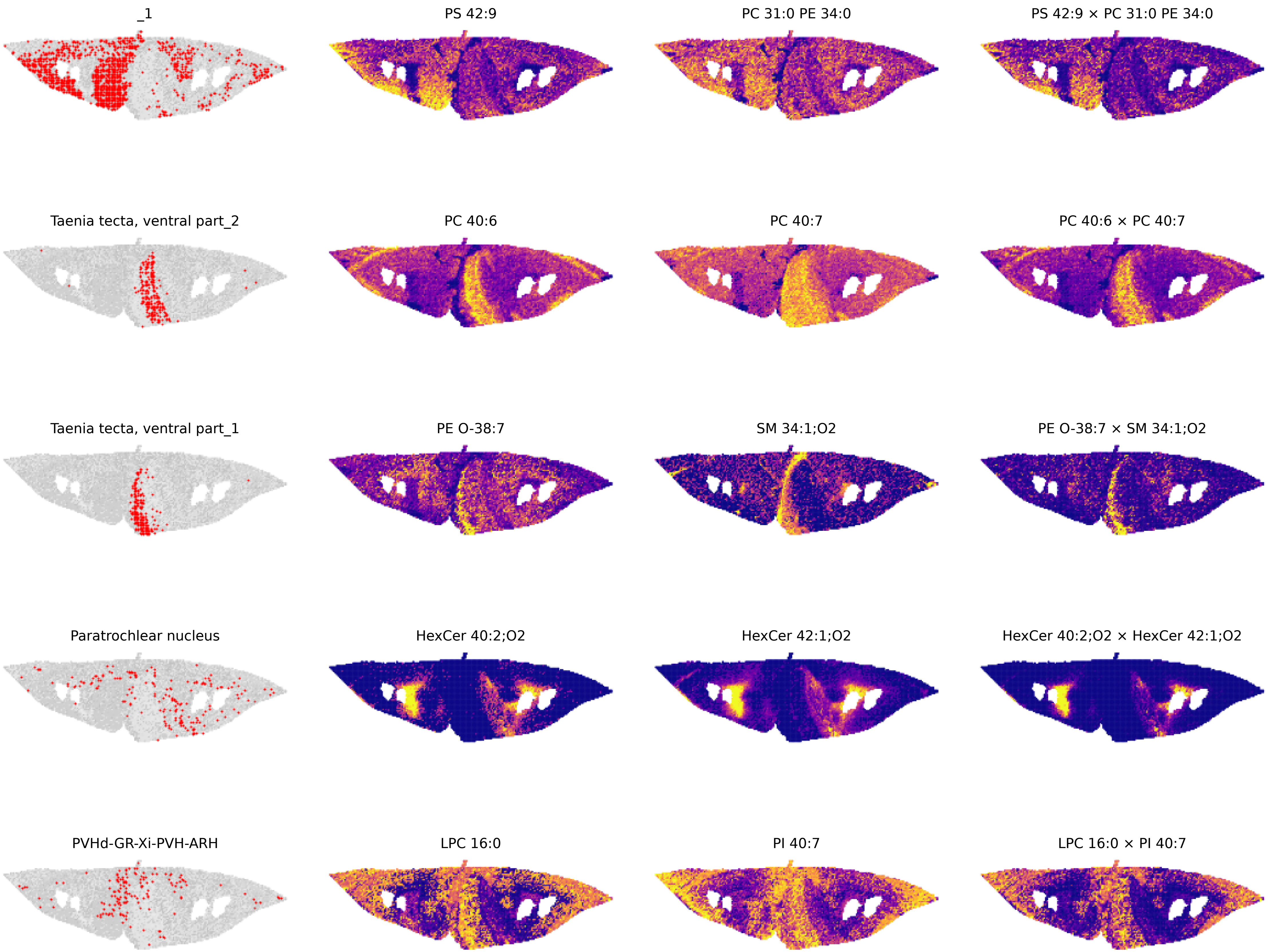

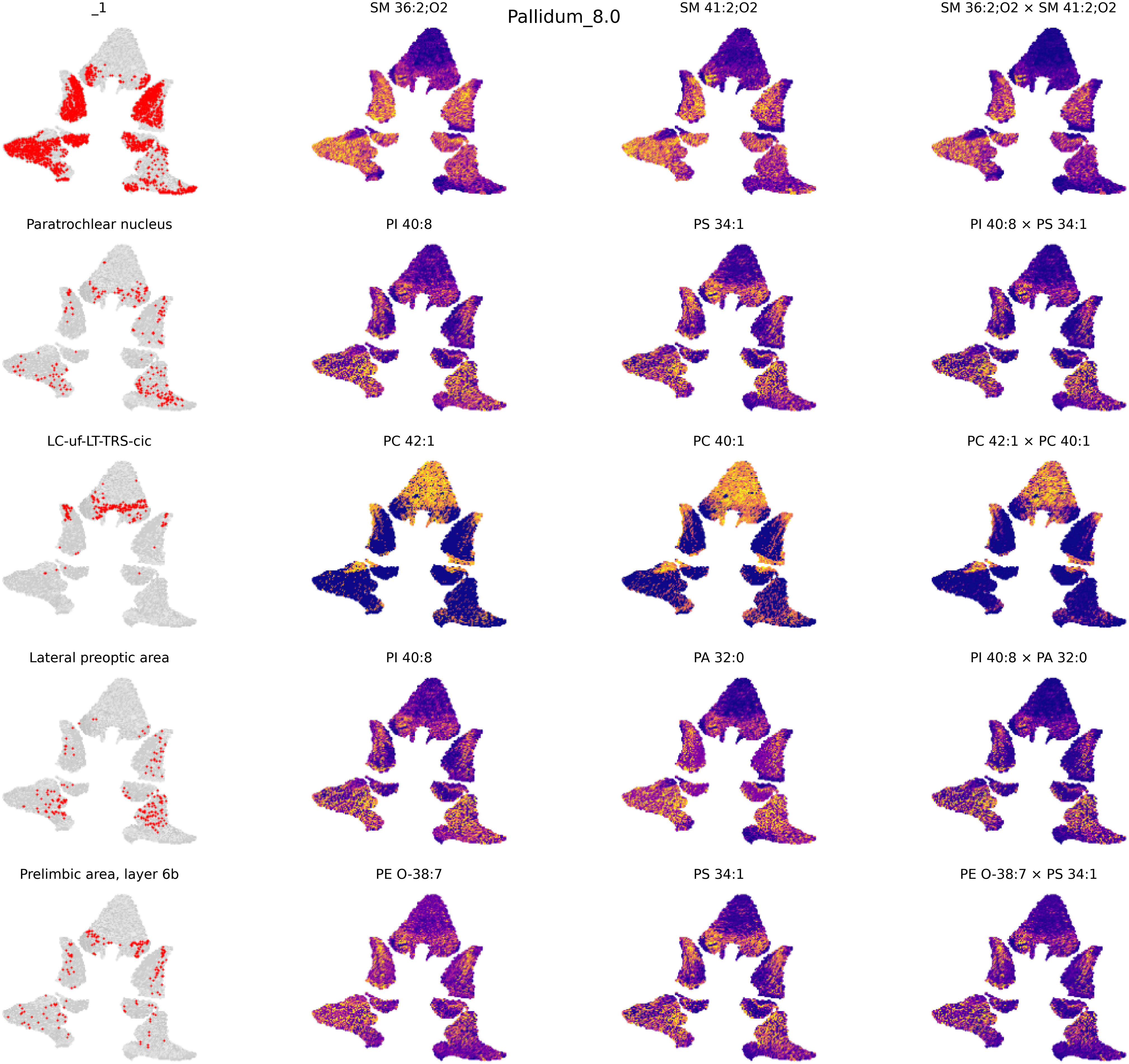
Pallidum_8.0
_1
SM 36:2;O2
SM 41:2;O2
SM 36:2;O2 × SM 41:2;O2
Paratrochlear nucleus
PI 40:8
PS 34:1
PI 40:8 × PS 34:1
LC-uf-LT-TRS-cic
PC 42:1
PC 40:1
PC 42:1 × PC 40:1
Lateral preoptic area
PI 40:8
PA 32:0
PI 40:8 × PA 32:0
Prelimbic area, layer 6b
PE O-38:7
PS 34:1
PE O-38:7 × PS 34:1

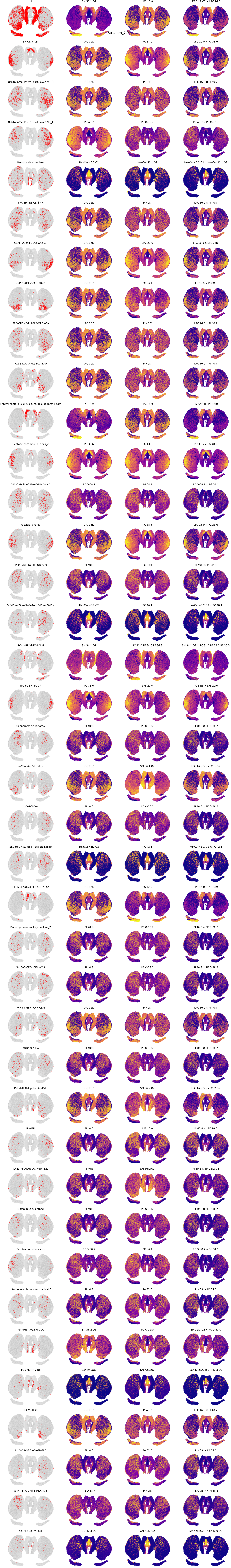

Striatum_7.0
_1
SM 31:1;O2
LPC 16:0
SM 31:1;O2 × LPC 16:0
SH-CEAc-LSr
LPC 16:0
PC 38:6
LPC 16:0 × PC 38:6
Orbital area, lateral part, layer 2/3_3
LPC 16:0
PI 40:7
LPC 16:0 × PI 40:7
Orbital area, lateral part, layer 2/3_1
PC 40:7
PE O-38:7
PC 40:7 × PE O-38:7
Parabrachial nucleus
HexCer 40:2;O2
HexCer 41:1;O2
HexCer 40:2;O2 × HexCer 41:1;O2
PRC-SPA-FE-CEAl-RH
LPC 16:0
PI 40:7
LPC 16:0 × PI 40:7
CEAc-DG-mo-BLAa-CA2-CP
LPC 16:0
LPC 22:6
LPC 16:0 × LPC 22:6
IG-PL1-ACAv1-XI-ORBvl5
LPC 16:0
PG 36:1
LPC 16:0 × PG 36:1
PRC-ORBvl5-RH-SPA-ORBm6a
LPC 16:0
PI 40:7
LPC 16:0 × PI 40:7
PL2/3-ILA2/3-PL5-PL1-ILA5
LPC 16:0
PI 40:7
LPC 16:0 × PI 40:7
Lateral septal nucleus, caudal (caudodorsal) part
PS 42:9
LPC 16:0
PS 42:9 × LPC 16:0
Septohippocampal nucleus_2
PC 38:6
PG 40:6
PC 38:6 × PG 40:6
SPA-ORBvl6a-SPFm-ORBvl5-IMD
PE O-38:7
PG 34:1
PE O-38:7 × PG 34:1
Fasciola cinerea
LPC 16:0
PC 38:6
LPC 16:0 × PC 38:6
SPFm-SPA-ProS-IPI-ORBvl6a
PI 40:8
PG 34:1
PI 40:8 × PG 34:1
VISrl6a-VISpm6b-Pa4-AUDd6a-VISal6a
HexCer 40:2;O2
PC 40:1
HexCer 40:2;O2 × PC 40:1
PVHd-GR-XI-PVH-ARH
SM 34:1;O2
PC 31:0 PE 34:0 PE 36:3
SM 34:1;O2 × PC 31:0 PE 34:0 PE 36:3
IPC-FC-SH-IPL-CP
PC 38:6
LPE 22:6
PC 38:6 × LPE 22:6
Subparafascicular area
PI 40:8
PE O-38:7
PI 40:8 × PE O-38:7
XI-CEAl-ACB-BST-LSx
LPC 16:0
SM 36:1;O2
LPC 16:0 × SM 36:1;O2
IPDM-SPFm
PI 40:8
PE O-38:7
PI 40:8 × PE O-38:7
SSp-tr6b-VISam6a-IPDM-ccs-SSs6b
HexCer 41:1;O2
PC 42:1
HexCer 41:1;O2 × PC 42:1
PERI2/3-AId2/3-PERI5-LSc-LSr
LPC 16:0
PS 42:9
LPC 16:0 × PS 42:9
Dorsal premammillary nucleus_2
PI 40:8
PE O-38:7
PI 40:8 × PE O-38:7
SH-CA2-CEAl-CEAi-CA3
PI 40:8
PE O-38:7
PI 40:8 × PE O-38:7
PVHd-PVH-XI-AHN-CEAl
LPC 16:0
PI 40:7
LPC 16:0 × PI 40:7
AUDpo6b-IPA
PI 40:8
PE O-38:7
PI 40:8 × PE O-38:7
PVHd-AHN-Alp6b-ILA5-PVH
LPC 16:0
SM 36:2;O2
LPC 16:0 × SM 36:2;O2
IPA-IPN
PI 40:8
LPE 18:0
PI 40:8 × LPE 18:0
ILA6a-PS-Alp6b-ACAv6b-PL6a
PI 40:8
SM 36:2;O2
PI 40:8 × SM 36:2;O2
Dorsal nucleus raphe
PI 40:8
PE O-38:7
PI 40:8 × PE O-38:7
Parabigeminal nucleus
PE O-38:7
PG 34:1
PE O-38:7 × PG 34:1
Interpeduncular nucleus, apical_2
PI 40:8
PA 32:0
PI 40:8 × PA 32:0
PS-AHN-AIv6a-XI-CLA
SM 36:2;O2
PC O-32:0
SM 36:2;O2 × PC O-32:0
LC-sf-LT-TRS-cic
Cer 40:2;O2
SM 42:3;O2
Cer 40:2;O2 × SM 42:3;O2
ILA2/3-ILA1
LPC 16:0
PI 40:7
LPC 16:0 × PI 40:7
ProS-DR-ORBm6a-PR-PL5
PI 40:8
PA 32:0
PI 40:8 × PA 32:0
SPFm-SPA-ORBl5-IMD-AIv5
PE O-38:7
PI 40:8
PE O-38:7 × PI 40:8
CS-NI-SLD-AVP-CLI
SM 42:3;O2
Cer 40:0;O2
SM 42:3;O2 × Cer 40:0;O2

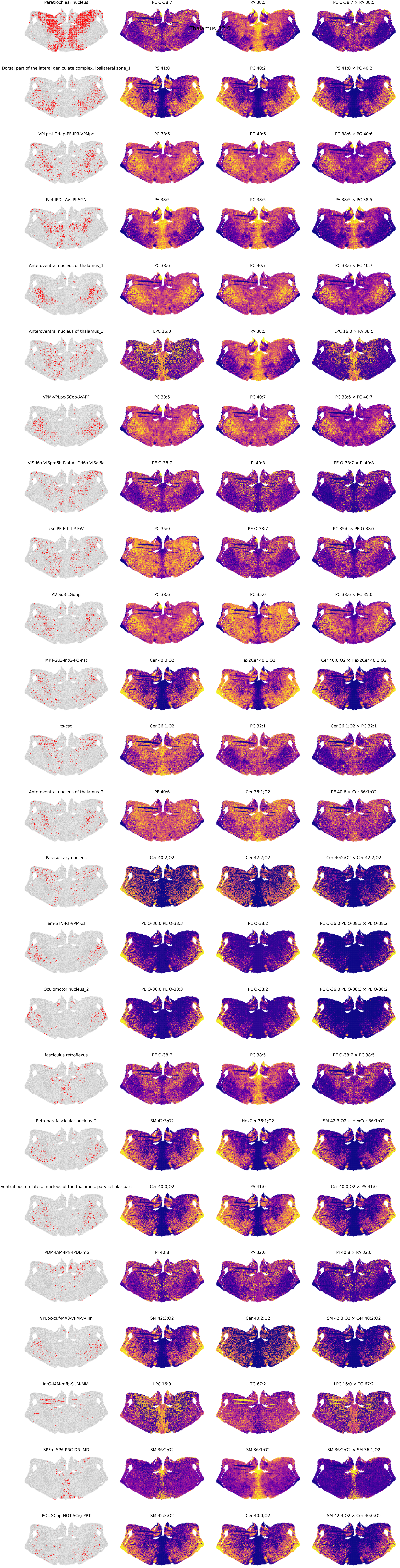
Thalamus_72.0
Paratrochlear nucleus
PE O-38:7
PA 38:5
PE O-38:7 × PA 38:5
Dorsal part of the lateral geniculate complex, ipsilateral zone_1
PS 41:0
PC 40:2
PS 41:0 × PC 40:2
VPLpc-LGd-ip-PF-IPR-VPMpc
PC 38:6
PG 40:6
PC 38:6 × PG 40:6
Pa4-IPDL-AV-IPI-SGN
PA 38:5
PC 38:5
PA 38:5 × PC 38:5
Anteroventral nucleus of thalamus_1
PC 38:6
PC 40:7
PC 38:6 × PC 40:7
Anteroventral nucleus of thalamus_3
LPC 16:0
PA 38:5
LPC 16:0 × PA 38:5
VPM-VPLpc-SCop-AV-PF
PC 38:6
PC 40:7
PC 38:6 × PC 40:7
VISrl6a-VISpm6b-Pa4-AUDd6a-VISal6a
PE O-38:7
PI 40:8
PE O-38:7 × PI 40:8
csc-PF-Eth-LP-EW
PC 35:0
PE O-38:7
PC 35:0 × PE O-38:7
AV-Su3-LGd-ip
PC 38:6
PC 35:0
PC 38:6 × PC 35:0
MPT-Su3-IntG-PO-nst
Cer 40:0;O2
Hex2Cer 40:1;O2
Cer 40:0;O2 × Hex2Cer 40:1;O2
ts-csc
Cer 36:1;O2
PC 32:1
Cer 36:1;O2 × PC 32:1
Anteroventral nucleus of thalamus_2
PE 40:6
Cer 36:1;O2
PE 40:6 × Cer 36:1;O2
Parasolitary nucleus
Cer 40:2;O2
Cer 42:2;O2
Cer 40:2;O2 × Cer 42:2;O2
em-STN-RT-VPM-ZI
PE O-36:0 PE O-38:3
PE O-38:2
PE O-36:0 PE O-38:3 × PE O-38:2
Oculomotor nucleus_2
PE O-36:0 PE O-38:3
PE O-38:2
PE O-36:0 PE O-38:3 × PE O-38:2
fasciculus retroflexus
PE O-38:7
PC 38:5
PE O-38:7 × PC 38:5
Retroparafascicular nucleus_2
SM 42:3;O2
HexCer 36:1;O2
SM 42:3;O2 × HexCer 36:1;O2
Ventral posterolateral nucleus of the thalamus, parvicellular part
Cer 40:0;O2
PS 41:0
Cer 40:0;O2 × PS 41:0
IPDM-IAM-IPN-IPDL-mp
PI 40:8
PA 32:0
PI 40:8 × PA 32:0
VPLpc-cuf-MA3-VPM-vVIIIn
SM 42:3;O2
Cer 40:2;O2
SM 42:3;O2 × Cer 40:2;O2
IntG-IAM-mfb-SUM-MMI
LPC 16:0
TG 67:2
LPC 16:0 × TG 67:2
SPFm-SPA-PRC-DR-IMD
SM 36:2;O2
SM 36:1;O2
SM 36:2;O2 × SM 36:1;O2
POL-SCop-NOT-SCig-PPT
SM 42:3;O2
Cer 40:0;O2
SM 42:3;O2 × Cer 40:0;O2

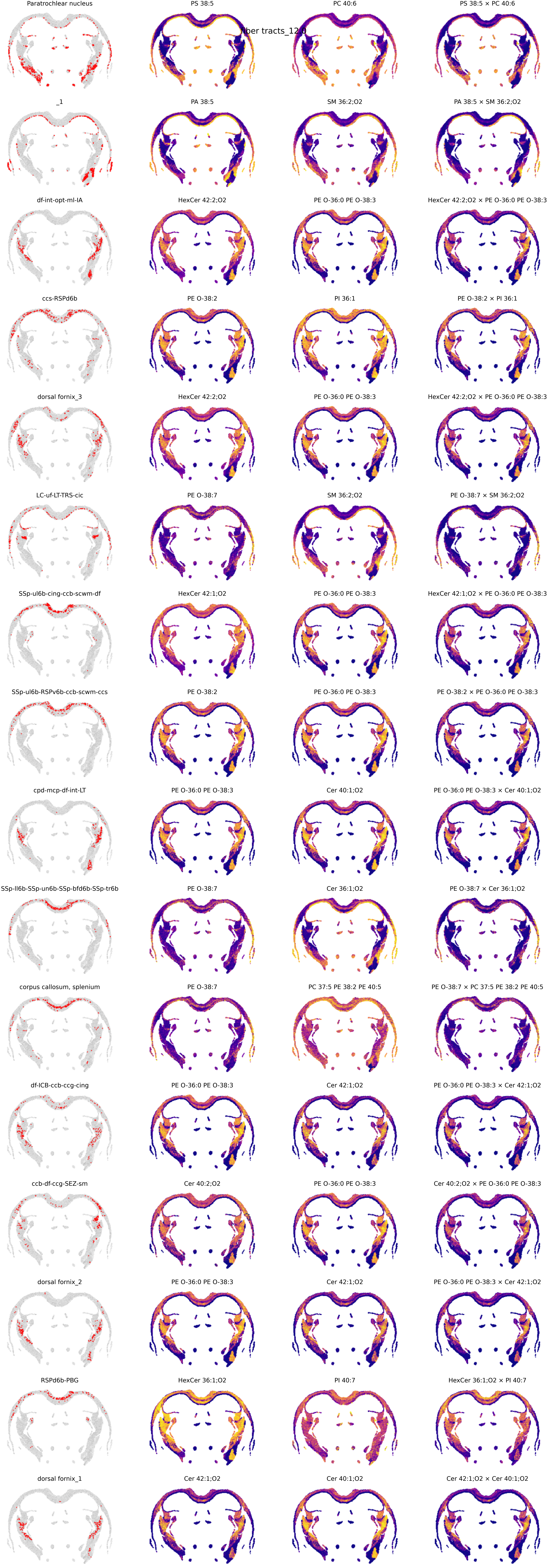
fiber tracts_12.0
Paratrochlear nucleus
PS 38:5
PC 40:6
PS 38:5 × PC 40:6
_1
PA 38:5
SM 36:2;O2
PA 38:5 × SM 36:2;O2
df-int-opt-ml-IA
HexCer 42:2;O2
PE O-36:0 PE O-38:3
HexCer 42:2;O2 × PE O-36:0 PE O-38:3
ccs-RSPd6b
PE O-38:2
PI 36:1
PE O-38:2 × PI 36:1
dorsal fornix_3
HexCer 42:2;O2
PE O-36:0 PE O-38:3
HexCer 42:2;O2 × PE O-36:0 PE O-38:3
LC-uf-LT-TRS-cic
PE O-38:7
SM 36:2;O2
PE O-38:7 × SM 36:2;O2
SSp-ul6b-cing-ccb-scwm-df
HexCer 42:1;O2
PE O-36:0 PE O-38:3
HexCer 42:1;O2 × PE O-36:0 PE O-38:3
SSp-ul6b-RSPv6b-ccb-scwm-ccs
PE O-38:2
PE O-36:0 PE O-38:3
PE O-38:2 × PE O-36:0 PE O-38:3
cpd-mcp-df-int-LT
PE O-36:0 PE O-38:3
Cer 40:1;O2
PE O-36:0 PE O-38:3 × Cer 40:1;O2
SSp-ll6b-SSp-un6b-SSp-bfd6b-SSp-tr6b
PE O-38:7
Cer 36:1;O2
PE O-38:7 × Cer 36:1;O2
corpus callosum, splenium
PE O-38:7
PC 37:5 PE 38:2 PE 40:5
PE O-38:7 × PC 37:5 PE 38:2 PE 40:5
df-ICB-ccb-ccg-cing
PE O-36:0 PE O-38:3
Cer 42:1;O2
PE O-36:0 PE O-38:3 × Cer 42:1;O2
ccb-df-ccg-SEZ-sm
Cer 40:2;O2
PE O-36:0 PE O-38:3
Cer 40:2;O2 × PE O-36:0 PE O-38:3
dorsal fornix_2
PE O-36:0 PE O-38:3
Cer 42:1;O2
PE O-36:0 PE O-38:3 × Cer 42:1;O2
RSPd6b-PBG
HexCer 36:1;O2
PI 40:7
HexCer 36:1;O2 × PI 40:7
dorsal fornix_1
Cer 42:1;O2
Cer 40:1;O2
Cer 42:1;O2 × Cer 40:1;O2

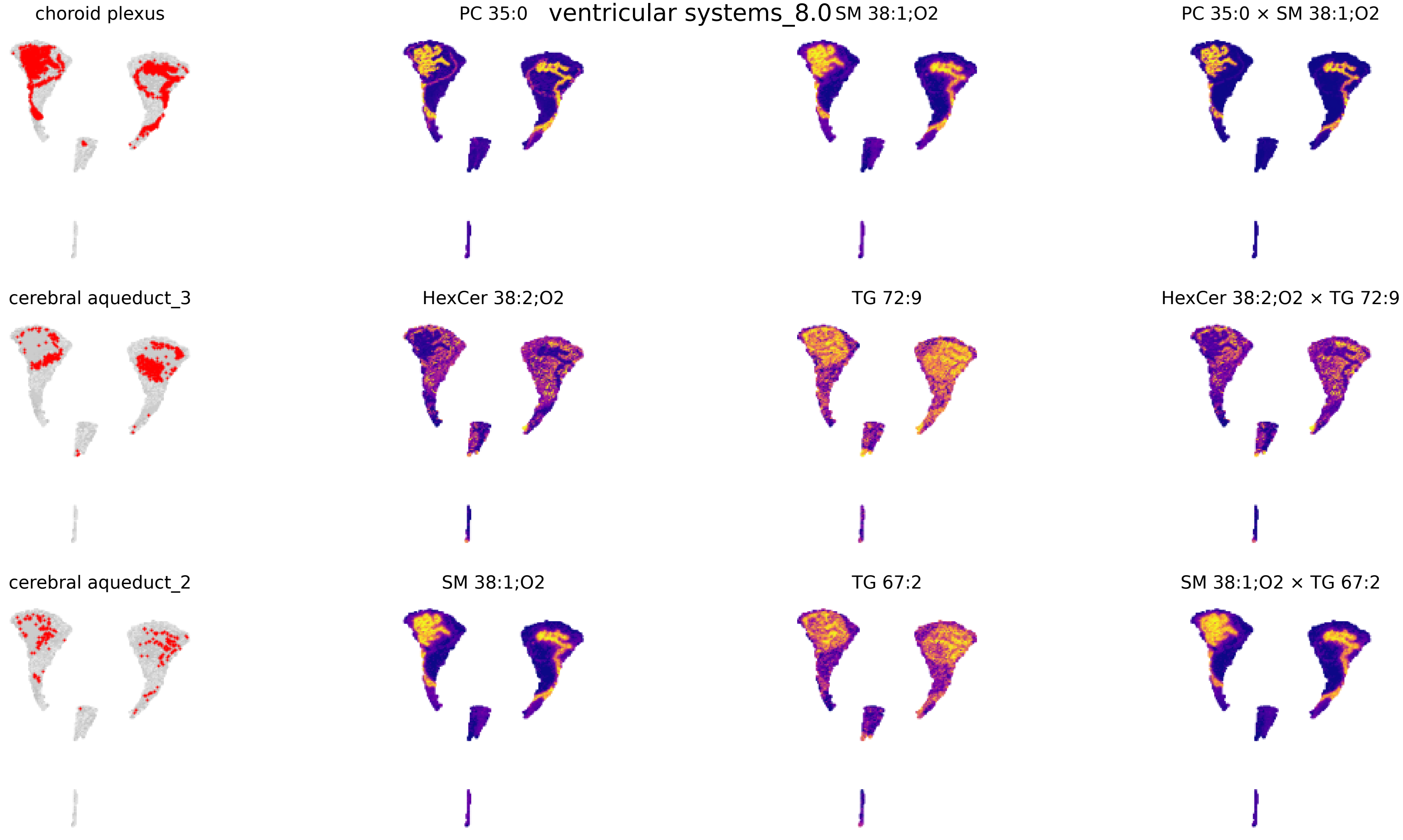

ventricular systems_8.0
choroid plexus
PC 35:0
SM 38:1;O2
PC 35:0 × SM 38:1;O2
cerebral aqueduct_3
HexCer 38:2;O2
TG 72:9
HexCer 38:2;O2 × TG 72:9
cerebral aqueduct_2
SM 38:1;O2
TG 67:2
SM 38:1;O2 × TG 67:2
