# Supplementary material for: The lipidomic architecture of the mouse brain
Source: Nature. 2026 Sep 23;658(8134):205–17. doi: 10.1038/s41586-026-11050-0 (PMC13626954; doi:10.1038/s41586-026-11050-0)

Lipizone-informed brain dissection tables for lipidomic studies

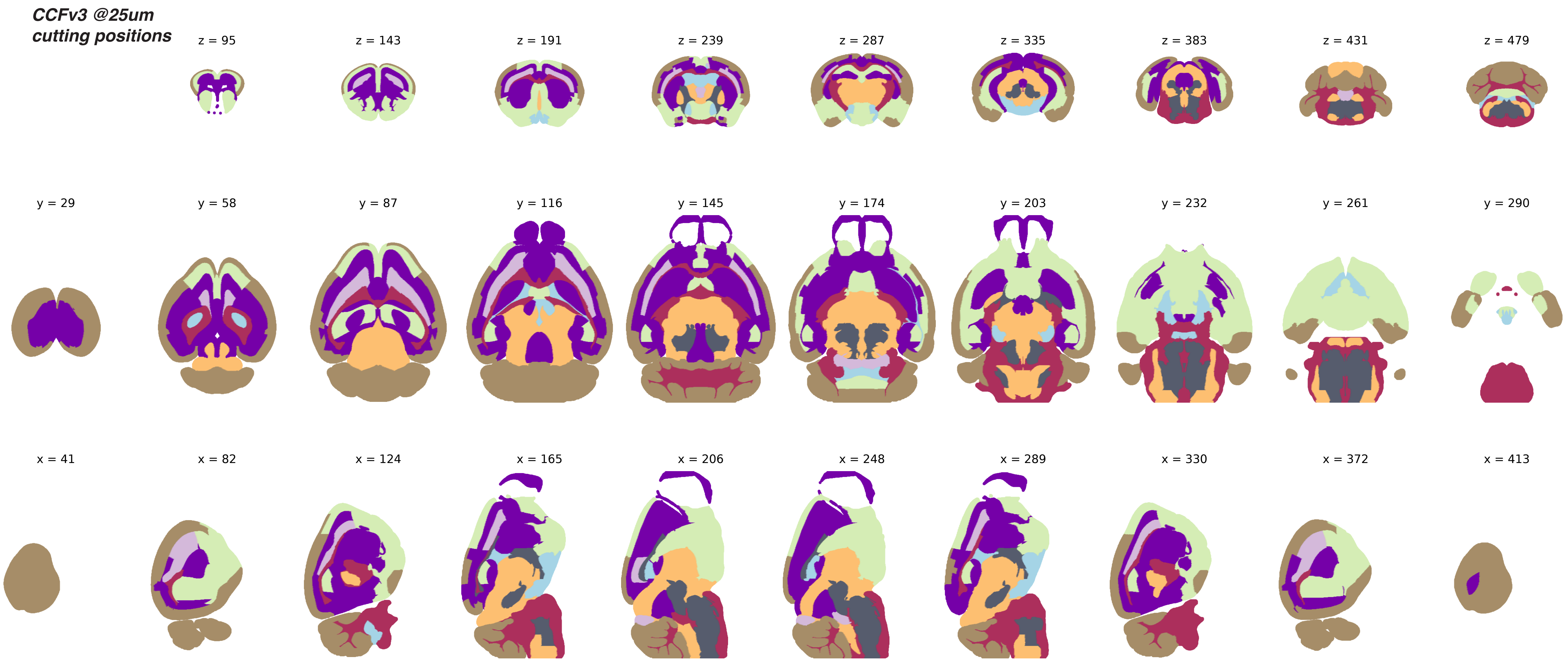

Supplement: Supplementary file 5 [file 41586_2026_11050_MOESM3_ESM.zip › 2025-06-14969CD-s3/LaManno_SuppTable9.pdf]
