# Supplementary material for: The lipidomic architecture of the mouse brain
Source: Nature. 2026 Sep 23;658(8134):205–17. doi: 10.1038/s41586-026-11050-0 (PMC13626954; doi:10.1038/s41586-026-11050-0)
Supplement: Supplementary file 5 [file 41586_2026_11050_MOESM3_ESM.zip › 2025-06-14969CD-s3/Supplementary_Tables_guide.docx]

## Supplementary Tables

**Supplementary Table 1.** Quantitative LC-MS/MS lipidomics. **Tab QuantitativeLCMS**: whole-brain quantitative LC-MS/MS lipidomics, male and female adult mice. **Tab CallosumPregnancyQuantLCMS**: bulk lipidomics for the microdissected corpus callosum comparing N=4 pregnant female and N=4 control female brains. **Tab HILIC_column**: HILIC-ESI-MS/MS lipid quantifications. **Tab ReversePhase_column**: reverse-phase LC-MS/MS lipid quantifications. **Tab QualitativeTANDEM_Goslin**: Goslin-canonicalized lipid table from TANDEM MS/MS. **Tab Fitzner_Goslin**: Goslin-canonicalized lipid table for Fitzner et al. bulk lipidomics. **Tab AnalyticalChemistry_PosIonMode**: lipids detected by Vandenbosch et al. MALDI-MSI, adapted from the original study.

**Supplementary Table 2.** Annotation tables for MALDI-MSI peaks. **Tab a**: annotation table for the 172 m/z peaks used throughout the study, with confidence-weighted scores, adducts, and annotation details. **Tab b**: annotation table for the 1,400 m/z peaks collected, with confidence-weighted scores, adducts, annotation details, and Moran’s I per acquisition.

**Supplementary Table 3.** Manual annotation of gray-matter *lipizones* along the bipartite hierarchy, based on anatomical inspection.

**Supplementary Table 4.** Lipidomes of the 539 terminal *lipizones*: mean normalized abundance per lipid across the 172 annotated species.

**Supplementary Table 5.** **Tab a**: region-average lipidomes across Allen Brain Atlas regions. **Tab b**: region-average lipid programs across Allen Brain Atlas regions.

**Supplementary Table 6.** Within-region lipid markers: for each Allen Brain Atlas region, the lipid species that discriminate its subregions. Intended to support regionally focused neurometabolic studies.

**Supplementary Table 7.** *Lipizone*–cell-type spatial overlap pairs, computed using the Yao et al. (2023) cell-type territories.

**Supplementary Table 8.** Inferred lipidomes of Allen subclass-level cell types, derived by non-negative elastic-net deconvolution of MALDI pixels against MERFISH cell-type maps.

**Supplementary Table 9.** *Lipizone*-informed anatomical dissection guide, listing Allen Brain Atlas regions and CCF coordinates to be co-sampled to obtain lipidomically coherent tissue.

**Supplementary Table 10.** Curated reagent–product–enzyme reaction list for the 172 annotated lipids, integrating LINEX2 output with manual curation. Used for the enzyme–lipid correlation analysis and the CORNETO signaling-to-lipid model.

**Supplementary Table 11.** Inventory of raw datasets released in this study, with Zenodo links.
